# Systematic Review and Inventory of Theory of Mind Measures for Young Children

**DOI:** 10.3389/fpsyg.2019.02905

**Published:** 2020-01-15

**Authors:** Cindy Beaudoin, Élizabel Leblanc, Charlotte Gagner, Miriam H. Beauchamp

**Affiliations:** ^1^Department of Psychology, University of Montreal, Montreal, QC, Canada; ^2^Sainte-Justine Hospital Research Center, Montreal, QC, Canada

**Keywords:** theory of mind, systematic review, childhood, psychometrics, assessment, preschool

## Abstract

Theory of mind (TOM), the ability to infer mental states to self and others, has been a pervasive research theme across many disciplines including developmental, educational, neuro-, and social psychology, social neuroscience and speech therapy. TOM abilities have been consistently linked to markers of social adaptation and have been shown to be affected in a broad range of clinical conditions. Despite the wealth and breadth of research dedicated to TOM, identifying appropriate assessment tools for young children remains challenging. This systematic review presents an inventory of TOM measures for children aged 0–5 years and provides details on their content and characteristics. Electronic databases (1983–2019) and 9 test publisher catalogs were systematically reviewed. In total, 220 measures, identified within 830 studies, were found to assess the understanding of seven categories of mental states and social situations: emotions, desires, intentions, percepts, knowledge, beliefs and mentalistic understanding of non-literal communication, and pertained to 39 types of TOM sub-abilities. Information on the measures' mode of presentation, number of items, scoring options, and target populations were extracted, and psychometric details are listed in summary tables. The results of the systematic review are summarized in a visual framework “Abilities in Theory of Mind Space” (ATOMS) which provides a new taxonomy of TOM sub-domains. This review highlights the remarkable variety of measures that have been created to assess TOM, but also the numerous methodological and psychometric challenges associated with developing and choosing appropriate measures, including issues related to the limited range of sub-abilities targeted, lack of standardization across studies and paucity of psychometric information provided.

## Introduction

Consolidating appropriate social skills is an essential part of typical development, as it allows individuals to establish and maintain satisfying social relationships and promotes community adaptation across the lifespan (Cacioppo, 2002). The emergence of social skills is a complex developmental process involving the maturation of a broad range of underlying cognitive functions, referred to as “social cognition” (Beauchamp and Anderson, 2010). Among these, Theory of Mind (TOM) has been a central focus of developmental and social psychology, as well as speech therapy (Byom and Turkstra, 2012) since Premack first coined the term TOM in the 1970s, referring to the ability to impute mental states to self and others, including desires, knowledge, beliefs, and intentions, in order to predict behavior (Premack and Woodruff, 1978). In order to display flexible and explicit TOM, it was acknowledged that children must have the capacity to construct different abstract representations of reality, and to navigate between them to distinguish their metal states from those of others using various cues, therefore acting as “theorists” (Wimmer and Perner, 1983). This field has since been one of the most studied in developmental cognitive science (Sabbagh and Paulus, 2018). More recently, TOM and other social cognitive constructs have also attracted attention within the field of social neuroscience, which has generated a large body of consensual literature regarding the brain networks underlying TOM (Gallagher and Frith, 2003; Frith and Frith, 2006; Blakemore, 2008; Bellerose et al., 2011; Bird and Viding, 2014).

Children who have good TOM generally display markers of social adaptation, such as better communication skills, better quality social relationships, increased peer popularity and higher academic achievement (Binnie, 2005; Fink et al., 2015; Slaughter, 2015; Slaughter et al., 2015; Imuta et al., 2016). Conversely, poorer TOM has been identified in a number of conditions and contexts characterized by altered social functioning, such as autism spectrum disorders (Yirmiya et al., 1998; Shaked and Yirmiya, 2004; Senju, 2012; Chung et al., 2014; Kimhi, 2014; Leekam, 2016), language impairment (Stanzione and Schick, 2014), attention-deficit/hyperactivity disorder (Bora and Pantelis, 2016), Tourette's syndrome (Eddy and Cavanna, 2013), childhood maltreatment (Luke and Banerjee, 2013; Benarous et al., 2015), conduct disorders (Anastassiou-Hadjicharalambous and Warden, 2008; Poletti and Adenzalo, 2013), anorexia nervosa (Bora and Köse, 2016), schizophrenia (Brune, 2005; Sprong et al., 2007; Bora et al., 2009; Cermolacce et al., 2011; Biedermann et al., 2012; Chung et al., 2014; Martin et al., 2014; Song et al., 2015; Healey et al., 2016), traumatic brain injury (Snodgrass and Knott, 2006; Walz et al., 2010; Dennis et al., 2012; McDonald, 2013; Bellerose et al., 2017), epilepsy (Bora and Meletti, 2016; Stewart et al., 2016), neurofibromatosis (Payne et al., 2016), and Fragile X syndrome (Turkstra et al., 2014).

Efforts to understand the role of TOM in normative development and in clinical conditions are ongoing. Furthering this knowledge relies on the use of validated, developmentally appropriate assessment tools, especially given that social cognition is now included in the assessment recommendations of the Diagnostic and Statistical Manual of Mental Disorders (DSM-V; American Psychiatric Association, 2013). Although a surfeit of measures have been developed to test TOM (particularly in the field of cognitive science), identifying the best measure for particular clinical or research needs is not an easy enterprise. Evaluating TOM presents many challenges, some of which are related to the numerous and varied definitions and conceptualisations of TOM that have been proposed (Premack and Woodruff, 1978; Wimmer and Perner, 1983; Leslie, 1987; Tager-Flusberg and Sullivan, 2000; Abu-Akel and Shamay-Tsoory, 2011; Dennis et al., 2013; Bird and Viding, 2014; Westby, 2014; Asakura and Inui, 2016; Happé et al., 2017), the changeable manifestations of TOM at different developmental stages (Wellman et al., 2011; Carlson et al., 2013; Slaughter, 2015), and the psychometric limitations associated with some measures (Mayes et al., 1996; Brune, 2001; Hutchins et al., 2008a; Carlson et al., 2013; Hiller et al., 2014).

### Defining Theory of Mind and Distinguishing It From Other Social Constructs

TOM is a complex construct encompassing a range of abilities, which are variably targeted as a function of the measurement tool chosen (German and Cohen, 2012). Each definition or theory provides slightly different conceptions regarding the specificity of TOM and what behavioral manifestations it reflects (Premack and Woodruff, 1978; Wimmer and Perner, 1983; Leslie, 1987; Tager-Flusberg and Sullivan, 2000; Abu-Akel and Shamay-Tsoory, 2011; Dennis et al., 2013; Bird and Viding, 2014; Westby, 2014; Asakura and Inui, 2016; Happé et al., 2017). Nonetheless, it is generally accepted that TOM represents a set of cognitive skills that enable reasoning about cognitive (e.g., beliefs) or affective (e.g., emotions) mental states.

In this review, the Self to Other Model of Empathy (SOME; Bird and Viding, 2014) is used as a framework to define TOM and set the inclusion and exclusion criteria for the literature search. The SOME is a comprehensive model based on empirical data from clinical and neuroimaging studies (Bird and Viding, 2014). It depicts how social cognitive constructs, such as TOM, come together to determine empathic behavior rather than focusing solely on internal TOM processes. Importantly, SOME distinguishes TOM from empathy: TOM is defined as a person's cognitive representation of self and other's mental states, whereas empathy is defined as an emotional contagion caused by exposure to another's emotion, while being conscious that this emotional state is experienced by the other (Bird and Viding, 2014). In the model, TOM is also differentiated from the “affective cue classification system,” a lower perceptual system responsible for processing and categorizing stimuli signaling affective states, such as facial emotions and tones of voice. The SOME model further posits that TOM is distinct from a “situation understanding system” responsible for processing situational cues and deducing or associating estimated emotional states of others based upon situational cues (e.g., people dressed in black at a cemetery = funeral = sadness) (Bird and Viding, 2014). The model is therefore useful for setting boundaries between TOM and other closely related social cognitive constructs, and was used in the current review to distinguish central TOM measures from those more distally related to TOM.

In addition to using a clear definition of TOM to identify and document relevant assessment tools, the construct of TOM should be distinguished from other abilities that, though they may build or rely on TOM, are better represented by other social cognitive functions. For example, many overt prosocial and self-promoting behaviors rely on TOM, but can be more directly assessed through targeted measures, such as those that document cooperation, adherence to social norms, lies and manipulative interpersonal tactics (Baurain and Nader-Grosbois, 2013; Slaughter, 2015). The way in which TOM is used in everyday social interactions also depends on other discrete factors, such as temperament, life experiences, integration of social values and executive functioning (Beauchamp and Anderson, 2010; Slaughter, 2015; Vera-Estay et al., 2015). As a result, in order to identify assessment measures that specifically target TOM, it is also critical to choose those that elicit TOM specifically, rather than those that evaluate more complex social cognitive skills, such as moral reasoning (Vera-Estay et al., 2015) and strategic social decision making (Steinmann et al., 2014), for example.

There are developmental considerations that should also be taken into account to constrain our search to the most unambiguous forms of TOM. There is ongoing debate around the definition of TOM with regards to which emerging social skills in infancy are considered direct, early manifestations of TOM, and which are distinct cognitive precursors allowing TOM to arise (Carlson et al., 2013). While the question of the first measurable manifestations of TOM remains to be answered theoretically and empirically, current literature and most authors suggest that early social skills, such as imitation, gaze following, pointing, and joint attention, may reflect, at most, more automatic, implicit manifestations of awareness of mental states (Carlson et al., 2013). These skills are thus thought to act as precursors of later-developing TOM skills that reflect an explicit, coherent, flexible and conceptual understanding of mental states (Carlson et al., 2013), and that constitute the topic of the current review. In sum, this review constrains TOM so as to distinguish it from empathy, classification of affective and situational cues, early non-explicit cognitive representations of mental states, such as joint attention and imitation, and more complex social abilities, such as cooperation or manipulation tactics.

### The Developmental Trajectory of TOM and Associated Measurement Tools

Taking into account the diverse definitions and conceptions of TOM, it is not surprising that a broad variety of paradigms and measures have been developed to study the construct. Despite the range of mental states a child must learn to interpret (e.g., emotions, knowledge, intents, beliefs, desires), there appears to be an over-representation of measures directed specifically at assessing one particular type of mental state: false beliefs (Hedger and Fabricius, 2011; Hiller et al., 2014). The false belief paradigm was initially proposed by Wimmer and Perner (1983) and has since been adapted and applied to a range of contexts (Wellman et al., 2001). Typically, children are presented with a short scenario depicting a contradiction between reality and a character's belief. For example, in the change of location paradigm referred to as the Sally and Ann task (Baron-Cohen et al., 1985), two dolls, Sally and Ann, are presented to a child. Sally places her marble in a basket, and then leaves the scene. Ann takes the marble out of the basket and puts it in a box. When Sally comes back, the child is asked where she would search for the marble. To succeed in this task, children have to answer “in the basket,” despite the fact that they know that the marble is really in the box. This type of scenario enables experimenters to determine a child's ability to understand that a person's mental state is not a simple reflection of reality, and suggests that the child is able to elaborate a theory about another person's mental content, a “theory of mind”.

Children typically complete false belief paradigms successfully somewhere between 3 and 5 years of age (Wellman et al., 2001), an observation which has long been linked to the assumption that this is the period during which TOM develops. However, the use of a broader variety of measures and methods has subsequently shown that TOM follows a more extended and nuanced developmental trajectory (Wellman et al., 2011). In particular, the emergence of implicit, non-verbal and simplified measures designed to be used in very young, pre-verbal infants, suggested that some TOM abilities may already be present in infancy, a conclusion that could not be reached using standard measures because of the extraneous factors inherent to the tests (Slaughter, 2015). For example, these studies used implicit methods, such as observation of imitation behaviors, violation-of-expectation paradigms and eye gaze tracking to show that children demonstrate some knowledge of the intentions of others around 12–18 months of age (Kristen et al., 2011), can appreciate others' desires around 18 months of age (Repacholi and Gopnik, 1997; Poulin-Dubois et al., 2007), and show some comprehension of false beliefs as early as 15 months of age (Onishi and Baillargeon, 2005; Southgate et al., 2007; Senju, 2012). The interpretation of these results has been the subject of much debate: whereas some claim that implicit tasks are valid methods to measure TOM (Carruthers, 2013; Powell et al., 2018), others suggest that they lack reliability and validity data to support their use (Dörrenberg et al., 2018; Kulke et al., 2018). This debate has been fueled by failed attempts to replicate studies using implicit measures of false-belief understanding, leading to a “replication crisis” (Sabbagh and Paulus, 2018). The issue of the reliability and validity of these tasks is intertwined with that of the nature of what is measured using implicit methods to test “theory of mind,” contributing to the debate regarding the conception and development of TOM and its first measurable manifestations (Heyes, 2014; Scott and Baillargeon, 2017; Sabbagh and Paulus, 2018). Conversely, the use of a variety of more complex explicit TOM tasks has suggested that TOM continues to develop after the age of 5 years. For example, children improve on their ability to understand second order false belief tasks (i.e., “Ann thinks that Sally thinks the marble is in the basket”) between 5 and 6 years of age, and develop an increasingly mature appreciation of sarcasm, *faux-pas* (social gaffes) and white lies throughout adolescence (Miller, 2009). Neuroimaging studies also depict longitudinal changes in patterns of cerebral activation during a variety of TOM tasks, and suggest protracted development well through adolescence and into adulthood (Blakemore, 2008, 2012). Together, these findings highlight that TOM cannot be seen as a unitary construct and must be appreciated in light of its ongoing development. They also support the importance of relying on diverse TOM measures that are reliable, valid and sensitive to developmental changes in order to adequately document a complex and rapidly changing cognitive ability.

### Psychometric Challenges Associated With TOM Measures

Despite significant advances in our understanding of both normative and altered TOM (Wellman et al., 2001; Gallagher and Frith, 2003; Vuadens, 2005; Poletti and Adenzalo, 2013; Kimhi, 2014; Imuta et al., 2016), it is still difficult to draw robust conclusions about its role in typical development and clinical conditions. Such challenges may be the result of the methodological weaknesses associated with measures used to assess TOM (Hiller et al., 2014; Henry et al., 2016). Indeed, the psychometric standards of TOM measures have been qualified as unsystematic, suboptimal, and immature (Mayes et al., 1996; Brune, 2001; Hutchins et al., 2008a; Carlson et al., 2013; Hiller et al., 2014). The methodological weaknesses of TOM assessment include reliance on measures with one or two tests items only (Cutting and Dunn, 1999; Garner et al., 2005), over-representation of false belief understanding as the sole measure of TOM (Wellman and Liu, 2004; Carlson et al., 2013; Hiller et al., 2014), and the fact that few TOM measures have empirically validated psychometric properties (Hutchins et al., 2008a; Hiller et al., 2014; Ziatabar Ahmadi et al., 2015).

### Existing Sources of Information on TOM Measures

To our knowledge, no systematic review has been conducted to document the characteristics of existing TOM measures for young children. Non-systematic reviews have been published on TOM measures that are widely used in clinical populations (Sprung, 2010), in adulthood (Henry et al., 2015), and in middle childhood and adolescence (Hayward and Homer, 2017). These reviews highlight the relevance of a number of TOM measures for understanding social functioning in clinical conditions and typical development and provide interesting insights in the ways to use them, but they are not systematic and do not cover tools destined for infants, toddlers and preschoolers. Ziatabar Ahmadi et al. (2015) conducted a systematic review of TOM measures for preschoolers, but constrained the scope to articles presenting the development and validation of comprehensive measures composed of multiple TOM tasks. Therefore, their review excludes single task measures (e.g., single false belief tasks) that constitute the majority of measures used in TOM research (Hiller et al., 2014). In addition, the review conducted by Ziatabar Ahmadi et al. (2015) is limited to studies that specifically aim to validate the psychometric properties of TOM measures, thus excluding other types of empirical studies (e.g., longitudinal, outcome or prediction papers).

The primary objective of this study was to systematically record an inventory of existing measures that assess TOM in children under the age of 6 years of age (0–5 years). This age range was chosen because the period between 3 and 5 years is widely recognized as a sensitive period for TOM development (Wellman et al., 2001). The range was extended down to infancy because there is no actual consensus regarding the age at which the first manifestations of TOM appear (Carlson et al., 2013). This inventory will assist researchers and clinicians in choosing measures that best fit their needs and will identify possible gaps or limits inherent to existing measures.

## Methods

A systematic review of the literature was conducted. Empirical studies referring to TOM measures used with young children were reviewed using a search protocol based on The Preferred Reporting Items for Systematic Reviews and Meta-Analyses statement (PRISMA; Moher et al., 2015). Eligibility criteria were pre-determined both at the level of study selection and identification of TOM measure (see Table 1 for the list of eligibility criteria and associated exclusion criteria).

**Table 1:** Eligibility and exclusion criteria for the systematic review.

**Eligibility Criteria**	**Exclusion criteria**
The document is accessible, in its full version, at the time of the search	**Missing document:** The document's reference is incomplete and does not allow identification of the full text or full text could not be found
	**Unpublished:** The document is not published or in press, or the in-press content is not accessible
The document is written in English or French. A list of possibly relevant titles in other languages is provided as Appendix I	**Other language:** The document is written in another language than English or French
The document is from a peer-reviewed journal or published by a test publisher/editor	**Non-reviewed or non-commercialized:** The document is not published in a peer reviewed journal (e.g., books, book chapters, conference proceedings, are excluded), nor is it commercialized
The document reports the results of an empirical study providing original data	**Not an empirical study:** The records do not report a study providing new and original data (e.g., theoretical articles, literature reviews, meta-analyses, letters, editorials, etc.)
The measure is used with human subjects	**Non-human subjects:** The measure was not administered to humans (e.g., animal studies)
The measure is administered to young children (<6 years of age). Studies with participants 6 years of age or over are included, provided the sample is also composed of children under 6 years. The sample may be composed of adults, as long as the measure aims to evaluate TOM in a child under 6 years of age (e.g., parental report in the form of a questionnaire)	**Older participants:** The measure is not administered to a child under 6 years of age
The measure provides a score or a classification. Subjective (i.e., questionnaires) or objective (i.e., direct testing, observational coding systems) are included	**Lack of score:** The measure does not provide a score or classification reflecting an individual's TOM (e.g., research paradigms used to solicit TOM during neuroimaging, but that do not score the participant's TOM abilities)
The measure can be used to assess TOM in normative or clinical conditions (physical, psychological or neurological)	**Narrow utility:** The measure is useful only in the case of a specific condition (e.g., blindness)
The measure aims to evaluate TOM as defined in the introduction, that is the ability to create a cognitive representation of self and other's mental states (SOME; Bird and Viding, 2014)	**No TOM or diverging TOM definition:** The measure does not assess TOM or does not assess it in a way that is consistent with the chosen theoretical framework (Bird and Viding, 2014). Measures that assess a more complex social behavior or ability (e.g., moral reasoning), a precursor social cognitive skill (e.g., joint attention), or another social cognitive construct (e.g., empathy, affective cues classification) are excluded

### Sources of Information and Search Strategy

The search strategy was created in collaboration with a psychology librarian. The following electronic databases were searched: Ovid PsycINFO, Health and Psychosocial Instruments, MEDLINE(R) In-Process and Other Non-Indexed Citations and MEDLINE(R). The dates of coverage were from 1983 to October 2019. The start date (1983) was chosen because of seminal work published in that year (Wimmer and Perner, 1983).

The following key search terms, pertaining to children (1), measures (2), and TOM (3) were used, in combination, and restrained to “all journals”:

1. (child^*^ or schoolchild^*^ or toddler^*^ or preschool^*^ or infan^*^).mp [mp = title, abstract, heading word, table of contents, key concepts, original title, tests, and measures]
2. (psychometric^*^ or validation or questionnaire^*^ or scale^*^ or inventor^*^ or instrument^*^ or measure^*^ or tool or assess^*^ or evaluation^*^).mp [mp = title, abstract, heading word, table of contents, key concepts, original title, tests, and measures]
3. (theory of mind or false belief^*^ or perspective taking^*^ or social attribution^*^ or belief attribution^*^ or desires reasoning).mp [mp = title, abstract, heading word, table of contents, key concepts, original title, tests, and measures]

In addition to the standard electronic search databases, the catalogs of the following English or French publishers of testing materials were manually reviewed: Pearson Assessment Canada, Psychological Assessment Ressources, Institut de Recherches Psychologiques, Western Psychological Services, Hogrefe, Les Éditions du Centre de Psychologie Appliquée, Eurotests Editions, PsychTest, Schuhfried. Whenever the age range of participants could not be extracted directly from an article, the corresponding author was contacted to obtain the information. Moreover, whenever the cited source of an assessment tool was not retrieved using the search strategy, it was manually searched and included as a record to be screened alongside others in the selection process, even though it was published before 1983.

### Selection Process

Search results were imported to an Endnote X7 database. Screening was performed in two phases. In phase 1, all search results were screened for the eligibility criteria based only on the content of the title and abstract, by two of the authors. Two decisions were possible at this stage: exclusion based on an eligibility criterion or inclusion for phase 2. In phase 2, the full texts of all remaining search results were screened for eligibility criteria by three of the authors. Two decisions were possible at this stage: exclusion based on an eligibility criterion or inclusion in the systematic review. For each phase, the first 15% of search results were screened independently by all reviewers in order to obtain an inter-rater agreement in terms of inclusion or exclusion of the search result. The inter-rater agreement was 89.9% at phase 1 and 93.9% at phase 2. During the entire process, any discrepancies or difficulties in the identification of inclusion/exclusion criteria were resolved by discussion with the other reviewers and authors if needed.

### Content Analysis and Data Extraction

A qualitative content analysis of the measures included was performed by all authors throughout the selection process in order to extract the discrete mental states and social situation understanding that were assessed by the included measures. Seven categories of mental states and social situations were identified across the collection of studies: emotions, desires, intentions, percepts, knowledge, beliefs, and mentalistic understanding of non-literal communication. An eighth category, called “comprehensive measures,” was added to represent measures encompassing the understanding of multiple mental states and social situations. These eight TOM categories were therefore used to classify the different measures during data collection.

Data collection was performed by the first three authors using a comprehensive pre-determined form. This form included the following variables related to the measures: category of mental state or social situation assessed, name of measure, author(s), and year of publication, reference(s) of articles that have used the measure, short description, administration format, number of items, scoring options, and administration time. It was also noted which articles provided original psychometric information. The data extraction form also included the following information regarding the participants assessed with the measures: age range of normative population, language(s) spoken, presence of adverse clinical (e.g., hearing impairments or deafness, Williams syndrome), psychological (e.g., anxiety or depression, externalizing behavior problems), or environmental (i.e., low socio-economic status, maltreatment) conditions assessed with the measures.

## Results

### Summary of Main Results and TOM Categories

**Figure 1:**
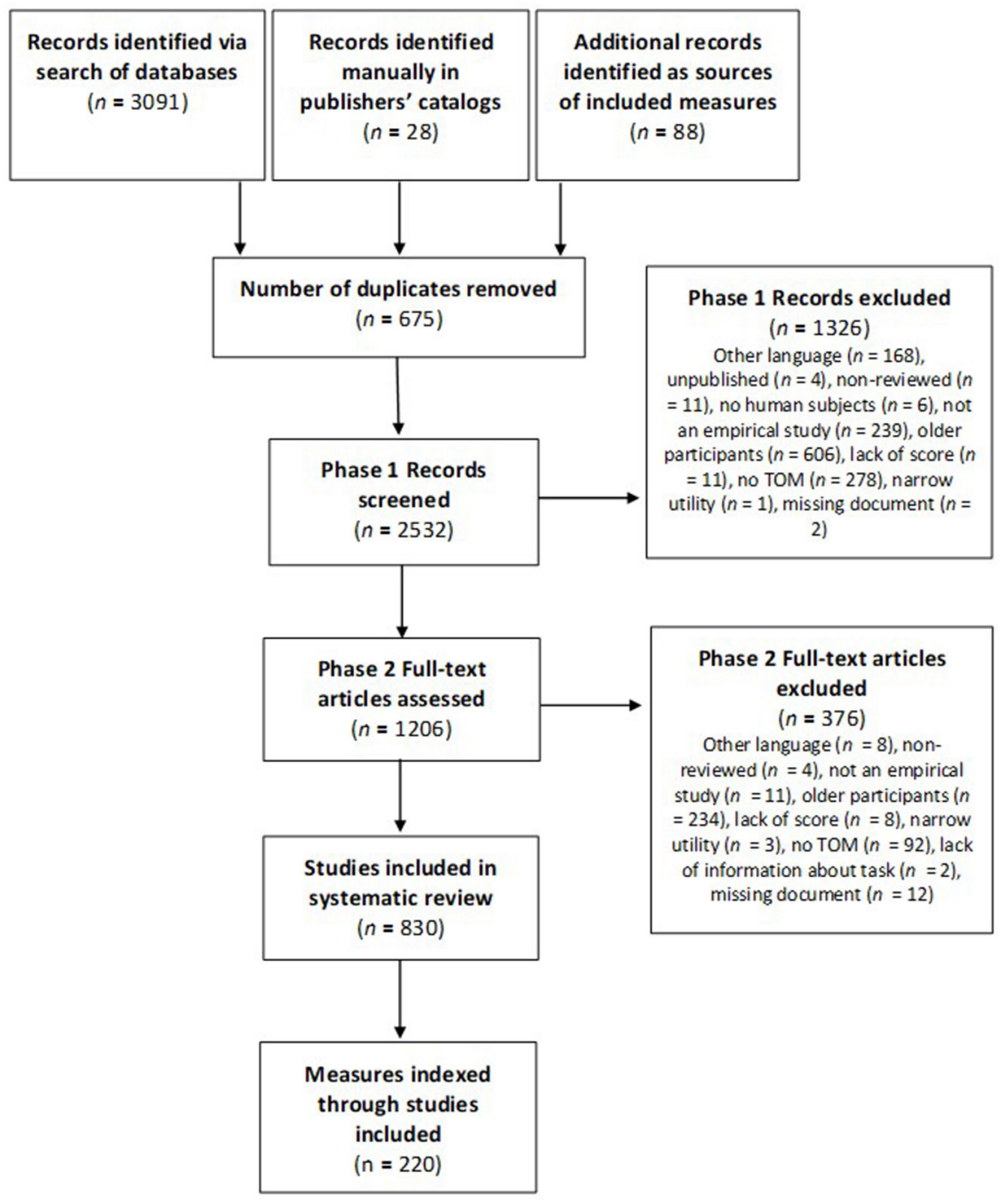
Flowchart of study identification and selection.

Figure 1 illustrates the steps in article selection. A total of 830 studies were included for data extraction. Given the large amount of studies and the numerous variations of the same measures found, a synthesis of the data was performed, which isolated 220 distinct measures and paradigms. Each is presented, along with their characteristics and details of participants that were tested across studies, in tables found in Appendix II. Appendix II contains eight separate tables according to the main TOM category they refer to: Emotions (Table a; 37 measures), Desires (Table b; 26 measures), Intentions (Table c; 16 measures), Percepts (Table d; 26 measures), Knowledge (Table e; 25 measures), Beliefs (Table f; 49 measures), Mentalistic understanding of non-literal communication (Table g; 16 measures) and Comprehensive measures (Table h; 25 measures). To further synthesize the results and provide clarity on the content of the tasks, the first seven categories were sub-divided into 39 TOM sub-abilities or sets of abilities assessed in the measures. Category 8, Comprehensive measures, was subdivided according to the format of the measures (i.e., questionnaires/interviews and direct tests). For example, the Desires category was divided into four sub-abilities: (1) understanding that different people may have discrepant desires, (2) understanding the co-existence of multiple desires at the same time or successively in one person, (3) understanding that people's emotions and actions are influenced by their desires/preferences, and (4) producing plausible explanations when action contradicts stated desires/preferences.

**Figure 2:**
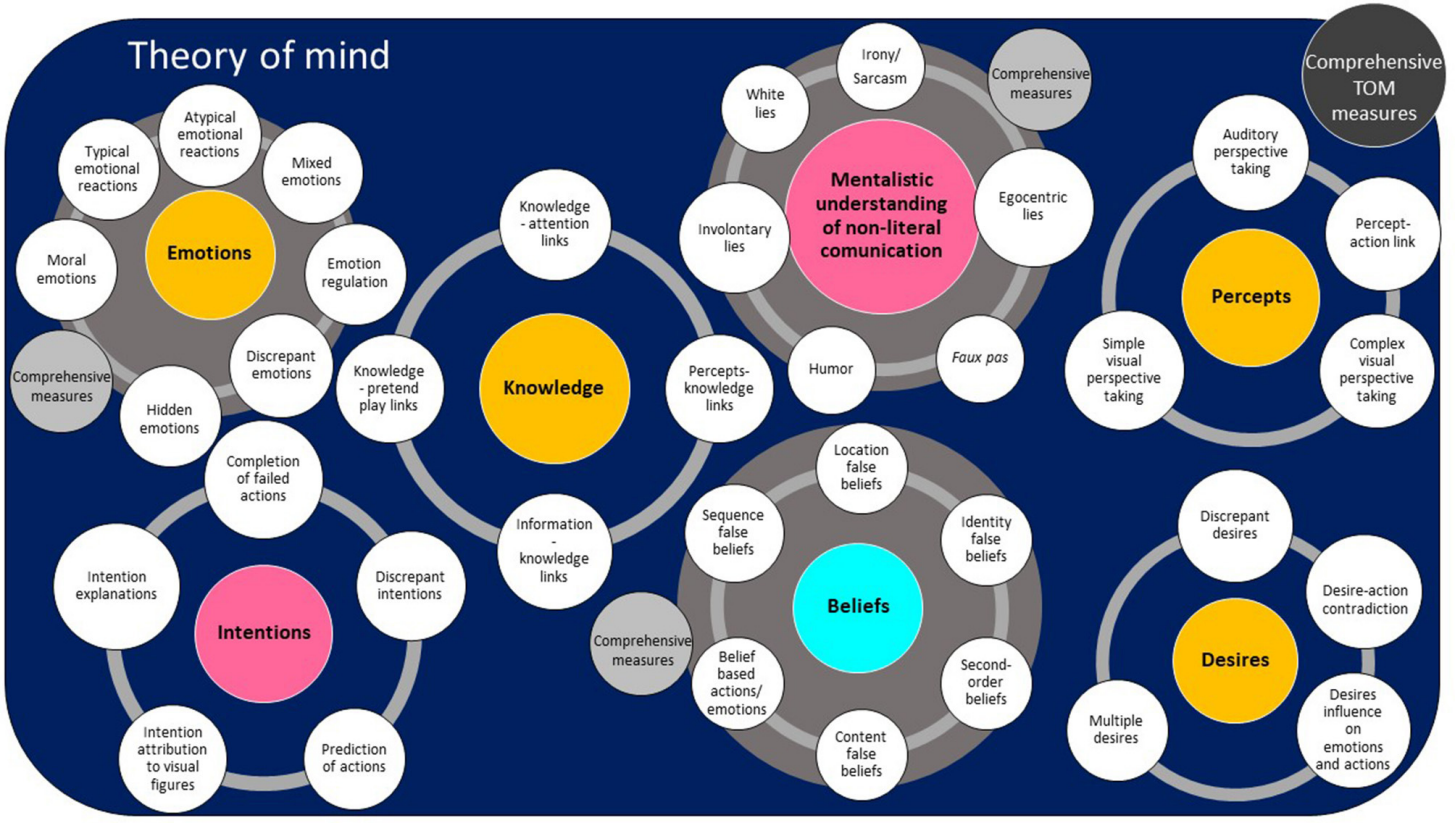
ATOMS framework. The ATOMS framework (Abilities in Theory of Mind Space) is a visual representation of the TOM categories and sub-abilities that emerge from the systematic review of TOM measures for young children. Theory of mind space is represented as a large area that includes seven TOM categories of mental states and social situations understanding (colored circles): Intentions, Desires, Emotions, Knowledge, Percepts, Beliefs, and mentalistic understanding of non-literal communication. Thirty-nine specific TOM sub-abilities (white circles) gravitate around the TOM category to which they pertain. When comprehensive measures exist that measure sets of abilities (multiple sub-abilities) for any one TOM categories, these are represented as gray circles. An eighth overall category “Comprehensive TOM measures” includes measures that encompass multiple TOM categories and is represented as a black circle. TOM categories (colored circles) are further represented using three different colors according to the proportion of reviewed studies that measured these types of TOM abilities: the pink circles represent TOM categories measured in <5% of studies, yellow circles represent TOM categories measured in 5–25% of studies, and the blue circle represent the only TOM category (Beliefs) measured in more than 25% of studies.

Table 2 provides an overview of the results and presents the first seven TOM categories and the 39 TOM sub-abilities, along with an example of a relevant measure and the number of measures and articles that were identified in relation to each sub-ability. Table 3 presents an overview of the measures included in the Comprehensive measures category. In order to visually represent the organization of the TOM abilities and sub-abilities that emerged from the systematic review, a framework depicting the various types of TOM measures and a related taxonomy was developed and is presented in Figure 2: Abilities in Theory of Mind Space (the ATOMS framework).

**Table 2:** TOM categories and sub-abilities and associated number of measures and articles.

**Mental states and social situations categories**	**TOM sub-abilities**	**Examples of measures/paradigms**	**Number of measures** ***n* (%)**	**Number of articles** ***n* (%)**
**Emotions**	1. Typical emotional reactions: Inferring a person's emotional reactions based on situations that typically elicit certain emotions/inferring a preceding event based on a person's emotional reaction	Affective knowledge understanding (Knafo et al., 2009)	19 (8.6%)	66 (8.0%)
	2. Atypical emotional reactions: Inferring or explaining a person's emotional reactions based on situations eliciting emotions that are atypical compared to what is usually expected	Affective perspective-taking (Denham, 1986)	6 (2.7%)	44 (5.3%)
	3. Discrepant emotions: Understanding that people may have discrepant feelings about an event	Affective perspective taking (Borke, 1971; Smith, 1973)	1 (0.5%)	4 (0.5%)
	4. Mixed emotions: Understanding that people may feel mixed emotions or different emotions successively	Mixed emotion understanding task (Gordis et al., 1989)	4 (1.8%)	16 (1.9%)
	5. Hidden emotions: Understanding that other people may hide their emotions	Appearance reality of emotions (Harris et al., 1986)	4 (1.8%)	107 (12.9%)
	6. Moral emotions: Understanding that negative feelings might arise following a reprehensible action	Morality-based emotions (Pons and Harris, 2000)	1 (0.5%)	8 (1.0%)
	7. Emotion regulation: Understanding that others might use strategies to regulate their emotions	Regulation of emotion (Pons and Harris, 2000)	1 (0.5%)	8 (1.0%)
	8. Comprehensive measure involving emotion understanding based on different factors/TOM categories (e.g., desires, beliefs, hiding emotions)	Test of emotion comprehension (Pons and Harris, 2000)	1 (0.5%)	16 (1.9%)
	**Emotions category totals**		**37 (16.8%)**	**198 (23.9%)**
**Desires**	1. Discrepant desires: Understanding that different people may have discrepant desires	Discrepant desires/Yummy-yucky task (Repacholi and Gopnik, 1997)	10 (4.5%)	130 (15.7%)
	2. Multiple desires: Understanding the co-existence of multiple desires simultaneously or successively in one person	Multiple desires task (Bennett and Galpert, 1993)	5 (2.3%)	5 (0.6%)
	3. Desires influence on emotions and actions: Understanding that people's emotions and actions are influenced by their desires/preferences	Desires task (Wellman and Bartsch, 1988; Wellman and Woolley, 1990)	10 (4.5%)	49 (5.9%)
	4. Desire-action contradiction: Producing plausible explanations when actions contradict stated desires/preferences	Anomalous-desires stories (Colonnesi et al., 2008)	1 (0.5%)	1 (0.1%)
	**Desires category totals**		**26 (11.8%)**	**178 (21.4%)**
**Intentions**	1. Completion of failed actions: Understanding another person's intent, as demonstrated by completing their failed action	Behavioral re-enactment procedure (Meltzoff, 1995)	1 (0.5%)	12 (1.4%)
	2. Discrepant intentions: Understanding that identical actions/results can be achieved with different intentions	Accidental transgression task (MoToM; Killen et al., 2011)	7 (3.2%)	12 (1.4%)
	3. Prediction of actions: Predicting people's actions based on their intentions	Attention to intention (Phillips et al., 2002)	5 (2.3%)	13 (1.5%)
	4. Intention attribution to visual figures: Tendency to attribute intentions to ambiguous visual figures	Valley task (Castelli, 2006)	1 (0.5%)	1 (0.1%)
	5. Intentions explanations: Producing plausible intention explanations for different types of observed social events	Intentions explanations (Smiley, 2001)	2 (0.9%)	2 (0.2%)
	**Intentions category totals**		**16 (7.3%)**	**36 (4.3%)**
**Percepts**	1. Simple visual perspective taking: Acknowledging that others have different visual percepts and adopting the visual perspective of another person	Visual perspective taking, Level 1/Picture identification task (Masangkay et al., 1974; Flavell et al., 1981)	15 (6.8%)	80 (9.6%)
	2. Complex visual perspective taking: Adopting another person's visual perspective in tasks demanding complex mental rotation or visualization	Visual perspective taking and spatial construction task (Ebersbach et al., 2011)	9 (4.1%)	14 (1.7%)
	3. Percept-action link: Understanding that other's actions are linked to their visual percepts	Perception based action (Hadwin et al., 1997)	1 (0.5%)	6 (0.7%)
	4. Auditory perspective taking: Considering the auditory percepts of another person	Auditory perspective taking (Williamson et al., 2015)	1 (0.5%)	1 (0.1%)
	**Percepts category totals**		**26 (11.8%)**	**97 (11.7%)**
**Knowledge**	1. Knowledge-pretend play links: Understanding that someone who does not know something exists cannot engage in “pretend play” that incorporates that knowledge	Sarah task (Aronson and Golomb, 1999)	3 (1.4%)	3 (0.4%)
	2. Percepts-knowledge links: Understanding that someone who does not have access to perceptual information (i.e., by looking, hearing, etc.) may not have access to knowledge	See-know task (Pillow, 1989; Ruffman and Olson, 1989)	11 (5.0%)	149 (18.0%)
	3. Information-knowledge links: Understanding that someone who was not informed or is not familiar with something may not know	Awareness of a reader's knowledge task (Peskin et al., 2014)	8 (3.6%)	10 (1.2%)
	4. Knowledge-attention links: Understanding that something new is more interesting to someone than something already known	Familiary-focus of attention (Moll et al., 2006)	2 (0.9%)	3 (0.4%)
	**Knowledge category totals**		**25 (11.4%)**	**163 (19.6%)**
**Beliefs**	1. Content false beliefs: Familiar container with an unexpected content: Understanding the false belief held by someone who never opened the container	Content false belief paradigm (Hogrefe et al., 1986; Perner et al., 1987)	4 (1.8%)	414 (49.9%)
	2. Location false beliefs: Unseen change: Understanding the false belief held by someone who did not witness or was not informed of a displacement or change of action	Change-in-location paradigm/Sally-Ann task (Wimmer and Perner, 1983; Baron-Cohen et al., 1985)	7 (3.2%)	396 (47.7%)
	3. Identity false beliefs: Understanding that when something looks/sounds/smells like something else, a person may hold a false belief about its identity	Appearance-reality test (Flavell et al., 1986)	16 (7.3%)	143 (17.2%)
	4. Second-order belief: Understanding the second-order belief or false belief held by someone who doe not know somebody else was informed (e.g., of a misleading identity, a misleading location, etc.)	Ice-cream van test (Perner and Wimmer, 1985)	7 (3.2%)	94 (11.3%)
	5. Beliefs based action/emotions: Predicting another emotions or actions based on their stated beliefs/Inferring another person's belief based on their stated action or emotion	The Tom task (Swettenham, 1996)	8 (3.6%)	154 (18.6%)
	6. Sequence false beliefs: Understanding the false belief created when a predictable sequence of stimuli is broken with the intrusion of an unexpected stimulus	Unexpected outcome (Brambring and Asbrock, 2010)	1 (0.5%)	1 (0.1%)
	7. Comprehensive measures of understanding beliefs	Battery of TOM tasks (Hughes et al., 2000)	6 (2.7%)	20 (2.4%)
	**Beliefs category totals**		**49 (22.3%)**	**627 (75.5%)**
**Mentalistic understanding of non-literal communication**	1. Irony/sarcasm: Understanding that other people may lie in order to be ironic/sarcastic	Lies and jokes task (Sullivan et al., 1995)	6 (2.7%)	19 (2.3%)
	2. Egocentric lies: Understanding that someone may consciously lie in order to avoid a problem or to get its way	Lie stories from the Strange stories (Happé, 1994)	4 (1.8%)	13 (1.6%)
	3. White lies: Understanding that someone may lie in order to spare another's feelings	White lie stories from the Strange stories (Happé, 1994)	1 (0.5%)	14 (1.7%)
	4. Involuntary lies: Understanding that someone may tell a “lie” without knowing	Forget stories from the Strange stories (Happé, 1994)	1 (0.5%)	11 (1.3%)
	5. Humor: understanding that someone may tell a “lie” in order to make a joke	Joke stories from the Strange stories (Happé, 1994)	1 (0.5%)	11 (1.3%)
	6. *Faux pas*: Ability to recognize *faux-pas* (social gaffe) situations	Recognition of faux pas (Baron-Cohen et al., 1999)	1 (0.5%)	6 (0.7%)
	7. Measures tapping multiple aspects of mentalistic understanding of non-literal communication	Strange stories (Happé, 1994)	2 (0.9%)	22 (2.7%)
	**Mentalistic understanding of non-literal communication totals**		**16 (7.3%)**	**30 (3.6%)**

*Percentages are calculated using the total number of measures (220) and studies (830) included in the review. Some TOM category total of articles are lower than the sum of articles assessing its sub-abilities because each article may assess more than one sub-ability*.

**Table 3:** Comprehensive measures and associated number of measures and articles.

**Formats**	**Examples of measures**	**Number of measures** ***n* (%)**	**Number of articles** ***n* (%)**
1. Multiple TOM abilities measured using questionnaires/interviews	Theory of mind inventory (ToMI) (Hutchins et al., 2012)	4 (1.8%)	19 (2.3%)
2. Multiple TOM abilities measured using direct testing	ToM scale (Wellman and Liu, 2004)	21 (9.5%)	183 (22.0%)
**Total comprehensive measures**		**25 (11.4%)**	**194 (23.4%)**

*Percentages are calculated using the total number of measures (220) and studies (830) included in the review*.

### Information for Navigating the Results Tables

In the tables (Appendix II, Tables a–h), within one TOM sub-ability, measures are presented in alphabetical order according to the first author of the original measure. Articles reporting the use of these measures follow the name of the measure in a numbered format referring to the alphabetical order of authors in the reference list. In addition, within one TOM sub-ability, participants' characteristics are also presented in alphabetical order, when relevant (i.e., languages and adverse conditions). It should be noted that a single article may be cited more than once since it may report the use of more than one TOM measure. Furthermore, measures entailing more than one subtask (i.e., measures from the comprehensive measures category and measures taping multiple sub-abilities within a specific category) were divided in subtasks and added to the single measures reported, whenever sufficient information was available to do so. Consequently, a single article may be cited as using a comprehensive measure (e.g., Theory of mind scale; Wellman and Liu, 2004) and its subtask (e.g., Content false belief paradigm; Hogrefe et al., 1986; Perner et al., 1987). This procedure for reporting task-related information was applied both to existing tasks embedded in a comprehensive measure (as in the preceding example), as well as, new subtasks created specifically for a comprehensive measure (e.g., Forget stories from the Strange stories; Happé, 1994). In Tables a–h, the column “Availability of psychometric information” informs on the presence (+) or absence (–) of psychometric properties related to a specific measure. When present, the information is then presented in detail in two distinct tables (Appendix III, Tables i, j).

When consulting the results tables, readers should be aware of some caveats associated with the data synthesis process. In particular, it is important to note that a specific measure or paradigm may tap more than one TOM category or sub-ability, but for practical reasons, it was placed under the one that was judged to best reflect its measurement scope. For example, the Ella the elephant task (Harris et al., 1989), which captures the emotions associated with false beliefs (e.g., happiness when seeing a can of a preferred beverage, without knowing the content has been replaced by a disliked beverage), was placed in the Beliefs category even though understanding of emotions and desires are also secondarily involved in the task. Related to this and given the existence of multiple variations of the same paradigms, measures were placed under a common banner when they had strong similarities, even if the authors did not refer directly to the original source. For example, the Ernie test and Linda test, presented by Ford et al. (2012), were referenced under the measure Change-in-location paradigm/Sally and Ann task because they rely on false beliefs associated with the unseen displacement of an object, a paradigm typically attributed to Wimmer and Perner (1983) by most authors. It is also important to note that the original source of a measure may not have been included in the review because of an exclusion criterion (e.g., the original reference for the Emotion Understanding Assessment is in a book; Howlin et al., 1999). In these cases, the source article was not included in the review, but the reference is provided in the tables, beside the name of the measure.

### Measure Characteristics

#### Modes of Presentation

Many different presentation modalities are used across TOM measures, but most rely on direct testing with the child, using read-aloud stories enacted with figurines (19 sub-abilities, e.g., Allen and Kinsey, 2013), or scenarios depicted with pictures (32 sub-abilities, e.g., Galende et al., 2011). Some measures rely on videos (8 sub-abilities, e.g., Mayes et al., 1996), audio-recordings or read-aloud scenarios (21 sub-abilities, e.g., Whitehouse and Hird, 2004), videogames, games or other realistic laboratory situations with the experimenter and/or other persons (14 sub-abilities, e.g., Brown, 2006). Many measures have variations in possible presentation modalities across studies. A good example of this is that all of the references cited in the first part of this section refer to assorted presentation modes of a single measure, the Change-in-location/Sally and Ann task. Most TOM measures use visual support, with few relying solely on verbal information (e.g., Faux pas task used by Hoogenhout and Malcolm-Smith, 2014), and few being entirely non-verbal (e.g., Behavioral re-enactment procedure used by Meltzoff, 1995). Only four measures using a questionnaire format were identified: Everyday mindreading skills and difficulties scale (Peterson et al., 2009), Theory of mind inventory (Hutchins et al., 2008a, 2012), Supplementary social and maladaptive items/É*chelle d'adaptation sociale pour enfants* (Frith et al., 1994) and Children's social understanding scale (Tahiroglu et al., 2014). These are completed by parents and/or a third-party adult, such as a daycare provider or educator.

#### Number of Items

The number of items in each measure varies from 1 to 54 in single category measures (Tables a–g) and from 1 to 110 in comprehensive measures (Table h). The number of items administered is highly variable from one study to another. For example, Wellman and Liu's Theory of mind scale (2004) is variably reported as being administered in 3, 4, 5, 6, and 7-item formats, each using a different sampling of items from the original scale (e.g., Davis et al., 2011; Suway et al., 2012; Strasser and del Rio, 2014; Dore and Lillard, 2015). Some authors also indicate that they used only a single task from the Theory of mind scale (e.g., O'Reilly et al., 2014).

#### Scoring Options

Many measures use a simple correct/incorrect scoring scheme (37 sub-abilities) for the child's verbal (e.g., saying where a character will search for an object; Wang et al., 2014) or behavioral (e.g., giving the experimenter a book he showed a preference for; Laranjo et al., 2010) response to test items. Some measures use a more elaborate scale or coding system (30 sub-abilities) to evaluate children's behavior (e.g., extent to which children adapt their behavior in order for their parent to see an object; Laranjo et al., 2010) or verbal explanation to open-ended questions (e.g., quality of justification when inferring an emotion; Nader-Grosbois et al., 2013). Timing and direction of eye gaze is also used as an indicator of TOM (9 sub-abilities), and assessed using observation coding systems (Poulin-Dubois and Yott, 2014) or eyetracking (Gliga et al., 2014). Of note, from one study to another, there are many adaptations of scoring schemes for the same measure. For example, in two studies using a Change-in-location paradigm/Sally-Ann task to assess false belief understanding, Adrian et al. (2005) asked questions and coded children's verbal answers in a correct/incorrect format, while Senju et al. (2011) coded children's eye movements using an eyetracker.

#### Administration Time

While initially extracted from the articles included in the review, administration time was not reported in the final tables of results since only a small proportion (5.1%) of authors reported this information. Moreover, it is highly probable that administration time varies substantially from one measure adaptation to another.

#### Psychometric Properties

Basic information on internal structure and consistency, inter-rater reliability and test-retest reliability are listed in Tables i and j when available (Appendix III), along with the 168 references providing this information (20.2% of included articles). The articles were further qualified as to whether they used an implicit (i.e., non-verbal, indirect and implied cues of children's TOM understanding, such as eye gaze tracking or behavioral observation) or explicit (i.e., direct response provided by the participant, such as verbal responses or pointing to a specific response choice) method for data collection. Fourty-one articles (4.9%) provided psychometric properties on implicit methods to measure TOM, using 20 different measures/paradigms. Measures are ordered according to the category of mental state and social situation understanding they pertain to and presented in alphabetical order using the name of the first author of the tool. Articles providing psychometric information are also listed in alphabetical order using first author's name. For many studies, the psychometric data were analyzed using individuals pooled from many age groups and/or adverse conditions. For this reason, the reader is invited to directly consult the studies in order to carefully interpret the data provided. Some studies (e.g., Yagmurlu et al., 2005; Guajardo et al., 2013) report the psychometric properties of aggregates of TOM measures, but these were not included in the tables since they do not refer to one specific measure reviewed. Table 4 provides an overview of the number of studies providing evidence for or against psychometric validation of four broad categories of indices: internal structure and consistency, inter-rater reliability, test/retest reliability and other psychometric information.

**Table 4:** Reliability and validity evidence of included TOM measures (number of studies supporting evidence/number of studies less supportive of evidence).

**Measures (source author)**	**Internal consistency**	**Interrater reliability**	**Test/retest reliability**	**Other psychometric information (e.g., scaling analysis, validity testing)**
**CATEGORY: EMOTIONS UNDERSTANDING**				
Affective perspective taking (Cassidy et al., 1992)	2/1	4	0	0
Affective perspective-taking tests (Denham, 1986)	4	1	0	0
Knowledge of emotion cause (Denham et al., 1994)	0	1	0	0
Description of emotional situation (Feshbach and Cohen, 1988)	0	1	0	0
Emotion situation knowledge task (Garner et al., 1994)	1/1	0	0	1
Mixed emotion understanding task (Gordis et al., 1989)	2	1	0	0
Appearance reality of emotions (Harris et al., 1986); Affective false-belief task (Davis, 1998)	0	5	0	0
Emotion understanding assessment (Howlin et al., 1999)	2/1	0	1/0	1
Affective attribution and reasoning task (Iannotti, 1978)	0/1	1	0	0
Test of emotion comprehension (Pons and Harris, 2000)	2/2	0	0	1
Emotion recognition questionnaire (Ribordy et al., 1988)	2/2	0	0	0
**CATEGORY: DESIRES UNDERSTANDING**				
Diverse desire (Bartsch and Wellman, 1989)	0	1	0	0
Gift task (Flavell, 1968)/Gift selection task (Jin et al., 2017)	0	1	0	0
Discrepant desires Yummy-yucky task (Repacholi and Gopnik, 1997)	0	2	0	0/1
Common and uncommon desires (Rieffe et al., 2001)	1	2	0	0
Desire and intention task (Schult, 2002)	0	2	0	0
Target-hitting game (Schult, 2002)	0	1	0	0
Not own desire tasks (Wellman and Woolley, 1990)	0	4	0	0
Desire task (actions and emotions stories) (Wellman and Woolley, 1990)	1	0	0	0
**CATEGORY: INTENTION UNDERSTANDING**				
Behavior-, skill-, and awareness-intentionality measures (Astington and Lee, 1991)	0	1	0	0
Visual habituation paradigm (Buresh and Woodward, 2007)	0	2	0	0
Intention and beliefs (Choi and Luo, 2015)	0	1	0	0
Behavioral re-enactment procedure (Meltzoff, 1995)	0	6	0	1/1
Accidental transgression task (MoToM; Killen et al., 2011)	0	1	0	0
Intention task (Phillips and Wellman, 2005)	0	1	0	0/1
Attention to intention (Phillips et al., 2002)	0	1	0	0
**CATEGORY: PERCEPTS**				
Visual perspective taking and spatial construction task (Ebersbach et al., 2011)	0	1	0	0
Photographers perspective taking (Frick et al., 2014)	1	0	0	0
Penny game task (Gratch, 1964)	2	1	0	1
Perception based action (Hadwin et al., 1997)	0	0	0/1	0
Gaze-following task (Meltzoff and Brooks, 2008)	0	1	0	1
Occluded object task (Moll and Tomasello, 2006)	0	2	0	0
Level-1 perspective taking tasks (Ricard et al., 1999)	0	1	0	0
**CATEGORY: KNOWLEDGE**				
Cognitive perspective taking (Brice and Torney-Purta, 1981)	0/1	0	0	0
Cognitive perspective taking (Flavell, 1968)	0	1	0	0
Familiary-focus of attention (Moll et al., 2006)	0	1	0	0
Knowledge theory of mind task (Moll and Tomasello, 2007)	0	2	0	0
See-know task (Pillow, 1989; Ruffman and Olson, 1989)	0	5	1	0
Hide an object (Viranyi et al., 2006)	0	1	0	0
**CATEGORY: BELIEFS UNDERSTANDING**				
Deceptive contents false-belief task (Bartsch and Wellman, 1989)	1/1	2	0	1
Picture false-belief task (Callaghan et al., 2012)	0	1	0	0
Lexical ambiguity (Carpendale and Chandler, 1996)	0	1	0	0
Droodle task (Chandler and Helm, 1984; Hughes et al., 1997)	0	2	0	0/1
Appearance-reality tasks (Flavell et al., 1986)	0	1	0	0
Ella the elephant or Emotion false belief task (Harris et al., 1989)	0	0	0/1	0
Content false belief paradigm (Hogrefe et al., 1986; Perner et al., 1987)	2	13	0/1	1
Battery of TOM tasks (Hughes et al., 2000)	0/2	1	0/1	0
ToM task (Kim and Phillips, 2014)	1	0	0	0
Message-desire discrepancy (Mitchell et al., 1997)	0	0	1	0
False-belief suspense (Moll et al., 2016)	0	1	0	0
Ice-cream van test (Perner and Wimmer, 1985)	0	1	0/1	0
False belief story (Riggio and Cassidy, 2009)	0	1	0	0
Birthday puppy (Sullivan et al., 1995)	0	1	0	0
Granddad story, Window story or Tom's crayon (Sullivan et al., 1995; Astington et al., 2002)	1	2	0/2	0
Ambiguity task (Taylor et al., 1988)	0	2	0	0
False-belief explanation task (de Villiers and de Villiers, 2000)	0/1	0	0	0
Belief tasks (Wellman and Bartsch, 1988)	0	7	0	0
Change-in-location paradigm (Wimmer and Perner, 1983)/Sally-Ann task (Baron-Cohen et al., 1985)	1/2	22	1/2	2/5
**CATEGORY: MENTALISTIC UNDERSTANDING OF NON-LITERAL COMMUNICATION**				
Irony task (Filippova and Astington, 2008)	0	1	0	0
Joke stories from the Strange stories (Happé, 1994)	0	1	0	0
Sarcasm stories from the Strange stories (Happé, 1994)	0	2	0	0
Strange stories (Happé, 1994)	2/1	6	0	0
White lies stories from the Strange stories (Happé, 1994)	0	1	0	0
Recognition of faux pas (Baron-Cohen et al., 1999)	1/1	1	0	1
**CATEGORY: COMPREHENSIVE MEASURES (DIRECT TESTS)**				
ToM storybooks (Blijd-Hoogewys et al., 2008)	1/1	1	1	2
Psychological explanation task (Colonnesi et al., 2008)	0	1	0	0
Comic strip task (Cornish et al., 2010)	0/1	0	0	1
Perspective taking task (Edelstein et al., 1984)	0	1	0	1
TOM task battery (Hutchins et al., 2008b)	2	2	2	1
Theory of mind subtest from a developmental neuropsychological assessment (NEPSY-II; Korkman et al., 2007)	1	1	1	1
Perspective-taking (Krcmar and Vieira, 2005)	1	1	0	0
Pragma test (Loukusa et al., 2014)	0	1	0	1
Social meaning scale from the SELweb (McKown et al., 2016)	1	0	0/1	1
TOM test (Muris et al., 1999)	3/1	2	1	1
Perspective-taking tasks (Oppenheimer and Rempt, 1986)	1	0	0	0
Theory of mind test (Pons and Harris, 2000)	0/1	0	0	0
Explanation of action task (Tager-Flusberg and Sullivan, 1994)	0	1	0	0
ToM scale (Wellman and Liu, 2004)	9/5	9	0	15
**CATEGORY: COMPREHENSIVE MEASURES (QUESTIONNAIRES)**				
Supplementary social and maladaptive items/É*chelle d'adaptation sociale pour enfants* (Frith et al., 1994)	1	1	0	2
Theory of mind inventory and Perceptions of children's theory of mind measure-experimental version (Hutchins et al., 2008a, 2012)	4	0	3	5
Everyday mindreading skills and difficulties scale (Peterson et al., 2009)	1	0	0	1
Children's social understanding scale (Tahiroglu et al., 2014)	3	0	1/1	2

*Evidence was generally determined as a value over 0.70 for internal consistency, inter-rater reliability, test/retest and intra-rater (other psychometric information) coefficients, or as % agreement over 80%, and as performed scaling analyses or explicitly tested convergence validity or replicability leading to positive results (other psychometric information). Included measures that are not part of this table had no studies reporting on their psychometric properties. Please see tables i and j (Appendix III) for more detailed psychometric information*.

##### Internal structure and consistency

Internal consistency refers to the extent to which different items of an assessment tool are inter-correlated, and so refer to the same construct (Terwee et al., 2007). It is recommended to first analyse the structure of the measure, using factor analysis or principal component analysis, to determine/confirm the number of scales before measuring the internal consistency of each scale (Terwee et al., 2007). Of note, hereafter, scaling analyses were not included as formal structure analyses and are instead included in “other psychometric information.” Information on internal consistency was found for 37 TOM measures (16.8%) within 72 studies (8.7%). However, only 10 measures also had formal structure analyses (4.5%): three emotions category measures, one Mentalistic understanding of non-literal communication measure and six comprehensive measures. Cronbach alpha is recognized as a good measure of internal consistency and is considered to be adequate when between 0.70 and 0.95 (Terwee et al., 2007). Only four measures had information on their internal structure and their Cronbach's alphas were always between 0.70 and 0.95 across all the studies that provided both structure and consistency information: Children's social understanding scale (Tahiroglu et al., 2014), Theory of Mind Inventory and Perceptions of Children's Theory of mind inventory and Perceptions of children's theory of mind measure-experimental version (Hutchins et al., 2008b, 2012), TOM task battery (Hutchins et al., 2008b) and “Social meaning scale (SELweb)” (McKown et al., 2016). All the measures were from the comprehensive measures category and all used explicit methods to test TOM.

##### Reliability

Inter-rater reliability and test-retest reliability were reported using similar parameters. Weighted Cohen's Kappa coefficient is the most recommended method for reporting the reliability of ordinal measures, whereas an intraclass correlation coefficient is recommended for continuous measures (Terwee et al., 2007). Other inter-rater reliability parameters reported include percentage of agreement and Pearson correlations, which are judged as less adequate measures of reliability (Terwee et al., 2007). *Inter-rater reliability*: Inter-rater reliability was reported for 62 measures (28.2%) within 95 studies (11.4%). Weighted Cohen's Kappa is available for 47 of these measures (21.4%), distributed through all TOM categories. Whenever reported, the Cohen's Kappa coefficients always met the 0.70 minimum standard for reliability, including implicit methods (16 Cohen's Kappa coefficients, reflecting on inter-rater reliability for nine implicit methods/paradigms) (Terwee et al., 2007). *Test-retest reliability:* Test-retest reliability was provided for 18 measures (8.2%) within 15 studies (1.8%), none of which pertained to implicit methods/paradigms. Cohen's Kappa coefficient or intraclass correlation coefficients are available for nine explicit measures (five in the Beliefs category, two in the Comprehensive measures category, one in Percepts category and one in Knowledge category; 4.1%). The 0.70 minimal standard value was attained in all studies reporting this information for three measures: See-know task (Pillow, 1989; Ruffman and Olson, 1989), Message-desire discrepancy (Mitchell et al., 1997) and TOM test (Muris et al., 1999).

##### Other psychometric information

Some studies (27 measures, 12.3%; 48 studies, 5.8%) also included other statistics related to a particular measure's psychometric properties. This information is detailed in Tables i, j under “Other psychometric information” and includes, for example, scalability (e.g., Guttman analyses) or construct validity testing, including analyses performed in order to test specific hypotheses regarding the construct validity of the measure (e.g., concurrent and discriminant validity). These additional types of psychometric properties were mostly tested in comprehensive measures (36 out of 48 studies providing specific validity information). In particular, each of the four questionnaires was reported to correlate with TOM scores from direct testing (Hughes et al., 1997; Comte-Gervais et al., 2008; Hutchins et al., 2008a, 2012; Peterson et al., 2009; Houssa et al., 2014; Tahiroglu et al., 2014; Smogorzewska et al., 2019). Among the information retrieved for validity testing, only 10 measures explicitly tested and demonstrated the links between test scores and a measure of social ability: these were all from the comprehensive measures except three tests: Theory of mind inventory (Hutchins et al., 2012), TOM storybooks (Blijd-Hoogewys et al., 2008), TOM test (Muris et al., 1999), TOM task battery (Hutchins et al., 2008b), Theory of mind scale (Wellman and Liu, 2004), Social meaning scale from the SEL web (McKown et al., 2016), Children's social understanding scale (Tahiroglu et al., 2014), Emotion situation knowledge task (Garner et al., 1994), Emotion understanding assessment (Howlin et al., 1999) and Recognition of faux pas (Baron-Cohen et al., 1999). Other important information presented in this section pertains to results from replicability testing: six studies reported independent results replication attempts using five TOM measures, including different variations in their modes of presentation and scoring methods. Most of those studies targeted implicit measures and were not or only partially able to replicate the past results. It is important to note that only articles providing clear objectives to test the validity or reliability of a measure were listed in the tables. However, multiple other articles may provide implicit cues regarding the validity of a measure, such as correlations with other relevant constructs.

### Participant Characteristics

#### Languages

While the majority of study samples were comprised exclusively of English-speaking participants (597 studies, 71.9%), some measures were also administered to children speaking 39 other languages (233 studies, 28.1%).

#### Age of Typically Developing Children Assessed

While this review specifically aimed to retrieve measures used with young children, typically developing children and adolescents across the pediatric range have also been tested using the measures identified. The youngest typically developing participants reported were 6 months old (Sodian et al., 2016) and some studies included both children and adults (e.g., Reed, 1994; Hirai et al., 2013). Infants have been tested using Intentions (age range: 6 months−17 years old), Percepts (age range: 11 months−40 years old), Desires (age range: 12 months−29 years old), Beliefs (age range: 12 months−92 years old) and Knowledge (age range: 17 months−16 years old) categories of TOM, whereas other categories are limited to older participants (Emotions: 23 months−15 years old; Mentalistic understanding of non-literal communication: 36 months−16 years old).

#### Adverse Conditions

**Figure 3:**
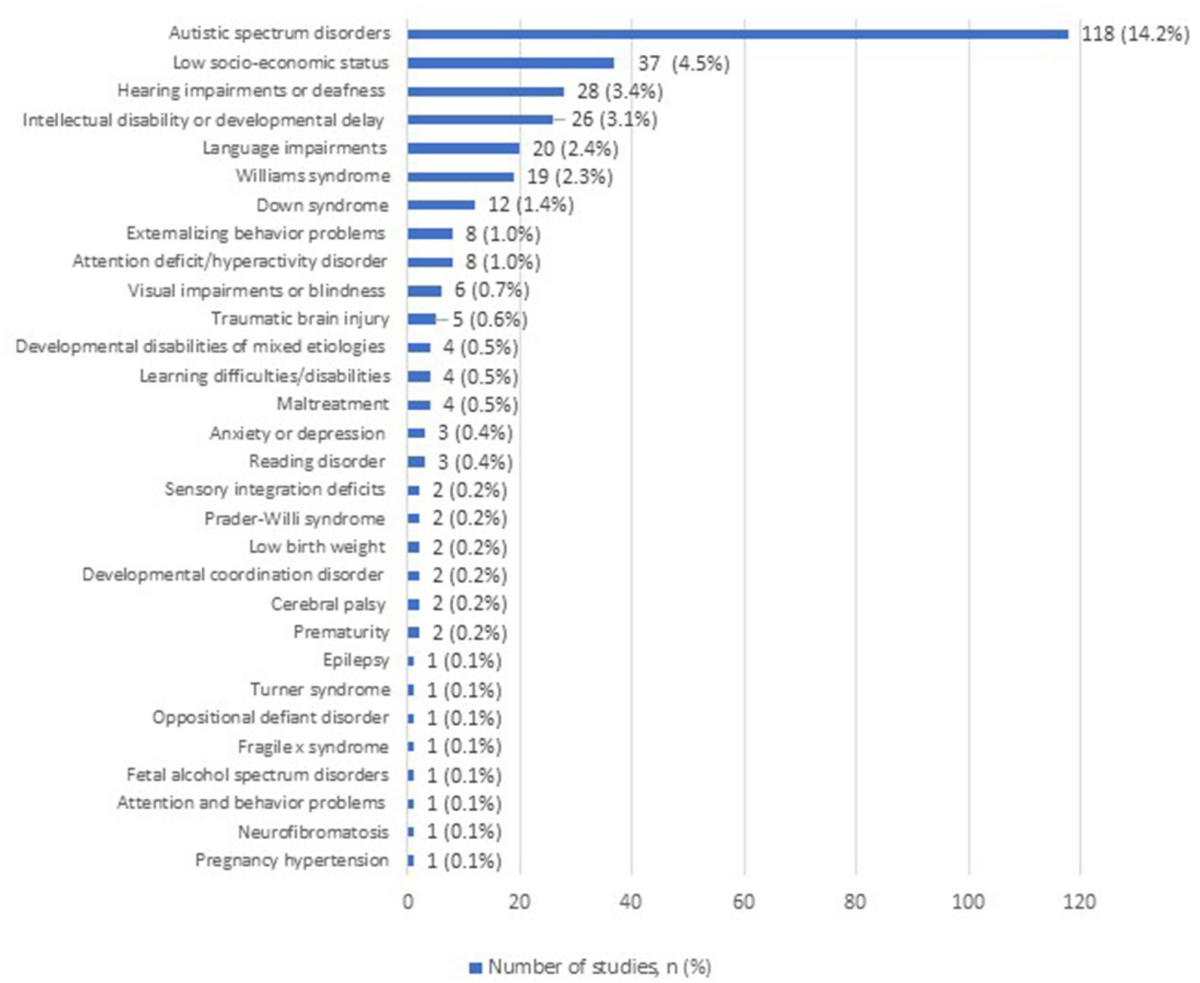
Number of studies including samples of children exposed to adverse medical, psychological, or environmental conditions.

In addition to using the measures with typically developing participants, many studies report on their use in children, adolescents or adults with medical (e.g., deafness), psychological (e.g., anxiety or mood disorders), or environmental (i.e., low SES and maltreatment) adverse conditions (236 studies, 28.4%). Thirty different conditions were documented throughout the measures reviewed (Figure 3). The most frequently studied conditions were autism spectrum disorders (118 studies, 14.2%), low socio-economic status (37 studies, 4.5%), hearing impairments and deafness (28 studies, 3.4%), intellectual disability and developmental delay (26 studies, 3.1%), and language impairments (20 studies, 2.4%).

## Discussion

Peer-reviewed literature and relevant test publishers' catalogs were systematically screened in order to generate an inventory of existing TOM measures that have been used with children under 6 years of age. A total of 220 measures, identified through 830 studies, were found to assess the understanding of seven different categories of mental states and social situations: Emotions, Desires, Intentions, Percepts, Knowledge, Beliefs, and Mentalistic understanding of non-literal communication. These were further divided into 39 distinct TOM sub-abilities that have been studied in infants, toddlers and preschoolers. In addition, an eighth category, Comprehensive measures, is comprised of tools assessing multiple categories. To our knowledge, this is the first comprehensive systematic review conducted to document of TOM measures for individuals of any age. This research extends the findings of previous non-systematic literature reviews in other populations (Sprung, 2010; Henry et al., 2015; Hayward and Homer, 2017) and of a systematic review targeting specifically comprehensive and validated TOM measures in preschool children (Ziatabar Ahmadi et al., 2015), and provides a more complete picture of existing TOM assessment methods that can be used with children under the age of six. Information gleaned from the measures and from the review provides an opportunity to identify some of the challenges and future directions associated with TOM assessment.

### Contributions, Challenges, and Possibilities in Relation to TOM Assessment

#### Diversity of TOM Abilities

In the last 36 years, studies have focused primarily on TOM abilities related to understanding of Beliefs (75.5% of studies), with fewer studies focussing on other aspects of TOM, such as the understanding of Emotions (23.9%), Desires (21.4%), Intentions (4.3% of studies), and Knowledge (19.6% of studies). However, it appears that an increasing number of studies use Comprehensive measures (23.4%) that tap more than one category of mental states and social situation understanding. These findings align with efforts to diversify sampling of TOM skills when assessing social cognition, in order to better capture its complex nature (Carlson et al., 2013; Hiller et al., 2014; Ziatabar Ahmadi et al., 2015). To this effect, Hiller et al. (2014) underscore the idea that isolated tests do not capture the rich manifestations of TOM abilities, limit the contributions of informative longitudinal assessment, and are an obstacle to understanding TOM development (Hiller et al., 2014). Social cues are among the most complex stimuli that the human brain has to process and are subject to both experiential and environmental influences; measures of social cognition should therefore reflect the complex nature of social stimuli and situations (Beauchamp, 2017). The measurement of more diverse TOM abilities, rather than a narrow focus on false belief understanding, could help enhance external validity, which was rarely tested in the studies included in this review, and has not typically been supported in other research (Happé et al., 2017).

#### Applications and Contributions of the ATOMS Framework

This review led to the elaboration of a new TOM taxonomy, the ATOMS framework (7 categories, 39 sub-abilities). While the primary goal of this classification was to facilitate synthesis and to structure the presentation of a substantial amount of data, the framework also provides an opportunity to reflect on theoretical, methodological and clinical challenges pertaining to TOM. At a theoretical level, the ATOMS classification highlights the need to better conceptualize TOM as a construct. To date, theoretical models mostly aim to explain the links between TOM and other socio-cognitive constructs, such as empathy, emotion recognition and pretend play (Leslie, 1987; Tager-Flusberg and Sullivan, 2000; Abu-Akel and Shamay-Tsoory, 2011; Bird and Viding, 2014; Happé and Frith, 2014; Westby, 2014; Asakura and Inui, 2016; Happé et al., 2017), but give few details on the make-up of TOM itself. The lack of theoretical structure and shared taxonomy in TOM definitions and its underlying composition impedes our ability to fully integrate TOM in a coherent and comprehensive framework linking it to various socio-cognitive abilities, a pervasive issue observed across the domain of social cognition (Beauchamp, 2017; Happé et al., 2017). The ATOMS framework provides structure for detailing TOM sub-components and for associating them with a nomenclature that could be applied to other work.

This classification may also contribute to guiding the development and interpretation of more comprehensive research protocols and clinical evaluations. The inventory may help enrich TOM evaluation by increasing and diversifying the TOM abilities that are targeted. It could also promote the creation of more comprehensive assessment tools, inspired by the multiple skills composing TOM and the variety of existing measurement methods highlighted in this review. In research and clinical settings, measures could be more precisely chosen and interpreted to target specific TOM abilities (Happé et al., 2017).

#### Diversity of Measurement Methods

This review highlights the creativity drawn on by those who develop new TOM measures, as reflected in the large variety of modes of presentation and administration: scenarios enacted directly with children and/or their entourage, scenarios enacted with the support of figurines, pictures, videos or audio-recordings, games played between the experimenter and the child, videogames, and so on. Measures have also been created or adapted for use with different populations: 40 different languages and 30 distinct adverse conditions are reported (e.g., hearing impairments, visual impairments, autism spectrum disorders).

Given that many other social measures have been limited to questionnaires (Crowe et al., 2011), it is somewhat surprising that only four adult-report questionnaires were found that measure TOM in young children, and these were only used in 2.4% of studies. Direct testing with children is therefore prominent in TOM research and represents a strength of the field, given that direct, laboratory testing provides an explicit opportunity for observing children's responses and may reduce bias associated with parental reports. However, sole reliance on direct testing may also have limits, because it depends on a single context (laboratory) and a single source of information (child) (Carlson et al., 2013). Given that triangulation of data is of importance in clinical (American Psychiatric Association, 2013; American Educational Research Association, A. P. A., and National Council on Measurement in Education, 2014) and research settings (Tashakkori and Teddlie, 2010), and that TOM abilities exhibited in the laboratory are not consistently applied in everyday life (Happé et al., 2017), collecting third party observations on children's natural functioning in social environments via questionnaires or interviews could provide additional information on the behavioral manifestations of TOM. Moreover, initial psychometric data on these questionnaires supports their convergent construct validity. Specifically, each of the four questionnaires was reported to correlate with TOM direct testing scores (Hughes et al., 1997; Comte-Gervais et al., 2008; Hutchins et al., 2008a, 2012; Peterson et al., 2009; Houssa et al., 2014; Tahiroglu et al., 2014). Other promising avenues to conduct ecological evaluation are related to the use of virtual reality and naturalistic, real-world observations of children's behavior, approaches that have seldom been used to date, but that may become more feasible as technology advances and with greater awareness of the importance of the use of real social stimuli in social cognitive assessment (Beauchamp, 2017).

#### Enrichment of Measurement Tools

This literature review portrays the structure of TOM measures used to date. Many measures reviewed here rely on only one or two test items when measuring a specific ability, essentially creating a “pass or fail” situation for the examinee, a problem that has also been raised by others (Cutting and Dunn, 1999; Garner et al., 2005). Such tools offer little score variation and sensitivity to qualify participants' social competence. As with other cognitive functions, TOM should be situated on a continuum and not treated dichotomously (capable or incapable). The need to collect a sample of items large enough to represent any psychological construct is a well-recognized issue in the establishment of adequate content validity and reliability (Slick, 2006; American Educational Research Association, A. P. A., and National Council on Measurement in Education, 2014). The numerous measures listed in this review provide several examples of tests and test items that could be used in order to enrich the evaluation on any TOM category or sub-ability.

#### Standardization of TOM Assessment

There is a sizeable number of variations in single tasks across studies. Synthesizing the data extracted in this review presented a significant challenge, owing to the numerous “free” adaptations of unique measures found in the literature. This added a layer of complexity when deciding whether an adaptation of a measure or paradigm should be seen as distinct from the original or not. The wide assortment of TOM measures leads to poor comparability across studies (Hiller et al., 2014) and can be detrimental to the reliability of results (Slick, 2006). For example, success on false belief paradigms may vary as a function of seemingly superficial aspects of the task, such as the type of material used (e.g., is it familiar to the child or new?; Adrien et al., 1995; Cassidy, 1998), the characters presented (e.g., are they real people or figurines?; Battacchi et al., 1997), and subtle differences in language used to question the child (e.g., positive or negative sentence?; Abu-Akel and Bailey, 2001; Geangu, 2002). These task variations constitute a challenge for researchers and clinicians seeking to identify the best measures among all existing task variations found in the literature.

#### Psychometric Properties of TOM Measures

This systematic review confirms some of the critiques that have been raised regarding TOM psychometry (Hutchins et al., 2008a; Hiller et al., 2014; Ziatabar Ahmadi et al., 2015). Notably, insufficient TOM measures have empirically validated psychometric properties: internal structure or internal consistency information was available for 37 measures, inter-rater reliability information was available for 62 measures, test-retest reliability was available for 18 measures, other psychometric information, including validity hypothesis testing, was available for only 27 measures. While presenting interesting inter-rater reliability data, implicit methods to measure TOM failed to provide any information on test-retest reliability and are challenged by independent replication studies suggesting globally poor replicability. It should be noted that the current study was not intended to comprehensively review and critique psychometric properties of TOM measures to provide guidelines for measure selection. This objective would require a specific methodology, including assessing study quality and reporting separate psychometric properties for different versions of the same tasks. The readers are thus invited to exercise caution when interpreting the psychometric data included in this review. Nevertheless, the summary tables included here provide basic information to begin a more detailed search of published psychometric properties for TOM measures. While pursuing such a search, readers should exert their judgment regarding the methodological quality of the validation studies, since the same psychometric property may be more or less powerful depending on study design (e.g., number of participants) and measure characteristics (e.g., number of items). Guidelines for evaluating the quality of tools, such as those published by Terwee et al. (2007), may be useful as they list psychometric properties and gold standard validation methodologies. The psychometric properties reported are likely only to reflect the properties of the specific version of the measure used in a particular study, and not necessarily other adaptations of the measure. Finally, lack of psychometric properties for a specific measure in the results tables does not necessarily reflect disregard of their importance on the part of the authors; some describe psychometric properties of aggregates of single measures (e.g., Yagmurlu et al., 2005; Guajardo et al., 2013), and these were not included in the current review since they did not refer to a specific measure.

### Limitations

The results of this systematic review should be interpreted in the context of certain limitations. First, given the large amount of search results obtained via electronic databases, publishers' catalogs and other sources (3,207 records), additional searches of the gray literature, such as screening of the references in the 830 articles was not performed, even though it is possible that this may have revealed additional measures or additional information on the measures listed herein (Moher et al., 2015). Second, despite the numerous search terms used, the selection of keywords and truncations to capture related terms, and the large amount of measures and articles found, the search strategy failed to retrieve a few pertinent articles that fit the inclusion criteria (e.g., Chen and Lin, 1994; Meltzoff, 1995; Tardif et al., 2004). This is likely due to a lack of common vocabulary in the field, with authors using different terms to refer to similar constructs somewhat interchangeably (i.e., “mentalizing,” “mind-reading,” and “theory of mind”; Happé et al., 2017). Third, the theoretical model selected to define TOM (SOME model; Bird and Viding, 2014) necessarily determined the inclusion and exclusion criteria for the review. As such, the review may have excluded measures that would have been identified as TOM tools using other models/definitions. In particular, implicit measures of the ability to infer mental states in others, often used with children under 2 years, were only partially captured (see Scott and Baillargeon, 2017 for a review of non-traditional and implicit methods used to measure TOM). Moreover, measures that were judged to primarily assess classification of affective cues (e.g., Reading the mind in the eyes task; Baron-Cohen et al., 1997) and cooperation and competition tasks were not included (e.g., Window task; Russell et al., 1991), nor were those that document the use (e.g., number of mental state terms used by the child; Internal state language questionnaire, Bellagamba et al., 2014) and understanding (e.g., understanding the difference between the words “know” and “believe”; Certainty task, Adrian et al., 2007) of mental state language, or children's verbal explanations when faced with TOM paradigms (e.g., Peskin and Astington, 2004; Veneziano and Christian, 2006). Fourth, this review did not cover “control tasks,” that is, tasks that match TOM tasks in terms of cognitive demands and modes of presentation, but that do not require mental state inferences. For example, there exists a control task for the change-in-location paradigm called the Natural false sign location (e.g., Lackner et al., 2012). The use of control tasks is increasingly recommended in order to take into account the confounding effect of general cognitive abilities and to identify specific social cognition impairments (Henry et al., 2016).

### Conclusions

This systematic review of TOM measures destined for young children identified 830 articles and 220 measures published in the last 36 years that have been administered in 40 different languages and in the context of 30 different medical, psychological and environmental adverse conditions, confirming the preponderance of TOM in many domains of research and practice. The detailed inventory of TOM measures is accompanied by a TOM taxonomy (ATOMS), which presents categories of mental states and social situation understanding that have been used in published research with young children. The findings associated with the review underscore a number of important challenges in TOM assessment. Given that interest in TOM and associated social cognitive constructs is pervasive across developmental psychology, neuropsychology, social psychology, educational psychology and social neuroscience research, and that the need to assess and intervene within these domains is now recognized clinically (Steerneman et al., 1996; Sprung, 2010; Hoddenbach et al., 2012; Lecce et al., 2014; Henry et al., 2016; Beauchamp, 2017), this inventory of TOM measures contributes to both fundamental science and clinical practice.

## Data Availability Statement

All datasets generated for this study are included in the article/Supplementary Material.

## Author Contributions

CB, CG, and MB contributed to the conception and design of the study. CB, ÉL, and CG collected and analyzed the data. CB wrote the first draft of the manuscript. All authors contributed to manuscript revision, read, and approved the submitted version. MB supervised the study.

### Conflict of Interest

The authors declare that the research was conducted in the absence of any commercial or financial relationships that could be construed as a potential conflict of interest.
