# Supplementary material for: Systematic Review and Inventory of Theory of Mind Measures for Young Children
Source: Front Psychol. 2020 Jan 15;10:2905. doi: 10.3389/fpsyg.2019.02905 (PMC6974541; doi:10.3389/fpsyg.2019.02905)
Supplement: Supplementary file 1 [file Table_1.pdf]

Appendix II

Table a.

Measures related to TOM- Emotions category

	Measures characteristics						Participants characteristics	
**TOM abilities**	**Measures (source author, year) [articles using this measure]**	**Availability of psychometric information**	**Modes of presentation**	**Number of items**	**Scoring options**	**Age ranges in TD population (months)**	**Languages**	**Adverse conditions**
Typical emotional reactions: Inferring a person's emotional reactions based on situations that typically elicit certain emotions/ inferring a preceding event based on a person's emotional reaction	a) Affective perspective taking (Cassidy, Parke, Butkovsky, and Braugart, 1992) [1-7]	a) +	Direct testing using interview, short stories read aloud or audiotaped with or without pictures or photographs, computerized games	1 to 54	Correct/ incorrect, rating scales or coding of number of emotions named by the child, coding of child's explanations	a) 36-71	Chinese [8]<br>Dutch [9-12]<br>French [13-22]<br>German [23]<br>Hebrew [24, 25]<br>Italian [26-30]<br>Japanese [31]<br>Norwegian [22, 32-34]<br>Portuguese [35]<br>Spanish [26, 36-39]	ASD [22, 26, 35, 40-43]<br>ADHD [40]<br>Behavior problems [44]<br>Hearing impairments or deafness [10]<br>Intellectual disability or develop-mental delay [13-16, 18, 19, 43, 45]<br>Low birth weight [46]<br>Low SES [5, 37, 40, 47]<br>ODD [40]<br>Prader-Willi syndrome [45]<br>William syndrome [45]
	b) The embarrassment task (Colonnesi, 2010) [9]	b) -				b) 48-108		
	c) Knowledge of emotion cause (Denham, Zoller & Couchoud, 1994) [8, 48]	c) +				c) 35-132		
	d) Simpe causal relations between antecedent event and subsequent emotion (Subtask 3 from the Perspective taking task; (Edelstein, Keller & Wahlen; 1984) [377]	d) -				d) 60-120		
	e) Description of emotional situation (Feshbach & Cohen, 1988) [204]	e) +				e) 36-71		
	f) Emotion situation knowledge task (Garner et al.,1994) [227, 622]	f) +				f) None		
	g) Emotional perspective taking task (Harwood & Farrar, 2006) [37, 194, 264]	g) -				g) 36-76		
	h) Emotion understanding assessment (Howlin, Baron-Cohen, & Hadwin, 1999) [21, 112, 170, 171, 598, 647, 648, 740, 752]	h) +				h) 36-73		
	i) Parent-child affective perspective-taking scale (MacQuiddy, Maise & Hamilton, 1987) [406]	i) -				i) 67-96		
	j) Gossip story (Example 3 from the TOM test; Muris, Steerneman, Meesters, Merckelbach, Horselenberg, van den Hogen & van Dongen, 1999)[466]	j) -				j) 60-144		
	k) The cause of emotion task (Nader-Grosbois, Thirion-Marissiaux & Grosbois, 2008, Unpublished manual) [13-20]	k) -				k) 25-83		
	l) Affective perspective taking (Paulus & Leitherer, 2017) [23]	l) -				l) 57-72		

	m) External cause of emotion (Pons & Harris, 2000; Pons, Harris & Rosnay, 2004) [11, 12, 22, 26-30, 32-34, 39, 49-53]	m) -				m) 31-132		
	n) Reminder-based emotion (Pons & Harris, 2000; Pons, Harris & Rosnay, 2004) [22, 26-30, 32, 49, 52, 53]	n) -				n) 36-132		
	o) Emotion recognition questionnaire (Ribordy, Camras, Stefani, & Spaccarelli, 1988) [24, 25, 54-56]	o) +				o) 36-72		
	p) Social behavior and social situation subscales from the Assessment of children's emotion's skills (Schultz, Izard and Bear, 2004) [57]	p) -				p) 41-61		
	q) Emotions stories (Tager-Flusberg & Sullivan, 1994) [43, 45]	q) -				q) 39-123		
	r) Emotion-situation task (Taumoepeau & Ruffman, 2006) [31, 58]	r) -				r) 23-39		
	s) Emotion attribution in prototypical situation (Wiefferink et al., 2013)[10, 59, 60]	s) -				s) 30-77		
Atypical emotional reactions: Inferring or explaining a person's emotional reactions based on situations eliciting emotions that are atypical compared to what is usually expected	a) Incongruent Expressions Task (Burns and Cavey, 1957) [61, 62]	a) -	Direct testing using figurines or pictures	1 to 23	Correct/ incorrect or rating scales, coding of children explanations	a) 24-*M* =78	Hebrew [63]	ASD [67, 68]
	b) Affective perspective-taking tests (Denham, 1986) [2, 3, 5, 6, 48, 50, 63-66, 69-95]	b) +				b) 24-96	Italian [64, 65]<br>Mandarin [66]	Anxiety or depression [69]<br>Behavior problems [69]
	c) Comprehension test and Unexpected outcomes test from the Emotion Recognition Scale (Dyck et al, 2001) [67, 68]	c) -				c) None		Developmental coordination disorder [67]
	d) Comprehension of complex or multiple psychological causality (Subtask 5 from the Perspective-taking task; Edelstein, Keller & Wahlen; 1984) [96]	d) -				d) 60-120		Language impairments [67]<br>Low SES [70, 71]
	e) Emotion prediction and explanation task (Gnepp and Chilamkurti, 1988) [97]	e) -				e) 64-99		Maltreatment [70]
	f) Affective attribution and reasoning task, (Iannotti, 1978) [98]	f) +				f) *M* = 68-96		
Discrepant emotions: Understanding that people may have discrepant feelings about an event	a) Affective perspective taking (Borke, 1971) [61, 99-101]	a) -	Direct texting using pictures	8	Rating scale or correct/incorrect	a) 24-96		Externalizing behavior problems [100]
Mixed emotions: Understanding that people may feel mixed emotions or different emotions successively	a) Surprise tasks (Gopnik & Astington) [102]	a) -	Direct testing under the form of a story-telling interview, pictures or using child and experimenter as protagonists	1 to 6	Correct/ incorrect, rating scale or coding of children's answer/ explanations	a) 42-54	French [22]	ASD [22, 26]
	b) Mixed emotion understanding task (Gordis, Rosen, and Grand, 1989) [2, 103-106]	b) +				b) 54-89	Italian [22, 26-30, 103-106]<br>Korean [107]	
	c) Emotion subtask from the Perspective taking ability test (Hudson, Forman, and Brion, 1982; Shin, 1996) [107]	c) -				c) 60-71	Norwegian [32]	
	d) Mixed emotions (Pons & Harris, 2000, Pons, Harris & Rosnay, 2004) [22, 26-30, 32, 49, 53]	d) -				d) 36-132	Spanish [26]	
Hidden emotions: Understanding that	a) Emotional and emotive faces task (Dennis et al., 2013) [108]	a) -	Direct testing using read-aloud	1 to 10	Correct/	a) 63-184	Bahasa Indonesia [109]	ADHD [42]

other people may hide their emotions	b) Appearance reality of emotions (Harris, Donnelly, Guz, and Pitt-Watson, 1986); Affective false-belief task (Davis, 1998) [4, 22, 23, 26-30, 32, 39, 49-53, 72, 79, 81, 88, 89, 94, 109-112, 114-149, 151-193]	b) +	stories, audio recordings and/or using pictures or figurines		incorrect or coding of children explanations	b) 24-168	Bislama [110]<br>Chinese [111, 112]<br>Dutch [113]<br>Farsi [114-116]<br>French [22, 117, 118]<br>German [23, 119-122]<br>Italian [22, 26-30]<br>Mandarin [123-125]<br>Nakanamanga [110]<br>Norwegian [32]<br>Polish [126, 127]<br>Spanish [26, 39, 128-130]<br>Turkish [131, 132]	ASD[22, 26, 42, 111, 133-140]<br>Anxiety [42]<br>Down syndrome [134, 141]<br>Hearing impairment or deafness [112, 126, 127, 133, 142-144]<br>Intellectual disability or developmental delay [126, 127]<br>Language impairment [119, 145]<br>Low birth weight [146]<br>Low SES [147]<br>Maltreatment [148]<br>Pregnancy hypertension [149]<br>Prematurity [122]<br>TBI [150]
	c) Display rules (Ketelaars, van Weerdendurg, Verhoeven, Cuperus & Jansonieus, 2010) [113]	c) -				c) 59-99		
	d) Hurt story (Example 4 from the TOM test; Muris, Steerneman, Meesters, Merckelbach, Horselenberg, van den Hogen & van Dongen, 1999)[42]	d) -				d) 60-144		
Moral emotions: Understanding that negative feelings might arise following a reprehensible action	a) Morality-based emotion (Pons & Harris, 2000, Pons, Harris & Rosnay, 2004)[22, 26-30, 32, 49]	a) -	Direct testing using pictures	1	Correct/incorrect	a) 36-132	French [22]<br>Italian [22, 26-30]<br>Norwegian [32]<br>Spanish[26]	ASD [22, 26]
Emotion regulation: Undestanding that others might use strategies to regulate their emotions	a) Regulation of emotion (Pons & Harris, 2000; Pons, Harris & Rosnay, 2004)[22, 26-30, 32, 49, 53]	a) -	Direct testing using pictures	1	Correct/incorrect	a) 36-132	French [22]<br>Italian [22, 26-30]<br>Norwegian [32]<br>Spanish [26]	ASD [22, 26]
Comprehensive measure involving emotion understanding based on different factors/TOM categories (e.g., desires, beliefs, hiding emotions)	a) Test of emotion comprehension (Pons & Harris, 2000; Pons, Harris & Rosnay, 2004) [22, 26-30, 32, 39, 49-53, 187, 194, 195]	a) +	Direct testing using pictures	3 to 38	Correct/incorrect or rating scale	a) 36-126	Italian [22, 26, 27, 29, 30]<br>Norwegian [32]<br>Spanish [26, 28, 39]	ASD [22, 26]

Note. **Measures:** when authors provided no name for their measure, it was named according to its content in order to facilitate identification within the tables. The original source of a measure, written in brackets, may have not been included in the review and is provided to facilitate identification of measures. For a single article, there may be several variations of the same measure (e.g., different tasks using a classic change-in-location paradigm). **Availability of psychometric information**: + = information provided; – = No information provided (see Table X). **Age range in TD population**: "None" signifies the absence of

TD children in the studies using the measure. "*M*" signifies the mean age of the sample and is presented only when no age range could be retrieved. **Languages**: Languages other than English in which the measure was administered, as reported in the studies. When the study specified no language of administration, nor language spoken by participants, it was assumed that it was administered in the language the article was written in. **Adverse conditions:** adverse clinical, psychological or environmental conditions. Children presenting adverse conditions may have different age ranges than the ones in TD population provided in the table.

Abbreviations: TD = Typically developing; SES = Socioeconomic Status; ASD = Autism spectrum disorders; ADHD = Attention-deficit/hyperactivity disorder; ODD = Oppositional defiant disorder; TBI = Traumatic brain injury

Table b.

Measures related to TOM- Desires category

	Measures characteristics					Participants characteristics		
**TOM abilities**	**Measures (source author, year) [articles using this measure]**	**Availability of psychometric information**	**Modes of presentation**	**Number of items**	**Scoring options**	**Age range in TD population (months)**	**Languages**	**Adverse conditions**
Discrepant desires: Understanding that different people may have discrepant desires	a) Charlie test/Four sweets task (Baron-Cohen et al., 1995) [196-199]<br>b) Diverse desire (Bartsch & Wellman, 1989) [218, 227, 228]<br>c) Matters of taste (Carpendale & Chandler, 1996) [229]<br>d) Gift Task (Flavell, 1968)/ Gift selection task (Jin et al, 2017) [230] [202]<br>e) Social activity desires task (Nguyen & Frye, 1999) [231]<br>f) Discrepant desires Yummy-yucky task (Repacholi & Gopnik, 1997) [206-214, 225, 232-234]<br>g) Common and uncommon desires (Rieffe et al., 2001) [59, 60, 203-205, 235]<br>h) Conflicting emotion task (Slaughter, Dennis and Pritchard, 2002) [198]<br>i) Desire task (Slaughter, Dennis and Pritchard, 2002)/Other person's desire' situation (Arranz, Artamendi, Ollabarieta, 2002) [198, 200, 220]<br>j) Not own desire tasks (Wellman & Wooley, 1990) [4, 23, 51, 65, 109-112, 114-127, 129, 131-146, 148, 149, 158-166, 168-174, 176-186, 188-191, 201, 215-217, 219, 221-224, 226, 227, 236-251]	a) -<br>b) +<br>c) -<br>d) +<br>e) -<br>f) +<br>g) +<br>h) -<br>i) -<br>j) +	Direct testing using figurines, pictures, audio recordings, video or spoken narration; or using the child and experimenter as protagonists	1 to 12	Correct/ Incorrect or coding of affect, timing of affect, looking gazes, behavior or verbal answers	a) 36-84<br>b) 28-42<br>c) 58-107<br>d) 48-66<br>e) 36-73<br>f) 13-36<br>g) 12-77<br>h) 53-72<br>i) 37-81<br>j) 18-168	Bahasa Indonesia [109]<br>Basque [200]<br>Bislama [110]<br>Brazilian Portuguese [201]<br>Chinese [111, 112, 202]<br>Dutch [203-205]<br>Farsi [114-116]<br>French [117, 118, 206-213]<br>German [23, 119-122, 214-217]<br>Italian [65]<br>Japanese [218]<br>Mandarin [123-125, 219]<br>Nakanamanga [110]<br>Polish [126, 127]<br>Spanish [129]<br>Turkish [131, 132]	ASD [111, 133-140, 144, 197, 199, 220-224]<br>Developmental disabilty of mixed etiologies [225]<br>Down syndrome [134, 141]<br>Fragile X syndrome [223]<br>Hearing impairments or deafness [112, 126, 127, 133, 142-144, 203, 205, 219, 226]<br>Intellectual disability or developmental delay [126, 127, 199]<br>Language impairments [119, 145, 197]<br>Low birth weight [146]<br>Maltreatment [148]<br>Pregnancy hypertension [149]<br>Prematurity [122]<br>TBI [210, 212]<br>Williams syndrome [225]

Multiple desires: Understanding the co-existence of multiple desires simultaneously or successively in one person	a) Desires and beliefs task (Bennett & Gasper, 1993) [252]<br>b) Multiple desires task (Bennett & Gapert, 1993) [252]<br>c) Successive Desires task (Bennett & Gapert, 1993) [252]<br>d) Desire task (Gopnik & Slaughter, 1991) [102, 254]<br>e) Conflicting-desires tasks (Moore et al.,1995) [253, 255]	a) -<br>b) -<br>c) -<br>d) -<br>e) -	Direct testing using stories read aloud with or without photographs or figurines, or games	1 to 4	Correct/incorrect	a) 69-90<br>b) 64-141<br>c) 62-99<br>d) 42-54<br>e) 37-64	German [253]	
Desires influence on emotions and actions: Understanding that people's emotions and actions are influenced by their desires/ preferences	a) Desires based emotions (Hadwin, Baron-Cohen, Howlin & Hill, 1996)[20, 256-260]<br>b) Desire based emotion (Pons & Harris, 2000, Pons, Harris & Rosnay, 2004) [22, 26-30, 32, 39, 49-53, 187]<br>c) Desire and intention task (Schult, 2002) [268, 269]<br>d) Target-hitting game (Schult, 2002) [269]<br>e) Desires stories (Tager-Flusberg & Sullivan, 1994)[43, 45]<br>f) Desire task (Terwogt & Rieffe, 2003) [261]<br>g) Want stories (Wellman & Bartsch, 1988) [270]<br>h) Desire task (actions and emotions stories) (Wellman & Woolley, 1990) [11, 12, 22, 31, 33, 34, 64, 70, 71, 77, 79, 200, 208, 210, 212, 232, 236, 263-267]<br>i) No preference desire task (Wellman & Wooley, 1990) [236]<br>j) Wicked desires task (Yuill, Perner, Pearson, Peerbhoy & van den Ende, 1996) [77, 79, 271, 272]	a) -<br>b) -<br>c) +<br>d) +<br>e) -<br>f) -<br>g) -<br>h) +<br>i) -<br>j) -	Direct testing using read-aloud stories, figurines, pictures or games	1 to 9	Correct/ incorrect or rating scales	a) 24-165<br>b) 36-132<br>c) 60-120<br>d) 36-72<br>e) 39-123<br>f) 48-71<br>g) 50-348<br>h) 24-132<br>i) 33-39<br>j) 30-120	Basque [200]<br>Dutch [11, 12, 261]<br>French [20, 22, 208, 210, 212, 262]<br>Italian [22, 26-30, 33, 34, 64]<br>Japanese [31]<br>Norwegian [32]<br>Polish [263]<br>Spanish [26, 39]	ASD [22, 26, 43, 256-258, 264-267] [256-260]<br>Intellectual disabilities or developmental delay [43, 45]<br>Low SES [70, 71]<br>Maltreatment [70]<br>Prader-Willi syndrome [45]<br>TBI [210, 212]<br>Williams syndrome [45]
Desire-action contradiction: Producing plausible explanations when actions contradicts stated desires/preferences	a) Anomalous-desires stories (Colonnesi, Rieffe, Koops & Perucchini, 2008) [273]	a) -	Direct testing using read-aloud stories and pictures	3	Coding of children's explanations/ answers	a) 38-40		

Note. **Measures:** when authors provided no name for their measure, it was named according to its content in order to facilitate identification within the tables. The original source of a measure, written in brackets, may have not been included in the review and is provided to facilitate identification of measures. For a single article, there may be several variations of the same measure (e.g., different tasks using a classic change-in-location paradigm). **Availability of psychometric information**: + = information provided; – = No information provided (see Table X). **Languages**: Languages other than English in which the measure was administered, as reported in the studies. When the study specified no language of administration, nor language spoken by participants, it was assumed that it was administered in the language the article was written in. **Adverse conditions:** adverse clinical, psychological or environmental conditions. Children presenting adverse conditions may have different age ranges than the ones in TD population provided in the table.

Abbreviations: TD = Typically developing; SES = Socioeconomic Status; ASD = Autism Spectrum Disorders; TBI = Traumatic brain injury.

Table c.

Measures related to TOM- Intentions category

	Measures characteristics					Participants characteristics		
**TOM abilities**	**Measures (source author, year) [articles using this measure]**	**Availability of psychometric information**	**Modes of presentation**	**Number of items**	**Scoring options**	**Age range in TD population (months)**	**Languages**	**Adverse conditions**
Completion of failed actions: Understanding another person's intent, as demonstrated by completing their failed action	a) Behavioral re-enactment procedure (Meltzoff, 1995) [203, 205, 213, 233, 273-280]	a) +	Direct testing, using videos or the child and the experimenter as protagonists	2 to 9	Correct/ incorrect, coding of the child behavior	a) 9-70	Nonverbal task	ASD [278]<br><br>Developmental disabilities of mixed etiologies [274]<br><br>Down syndrome [274]<br><br>Hearing impairments or deafness [203, 205]<br><br>Intellectual disability or developmental delay [278]<br><br>Language impairments [274]
Discrepant intentions: Understanding that identical actions/results can be achieved with different intentions	a) Behavior-, skill- and awareness-intentionality measures (Astington & Lee, 1991) [281]<br>b) Intention tasks (Browne & Woolley, 2001) [284, 285]<br>c) Intention and beliefs (Choi & Luo, 2015) [286]<br>d) Intention task (Gopnik & Slauther, 1991) [102]<br>e) Accidental transgression task (MoToM; Killen, Lynn Mulvey, Richardson, Jampol & Woodward, 2011) [214, 282, 287, 288]<br>f) Transparent intention task (Russell, Hill, and Franco, 2001) [273, 283, 289]<br>g) Knee-jerk task (Williams & Happé, 2010) [283]	a) +<br>b) -<br>c) +<br>d) -<br>e) +<br>f) -<br>g) -	Direct testing using figurines, pictures, audio recordings, and/or videos	1 to 8	Correct/ incorrect, likert scale or coding of children's explanations	a) 38-119<br>b) 40-97<br>c) 11-14<br>d) 42-54<br>e) 35-119<br>f) 36-58<br>g) *M* = 53.52	Cantonese [282]<br><br>German [214]<br><br>Italian [273]	ASD [283]<br><br>Developmental disabilities of mixed etiologies [283]
Prediction of actions: Predicting people's	a) Picture sequencing task (Baron-Cohen, Leslie & Frith, 1986) [290-294]	a) -	Direct testing using stories read	1 to 48	Correct/ incorrect and/or coding of	a) 41-140		ASD [290]

actions based on their intentions	b) Visual habituation paradigm (Buresh & Woodward, 2007) [214, 296]<br>c) Intention task (Phillips & Wellman, 2005) [211, 213, 297]<br>d) Attention to intention (Phillips, Wellman & Spelke, 2002) [170, 298]<br>e) Visual and verbal attribution of intention tasks (Santos & Deruelle, 2009) [295]	b) +<br>c) +<br>d) +<br>e) -	aloud, videos, pictures or live actors		looking gazes or child's narration	b) 6-12<br>c) 11-20<br>d) 8-14<br>e) 48-204	French [211, 213, 290, 292, 295]<br>German [214]	Down syndrome [291]<br>Language impairment [292]<br>Williams syndrome [291, 295]
Intention attribution to visual figures: Tendency to attribute intentions to ambiguous visual figures	a) Visual habituation study using videos of two balls (Gergely, Nadasdy, Csibra & Biro, 1995) [299]	a) -	Direct testing using videos	4	Coding of looking gazes	a) 11-16		
Intention explanations: Producing plausible intention explanations for different types of observed social events	a) Intention subtask from the Perspective taking ability test (Hudson, Forman, and Brion, 1982; Shin, 1996) [107]<br>b) Intentions explanations (Smiley, 2001) [300]	a) -<br>b) -	Direct testing using videos or pictures	8	Correct/incorrect, rating scales or coding of children's explanations/ answers	a) 60-71<br>b) 19-39	Korean [107]	

Note. **Measures:** when authors provided no name for their measure, it was named according to its content in order to facilitate identification within the tables. The original source of a measure, written in brackets, may have not been included in the review and is provided to facilitate identification of measures. For a single article, there may be several variations of the same measure (e.g., different tasks using a classic change-in-location paradigm). **Availability of psychometric information**: + = information provided; – = No information provided (see Table X). **Age range in TD population**: "*M*" signifies the mean age of the sample and is presented only when no age range could be retrieved. **Languages**: Languages other than English in which the measure was administered, as reported in the studies. When the study specified no language of administration, nor language spoken by participants, it was assumed that it was administered in the language the article was written in. **Adverse conditions:** adverse clinical, psychological or environmental conditions. Children presenting adverse conditions may have different age ranges than the ones in TD population provided in the table.

Abbreviations: TD = Typically developing; ASD = Autism Spectrum Disorders.

Table d.

Measures related to TOM- Percepts category

	Measures characteristics					Participants characteristics		
**TOM abilities**	**Measures (source author, year) [articles using this measure]**	**Availability of psychometric information**	**Modes of presentation**	**Number of items**	**Scoring options**	**Age range in TD population (months)**	**Languages**	**Adverse conditions**
Simple visual perspective taking: Acknowledging that others have different visual percepts and adopting the visual perspective of another person	a) Visual perspective taking (Carlson et al., 2004) [206, 209, 233, 239, 301, 302]	a) -	Direct testing using figurines, pictures or a game between the child and experimenter/ parent	1 to 14	Correct/ incorrect or coding of children behavior/ instructtions/ explanations/ looking gazes	a) 12-31	Cameroonian [303]<br>Dutch [217]<br>French [13, 15-17, 19, 20, 206, 208, 209, 301, 304]<br>German [303, 305]<br>Italian [29, 273]<br>Norwegian [306]<br>Pacific Islandic languages [307]<br>Spanish [303]	ASD [136, 220, 224, 264, 266, 267, 308-310]<br>Down syndrome [15, 16]<br>Externalizing behavior problems [13, 73, 74]<br>Hearing impairment {Kusché, 1983 #542}<br>Intellectual disability or developmental delay [13, 19, 304, 310]<br>Language impairments [145, 232]<br>Low SES [311-316]<br>Maltreatment [70]<br>Sensory integration deficits [317]<br>Visual impairments or blindess [318]
	b) Non-deceptive, explicit deception and implied deception task (Dalke, 1995) [319]	b) -				b) 40-59		
	c) Visual perspective taking, level 1/Picture identification task (Flavell et al., 1968; 1981) [17, 19, 20, 48, 61, 70, 71, 102, 145, 173, 208, 220, 232, 233, 273, 306, 320-325]	c) -				c) 24-82		
	d) Move object task (Flavell, Shipstead, & Croft, 1978) [217, 307, 326, 327]	d) -				d) 26-48		
	e) Seeing tasks (Gonzales, Fabricius & Kupfer, 2017) [328]	e) -				e) 24-82		
	f) Penny game task (Gratch, 1964) [13, 15-17, 19, 20, 73, 74, 222, 224, 230, 249, 264, 266, 267, 303, 304, 307-317, 329-333] {Kusché, 1983 #542}	f) +				f) 25-192		
	g) Policeman tasks (Hughes & Donaldson, 1979) [29, 334]	g) -				g) 37-74		
	h) Visual perspective taking, level 2 (Masangkay, 1974; Flavell et al.,1981) [13, 15-17, 19, 20, 29, 48, 95, 102, 145, 153, 173, 206, 208, 220, 232, 233, 239, 243, 302, 304, 318, 320, 322-324, 335-337]	h) -				h) 20-84		
	i) Gaze-following task (Meltzoff et Brooks, 2008) [338]	i) +				i) 11-18		
	j) Occluded object task (Moll and Tomasello, 2006) [305, 307, 339]	j) +				j) 17-30		
	k) Spatial view point of the other (Peisach & Hardeman 1985) [340]	k) -				k) *M* = 58-84		
	l) Hide and seek (Peskin & Ardino, 2003) [341]	l) -				l) 36-69		
	m) Level-1 perspective taking tasks (Ricard et al., 1999) [342]	m) +				m) 18-30		
	n) Blow-football game (Russell, Alexis & Clayton, 2010) [343]V	n) -				n) 36-71		

	o) Level 1 perspective taking (Sodian, Thoermer, and Metz, 2007) [217, 344]	o) -				o) 12-*M* = 15		
Complex visual perspective taking: Adopting another person's visual perspective in tasks demanding complex mental rotation or visualisation	a) Visual perspective taking and spatial construction task (Ebersbach, Stiehler & Asmus, 2011) [345]<br>b) Referential communication game (Epley, Morewedge & Keysar, 2004) [348]<br>c) Village construction task (Doise & Mugny, 1984) [349]<br>d) Photographers perspective taking (Frick, Mohring & Newcombe, 2014) [350]<br>e) Level 2 visual perspective taking (Hamilton, Brindley & Frith, 2009) [136, 224, 347]<br>f) Array visual perspective taking task (Langdon and Coltheart's, 2001) [145]<br>g) Comprehension task (Nadig and Sedivy, 2002) [351, 352]<br>h) Visual perspective-taking circle (Newcombe and Huttenlocher, 1992) [346, 353, 354]<br>i) Big bird visual perspective taking (Rosser, Chandler & Lane, 1993) [355]	a) +<br>b) -<br>c) -<br>d) +<br>e) -<br>f) -<br>g) -<br>h) -<br>i) -	Direct testing using figurines or experimenter and child as protagonists	4 to 15	Correct/ incorrect or coding of looking gazes, constructions or verbal answers	a) *M* = 60- 108<br>b) 48-144<br>c) 63-78<br>d) 48-108<br>e) 45-482<br>f) 51-70<br>g) 44-82<br>h) 42-78<br>i) 48- 120	German [345]	ASD [136, 224]<br>Language impairments [145]<br>Williams syndrome [346, 347]
Percept-action link: Understanding that other's actions are linked to their visual percepts	a) Perception based action (Hadwin, Baron-Cohen, Howlin & Hill, 1996) [20, 256-260]	a) -	Direct testing using pictures	1	Correct/incorrect	a) 24-165	French [20]	ADHD [259]<br>ASD [256-260]
Auditory perspective taking: Considering the auditory percepts of another person	a) Auditory perspective taking (Williamson, Brooks, & Meltzoff, 2015) [356]	a) -	Direct testing using figurines	1	Correct/incorrect	a) 26-37		

Note. **Measures:** when authors provided no name for their measure, it was named according to its content in order to facilitate identification within the tables. The original source of a measure, written in brackets, may have not been included in the review and is provided to facilitate identification of measures. For a single article, there may be several variations of the same measure (e.g., different tasks using a classic change-in-location paradigm). **Availability of psychometric information**: + = information provided; – = No information provided (see Table X). **Age range in TD population**: "*M*" signifies the mean age of the sample and is presented only when no age range could be retrieved. **Languages**: Languages other than English in which the measure was administered, as reported in the studies. When the study specified no language of administration, nor language spoken by participants, it was assumed that it was administered in the language the article was written in. **Adverse conditions:** adverse clinical, psychological or environmental conditions. Children presenting adverse conditions may have different age ranges than the ones in TD population provided in the table.

Abbreviations: TD = Typically developing; SES = Socioeconomic Status; ASD = Autism Spectrum Disorders; ADHD= Attention deficit/hyperactivity disorder

Table e.

Measures related to TOM- Knowledge category

	Measures characteristics					Participants characteristics		
**TOM abilities**	**Measures (source author, year) [articles using this measure]**	**Availability of psychometric information**	**Modes of presentation**	**Number of items**	**Scoring options**	**Age range in TD population (months)**	**Languages**	**Adverse conditions**
Knowledge-pretend play links: Understanding that someone who does not know something exists cannot engage in "pretend play" that incorporates that knowledge	a) Sarah task (Aronson & Golomb, 1999) [357]<br>b) Pretense tasks (Lillard, 1993) [357-359]<br>c) Awareness task (Sobel, 2004) [359]	a) -<br>b) -<br>c) -	Direct testing using pictures or figurines	1	Correct/ incorrect	a) 47-70<br>b) 45-96<br>c) 48-96		
Percepts-knowledge links: Understanding that someone who does not have access to perceptual information (i.e. by looking, hearing, etc.) may not have access to knowledge	a) Theory of social mind (Abrams, Rutland, Pelletier & Ferrell, 2009) [360]<br>b) Knowledge-attribution task (Birch & Bloom, 2003) [376]<br>c) Cognitive perspective taking (Flavell, Botkin, Fry, Wright, and Jarvis, 1968) [98, 99]{Blotner, 1984 #514}<br>d) Knowing tasks (Gonzales, Fabricius & Kupfer, 2017) [328]<br>e) Cow task (Luckett, Powell, Messer, Thornton, & Schulz, 2002) [136, 309]<br>f) Origin-of-belief task (O'Neill & Gopnik, 1991) [377-379]<br>g) See-know task (Pillow, 1989; Ruffman and Olson, 1989) [4, 11, 12, 20, 22, 23, 33, 34, 50, 51, 54, 57, 109-112, 114-127, 129, 131-146, 148, 149, 158-174, 176-186, 188-192, 215, 216, 221-224, 226, 227, 237, 238, 241, 242, 245, 246, 248, 249, 256, 258-260, 264, 266, 267, 302, 321, 324, 325, 362, 365, 366, 369, 371-375, 380-391]<br>h) Hidden sticker games (Povinelli and deBlois,1992) [361, 363, 364]<br>i) Inference tasks (Sodian & Wimmer, 1987) [377, 392]<br>j) Hide an object (Viranyi, Topal, Miklosi, & Csanyi 2006) [367]	a) -<br>b) -<br>c) +<br>d) -<br>e) -<br>f) -<br>g) +<br>h) -<br>i) -<br>j) +	Direct testing using read-aloud stories, pictures, audio recordings and/or figurines, or the child and experimenter/ parent/another child as protagonists	1 to 24	Correct/ incorrect or coding of children's reaction/ behavioral response/ gaze/verbal explanation	a) 60-132<br>b) 39-69<br>c) 36-132<br>d) 24-82<br>e) 37-101<br>f) 30-77<br>g) 18-168<br>h) 35-80<br>i) 42-80<br>j) 26-35	Bahasa Indonesia [109]<br>Cantonese [361, 362]<br>Chinese [111, 112]<br>Bislama [110]<br>Dutch [11, 12]<br>English sign language [363]<br>Farsi[114-116]<br>French [20, 22, 117, 118, 364]<br>German [23, 119-122, 215, 216, 365, 366]<br>Hungarian [367]<br>Indian [368]	ADHD [259]<br>ASD [22, 111, 133-140, 144, 221-224, 256, 258-260, 264, 266, 267, 309, 372-375]<br>Down syndrome[134, 141]<br>Fragile X syndrome [223]<br>Hearing impairment or deafness [112, 126, 127, 133, 142-144, 226]<br>Intellectual disability or developmental delay [126, 127, 373, 374]<br>Language impairment [119, 145]<br>Low birth weight [146]<br>Pregnancy hypertension [149]

	k) Visual access tasks (Wimmer, Hogrefe and Perner, 1988) [368, 370, 393, 394]	k) -				k) 36-72	Italian [33, 34]<br>Mandarin[123-125, 369]<br>Nakanamanga [110]<br>Oriya [370]<br>Polish [126, 127]<br>Portuguese [54]<br>Spanish [129, 371]<br>Turkish [132]	Prematurity [122]<br>Visual impairments or blindness [363]
Information-knowledge links: Understanding that someone who was not informed or is not familiar with something may not know	a) Cognitive perspective taking (Brice & Torney- Purta, 1981) [395]	a) +	Direct testing using a read-aloud story, pictures and/or, or using another child and/or the experimenter as protagonists	1 to 8	Correct/ incorrect	a) 24-48	Turkish [396]	ADHD [42]<br>Anxiety [42]<br>ASD [42]<br>Low SES [396]
	b) Opacity task (Kamawar & Olson, 2009) [397, 398]	b) -				b) 49-202		
	c) Modified ToM test (Kovacs, 2009) [399]	c) -				c) 34-42		
	d) Apple story (Example 2 from the TOM test; Muris, Steerneman, Meesters, Merckelbach, Horselenberg, van den Hogen & van Dongen, 1999)[42]	d) -				d) 60-144		
	e) Misinformation story (Perner et al., 1994) [396, 400]	e) -				e) 37-70		
	f) Awareness of a reader's knowledge task (Peskin, Prusky & Cromay, 2014) [401]	f) -				f) 60-96		
	g) Perspective evaluation task (Roberts & Patterson, 1983)	g) -				g) 46-75		
	h) Linguistic access task (Wimmer, Hogrefe & Perner, 1988) [394]	g) -				g) 38-70		
Knowledge-attention links: Understanding that something new is more interesting to someone than something already known	a) Familiary-focus of attention (Moll, Koring, Carpenter & Tomasello, 2006) [402]	a) +	Direct testing using child and experimenter/ parent as protagonists	1 to 6	Coding of children's reaction /behavior/ eye gaze	a) 17-24	German [402]<br>French [403]	
	b) Knowledge task (Moll & Tomasello, 2007) [403, 404]	a) +				a) 17-20		

Note. **Measures:** when authors provided no name for their measure, it was named according to its content in order to facilitate identification within the tables. The original source of a measure, written in brackets, may have not been included in the review and is provided to facilitate identification of measures. For a single article, there may be several variations of the same measure (e.g., different tasks using a classic change-in-location paradigm). **Availability of psychometric information**: + = information provided; – = No information provided (see Table X). **Languages**: Languages other than English in which the measure was administered, as reported in the studies. When the study specified no language of administration, nor language spoken by participants, it was assumed that it was administered in the language the article was written in. **Adverse conditions:** adverse clinical, psychological or environmental conditions. Children presenting adverse conditions may have different age ranges than the ones in TD population provided in the table.

Abbreviations: TD = Typically developing; SES = Socioeconomic Status; ASD = Autism Spectrum Disorders.

Table f.

Measures related to TOM- Beliefs category

	Measures characteristics					Participants characteristics		
**TOM abilities**	**Measures (source author, year) [articles using this measure]**	**Availability of psychometric information**	**Modes of presentation**	**Number of items**	**Scoring options**	**Age range in TD population (months)**	**Languages**	**Adverse conditions**
Content false beliefs: Familiar container with an unexpected content: Understanding the false belief held by someone who never opened the container	a) Deceptive contents FB task (Bartsch & Wellman, 1989) [1-3, 6, 63, 73, 74, 78, 80-83, 88, 89, 92, 103, 105, 262, 314, 315, 330, 405-416]<br>b) Deception task (Guajardo & Watson, 2002) [1, 408, 416, 461, 513]<br>c) Ella the Elephant or Emotion false belief task (Harris, Johnson, Hutton, Andrews, & Cooke, 1989) [2-4, 6, 23, 67, 68, 73, 74, 82, 85, 103, 104, 106, 110, 115, 125, 129, 130, 134, 148, 149, 153, 158, 175, 177-179, 182-187, 191, 239, 240, 245, 247, 251, 262, 329, 387, 411, 412, 417, 418, 434, 449, 451, 455, 479, 492, 493, 505, 514-528]<br>d) Content false belief paradigm (Hogrefe, Wimmer, & Perner, 1986; Perner, Leekam, & Wimmer, 1987) [9, 13, 15, 16, 21, 26, 42, 45, 48, 66-68, 72, 77, 79, 84, 85, 102, 104, 106, 135, 152, 157, 164, 175, 198, 209, 220, 228, 233, 239, 242, 243, 247, 253-255, 264, 266, 267, 281-284, 292, 293, 304, 306, 308, 310, 318, 320, 332, 335, 341, 347, 363, 368, 372, 377, 380-382, 386-388, 393, 396, 406, 413, 414, 417-422, 424-428, 430-441, 445-448, 451-454, 456-463, 465, 467, 469-488, 492-494, 496, 497, 499-504, 506-511, 513, 517, 521, 524, 529-611][4, 8, 17, 20, 23, 39, 42, 50, 51, 54, 57, 60, 109-112, 115-127, 129-146, 148, 149, 158-174, 176-183, 185-193, 201, 215, 217, 219, 221, 223, 226, 237, 238, 240, 244-249, 251, 287, 302, 324, 359, 362, 365, 366, 375, 385-387, 389, 390, 416, 424, 429, 442-444, 450, 464, 468, 489-491, 498, 505, 525, 528, 543, 550, 578, 612-619]	a) +<br>b) -<br>c) +<br>d) +	Direct testing using figurines, pictures, audio recordings or another child or a video or the experimenter and the child as protagonists	1 to 6	Correct/ incorrect or coding system for children's explanations	a) 32-89<br>b) 33-71<br>c) 35-168<br>d) 12-210	Bahasa Indonesia [109]<br>Basque [417, 418]<br>Bislama [110]<br>Brazilian Portuguese [201]<br>Cantonese [362, 419-421]<br>Chinese [8, 111, 112, 422]<br>Dutch [42, 423, 424]<br>English sign language [363, 425-428]<br>Farsi [115, 116]<br>Filipino [429]<br>French [13, 15-17, 19-21, 117, 118, 209, 262, 304, 308, 411, 430-437]<br>German [23, 119-122, 215, 217, 253, 365, 366, 423, 438-444]<br>Greek [445-447]	ADHD [42, 470, 471]<br>Anxiety [42]<br>Anxiety or depression [42]<br>ASD [26, 42, 67, 68, 111, 133-140, 144, 221, 223, 264, 266, 267, 283, 308, 310, 372, 375, 432, 436, 449, 450, 453, 456, 471-491]<br>Attention and behavior problems [492]<br>Developmental Coordination Disorder [67, 493]<br>Developmental disabilities of mixed etiologies [42, 484]<br>Down Syndrome [134, 141, 453, 482]<br>Externalizing behavior problems [13, 415]<br>Fragile X syndrome [223]<br>Hearing impairment or deafness [112, 126, 127, 133, 142-144, 219, 226, 363, 425-428, 494-496]<br>Intellectual disability or developmental delay

							Hebrew [63, 448-450]<br><br>Indian [368]<br><br>Italian [26, 103-106, 451-454]<br><br>Japanese [455]<br><br>Korean [456-458]<br><br>Mandarin [66, 123-125, 219, 459-461]<br><br>Nakanamanga [110]<br><br>Norwegian [306]<br><br>Polish [126, 127]<br><br>Portuguese [54]<br><br>Spanish [26, 39, 129, 130, 292, 417, 418, 462-464]<br><br>Turkish [131, 132, 420, 465-468]<br><br>Ulithian [469]<br><br>Yapese [469]	[13, 19, 45, 126, 127, 436, 480, 484, 487]<br><br>Language impairment [67, 119, 145, 292, 436, 462, 463, 493, 497, 498]<br><br>Learning difficulties [483, 486]<br><br>Low birth weight [146]<br><br>Low SES [153, 314, 315, 396, 409, 413, 488, 499-507]<br><br>Maltreatment [148, 504]<br><br>Prader-Willi syndrome [45, 508]<br><br>Pregnancy hypertension [149]<br><br>Prematurity [122, 509]<br><br>Reading disorder [436]<br><br>TBI [510]<br><br>Visual impairment or blindness [318, 423, 511, 512]<br><br>Williams syndrome [15, 16, 45, 304, 347, 508]
Location false beliefs: Unseen change: Understanding the false belief held by someone who did not witness or was not informed of a displacement or change of action	a) Sandbox task (Begeer, Bernstein, van Wijhe, Scheeren & Koot, 2012) [612]<br><br>b) Change-of location own false-belief (Buttelmann et al., 2016) [605]<br><br>c) Changing appearance tasks (Leekam & Perner, 1991) [434, 659]<br><br>d) Message-desire discrepancy (Mitchell, Saltmarsh & Russell, 1997) [20, 257-260]<br><br>e) Change in location and deceive (Murray, Woolgar et al., 1999) [671]<br><br>f) Social activity false belief task (Nguyen & Frye, 1999) [231, 263, 370, 588, 610, 666]	a) -<br><br>b) -<br><br>c) -<br><br>d) -<br><br>e) -<br><br>f) -	Direct testing using a narrated story, figurines, pictures, computerized games, CD-ROM, videos, live actors and/or the child and experimenter as protagonists	1 to 32	Correct/ incorrect or coding of children's explanations, facial expressions or eye gaze, or eyetracking, or measuring distances between child's answer and correct	a) 36- 90<br><br>b) 33-52<br><br>c) 41-99<br><br>d) 24-108<br><br>e) *M* = 60<br><br>f) 34-164	Basque [200, 417, 418]<br><br>Bislama [110]<br><br>Cameroonian [303, 620]<br><br>Cantonese [282, 419-421, 621]<br>Chinese [8, 422, 594, 622]	ADHD [42, 259, 471]<br><br>Anxiety or depression [42]<br><br>Attention and behavior problems [492]<br><br>ASD [22, 26, 42, 43, 68, 135-137, 197, 220, 221, 224, 256-260, 264, 266, 267, 283, 290, 308, 309, 432, 436, 453, 456, 471-479, 481-483, 485,

g) Change-in-location paradigm (Wimmer & Perner, 1983)/Sally-Ann task, (Baron-Cohen, Leslie & Frith, 1985) [1, 15, 16, 29, 45, 66, 72, 76, 77, 79, 84, 86, 106, 112-115, 128, 150-152, 177, 200, 217, 231, 239, 242, 243, 253, 263, 264, 275, 303, 304, 308, 315, 317, 318, 320, 329, 332, 336, 341, 363, 370, 372, 381, 387, 388, 393, 396, 399, 405, 406, 408, 409, 411-413, 420-425, 427, 428, 431, 433, 434, 437, 439, 440, 445-449, 453, 458, 460, 461, 465, 477, 481, 483, 486, 488, 492, 493, 499-503, 507, 510, 512, 513, 516, 520-522, 525, 529-534, 536, 540, 542, 543, 547, 551, 556, 560, 561, 565, 566, 568, 569, 572-574, 580, 581, 583, 585, 587, 590, 592, 594, 599-601, 620-622, 625, 629-635, 642, 645, 649, 650, 656, 660-663, 666-668, 670, 672-709][8, 13, 21, 26, 30, 39, 42, 43, 48, 59, 60, 63, 67, 68, 93, 103, 105, 114, 135-137, 143, 148, 159, 163, 175, 187, 196-198, 203, 211, 214, 217, 220, 221, 224, 226, 229, 237, 247, 266, 267, 281-283, 290, 292, 293, 309, 314, 354, 365, 366, 368, 371, 374, 377, 382, 400, 412, 414, 416-419, 426, 429, 430, 432, 436, 454, 456, 459, 467, 471, 472, 474-476, 478-480, 482, 485, 494, 497, 505, 509, 517, 524, 537-539, 546, 551, 552, 554, 557, 559, 562, 570, 571, 575, 578, 584, 587, 588, 598, 600, 604, 605, 607, 609, 610, 612, 623-625, 638-641, 643, 646, 647, 650, 651, 653-655, 657, 658, 662, 664, 665, 682, 683, 690, 703, 710-732] [11, 12, 17, 19, 20, 22, 33, 34, 54, 110, 126, 193, 205, 213, 217, 219, 238, 240, 247, 256-260, 287, 294, 324, 385, 387, 389, 390, 403, 464, 491, 550, 614, 616, 617, 627, 628, 636, 637, 648, 725, 733-740]	g) +		response on a continuous scale	g) 13-1104	Dutch [11, 12, 42, 113, 203, 205, 217, 423, 623]<br>English sign language [143, 363, 425-428, 624]<br>Farsi [114, 115]<br>Filipino [429]<br>French [13, 15-17, 19-22, 211, 213, 304, 308, 403, 430-432, 434, 436, 437, 599, 625-628]<br>German [214, 217, 253, 303, 365, 366, 423, 439, 440, 620, 629-637]<br>Greek [445-447, 638]<br>Hebrew [63, 448, 449]<br>Hungarian [399]<br>Indian [368, 453]<br>Italian [22, 26, 29, 30, 33, 34, 103, 105, 106, 454, 639]<br>Japanese [640-643]<br>Korean [456, 458, 644]<br>Mandarin [66, 219, 460, 461]<br>Nakanamanga [110]<br>Oriya [370]<br>Polish [126, 263, 645]	486, 488, 491, 493, 532, 566, 570, 626, 627, 631, 640, 648-659]<br>Cerebral palsy [646, 660]<br>Developmental coordination disorder [67, 493]<br>Down syndrome [15, 16, 453, 482, 658, 660, 661]<br>Externalizing behavior problems [13]<br>Fetal alcohol spectrum disorders [662]<br>Hearing impairments or deafness [126, 143, 203, 205, 219, 226, 363, 425-428, 494, 531, 639, 647]<br>Intellectual disability or developmental delay [13, 15, 19, 43, 45, 126, 304, 436, 640, 651, 652, 663]<br>Language impairments [67, 197, 292, 436, 497, 623, 626, 664, 665]<br>Learning difficulties [483, 654]<br>Low SES [177, 314, 315, 413, 466, 488, 499-503, 505, 507, 666-669]<br>Malteatment [148]<br>Prader-Willi syndrome [45]<br>Prematurity [509]<br>Reading disorder [436]<br>Sensory integration deficits [303]<br>TBI [150, 510]

							Portuguese [54, 601]<br><br>Romanian [399]<br><br>Spanish [26, 39, 128, 292, 303, 371, 417, 418, 463, 464, 601]<br><br>Swedish [646-648]<br><br>Turkish [396, 420, 459, 465, 467, 601]	Visual impairments or blindness [318, 423, 512]<br><br>Williams syndrome [45, 650, 670]
Identity false beliefs: Understanding that when something looks/sounds/ smells like something else, a person may hold a false belief about its identity/chara-cteristics/location	a) Little Red Riding Hood (Bradmetz & Gauthier, 1999)[741]	a) -	Direct testing using pictures, storybooks, computerized animations, peep-through books and/or figurines	1 to 22	Correct/ incorrect or coding of children's explanations, pointing gestures or eye gazes	a) 36-83	Basque [417, 418]<br><br>Bislama [110]<br><br>Cantonese [742]<br><br>Chinese [422, 594]<br><br>Dutch [113, 424]<br><br>English sign language [427, 743]<br><br>French [15-17, 19, 20, 210, 212, 304, 411, 433-435, 437, 470, 625, 709]<br><br>German [439, 633, 637]<br><br>Greek [445-447]<br><br>Hebrew [448]<br><br>Indian [368]<br><br>Korean [457]<br><br>Nakanamanga [110]<br><br>Oriya [370]	ADHD [470]<br><br>ASD [470, 478, 570]<br><br>Cerebral Palsy [646]<br><br>Down syndrome [15, 16]<br><br>Hearing impairments or deafness [427, 531, 743, 745]<br><br>Intellectual disability or developmental delay [19, 304]<br><br>Language impairment [497]<br><br>Low SES [70, 153, 311-316, 396, 409, 413, 499, 504, 506, 507, 570]<br><br>Maltreatment [504, 570]<br><br>Prematurity [509]<br><br>TBI [210, 510]<br><br>William syndrome [670]
	b) Picture false-belief task (Callaghan, Rochat & Corbit, 2012) [604, 744]	b) +				b) 34-168		
	c) Ambiguous figures (Carpendale & Chandler, 1996)[229]	c) -				c) 58-107		
	d) Ambiguous referential communication (Carpendale & Chandler, 1996) [229]	d) -				d) 58-107		
	e) Lexical ambiguity (Carpendale & Chandler, 1996) [229, 281, 742, 746-748]	e) +				e) 49-135		
	f) Mother-infant separation test (MIST) (de Rosnay & Harris, 2002) [518, 527]	f) -				f) 43-76		
	g) Droodle task (Chandler & Helm, 1984; Hughes, Dunn & White 1998) [2, 3, 6, 73, 74, 82, 210, 212, 311-316, 330, 405, 412, 427, 433, 434, 437, 499, 504, 506, 575, 581, 583, 619, 735, 749]	g) +				g) 24-138		
	h) Appearance-reality tasks (Flavell, 1986) [15-17, 19, 20, 70, 110, 153, 232, 233, 239, 242, 304, 319, 320, 336, 337, 368, 380-382, 388, 393, 406, 409, 411, 417, 418, 422, 424, 427, 435, 439, 445-448, 457, 464, 465, 470, 471, 478, 497, 507, 510, 513, 531, 533, 539, 542, 545, 562, 564, 565, 568, 570, 573, 574, 578, 583, 584, 589, 594, 596, 612, 633, 637, 674, 709, 729, 734, 750-752]	h) +				h) 25-189		
	i) The deception stories (Johnson, 1997) [591]	i) -				i) 48-108		
	j) False belief story (Riggio & Cassidy 2009) [753]	j) +				j) *M* = 51.87		
	k) Deception task (Ruffman, Olson, Ash, and Keenan, 1993)[396, 611]	k) -				k) 43-70		
	l) Misinformation tests (San Juan & Astington, 2017) [729]	l) -				l) 30-53		

	m) Ambiguity task (Taylor, 1988) [77, 87, 113, 302, 382, 393, 401, 413, 417, 418, 556, 596, 703, 710, 754-758]	m) +				m) 34-138	Samoan [744]<br>Spanish [417, 418, 464]<br>Swedish [646]<br>Turkish [465, 466]	
	n) Appareance-reality tasks (Taylor and Hort, 1990) [335, 759]	n) -				n) 31-65		
	o) Understanding of pretense (Watson & Guajardo, 2000) [760]	o) -				o) 50-76		
	p) Thought pictures/Balloon task (Wellman, Hollander & Schult,1996) [509, 531, 551, 587, 646, 670, 743, 745, 761]	p) -				p) 34-117		
Second-order belief: understanding the second-order belief or false belief held by someone who doe not know somebody else was informed (e.g., of a misleading identity, a misleading location, etc.)	a) Mean looking dog (Bradmetz & Gauthier, 2005) [625]	a) -	Direct testing using figurines, pictures, audio recordings, videos or CD-ROM	1 to 4	Correct/ incorrect, coding systems of children's explanation	a) 60-107	Basque [417, 418]<br>Cantonese [282]<br>Cameroonian [620]<br>Dutch [42]<br>English sign language [426, 428, 624]<br>French [20, 433, 434, 625]<br>German [272, 365, 441, 620]<br>Hebrew [448]<br>Italian [103-106, 451, 639]<br>Japanese [455, 642]<br>Polish [762]<br>Spanish [129, 130, 417, 418]<br>Swedish [647]	ADHD [42, 259]<br>Anxiety or depression [42]<br>ASD [42, 67, 68, 256-260, 264, 266, 474-477, 488-491, 505]<br>Developmental coordination disorder [67]<br>Hearing impairments or deafness [426, 428, 494, 624, 639, 647]<br>Language impairments [67]<br>Low SES [413, 505, 667, 763]<br>Maltreatment [148]
	b) ToM task (Kim & Phillips, 2014) [763]	b) +				b) 60-97		
	c) Second order false belief task (Miller, 2013a) [764]	c) -				c) 63-92		
	d) Second order false belief with deception (Miller, 2013b) [764]	d) -				d) 64-81		
	e) Ice-cream van test (Perner & Wimmer, 1985) [42, 67, 68, 79, 224, 272, 413, 416, 426, 428, 433, 434, 441, 474-477, 488-491, 505, 516, 520, 531, 537, 579, 612, 614, 617, 618, 625, 639, 667, 711, 731, 732, 765-768]	e) +				e) 36-174		
	f) Birthday puppy (Sullivan, Zaitchik, & Tager- Flusberg, 1994) [53, 103-105, 129, 154, 264, 266, 282, 365, 412, 416, 451, 455, 579, 618, 620, 766, 769]	f) +				f) 47-148		
	g) Granddad story, Window story or Tom's crayon (Sullivan, Zaitchik, & Tager-Flusberg, 1994; Astington, Pelletier & Homer, 2002) [2, 20, 85, 106, 129, 130, 148, 152, 163, 256-260, 282, 320, 401, 412, 417, 418, 448, 505, 521, 524, 544, 609, 616, 617, 624, 625, 642, 647, 717, 731, 748, 762, 766, 767, 770-775]	g) +				g) 24-156		
Beliefs-based actions/ emotions: Predicting another emotions or actions based on their stated beliefs /Inferring	a) Anomalous-belief stories (Colonnesi, Rieffe, Koops & Perucchini, 2008)[273]	a) -				a) 38-40	Africaans [267]<br>Bahasa Indonesia [109]	ADHD [777, 778]<br>ASD [22, 26, 67, 68, 111, 133-140, 144, 221-
	b) Misunderstanding stories from the Strange stories (Happé, 1994)[8, 67, 68, 267, 493, 777-782]	b) -				b) 36-192		

another person’s belief based on their stated action or emotion	c) Predicting actions on the basis of a person’s knowledge (Howlin, Baron-Cohen & Hadwin, 1999) [324, 390]<br>d) False belief-based emotion (Pons & Harris, 2000, Pons, Harris & Rosnay, 2004) [22, 26-30, 32, 49, 51-53, 187]<br>e) The Tom task (Swettenham, 1996) [439, 482]<br>f) False belief justification (Wellman, 1991) [599, 776]<br>g) Belief tasks/Diverse beliefs (Wellman & Bartsch, 1988) [4, 11, 12, 22, 23, 31, 33, 34, 50, 51, 54, 57, 64, 70, 71, 109-112, 114-127, 129-146, 148, 149, 158-166, 168-174, 176-186, 188-192, 201, 215, 216, 221-224, 226, 227, 237-242, 244-246, 248, 249, 265, 270, 385, 386, 389, 407, 424, 442, 444, 489, 490, 531, 539, 550, 551, 553, 573, 594, 614, 653, 783]<br>h) Think stories (Wellman & Bartsch, 1988) [270]	c) -<br>d) -<br>e) -<br>f) -<br>g) +<br>h) -				c) 57-70<br>d) 36-132<br>e) 39-46<br>f) 36-77<br>g) 12-156<br>h) 50-348	Bislama [110]<br>Brazilian Portuguese [201]<br>Chinese [8, 111, 112, 594]<br>Dutch [11, 12]<br>Farsi [114-116]<br>French [22, 117, 599, 776]<br>German [23, 119-122, 215, 216, 439, 442, 444]<br>Italian [22, 26-30, 33, 34, 64]<br>Japanese [31]<br>Mandarin[123-125]<br>Nakanamanga [110]<br>Norwegian [32]<br>Polish [126, 127]<br>Portuguese [54]<br>Spanish [26, 129, 130]<br>Turkish [131, 132]	224, 265, 267, 482, 489, 490, 653, 777-779]<br>Developmental coordination disorder [67, 493]<br>Down Syndrome [134, 141, 482]<br>Fragile X syndrome [223]<br>Hearing impairments or deafness [112, 126, 127, 133, 142-144, 226, 531]<br>Intellectual disability or developmental delay [126, 127, 777]<br>Language impairment [67, 119, 145, 493]<br>Low birth weight [146]<br>Low SES [70, 71]<br>Maltreatment [70, 148]<br>Pregnancy hypertension [149]<br>Prematurity [122]<br>Reading disorder [778]
Sequence false beliefs: Understanding the false belief created when a predictable sequence of stimuli is broken with the intrusion of an unexpected stimulus	a) Unexpected outcome (Newly learned & Previously learned) (Brambring & Asbrock, 2010) [423]	a) -	Direct testing using audiotracks	6	Correct/ incorrect	a) 42-72	Dutch [423]<br>German [423]	Visual impairments or blindess [423]
	a) False-belief explanation task (de Villiers & de Villiers, 2000) [30, 454, 496]	a) +	Direct testing using figurines,	2 to 12	Correct/	a) 46-75	French [17, 19, 20]	ASD [489]

Comprehensive measures of understanding beliefs	b) Battery of TOM tasks (Hughes, Adlam, Happe, Jackson, Taylor & Caspi, 2000) [54, 103, 105, 106, 451, 505, 585, 785]	b) +	pictures or videos	incorrect, coding of child's explana-tion, coding of use of mental state terms, or coding of children's facial expression	b) 43-108	Italian [30, 103, 105, 106, 451, 454]	Externalizing behavior problems [785]
	c) False-belief suspense (Moll, Kane & McGowan, 2016) [725]	c) +			c) 36-42		Hearing impairments or deafness [496]
	d) TOM belief tasks (Nader-Grosbois & Thirion-Marissiaux, 2011) [17, 19, 20]	d) -			d) 39-83		Intellectual disability or developmental delay [19]
	e) Birthday party story and school cricket team story (Raisa, Karuppali, Bhat & Bajaj, 2019) [784]	e) -			e) 36-107	Kannada [784]	
	f) Theory-of-mind tasks protocol (Sparrevohn & Howie, 1995) [489, 490, 550, 614]	f) -			f) 32-72		

Note. **Measures:** when authors provided no name for their measure, it was named according to its content in order to facilitate identification within the tables. The original source of a measure, written in brackets, may have not been included in the review and is provided to facilitate identification of measures. For a single article, there may be several variations of the same measure (e.g., different tasks using a classic change-in-location paradigm). **Availability of psychometric information**: + = information provided; – = No information provided (see Table X). **Age range in TD population**: "*M*" signifies the mean age of the sample and is presented only when no age range could be retrieved. **Languages**: Languages other than English in which the measure was administered, as reported in the studies. When the study specified no language of administration, nor language spoken by participants, it was assumed that it was administered in the language the article was written in. **Adverse conditions:** adverse clinical, psychological or environmental conditions. Children presenting adverse conditions may have different age ranges than the ones in TD population provided in the table.

Abbreviations: TD = Typically developing; SES = Socioeconomic Status; ASD = Autism Spectrum Disorders; ADHD = Attention-deficit/hyperactivity disorder; TBI = Traumatic brain injury

Table g.

Measures related to TOM- Mentalistic understanding of non-literal communication category

	Measures characteristics					Participants characteristics		
**TOM abilities**	**Measures (source author, year) [articles using this measure]**	**Availability of psychometric information**	**Modes of presentation**	**Number of items**	**Scoring options**	**Age range in TD population (months)**	**Languages**	**Adverse conditions**
Irony/sarcasm: Understanding that other people may lie in order to be ironic/sarcastic	a) Irony task (Filippova & Astington, 2008) [624, 774]<br>b) Sarcasm stories from the Strange stories (Happé, 1994) [8, 67, 68, 114, 122, 144, 267, 292, 493, 624, 777, 778, 780-782]<br>c) Party food story (Langdon & Coltheart, 2004) [624]<br>d) Party story (Example 6 from the TOM test; Muris, Steerneman, Meesters, Merckelbach, Horselenberg, van den Hogen & van Dongen, 1999) [42]<br>e) Rain story (Examle 9 from the TOM test; Muris, Steerneman, Meesters, Merckelbach, Horselenberg, van den Hogen & van Dongen, 1999)[42]<br>f) Lies and jokes task (Tager-Flusberg & Sullivan, 1995) [264, 786]	a) +<br>b) +<br>c) -<br>d) -<br>e) -<br>f) -	Direct testing using pictures or read-aloud stories	1 to 8	Correct/ incorrect or coding of children explanations or likert scales	a) 61-121<br>b) 38-192<br>c) 72-121<br>d) 60-144<br>e) 60-144<br>f) None	Africaans [267]<br>Chinese [8]<br>Farsi [114]<br>German [122]<br>Signed English [624]<br>Spanish [292]	ADHD [42, 777, 778]<br>Anxiety [42]<br>ASD [42, 67, 68, 144, 264, 267, 777, 778, 786]<br>Developmental coordination disorder [67, 493]<br>Hearing impairment [144, 624]<br>Intellectual disability or developmental delay [777]<br>Language impairment [67, 292, 493]<br>Prematurity[122]<br>Reading disorders [778]
Egocentric lies: Understanding that someone may consciously lie in order to avoid a problem or to get its way	a) Contrary emotions stories from the Strange stories (Happé, 1994)[8, 67, 68, 267, 493, 777-782]<br>b) Double-bluff stories from the Strange stories (Happé, 1994)[8, 67, 68, 267, 493, 777, 778, 780-782]<br>c) Lie stories from the Strange stories (Happé, 1994) [8, 67, 68, 267, 292, 493, 721, 777-782]<br>d) Persuade stories from the Strange stories (Happé, 1994) [8, 67, 68, 267, 493, 777, 778, 780-782]	a) -<br>b) -<br>c) -<br>d) -	Direct testing using read-aloud stories with pictures	1-2	Correct/ incorrect or coding of children explanations	a) 36-192<br>b) 36-192<br>c) 36-192<br>d) 36-192	Africaans [267]<br>Chinese [8]<br>Spanish [292]	ADHD [777, 778]<br>ASD [67, 68, 267, 777-779]<br>Developmental coordination disorder [67, 493]<br>Intellectual disability or development delay [777]<br>Language impairment [67, 292, 493]<br>Reading disorders [778]

White lies: Understanding that someone may lie in order to spare another's feelings	a) White lie stories from the Strange stories (Happé, 1994) [8, 67, 68, 267, 292, 493, 721, 777-782, 787]	a) +	Direct testing using read-aloud stories with pictures	1-2	Correct/ incorrect or coding of children explanations	a) 36-192	Africaans [267]<br><br>Chinese [8]<br><br>Spanish [292]	ADHD [777, 778]<br><br>ASD [67, 68, 267, 777-779]<br><br>Developmental coordination disorder [67, 493]<br><br>Intellectual disability or development delay [777]<br><br>Language impairment [67, 292, 493]<br><br>Reading disorders [778]
Involontary lies: Understanding that someone may tell a "lie" without knowing	a) Forget stories from the Strange stories (Happé, 1994)[8, 67, 68, 267, 493, 721, 777-782]	a) -	Direct testing using read-aloud stories with pictures	1-2	Correct/ incorrect or coding of children explanations	a) 36-192	Africaans [267]<br><br>Chinese [8]	ADHD [777, 778]<br><br>ASD [67, 68, 267, 777-779]<br><br>Developmental coordination disorder [67, 493]<br><br>Intellectual disability or development delay [777]<br><br>Language disorder [67, 493]<br><br>Reading disorders [778]
Humor: understanding that someone may tell a "lie" in order to make a joke	a) Joke stories from the Strange stories (Happé, 1994)[8, 67, 68, 267, 493, 777, 778, 780-782, 787]	a) +	Direct testing using read-aloud stories with pictures	1-2	Correct/ incorrect or coding of children explanations	a) 36-192	Africaans [267]<br><br>Chinese [8]	ADHD [777, 778]<br><br>ASD [67, 68, 267, 777, 778]<br><br>Developmental coordination disorder [67, 493]<br><br>Intellectual disability or development delay [777]<br><br>Language disorder [67, 493]<br><br>Reading disorders [778]
Faux pas: Ability to recognize *faux-pas* (social gaffe) situations	a) Recognition of faux pas (Baron-Cohen, O'Riordan, Stone, Jones, & Plaisted, 1999) [119, 126, 267, 401, 774]	a) +	Direct testing using audio stories or figurines	2 to 10	Correct/ incorrect	a) 48-192	German [119]<br><br>Polish [126, 127]	ASD [267]<br><br>Hearing impairment or deafness [126, 127]

								Intellectual disability of developmental delay [126, 127]<br><br>Language impairments [119]
Measures tapping multiple aspects of mentalistic understanding of non-literal communication	a) Ironic criticism and empathic praise task (Dennis et al., 2001) [788]<br>b) Strange stories (Happé, 1994) [8, 67, 68, 114, 138, 144, 267, 292, 493, 624, 721, 774, 777-782, 787, 790, 791]	a) -<br>b) +	Direct testing using read-aloud stories with pictures	1 to 12	Correct/ incorrect or coding of children explanations	a) 63-184<br>b) 36-192	Chinese [8]<br><br>English Sign Language [144, 624]<br><br>Farsi [114]	ADHD [777, 778]<br><br>ASD [68, 138, 144, 267, 778, 779, 789, 790]<br><br>Developmental coordination disorder [67, 493]<br><br>Hearing impairments or deafness [144, 624]<br><br>Language impairments [67, 292, 493, 779]<br><br>Reading disorder [778, 789]<br><br>TBI [788]

Note. **Measures:** when authors provided no name for their measure, it was named according to its content in order to facilitate identification within the tables. The original source of a measure, written in brackets, may have not been included in the review and is provided to facilitate identification of measures. For a single article, there may be several variations of the same measure (e.g., different tasks using a classic change-in-location paradigm). **Availability of psychometric information**: + = information provided; – = No information provided (see Table X). **Age range in TD population**: “None” signifies the absence of TD children in the studies using the measure. **Languages**: Languages other than English in which the measure was administered, as reported in the studies. When the study specified no language of administration, nor language spoken by participants, it was assumed that it was administered in the language the article was written in. **Adverse conditions:** adverse clinical, psychological or environmental conditions. Children presenting adverse conditions may have different age ranges than the ones in TD population provided in the table.

Abbreviations: TD = Typically developing; ASD = Autism Spectrum Disorders; ADHD = Attention-deficit/hyperactivity disorder; TBI = Traumatic brain injury

Table h.

Comprehensive measures related to TOM

	Measures characteristics					Participants characteristics		
**TOM abilities**	**Measures (source author, year) [articles using this measure]**	**Availability of psychometric information**	**Modes of presentation**	**Number of items**	**Scoring options**	**Age range in TD population (months)**	**Languages**	**Adverse conditions**
Multiple TOM abilities measured using que[127]stionnaires/interviews	a) Supplementary social and maladaptive items/*Échelle d'adaptation sociale pour enfants* (Frith, Happé & Sidons, 1994) [17, 20, 117, 308, 436, 483]<br>b) Theory of mind inventory & Perceptions of children's theory of mind measure-experimental version (Hutchins, Bonazinga, et al., 2008; Hutchins, Prelock & Bonazinga, 2012) [17, 20, 257-259, 792-796]<br>c) Everyday mindreading skills and difficulties scale (Peterson, Garnett, Kelly & Attwood, 2009) [478]<br>d) Children's social understanding scale (Tahiroglu, Moses, Carlson, Mahy, Olofson & Sabbagh, 2014) [126, 127, 302, 619]	a) +<br>b) +<br>c) +<br>d) +	Questionnaires completed by parents and/or other adults	8 to 85	Likert scale or response continuum of 20 metric units	a) 36-74<br>b) 24-168<br>c) *M* = 117<br>d) 28-144	French [17, 20, 117, 308, 436]<br>Polish [126, 127]<br>Spanish [792]	ADHD [259]<br>ASD [257-259, 302, 308, 436, 478, 483, 792-794]<br>Hearing impairment or deafness [126, 127]<br>Intellectual disability or developmental delay [126, 127, 436]<br>Language impairments [436]<br>Learning disabilities [483]<br>Reading disorder [436]
Multiple TOM abilities measured using direct testing	a) ToM storybooks (Blijd-Hoogewys et al., 2008) [11, 12, 22, 33, 34, 618, 797, 798]<br>b) Five movies for assessing the use of mental words and the aqcuisition of a TOM (Bouchand & Caron, (1999) [809]<br>c) Psychological explanation task (Colonnesi, Rieffe, Koops & Perucchini, 2008) [273]<br>d) Comic strip Task (Cornish et al. 2010) [813, 817]<br>e) Perspective taking task (Edelstein, Keller & Wahlen; 1984) [96]<br>f) Perspective taking ability test (Hudson, Forman, and Brion, 1982; Shin, 1996) [107]<br>g) TOM task battery (Hutchins, Prelock, Chace, 2008) [20, 256-260]	a) +<br>b) -<br>c) -<br>d) +<br>e) -<br>f) -<br>g) -	Direct testing using web vignettes, pictures, read aloud stories, audio recordings, storybooks, figurines and/or games	1 to 110	Correct/ incorrect or likert scales or coding children's explanations	a) 31-148<br>b) 36-78<br>c) 38-40<br>d) 48-147<br>e) *M*= 70- *M*= 106<br>f) 60-71<br>g) 36-165	Auslan [112]<br>Bahasa Indonesia [109]<br>Cantonese [362]<br>Chinese [111]<br>Dutch [11, 12, 42, 799-804]<br>English sign language [112, 143, 144]<br>Farsi [114-116]	ADHD [42, 259, 778, 781]<br>Anxiety or depression [42, 781]<br>ASD [11, 22, 41-43, 111, 133-139, 144, 221-224, 256-260, 375, 778, 781, 806, 808, 810, 812-818]<br>Down syndrome [134, 141, 223]<br>Fragile x syndrome [223]

h) Theory of mind subtest of a developmental neuropsychological assessment (NEPSY-II; Korkman, Kirk & Kamp, 2007) [41, 674, 778, 781, 805-808, 810, 812, 815, 821]	h) +	h) 34-191	Filipino [429]<br><br>Finnish [805] [806, 807]<br><br>French [20, 117, 118, 808, 809]<br><br>German [120, 121, 215, 217, 442, 443] [23, 119, 122, 365, 366, 444]<br><br>Italian [22, 28, 33, 34, 65, 273, 797, 807, 810]<br><br>Mandarin [123-125, 219]<br><br>Polish [126, 127]<br><br>Portuguese [201]<br><br>Spanish [129, 130]<br><br>Swedish [647]<br><br>Turkish [131, 132, 811] [468]	Hearing impairments or deafness [112, 126, 127, 133, 142-144, 219, 226, 647, 819]<br><br>Intellectual disability or developmental delay [43, 126, 127, 814]<br><br>Language impairments [119, 145, 498, 806]<br><br>Learning difficulties [781]<br><br>Low birth weight [146]<br><br>Low SES [109, 177, 178]<br><br>Maltreatment [148]<br><br>Neurofibromato-sis [820]<br><br>Prader-Willi syndrome [45]<br><br>Pregnancy hypertension [149]<br><br>Prematurity [122]<br><br>Reading disorder [778]<br><br>Turner syndrome [821]<br><br>Visual impairment or blindness [797]<br><br>Williams syndrome [45, 822]
i) Perspective-taking (Krcmar & Vieira, 2005) [823]	i) -	i) 60-144		
j) Perspective-taking tests (Kurdek & Rodgon,1975) [811]	j) -	j) 60-72		
k) Past to future reasoning task (Lagattuta & Sayfan, 2011) [824, 825]	k) -	k) 48- 300		
l) Picture sequencing task (Langdon et al., 1997) [820, 822]	l) -	l) 48-144		
m) Pragma test (Loukusa et al., 2018)	m) +	m) 62-120		
m) Social meaning scale from the SELweb (McKown, Russo-Ponsaran, Johnson, Russo & Allen, 2016) [791]	m) -	m) *M* = 87.6-91.2		
n) TOM test (Muris, Steerneman, Meesters, Merckelbach, Horselenberg, van den Hogen & van Dongen, 1999) [42, 799-804]	n) -	n) 48-156		
o) Perspective-taking tasks (Oppenheimer & Thijssen, 1983) {Oppenheimer, 1983 #500}{Oppenheimer, 1983 #511}	o) +	o) 59-156		
p) Theory of mind test (Pons & Harris, 2002) [28]	p) -	p) *M* = 53		
q) Deaf children series (Serra, Spinato, Cocuzza et al. 2017) [819]	q) -	q) 54-74		
r) Socio-emotional ToM test (Sundqvist, Lyxell, Jonsson, Heimann, 2014) [647]	r) -	r) 24-152		
s) Explanation of Action task (Tager-Flusberg and Sullivan, 1994) [43, 45]	s) -	s) 39-123		
t) ToM scale (Wellman and Liu, 2004) [4, 23, 50, 51, 54, 57, 65, 109, 111, 112, 114-125, 127, 129-146, 148, 149, 158-184, 186, 188, 190-192, 201, 215-217, 219, 221-224, 226, 227, 237-249, 251, 287, 302, 362, 365, 366, 375, 386-389, 424, 429, 442-444, 468, 498, 528, 539, 578, 619, 733, 826-829]	t) +	t) 14-156		

Note. –: no information provided in the articles. **Measures:** when authors provided no name for their measure, it was named according to its content in order to facilitate identification within the tables. The original source of a measure, written in brackets, may have not been included in the review and is provided to facilitate identification of measures. For a single article, there may be several variations of the same measure (e.g., different tasks using a classic change-in-location paradigm). **Availability of psychometric information**: + = information provided; – = No information provided (see Table X). **Age range in TD population**: "*M*" signifies the mean age of the sample and is presented only when no age range could be retrieved. **Languages**: Languages other than English in which the measure was administered, as reported in the studies. When the study specified no language of administration, nor language spoken by participants, it was assumed that it was administered in the language the article was written in. **Adverse conditions:** adverse clinical, psychological or environmental conditions. Children presenting adverse conditions may have different age ranges than the ones in TD population provided in the table.

Abbreviations: TD = Typically developing; SES = Socioeconomic status; ASD = Autism spectrum disorders; ADHD = Attention-deficit/hyperactivity disorder.

1. Weimer, A.A. and N.R. Guajardo, *False Belief, Emotion Understanding, and Social Skills Among Head Start and Non-Head Start Children.* Early Education and Development, 2005. **16**(3): p. 341-366.
2. Cutting, A.L. and J. Dunn, *The cost of understanding other people: social cognition predicts young children's sensitivity to criticism.* Journal of Child Psychology & Psychiatry & Allied Disciplines, 2002. **43**(7): p. 849-60.
3. Dunn, J., A.L. Cutting, and H. Demetriou, *Moral sensibility, understanding others, and children's friendship interactions in the preschool period.* British Journal of Developmental Psychology, 2000. **18**(2): p. 159-177.
4. Guajardo, N.R., G. Snyder, and R. Petersen, *Relationships among parenting practices, parental stress, child behaviour, and children's social-cognitive development.* Infant and Child Development, 2009. **18**(1): p. 37-60.
5. Shields, A., et al., *Emotional competence and early school adjustment: A study of preschoolers at risk.* Early Education and Development, 2001. **12**(1): p. 73-96.
6. Cutting, A.L. and J. Dunn, *Conversations with siblings and with friends: Links between relationship quality and social understanding.* British Journal of Developmental Psychology, 2006. **24**(1): p. 73-87.
7. Cassidy, J., et al., *Family-Peer Connections: The Roles of Emotional Expressiveness within the Family and Children's Understanding of Emotions.* Child Development, 1992. **63**(3): p. 603-618.
8. Liu, B., et al., *Altruistic sharing behavior in children: Role of theory of mind and inhibitory control.* Journal of Experimental Child Psychology, 2016. **141**: p. 222-8.
9. Colonnesi, C., I.M. Engelhard, and S.M. Bogels, *Development in children's attribution of embarrassment and the relationship with theory of mind and shyness.* Cognition and Emotion, 2010. **24**(3): p. 514-521.
10. Wiefferink, C.H., et al., *Emotion Understanding in Deaf Children with a Cochlear Implant.* The Journal of Deaf Studies and Deaf Education, 2013. **18**(2): p. 175-186.
11. Blijd-Hoogewys, E., et al., *Measuring theory of mind in children. Psychometric properties of the ToM storybooks.* Journal of Autism and Developmental Disorders, 2008. **38**(10): p. 1907-1930.
12. Blijd-Hoogewys, E.M. and P.L. van Geert, *Non-linearities in Theory-of-Mind Development.* Frontiers in Psychology, 2016. **7**: p. 1970.
13. Nader-Grosbois, N., M. Houssa, and S. Mazzone, *How could Theory of Mind contribute to the differentiation of social adjustment profiles of children with externalizing behavior disorders and children with intellectual disabilities?* Research in Developmental Disabilities, 2013. **34**(9): p. 2642-60.
14. Thirion-Marissiaux, A.F. and N. Nader-Grosbois, *Theory of Mind "emotion", developmental characteristics and social understanding in children and adolescents with intellectual disabilities.* Research in Developmental Disabilities, 2008. **29**(5): p. 414-30.
15. Thirion-Marissiaux, A.-F. and N. Nader-Grosbois, *Longitudinal profiles of Theory of Mind development: Cases study of normally developing children and children with Down syndrome.* Devenir, 2008. **20**(4): p. 361-391.
16. Thirion-Marissiaux, A.-F. and N. Nader-Grosbois, *Approach of development of theory of mind in Down syndrome children according to their cognitive, language and socio-affective abilities.* Revue Francophone de la Deficience Intellectuelle, 2006. **17**: p. 12-33.
17. Houssa, M., S. Mazzone, and N. Nader-Grosbois, *Validation of a French version of the Theory of Mind Inventory (ToMI-vf).* European Review of Applied Psychology / Revue Europeenne de Psychologie Appliquee, 2014. **64**(4): p. 169-179.
18. Baurain, C. and N. Nader-Grosbois, *Theory of mind, socio-emotional problem-solving, socio-emotional regulation in children with intellectual disability and in typically developing children.* Journal of Autism and Developmental Disorders, 2013. **43**(5): p. 1080-1097.
19. Fiasse, C. and N. Nader-Grosbois, *Perceived social acceptance, theory of mind and social adjustment in children with intellectual disabilities.* Research in Developmental Disabilities, 2012. **33**(6): p. 1871-80.
20. Nader-Grosbois, N. and M. Houssa, *La Batterie de tâches de Théorie de l'esprit: Validation de la version francophone. [Validation of the French version of the ToM Task Battery.].* Enfance, 2016. **68**(2): p. 141-163.

21. Bensalah, L., S. Caillies, and M. Anduze, *Links among cognitive empathy, theory of mind, and affective perspective taking by young children.* The Journal of Genetic Psychology: Research and Theory on Human Development, 2016. **177**(1): p. 17-31.
22. Thommen, É., et al., *L'évolution de la cognition sociale chez les enfants avec un trouble du spectre de l'autisme: Approche développementale mixte. [Development of social cognition in children with autism spectrum disorders: A combined developmental approach.].* A.N.A.E. Approche Neuropsychologique des Apprentissages chez l'Enfant, 2016. **28**(5[144]): p. 527-537.
23. Paulus, M. and M. Leitherer, *Preschoolers' social experiences and empathy-based responding relate to their fair resource allocation.* Journal of Experimental Child Psychology, 2017. **161**: p. 202-210.
24. Ben-Israel, S., et al., *Dopamine D4 receptor polymorphism and sex interact to predict children's affective knowledge.* Frontiers in Psychology, 2015. **6**: p. 846.
25. Knafo, A., T. Steinberg, and I. Goldner, *Children's low affective perspective-taking ability is associated with low self-initiated pro-sociality.* Emotion, 2011. **11**(1): p. 194-8.
26. Sotgiu, I., et al., *Parental attitudes, attachment styles, social networks, and psychological processes in autism spectrum disorders: a cross-cultural perspective.* Journal of Genetic Psychology, 2011. **172**(4): p. 353-75.
27. Belacchi, C. and E. Farina, *Feeling and thinking of others: Affective and cognitive empathy and emotion comprehension in prosocial/hostile preschoolers.* Aggressive Behavior, 2012. **38**(2): p. 150-165.
28. Lecce, S., et al., *The origins of children's metamemory: The role of theory of mind.* Journal of Experimental Child Psychology, 2015. **131**: p. 56-72.
29. Cigala, A., A. Mori, and F. Fangareggi, *Learning others' point of view: Perspective taking and prosocial behaviour in preschoolers.* Early Child Development and Care, 2015. **185**(8): p. 1199-1215.
30. Ornaghi, V., A. Pepe, and I. Grazzani, *False-Belief Understanding and Language Ability Mediate the Relationship between Emotion Comprehension and Prosocial Orientation in Preschoolers.* Frontiers in Psychology, 2016. **7**: p. 1534.
31. Tsuji, H., *Development of mentalizing ability in Japanese children.* Psychological Studies, 2011. **56**(2): p. 167-175.
32. Kårstad, S.B., et al., *What enhances the development of emotion understanding in young children? A longitudinal study of interpersonal predictors.* British Journal of Developmental Psychology, 2015. **33**(3): p. 340-354.
33. Bulgarelli, D. and P. Molina, *Social Cognition in Preschoolers: Effects of Early Experience and Individual Differences.* Frontiers in Psychology, 2016. **7**: p. 1762.
34. Bulgarelli, D., S. Testa, and P. Molina, *Factorial structure of the 'ToM Storybooks': A test evaluating multiple components of Theory of Mind.* British Journal of Developmental Psychology, 2015. **33**(2): p. 187-202.
35. Salgueiro, E., et al., *Effects of a dolphin interaction program on children with autism spectrum disorders: an exploratory research.* BMC Research Notes, 2012. **5**: p. 199.
36. Barbosa-Leiker, C., et al., *Psychometric properties of the Emotion Understanding Assessment with Spanish- and English-speaking preschoolers attending Head Start.* Assessment, 2014. **21**(5): p. 628-36.
37. Downs, A., P. Strand, and S. Cerna, *Emotion Understanding in English- and Spanish-speaking Preschoolers Enrolled in Head Start.* Social Development, 2007. **16**(3): p. 410-439.
38. Strand, P.S., S. Cerna, and A. Downs, *Shyness and emotion-processing skills in preschoolers: A 6-month longitudinal study.* Infant and Child Development, 2008. **17**(2): p. 109-120.
39. Weimer, A.A. and P.G. Gasquoine, *Belief Reasoning and Emotion Understanding in Balanced Bilingual and Language-Dominant Mexican American Young Children.* Journal of Genetic Psychology, 2016. **177**(2): p. 33-43.
40. Downs, A. and T. Smith, *Emotional understanding, cooperation, and social behavior in high-functioning children with autism.* Journal of Autism & Developmental Disorders, 2004. **34**(6): p. 625-35.

41. Williams, B.T., K.M. Gray, and B.J. Tonge, *Teaching emotion recognition skills to young children with autism: a randomised controlled trial of an emotion training programme.* Journal of Child Psychology & Psychiatry & Allied Disciplines, 2012. **53**(12): p. 1268-76.
42. Muris, P., et al., *The TOM test: a new instrument for assessing theory of mind in normal children and children with pervasive developmental disorders.* Journal of Autism & Developmental Disorders, 1999. **29**(1): p. 67-80.
43. Tager-Flusberg, H. and K. Sullivan, *Predicting and Explaining Behavior: A Comparison of Autistic, Mentally Retarded and Normal Children.* Journal of Child Psychology and Psychiatry, 1994. **35**(6): p. 1059-1075.
44. MacQuiddy, S.L., S.J. Maise, and S.B. Hamilton, *Empathy and affective perspective-taking skills in parent-identified conduct-disordered boys.* Journal of Clinical Child Psychology, 1987. **16**(3): p. 260-268.
45. Tager-Flusberg, H. and K. Sullivan, *A componential view of theory of mind: evidence from Williams syndrome.* Cognition, 2000. **76**(1): p. 59-90.
46. Wolfe, K.R., et al., *Self-awareness of peer-rated social attributes in children with traumatic brain injury.* Journal of Pediatric Psychology, 2015. **40**(3): p. 272-284.
47. Garner, P.W., D.C. Jones, and J.L. Miner, *Social Competence among Low-Income Preschoolers: Emotion Socialization Practices and Social Cognitive Correlates.* Child Development, 1994. **65**(2): p. 622-637.
48. Blankson, A.N., et al., *Cognitive and emotional processes as predictors of a successful transition into school.* Early Education and Development, 2017. **28**(1): p. 1-20.
49. Pons, F., P.L. Harris, and M. de Rosnay, *Emotion comprehension between 3 and 11 years: Developmental periods and hierarchical organization.* European Journal of Developmental Psychology, 2004. **1**(2): p. 127-152.
50. Centifanti, L.C.M., E. Meins, and C. Fernyhough, *Callous-unemotional traits and impulsivity: Distinct longitudinal relations with mind-mindedness and understanding of others.* Journal of Child Psychology and Psychiatry, 2016. **57**(1): p. 84-92.
51. Cliff, D.P., et al., *The Preschool Activity, Technology, Health, Adiposity, Behaviour and Cognition (PATH-ABC) cohort study: rationale and design.* BMC Pediatrics, 2017. **17**(1): p. 95.
52. Weimer, A.A., J. Sallquist, and R.R. Bolnick, *Young children's emotion comprehension and theory of mind understanding.* Early Education and Development, 2012. **23**(3): p. 280-301.
53. De Rosnay, M., et al., *Talking theory of mind talk: young school-aged children's everyday conversation and understanding of mind and emotion.* Journal of Child Language, 2014. **41**(5): p. 1179-93.
54. Martins, E.C., et al., *Emotion understanding in preschool children: The role of executive functions.* International Journal of Behavioral Development, 2016. **40**(1): p. 1-10.
55. Ribordy, S.C., et al., *Vignettes for Emotion Recognition Research and Affective Therapy With Children.* Journal of Clinical Child Psychology, 1988. **17**(4): p. 322-325.
56. Knafo, A., et al., *Empathy in early childhood: genetic, environmental, and affective contributions.* Ann N Y Acad Sci, 2009. **1167**: p. 103-14.
57. Seidenfeld, A.M., et al., *Theory of mind predicts emotion knowledge development in Head Start children.* Early Education and Development, 2014. **25**(7): p. 933-948.
58. Taumoepeau, M. and T. Ruffman, *Mother and Infant Talk About Mental States Relates to Desire Language and Emotion Understanding.* Child Development, 2006. **77**(2): p. 465-481.
59. Veiga, G., et al., *Alone at the playground.* European Journal of Developmental Psychology, 2017. **14**(1): p. 44-61.
60. Veiga, G., C. Neto, and C. Rieffe, *Preschoolers' free play—Connections with emotional and social functioning.* The International Journal of Emotional Education, 2016. **8**(1): p. 48-62.
61. Klemchuk, H.P., L.A. Bond, and D.C. Howell, *Coherence and correlates of Level 1 perspective taking in young children.* Merrill-Palmer Quarterly, 1990. **36**(3): p. 369-387.

62. Burns, N. and L. Cavey, *Age differences in emphatic ability among children.* Canadian Journal of Psychology/Revue canadienne de psychologie, 1957. **11**(4): p. 227-230.
63. Diesendruck, G. and A. Ben-Eliyahu, *The relationships among social cognition, peer acceptance, and social behavior in Israeli kindergartners.* International Journal of Behavioral Development, 2006. **30**(2): p. 137-147.
64. Grazzani, I., V. Ornaghi, and J. Brockmeier, *Conversation on mental states at nursery: Promoting social cognition in early childhood.* European Journal of Developmental Psychology, 2016. **13**(5): p. 563-581.
65. Conte, E., et al., *Emotion Knowledge, Theory of Mind, and Language in Young Children: Testing a Comprehensive Conceptual Model.* Frontiers in Psychology, 2019. **10**: p. 2144.
66. Wang, Y., H. Liu, and Y. Su, *Development of preschoolers' emotion and false belief understanding: A longitudinal study.* Social Behavior and Personality, 2014. **42**(4): p. 645-654.
67. Wisdom, S.N., et al., *Can autism, language and coordination disorders be differentiated based on ability profiles?* European Child & Adolescent Psychiatry, 2007. **16**(3): p. 178-86.
68. Dyck, M.J., et al., *The relationship between symptoms and abilities in autism.* Journal of Developmental and Physical Disabilities, 2007. **19**(3): p. 251-261.
69. O'Kearney, R., et al., *Emotional Abilities in Children with Oppositional Defiant Disorder (ODD): Impairments in Perspective-Taking and Understanding Mixed Emotions are Associated with High Callous-Unemotional Traits.* Child Psychiatry & Human Development, 2017. **48**(2): p. 346-357.
70. Pears, K.C. and P.A. Fisher, *Emotion understanding and theory of mind among maltreated children in foster care: Evidence of deficits.* Development and Psychopathology, 2005. **17**(1): p. 47-65.
71. Pears, K.C. and L.J. Moses, *Demographics, parenting, and theory of mind in preschool children.* Social Development, 2003. **12**(1): p. 1-19.
72. Cassidy, K.W., et al., *The Relationship Between Psychological Understanding and Positive Social Behaviors.* Social Development, 2003. **12**(2): p. 198-221.
73. Hughes, C., J. Dunn, and A. White, *Trick or treat?: uneven understanding of mind and emotion and executive dysfunction in "hard-to-manage" preschoolers.* Journal of Child Psychology & Psychiatry & Allied Disciplines, 1998. **39**(7): p. 981-94.
74. Hughes, C., et al., *Antisocial, angry, and unsympathetic: "hard-to-manage" preschoolers' peer problems and possible cognitive influences.* Journal of Child Psychology & Psychiatry & Allied Disciplines, 2000. **41**(2): p. 169-79.
75. Mathieson, K. and R. Banerjee, *Peer play, emotion understanding, and socio-moral explanation: the role of gender.* British Journal of Developmental Psychology, 2011. **29**(Pt 2): p. 188-96.
76. McElwain, N.L. and B.L. Volling, *Relating individual control, social understanding, and gender to child-friend interaction: A relationships perspective.* Social Development, 2002. **11**(3): p. 362-385.
77. Ruffman, T., et al., *What mothers say and what they do: The relation between parenting, theory of mind, language and conflict/cooperation.* British Journal of Developmental Psychology, 2006. **24**(1): p. 105-124.
78. Dunn, J., *Children as psychologists: The later correlates of individual differences in understanding of emotions and other minds.* Cognition and Emotion, 1995. **9**(2-3): p. 187-201.
79. Ruffman, T., et al., *How language relates to belief, desire, and emotion understanding.* Cognitive Development, 2003. **18**(2): p. 139-158.
80. Slomkowski, C. and J. Dunn, *Young children's understanding of other people's beliefs and feelings and their connected communication with friends.* Developmental Psychology, 1996. **32**(3): p. 442-447.
81. Choe, D.E., et al., *Developmental precursors of young school-age children's hostile attribution bias.* Developmental Psychology, 2013. **49**(12): p. 2245-56.
82. Cutting, A.L. and J. Dunn, *Theory of mind, emotion understanding, language, and family background: individual differences and interrelations.* Child Development, 1999. **70**(4): p. 853-65.
83. Dunn, J. and A.L. Cutting, *Understanding others, and individual differences in friendship interactions in young children.* Social Development, 1999. **8**(2): p. 201-219.

84. Eggum, N.D., et al., *Emotion understanding, theory of mind, and prosocial orientation: Relations over time in early childhood.* The Journal of Positive Psychology, 2011. **6**(1): p. 4-16.
85. Ereky-Stevens, K., *Associations between mothers' sensitivity to their infants' internal states and children's later understanding of mind and emotion.* Infant and Child Development, 2008. **17**(5): p. 527-543.
86. Dunn, J., et al., *Young children's understanding of other people's feelings and beliefs: individual differences and their antecedents.* Child Development, 1991. **62**(6): p. 1352-66.
87. Hinnant, J. and M. O'Brien, *Cognitive and emotional control and perspective taking and their relations to empathy in 5-year-old children.* The Journal of Genetic Psychology: Research and Theory on Human Development, 2007. **168**(3): p. 301-322.
88. LaBounty, J., et al., *Mothers' and fathers' use of internal state talk with their young children.* Social Development, 2008. **17**(4): p. 757-775.
89. Lane, J.D., et al., *Theory of mind and emotion understanding predict moral development in early childhood.* British Journal of Developmental Psychology, 2010. **28**(Pt 4): p. 871-89.
90. Hughes, C. and T. Cline, *An evaluation of the preschool PATHS curriculum on the development of preschool children.* Educational Psychology in Practice, 2015. **31**(1): p. 73-85.
91. Denham, S.A., et al., *Emotional and Behavioral Predictors of Preschool Peer Ratings.* Child Development, 1990. **61**(4): p. 1145-1152.
92. Youngblade, L.M. and J. Dunn, *Individual differences in young children's pretend play with mother and sibling: links to relationships and understanding of other people's feelings and beliefs.* Child Development, 1995. **66**(5): p. 1472-92.
93. LaBounty, J., et al., *Relationship between social cognition and temperament in preschool-aged children.* Infant and Child Development, 2017. **26**(2): p. No Pagination Specified-No Pagination Specified.
94. Tarullo, A.R., et al., *Emotion understanding, parent mental state language, and behavior problems in internationally adopted children.* Development and Psychopathology, 2015. **28**(2): p. 371-383.
95. Khu, M., C. Chambers, and S.A. Graham, *When You're Happy and I Know It: Four-Year-Olds' Emotional Perspective Taking During Online Language Comprehension.* Child Development, 2018. **89**(6): p. 2264-2281.
96. LeMare, L.J. and K.H. Rubin, *Perspective taking and peer interaction: Structural and developmental analyses.* Child Development, 1987. **58**(2): p. 306-315.
97. Taylor, M., et al., *The characteristics and correlates of fantasy in school-age children: imaginary companions, impersonation, and social understanding.* Developmental Psychology, 2004. **40**(6): p. 1173-87.
98. Carlo, G., et al., *Cognitive processes and prosocial behaviors among children: The role of affective attributions and reconciliations.* Developmental Psychology, 1991. **27**(3): p. 456-461.
99. Lempers, J., et al., *The relationship between psychometric brightness and cognitive-developmental precocity in gifted preschoolers.* Merrill-Palmer Quarterly, 1987. **33**(4): p. 489-503.
100. Minde, K., *Aggression in preschoolers: Its relation to socialization.* Journal of the American Academy of Child & Adolescent Psychiatry, 1992. **31**(5): p. 853-862.
101. Borke, H., *Interpersonal perception of young children: Egocentrism or empathy?* Developmental Psychology, 1971. **5**(2): p. 263-269.
102. Gopnik, A. and V. Slaughter, *Young Children's Understanding of Changes in Their Mental States.* Child Development, 1991. **62**(1): p. 98-110.
103. Lecce, S., M. Caputi, and C. Hughes, *Does sensitivity to criticism mediate the relationship between theory of mind and academic achievement?* Journal of Experimental Child Psychology, 2011. **110**(3): p. 313-31.
104. Lecce, S., M. Caputi, and A. Pagnin, *Long-term effect of theory of mind on school achievement: The role of sensitivity to criticism.* European Journal of Developmental Psychology, 2014. **11**(3): p. 305-318.
105. Lecce, S. and C. Hughes, *'The Italian job?': comparing theory of mind performance in British and Italian children.* British Journal of Developmental Psychology, 2010. **28**(Pt 4): p. 747-66.

106. Caputi, M., et al., *Longitudinal effects of theory of mind on later peer relations: the role of prosocial behavior.* Developmental Psychology, 2012. **48**(1): p. 257-70.
107. Park, J. and J. Lee, *Dyadic collaboration among preschool-age children and the benefits of working with a more socially advanced peer.* Early Education and Development, 2015. **26**(4): p. 574-593.
108. Ryan, N.P., et al., *Theory of mind mediates the prospective relationship between abnormal social brain network morphology and chronic behavior problems after pediatric traumatic brain injury.* Social Cognitive & Affective Neuroscience, 2016. **11**(4): p. 683-92.
109. Kuntoro, I.A., et al., *Micro-cultural influences on theory of mind development: A comparative study of middle-class and pemulung children in Jakarta, Indonesia.* International Journal of Behavioral Development, 2013. **37**(3): p. 266-273.
110. Dixson, H.G.W., et al., *Scaling Theory of Mind in a Small-Scale Society: A Case Study From Vanuatu.* Child Development, 2018. **89**(6): p. 2157-2175.
111. Zhang, T., Z. Shao, and Y. Zhang, *Developmental steps in theory of mind of typical Chinese children and Chinese children with autism spectrum disorder.* Research in Autism Spectrum Disorders, 2016. **23**: p. 210-220.
112. Wellman, H.M., F. Fang, and C.C. Peterson, *Sequential progressions in a theory-of-mind scale: Longitudinal perspectives.* Child Development, 2011. **82**(3): p. 780-792.
113. Ketelaars, M.P., et al., *Dynamics of the theory of mind construct: A developmental perspective.* European Journal of Developmental Psychology, 2010. **7**(1): p. 85-103.
114. Shahaeian, A., et al., *Cultural and family influences on children's theory of mind development: A comparison of Australian and Iranian school-age children.* Journal of Cross-Cultural Psychology, 2014. **45**(4): p. 555-568.
115. Shahaeian, A., et al., *Iranian mothers' disciplinary strategies and theory of mind in children: A focus on belief understanding.* Journal of Cross-Cultural Psychology, 2014. **45**(7): p. 1110-1123.
116. Shahaeian, A., et al., *Culture and the sequence of steps in theory of mind development.* Developmental Psychology, 2011. **47**(5): p. 1239-47.
117. Cochet, H., et al., *Relationships between declarative pointing and theory of mind abilities in 3- to 4-year-olds.* European Journal of Developmental Psychology, 2017. **14**(3): p. 324-336.
118. Burnel, M., et al., *Reducing the language content in ToM tests: A developmental scale.* Developmental Psychology, 2018. **54**(2): p. 293-307.
119. Jester, M. and C.J. Johnson, *Differences in theory of mind and pretend play associations in children with and without specific language impairment.* Infant and Child Development, 2016. **25**(1): p. 24-42.
120. Aschersleben, G., T. Hofer, and B. Jovanovic, *The link between infant attention to goal-directed action and later theory of mind abilities.* Developmental Science, 2008. **11**(6): p. 862-8.
121. Henning, A., F.M. Spinath, and G. Aschersleben, *The link between preschoolers' executive function and theory of mind and the role of epistemic states.* Journal of Experimental Child Psychology, 2011. **108**(3): p. 513-31.
122. Witt, S., et al., *Delayed theory of mind development in children born preterm: A longitudinal study.* Early Human Development, 2018. **127**: p. 85-89.
123. Wellman, H.M., et al., *Scaling of theory-of-mind understandings in Chinese children.* Psychological Science, 2006. **17**(12): p. 1075-81.
124. Duh, S., et al., *Theory of mind and executive function in Chinese preschool children.* Developmental Psychology, 2016. **52**(4): p. 582-591.
125. Ding, X.P., et al., *Theory-of-mind training causes honest young children to lie.* Psychological Science, 2015. **26**(11): p. 1812-1821.
126. Smogorzewska, J., G. Szumski, and P. Grygiel, *Same or different? Theory of mind among children with and without disabilities.* PLoS ONE [Electronic Resource], 2018. **13**(10): p. e0202553.
127. Smogorzewska, J., G. Szumski, and P. Grygiel, *The Children’s Social Understanding Scale: An advanced analysis of a parent-report measure for assessing theory of mind in Polish children with and without disabilities.* Developmental Psychology, 2019. **55**(4): p. 835-845.
128. Azurmendi, A., et al., *Cognitive abilities, androgen levels, and body mass index in 5-year-old children.* Hormones & Behavior, 2005. **48**(2): p. 187-95.
129. Fernandez, C., *Mindful storytellers: Emerging pragmatics and theory of mind development.* First Language, 2013. **33**(1): p. 20-46.

130. Strasser, K. and F. del Rio, *The role of comprehension monitoring, theory of mind, and vocabulary depth in predicting story comprehension and recall of kindergarten children.* Reading Research Quarterly, 2014. **49**(2): p. 169-187.
131. Etel, E. and B. Yagmurlu, *Social competence, theory of mind, and executive function in institution-reared Turkish children.* International Journal of Behavioral Development, 2015. **39**(6): p. 519-529.
132. Doenyas, C., H.M. Yavuz, and B. Selcuk, *Not just a sum of its parts: How tasks of the theory of mind scale relate to executive function across time.* Journal of Experimental Child Psychology, 2018. **166**: p. 485-501.
133. Peterson, C.C., H.M. Wellman, and D. Liu, *Steps in theory-of-mind development for children with deafness or autism.* Child Development, 2005. **76**(2): p. 502-17.
134. Wong, P.M. and S.O. Leung, *The development of theory of mind ability in autistic children.* Psychologia: An International Journal of Psychological Sciences, 2010. **53**(3): p. 151-162.
135. Paynter, J. and C.C. Peterson, *Further evidence of benefits of thought-bubble training for theory of mind development in children with autism spectrum disorders.* Research in Autism Spectrum Disorders, 2013. **7**(2): p. 344-348.
136. de, C., R. Brindley, and U. Frith, *Visual perspective taking impairment in children with autistic spectrum disorder.* Cognition, 2009. **113**(1): p. 37-44.
137. Peterson, C.C., V. Slaughter, and C. Brownell, *Children with autism spectrum disorder are skilled at reading emotion body language.* Journal of Experimental Child Psychology, 2015. **139**: p. 35-50.
138. Kang, E., et al., *Predictors and Moderators of Spontaneous Pretend Play in Children with and without Autism Spectrum Disorder.* Frontiers in Psychology, 2016. **7**: p. 1577.
139. Knutsen, J., D.S. Mandell, and D. Frye, *Children with autism are impaired in the understanding of teaching.* Developmental Science, 2017. **20**(2): p. No Pagination Specified-No Pagination Specified.
140. Tsang, T., K. Gillespie-Lynch, and T. Hutman, *Theory of Mind Indexes the Broader Autism Phenotype in Siblings of Children with Autism at School Age.* Autism Research & Treatment Print, 2016. **2016**: p. 6309189.
141. Lee, M., et al., *A Multi-Method Investigation of Pragmatic Development in Individuals With Down Syndrome.* American Journal on Intellectual & Developmental Disabilities, 2017. **122**(4): p. 289-309.
142. Remmel, E. and K. Peters, *Theory of mind and language in children with cochlear implants.* Journal of Deaf Studies & Deaf Education, 2009. **14**(2): p. 218-36.
143. Wellman, H.M. and C.C. Peterson, *Deafness, thought bubbles, and theory-of-mind development.* Developmental Psychology, 2013. **49**(12): p. 2357-67.
144. Peterson, C.C., H.M. Wellman, and V. Slaughter, *The mind behind the message: advancing theory-of-mind scales for typically developing children, and those with deafness, autism, or Asperger syndrome.* Child Development, 2012. **83**(2): p. 469-85.
145. Farrant, B.M., J. Fletcher, and M.T. Maybery, *Specific Language Impairment, Theory of Mind, and Visual Perspective Taking: Evidence for Simulation Theory and the Developmental Role of Language.* Child Development, 2006. **77**(6): p. 1842-1853.
146. Wolfe, K.R., et al., *Executive functions, social information processing, and social adjustment in young children born with very low birth weight.* Child Neuropsychology, 2015. **21**(1): p. 41-54.
147. Holmes-Lonergan, H.A., *Understanding of affective false beliefs, perceptions of parental discipline, and classroom behavior in children from Head Start.* Early Education and Development, 2003. **14**(1): p. 29-46.
148. O'Reilly, J. and C.C. Peterson, *Maltreatment and advanced theory of mind development in school-aged children.* Journal of Family Violence, 2015. **30**(1): p. 93-102.
149. Wade, M. and J.M. Jenkins, *Pregnancy hypertension and the risk for neuropsychological difficulties across early development: A brief report.* Child Neuropsychology, 2016. **22**(2): p. 247-54.
150. Ryan, N.P., et al., *Relationships between acute imaging biomarkers and theory of mind impairment in post-acute pediatric traumatic brain injury: A prospective analysis using susceptibility weighted imaging (SWI).* Neuropsychologia, 2015. **66**: p. 32-8.

151. Miller, M.R., et al., *The contribution of executive function and social understanding to preschoolers' letter and math skills.* Cognitive Development, 2013. **28**(4): p. 331-349.
152. Rice, K., et al., *Amygdala volume linked to individual differences in mental state inference in early childhood and adulthood.* Developmental Cognitive Neuroscience, 2014. **8**: p. 153-163.
153. Lucariello, J.M., T.M. Durand, and L. Yarnell, *Social versus intrapersonal ToM: Social ToM is a cognitive strength for low- and middle-SES children.* Journal of Applied Developmental Psychology, 2007. **28**(4): p. 285-297.
154. Banerjee, R. and N. Yuill, *Children's understanding of self-presentational display rules: Associations with mental-state understanding.* British Journal of Developmental Psychology, 1999. **17**(Pt 1): p. 111-124.
155. Misailidi, P., *Children's understanding of display rules: The role of second-order intentions.* Hellenic Journal of Psychology, 2007. **4**(1): p. 36-51.
156. Harris, P.L., et al., *Children's Understanding of the Distinction between Real and Apparent Emotion.* Child Development, 1986. **57**(4): p. 895-909.
157. Davis, T.L., *Children's understanding of false beliefs in different domains: Affective vs. physical.* British Journal of Developmental Psychology, 2001. **19**(1): p. 47-58.
158. Wellman, H.M. and D. Liu, *Scaling of theory-of-mind tasks.* Child Development, 2004. **75**(2): p. 523-41.
159. Hiller, R.M., N. Weber, and R.L. Young, *The validity and scalability of the Theory of Mind Scale with toddlers and preschoolers.* Psychological Assessment, 2014. **26**(4): p. 1388-93.
160. Wellman, H.M., et al., *Infant social attention predicts preschool social cognition.* Developmental Science, 2004. **7**(3): p. 283-8.
161. Dore, R.A. and A.S. Lillard, *Theory of mind and children's engagement in fantasy worlds.* Imagination, Cognition and Personality, 2015. **34**(3): p. 230-242.
162. Gola, A.A.H., *Mental verb input for promoting children's theory of mind: A training study.* Cognitive Development, 2012. **27**(1): p. 64-76.
163. O'Reilly, J. and C.C. Peterson, *Theory of mind at home: Linking authoritative and authoritarian parenting styles to children's social understanding.* Early Child Development and Care, 2014. **184**(12): p. 1934-1947.
164. Olineck, K.M. and D. Poulin-Dubois, *Imitation of intentional actions and internal state language in infancy predict preschool theory of mind skills.* European Journal of Developmental Psychology, 2007. **4**(1): p. 14-30.
165. d'Entremont, B., E. Seamans, and E. Boudreau, *The relationship between children's gaze reporting and theory of mind.* Journal of Cognition and Development, 2012. **13**(4): p. 505-523.
166. Sabbagh, M.A. and E.L. Seamans, *Intergenerational transmission of theory-of-mind.* Developmental Science, 2008. **11**(3): p. 354-60.
167. Lillard, A.S., *Preschool children's development in classic Montessori, supplemented Montessori, and conventional programs.* Journal of School Psychology, 2012. **50**(3): p. 379-401.
168. Wade, M., et al., *Normal birth weight variation and children's neuropsychological functioning: Links between language, executive functioning, and theory of mind.* Journal of the International Neuropsychological Society, 2014. **20**(9): p. 909-919.
169. Brooks, R. and A.N. Meltzoff, *Connecting the dots from infancy to childhood: a longitudinal study connecting gaze following, language, and explicit theory of mind.* Journal of Experimental Child Psychology, 2015. **130**: p. 67-78.
170. Wellman, H.M., et al., *Infant attention to intentional action predicts preschool theory of mind.* Developmental Psychology, 2008. **44**(2): p. 618-23.
171. White, R.E. and S.M. Carlson, *What would batman do? Self-distancing improves executive function in young children.* Developmental Science, 2015: p. No Pagination Specified.
172. Vanderbilt, K.E., D. Liu, and G.D. Heyman, *The development of distrust.* Child Development, 2011. **82**(5): p. 1372-1380.
173. Dore, R.A. and A.S. Lillard, *Do children prefer mentalistic descriptions?* The Journal of Genetic Psychology: Research and Theory on Human Development, 2014. **175**(1): p. 1-15.
174. Mar, R.A., J.L. Tackett, and C. Moore, *Exposure to media and theory-of-mind development in preschoolers.* Cognitive Development, 2010. **25**(1): p. 69-78.
175. McAlister, A.R. and C.C. Peterson, *Siblings, theory of mind, and executive functioning in children aged 3-6 years: new longitudinal evidence.* Child Development, 2013. **84**(4): p. 1442-58.

176. Karpinski, A.C. and M.H. Scullin, *Suggestibility under pressure: Theory of mind, executive function, and suggestibility in preschoolers.* Journal of Applied Developmental Psychology, 2009. **30**(6): p. 749-763.
177. Shahaeian, A., *Sibling, family, and social influences on children's theory of mind understanding: New evidence from diverse intracultural samples.* Journal of Cross-Cultural Psychology, 2015. **46**(6): p. 805-820.
178. Shahaeian, A., et al., *Towards a better understanding of the relationship between executive control and theory of mind: an intra-cultural comparison of three diverse samples.* Developmental Science, 2015. **18**(5): p. 671-85.
179. Wade, M., T.J. Hoffmann, and J.M. Jenkins, *Gene-environment interaction between the oxytocin receptor (OXTR) gene and parenting behaviour on children's theory of mind.* Social Cognitive & Affective Neuroscience, 2015. **10**(12): p. 1749-57.
180. Brown, M.M., et al., *Supporting the development of empathy: The role of theory of mind and fantasy orientation.* Social Development, 2017: p. No Pagination Specified-No Pagination Specified.
181. Song, J.H., et al., *Aggression, Sibling Antagonism, and Theory of Mind During the First Year of Siblinghood: A Developmental Cascade Model.* Child Development, 2016. **87**(4): p. 1250-63.
182. Gordon, K.R., *High proficiency across two languages is related to better mental state reasoning for bilingual children.* Journal of Child Language, 2016. **43**(2): p. 407-424.
183. Pauker, S., et al., *Differential parenting and children's social understanding.* Social Development, 2016: p. No Pagination Specified-No Pagination Specified.
184. Wade, M., et al., *Cumulative risk disparities in children's neurocognitive functioning: A developmental cascade model.* Developmental Science, 2015: p. No Pagination Specified.
185. Prime, H., et al., *The development of a measure of maternal cognitive sensitivity appropriate for use in primary care health settings.* Journal of Child Psychology and Psychiatry, 2015. **56**(4): p. 488-495.
186. Nathanson, A.I. and P.T. Fries, *Television exposure, sleep time, and neuropsychological function among preschoolers.* Media Psychology, 2014. **17**(3): p. 237-261.
187. Kuhnert, R.L., et al., *Gender-differentiated effects of theory of mind, emotion understanding, and social preference on prosocial behavior development: A longitudinal study.* Journal of Experimental Child Psychology, 2017. **154**: p. 13-27.
188. Hasni, A.A., et al., *Adding sound to theory of mind: Comparing children's development of mental-state understanding in the auditory and visual realms.* Journal of Experimental Child Psychology, 2017. **164**: p. 239-249.
189. Jambon, M., et al., *Developmental trajectories of physical aggression and prosocial behavior in early childhood: Family antecedents and psychological correlates.* Developmental Psychology, 2019. **55**(6): p. 1211-1225.
190. Song, J.H. and B. Volling, *Theory-of-Mind Development and Early Sibling Relationships after the Birth of a Sibling: Parental Discipline Matters.* Infant & Child Development, 2018. **27**(1): p. Jan-Feb.
191. Wade, M., et al., *Cumulative psychosocial risk, parental socialization, and child cognitive functioning: A longitudinal cascade model.* Developmental Psychology, 2018. **54**(6): p. 1038-1050.
192. Lillard, A.S., et al., *Montessori Preschool Elevates and Equalizes Child Outcomes: A Longitudinal Study.* Frontiers in Psychology, 2017. **8**: p. 1783.
193. Warnell, K.R. and E. Redcay, *Minimal coherence among varied theory of mind measures in childhood and adulthood.* Cognition, 2019. **191**.
194. Cliff, D.P., et al., *Adherence to 24-Hour Movement Guidelines for the Early Years and associations with social-cognitive development among Australian preschool children.* BMC Public Health, 2017. **17**(Suppl 5): p. 857.
195. Fidalgo, A.M., H.R. Tenenbaum, and A. Aznar, *Are there gender differences in emotion comprehension? Analysis of the Test of Emotion Comprehension.* Journal of Child and Family Studies, 2018. **27**(4): p. 1065-1074.
196. Pellicano, E. and G. Rhodes, *The role of eye-gaze in understanding other minds.* British Journal of Developmental Psychology, 2003. **21**(1): p. 33-43.

197. van Buijsen, M., et al., *Assessment of theory of mind in children with communication disorders: role of presentation mode.* Research in Developmental Disabilities, 2011. **32**(3): p. 1038-45.
198. Slaughter, V., M.J. Dennis, and M. Pritchard, *Theory of mind and peer acceptance in preschool children.* British Journal of Developmental Psychology, 2002. **20**(4): p. 545-564.
199. Baron-Cohen, S., et al., *Are children with autism blind to the mentalistic significance of the eyes?* British Journal of Developmental Psychology, 1995. **13**(4): p. 379-398.
200. Arranz, E., et al., *Family context and theory of mind development.* Early Child Development and Care, 2002. **172**(1): p. 9-22.
201. Xu, W., *Relationship between children's theory of mind and moral judgement.* Chinese Journal of Clinical Psychology, 2015. **23**(1): p. 67-70.
202. Jin, X., et al., *Cooperation, but not competition, improves 4-year-old children's reasoning about others' diverse desires.* Journal of Experimental Child Psychology, 2017. **157**: p. 81-94.
203. Ketelaar, L., et al., *Does hearing lead to understanding? Theory of mind in toddlers and preschoolers with cochlear implants.* Journal of Pediatric Psychology, 2012. **37**(9): p. 1041-1050.
204. Rieffe, C., L. Ketelaar, and C.H. Wiefferink, *Assessing empathy in young children: Construction and validation of an Empathy Questionnaire (EmQue).* Personality and Individual Differences, 2010. **49**(5): p. 362-367.
205. Netten, A.P., et al., *Can You Hear What I Think? Theory of Mind in Young Children With Moderate Hearing Loss.* Ear & Hearing, 2017. **38**(5): p. 588-597.
206. Laranjo, J., et al., *Early manifestations of children's theory of mind: The roles of maternal mind-mindedness and infant security of attachment.* Infancy, 2010. **15**(3): p. 300-323.
207. Wright, K. and D. Poulin-Dubois, *Modified Checklist for Autism in Toddlers (M-CHAT) screening at 18 months of age predicts concurrent understanding of desires, word learning and expressive vocabulary.* Research in Autism Spectrum Disorders, 2012. **6**(1): p. 184-192.
208. Chiarella, S.S., et al., *Concurrent relations between perspective-taking skills, desire understanding, and internal-state vocabulary.* Journal of Cognition and Development, 2013. **14**(3): p. 480-498.
209. Laranjo, J., et al., *The roles of maternal mind-mindedness and infant security of attachment in predicting preschoolers' understanding of visual perspective taking and false belief.* Journal of Experimental Child Psychology, 2014. **125**: p. 48-62.
210. Bellerose, J., et al., *When Injury Clouds Understanding of Others: Theory of Mind after Mild TBI in Preschool Children.* Journal of the International Neuropsychological Society, 2015. **21**(7): p. 483-93.
211. Yott, J. and D. Poulin-Dubois, *Are infants' theory-of-mind abilities well integrated? Implicit understanding of intentions, desires, and beliefs.* Journal of Cognition and Development, 2016. **17**(5): p. 683-698.
212. Bellerose, J., et al., *Long-term brain-injury-specific effects following preschool mild TBI: A study of theory of mind.* Neuropsychology, 2017. **31**(3): p. 229-241.
213. Poulin-Dubois, D. and J. Yott, *Probing the depth of infants' theory of mind: disunity in performance across paradigms.* Developmental Science, 2018. **21**(4): p. e12600.
214. Sodian, B., et al., *Understanding of goals, beliefs, and desires predicts morally relevant theory of mind: A longitudinal investigation.* Child Development, 2016. **87**(4): p. 1221-1232.
215. Burkart, J.M. and K. Rueth, *Preschool children fail primate prosocial game because of attentional task demands.* PLoS ONE [Electronic Resource], 2013. **8**(7): p. e68440.
216. Mink, D., A. Henning, and G. Aschersleben, *Infant shy temperament predicts preschoolers Theory of Mind.* Infant Behavior & Development, 2014. **37**(1): p. 66-75.
217. Thoermer, C., et al., *Continuity from an implicit to an explicit understanding of false belief from infancy to preschool age.* British Journal of Developmental Psychology, 2012. **30**(Pt 1): p. 172-87.

218. Tanaka, E., et al., *Implications of social competence among thirty-month-old toddlers: a theory of mind perspective.* Journal of Epidemiology, 2010. **20 Suppl 2**: p. S447-51.
219. Liu, M., et al., *The relationships among verbal ability, executive function, and theory of mind in young children with cochlear implants.* International Journal of Audiology, 2018. **57**(12): p. 875-882.
220. Slaughter, V., C.C. Peterson, and E. Mackintosh, *Mind what mother says: narrative input and theory of mind in typical children and those on the autism spectrum.* Child Development, 2007. **78**(3): p. 839-58.
221. Hanson, L.K. and C.M. Atance, *Brief report: Episodic foresight in autism spectrum disorder.* Journal of Autism and Developmental Disorders, 2014. **44**(3): p. 674-684.
222. Hamilton, A.F.d.C., R.M. Brindley, and U. Frith, *Imitation and action understanding in autistic spectrum disorders: How valid is the hypothesis of a deficit in the mirror neuron system?* Neuropsychologia, 2007. **45**(8): p. 1859-1868.
223. Losh, M., et al., *Social communication and theory of mind in boys with autism and fragile x syndrome.* Frontiers in Psychology, 2012. **3**: p. 266.
224. Pearson, A., et al., *Cognitive mechanisms underlying visual perspective taking in typical and ASC children.* Autism Research, 2016. **9**(1): p. 121-130.
225. Fidler, D.J., et al., *Emotional responsivity in young children with Williams syndrome.* American Journal on Mental Retardation, 2007. **112**(3): p. 194-206.
226. Tucci, S.L., S.R. Easterbrooks, and A.R. Lederberg, *The Effects of Theory of Mind Training on the False Belief Understanding of Deaf and Hard-of-Hearing Students in Prekindergarten and Kindergarten.* Journal of Deaf Studies & Deaf Education, 2016. **21**(3): p. 310-25.
227. Leduc, K., et al., *The contributions of mental state understanding and executive functioning to preschool-aged children's lie-telling.* British Journal of Developmental Psychology, 2017. **35**(2): p. 288-302.
228. Bartsch, K. and H. Wellman, *Young Children's Attribution of Action to Beliefs and Desires.* Child Development, 1989. **60**(4): p. 946-964.
229. Carpendale, J.I. and M.J. Chandler, *On the Distinction between False Belief Understanding and Subscribing to an Interpretive Theory of Mind.* Child Development, 1996. **67**(4): p. 1686-1706.
230. Iannotti, R.J., *Naturalistic and structured assessments of prosocial behavior in preschool children: The influence of empathy and perspective taking.* Developmental Psychology, 1985. **21**(1): p. 46-55.
231. Nguyen, L. and D. Frye, *Children's theory of mind: Understanding of desire, belief and emotion with social referents.* Social Development, 1999. **8**(1): p. 70-92.
232. Lim, A.K., et al., *Cross-cultural variations in naive psychology among 2-year-olds: A comparison of children in the United Kingdom and Singapore.* Journal of Cognition and Culture, 2010. **10**(3-4): p. 221-251.
233. Carlson, S.M., D.J. Mandell, and L. Williams, *Executive function and theory of mind: stability and prediction from ages 2 to 3.* Developmental Psychology, 2004. **40**(6): p. 1105-22.
234. Repacholi, B.M. and A. Gopnik, *Early Reasoning About Desires: Evidence From 14- and 18-Month-Olds.* Developmental Psychology, 1997. **33**(1): p. 12-21.
235. Rieffe, C., et al., *Preschoolers' appreciation of uncommon desires and subsequent emotions.* British Journal of Developmental Psychology, 2001. **19**(2): p. 259-274.
236. Wellman, H.M. and J.D. Woolley, *From simple desires to ordinary beliefs: the early development of everyday psychology.* Cognition, 1990. **35**(3): p. 245-75.
237. Hanson, L.K., C.M. Atance, and S.W. Paluck, *Is thinking about the future related to theory of mind and executive function? Not in preschoolers.* Journal of Experimental Child Psychology, 2014. **128**: p. 120-37.
238. Dunphy-Lelii, S., *Initial investigations into preschoolers' mirror-style versus transposed bodily imitation.* Canadian Journal of Experimental Psychology/Revue canadienne de psychologie experimentale, 2014. **68**(1): p. 38-45.
239. Muller, U., et al., *Knowing minds, controlling actions: the developmental relations between theory of mind and executive function from 2 to 4 years of age.* Journal of Experimental Child Psychology, 2012. **111**(2): p. 331-48.

240. Smetana, J.G., et al., *Reciprocal associations between young children's developing moral judgments and theory of mind.* Developmental Psychology, 2012. **48**(4): p. 1144-55.
241. Tare, M. and S.A. Gelman, *Can you say it another way? Cognitive factors in bilingual children's pragmatic language skills.* Journal of Cognition and Development, 2010. **11**(2): p. 137-158.
242. Benson, J.E., et al., *Individual differences in executive functioning predict preschoolers' improvement from theory-of-mind training.* Developmental Psychology, 2013. **49**(9): p. 1615-1627.
243. Bigelow, A.E. and K. Dugas, *Relations among preschool children's understanding of visual perspective taking, false belief, and lying.* Journal of Cognition and Development, 2008. **9**(4): p. 411-433.
244. Farrant, B.M., et al., *Empathy, perspective taking and prosocial behaviour: The importance of parenting practices.* Infant and Child Development, 2012. **21**(2): p. 175-188.
245. Nathanson, A.I., et al., *The relation between television exposure and theory of mind among preschoolers.* Journal of Communication, 2013. **63**(6): p. 1088-1108.
246. Brosseau-Liard, P., D. Penney, and D. Poulin-Dubois, *Theory of mind selectively predicts preschoolers' knowledge-based selective word learning.* British Journal of Developmental Psychology, 2015. **33**(4): p. 464-75.
247. Conry-Murray, C., *Young children's understanding of beliefs about moral and conventional rule violations.* Merrill-Palmer Quarterly, 2013. **59**(4): p. 489-510.
248. Bowman, L.C., et al., *Action mechanisms for social cognition: Behavioral and neural correlates of developing theory of mind.* Developmental Science, 2016: p. No Pagination Specified-No Pagination Specified.
249. Kenny, L., E. Hill, and A.F.d.C. Hamilton, *The relationship between social and motor cognition in primary school age-children.* Frontiers in Psychology, 2016. **7**.
250. Simcock, G., et al., *Prenatal maternal stress shapes children's theory of mind: the QF2011 Queensland Flood Study.* Journal of Developmental Origins of Health and Disease, 2017. **8**(4): p. 483-492.
251. Rizzo, M.T. and M. Killen, *Theory of mind is related to children's resource allocations in gender stereotypic contexts.* Developmental Psychology, 2018. **54**(3): p. 510-520.
252. Bennett, M. and L. Galpert, *Children's understanding of multiple desires.* International Journal of Behavioral Development, 1993. **16**(1): p. 15-33.
253. Rakoczy, H., *Executive function and the development of belief desire psychology.* Developmental Science, 2010. **13**(4): p. 648-661.
254. Moore, C., J. Barresi, and C. Thompson, *The cognitive basis of future-oriented prosocial behavior.* Social Development, 1998. **7**(2): p. 198-218.
255. Moore, C., et al., *Conflicting desire and the child's theory of mind.* Cognitive Development, 1995. **10**(4): p. 467-482.
256. Hutchins, T.L., P.A. Prelock, and W. Chace, *Test-retest reliability of a theory of mind task battery for children with Autism Spectrum Disorders.* Focus on Autism and Other Developmental Disabilities, 2008. **23**(4): p. 195-206.
257. Hutchins, T.L., et al., *Beyond false beliefs: the development and psychometric evaluation of the perceptions of children's theory of mind measure-experimental version (PCToMM-E).* Journal of Autism & Developmental Disorders, 2008. **38**(1): p. 143-55.
258. Hutchins, T.L., P.A. Prelock, and L. Bonazinga, *Psychometric evaluation of the Theory of Mind Inventory (ToMI): a study of typically developing children and children with autism spectrum disorder.* Journal of Autism & Developmental Disorders, 2012. **42**(3): p. 327-41.
259. Hutchins, T.L., et al., *Explicit vs. applied theory of mind competence: A comparison of typically developing males, males with ASD, and males with ADHD.* Research in Autism Spectrum Disorders, 2016. **21**: p. 94-108.
260. Lin, S.-K., et al., *Theory of mind predominantly associated with the quality, not quantity, of pretend play in children with autism spectrum disorder.* European Child & Adolescent Psychiatry, 2017: p. No Pagination Specified-No Pagination Specified.
261. Terwogt, M.M. and C. Rieffe, *Stereotyped beliefs about desirability: Implications for characterizing the child's theory of mind.* New Ideas in Psychology, 2003. **21**(1): p. 69-84.

262. Le Sourn-Bissaoui, S. and M. Deleau, *Maternal talks and children's understanding of beliefs and feelings.* Enfance, 2001. **53**(4): p. 329-348.
263. Putko, A., *Developmental differences in the understanding of beliefs and desires.* Polish Psychological Bulletin, 2005. **36**(2): p. 109-115.
264. Hale, C.M. and H. Tager-Flusberg, *Social communication in children with autism: the relationship between theory of mind and discourse development.* Autism, 2005. **9**(2): p. 157-78.
265. Serra, M., et al., *Theory of mind in children with 'lesser variants' of autism: A longitudinal study.* Journal of Child Psychology and Psychiatry, 2002. **43**(7): p. 885-900.
266. Steele, S., R.M. Joseph, and H. Tager-Flusberg, *Brief report: developmental change in theory of mind abilities in children with autism.* Journal of Autism & Developmental Disorders, 2003. **33**(4): p. 461-7.
267. Hoogenhout, M. and S. Malcolm-Smith, *Theory of mind in autism spectrum disorder: Does DSM classification predict development?* Research in Autism Spectrum Disorders, 2014. **8**(6): p. 597-607.
268. Shiverick, S.M. and C.F. Moore, *Fulfilment of intention and desire in children's judgements of emotion for sociomoral events.* British Journal of Developmental Psychology, 2013. **31**(4): p. 395-407.
269. Schult, C.A., *Children's Understanding of the Distinction between Intentions and Desires.* Child Development, 2002. **73**(6): p. 1727-1747.
270. Wellman, H.M. and K. Bartsch, *Young children's reasoning about beliefs.* Cognition, 1988. **30**(3): p. 239-277.
271. Yuill, N., et al., *Children's changing understanding of wicked desires: From objective to subjective and moral.* British Journal of Developmental Psychology, 1996. **14**(4): p. 457-475.
272. Perner, J. and H. Wimmer, *"John thinks that Mary thinks that...": Attribution of second-order beliefs by 5- to 10-year-old children.* Journal of Experimental Child Psychology, 1985. **39**(3): p. 437-471.
273. Colonnesi, C., et al., *Precursors of a theory of mind: A longitudinal study.* British Journal of Developmental Psychology, 2008. **26**(4): p. 561-577.
274. Hahn, L.J., et al., *Early intersubjective skills and the understanding of intentionality in young children with Down syndrome.* Research in Developmental Disabilities, 2013. **34**(12): p. 4455-65.
275. Poulin-Dubois, D. and J. Yott, *Executive functions and theory of mind understanding in young children: A reciprocal relation?* Psychologie Francaise, 2014. **59**(1): p. 59-69.
276. Bellagamba, F., et al., *Re-enactment of intended acts from a video presentation by 18- and 24-month-old children.* Cognitive Processing, 2012. **13**(4): p. 381-6.
277. Camaioni, L., et al., *The Role of Declarative Pointing in Developing a Theory of Mind.* Infancy, 2004. **5**(3): p. 291-308.
278. Carpenter, M., B.F. Pennington, and S.J. Rogers, *Understanding of others' intentions in children with autism.* Journal of Autism & Developmental Disorders, 2001. **31**(6): p. 589-99.
279. Kristen, S., et al., *Infants' joint attention skills predict toddlers' emerging mental state language.* Developmental Psychology, 2011. **47**(5): p. 1207-1219.
280. Meltzoff, A.N., *Understanding the Intentions of Others: Re-Enactment of Intended Acts by 18-Month-Old Children.* Developmental psychology, 1995. **31**(5): p. 838-850.
281. Mull, M.S. and E.M. Evans, *Did she mean to do it? Acquiring a folk theory of intentionality.* Journal of Experimental Child Psychology, 2010. **107**(3): p. 207-28.
282. Fu, G., et al., *Moral judgment and its relation to second-order theory of mind.* Developmental Psychology, 2014. **50**(8): p. 2085-92.
283. Williams, D. and F. Happe, *Representing intentions in self and other: studies of autism and typical development.* Developmental Science, 2010. **13**(2): p. 307-19.
284. Leyva, D., S. Hopson, and A. Nichols, *Reading a note, reading a mind: Children's notating skills and understanding of mind.* Reading and Writing, 2012. **25**(3): p. 701-716.
285. Browne, C.A. and J.D. Woolley, *Theory of mind in children's naming of drawings.* Journal of Cognition and Development, 2001. **2**(4): p. 389-412.
286. Choi, Y.J. and Y. Luo, *13-month-olds' understanding of social interactions.* Psychological Science, 2015. **26**(3): p. 274-83.

287. Killen, M., et al., *The accidental transgressor: morally-relevant theory of mind.* Cognition, 2011. **119**(2): p. 197-215.
288. Li, L., et al., *Theory of Mind and Resource Allocation in the Context of Hidden Inequality.* Cognitive Development, 2017. **43**: p. 25-36.
289. Russell, J., E.L. Hill, and F. Franco, *The role of belief veracity in understanding intentions-in-action. Preschool children's performance on the transparent intentions task.* Cognitive Development, 2001. **16**(3): p. 775-792.
290. Gattegno, M., et al., *Preliminary study about specific relationship between joint attention and "theory of mind" in infantile autism.* A N A E Approche Neuropsychologique des Apprentissages chez l'Enfant, 1999. **11**(2; 52): p. 42-48.
291. Baron-Cohen, S., A.M. Leslie, and U. Frith, *Mechanical, behavioural and Intentional understanding of picture stories in autistic children.* British Journal of Developmental Psychology, 1986. **4**(2): p. 113-125.
292. Andres-Roqueta, C., et al., *Social cognition makes an independent contribution to peer relations in children with Specific Language Impairment.* Research in Developmental Disabilities, 2016. **49-50**: p. 277-90.
293. Atkinson, L., et al., *Theory of mind in emerging reading comprehension: A longitudinal study of early indirect and direct effects.* Journal of Experimental Child Psychology, 2017: p. No Pagination Specified-No Pagination Specified.
294. Tsuji, H. and P. Mitchell, *Modelling the executive components involved in processing false belief and mechanical/intentional sequences.* British Journal of Developmental Psychology, 2019. **37**(2): p. 184-198.
295. Santos, A. and C. Deruelle, *Verbal peaks and visual valleys in Theory of Mind Ability in Williams syndrome.* Journal of Autism and Developmental Disorders, 2009. **39**(4): p. 651-659.
296. Buresh, J.S. and A.L. Woodward, *Infants track action goals within and across agents.* Cognition, 2007. **104**(2): p. 287-314.
297. Phillips, A.T. and H.M. Wellman, *Infants' understanding of object-directed action.* Cognition, 2005. **98**(2): p. 137-155.
298. Phillips, A.T., H.M. Wellman, and E.S. Spelke, *Infants' ability to connect gaze and emotional expression to intentional action.* Cognition, 2002. **85**(1): p. 53-78.
299. Gergely, G., et al., *Taking the intentional stance at 12 months of age.* Cognition, 1995. **56**(2): p. 165-93.
300. Smiley, P.A., *Intention understanding and parter-sensitive behaviors in young children's peer interactions.* Social Development, 2001. **10**(3): p. 330-354.
301. Bernier, A., et al., *My mother is sensitive, but I am too tired to know: infant sleep as a moderator of prospective relations between maternal sensitivity and infant outcomes.* Infant Behavior & Development, 2014. **37**(4): p. 682-94.
302. Tahiroglu, D., et al., *The Children's Social Understanding Scale: construction and validation of a parent-report measure for assessing individual differences in children's theories of mind.* Developmental Psychology, 2014. **50**(11): p. 2485-97.
303. Chasiotis, A., et al., *Theory of mind and inhibitory control in three cultures: Conflict inhibition predicts false belief understanding in Germany, Costa Rica and Cameroon.* International Journal of Behavioral Development, 2006. **30**(3): p. 249-260.
304. Thirion-Marissiaux, A.F. and N. Nader-Grosbois, *Theory of mind "beliefs", developmental characteristics and social understanding in children and adolescents with intellectual disabilities.* Research in Developmental Disabilities, 2008. **29**(6): p. 547-66.
305. Moll, H. and M. Tomasello, *Level 1 perspective-taking at 24 months of age.* British Journal of Developmental Psychology, 2006. **24**(3): p. 603-613.
306. Melinder, A., T. Endestad, and S. Magnussen, *Relations between episodic memory, suggestibility, theory of mind, and cognitive inhibition in the preschool child.* Scandinavian Journal of Psychology, 2006. **47**(6): p. 485-95.
307. Taumoepeau, M. and E. Reese, *Understanding the self through siblings: Self-awareness mediates the sibling effect on social understanding.* Social Development, 2014. **23**(1): p. 1-18.
308. Hughes, C., et al., *Social behaviour in pervasive developmental disorders: effects of informant, group and "theory-of-mind".* European Child & Adolescent Psychiatry, 1997. **6**(4): p. 191-8.
309. de, C., R.M. Brindley, and U. Frith, *Imitation and action understanding in autistic spectrum disorders: How valid is the hypothesis of a deficit in the mirror neuron system?* Neuropsychologia, 2007. **45**(8): p. 1859-1868.

310. Baron-Cohen, S., *Out of sight or out of mind? Another look at deception in autism.* Journal of Child Psychology & Psychiatry & Allied Disciplines, 1992. **33**(7): p. 1141-55.
311. Hughes, C. and R. Ensor, *Executive function and theory of mind in 2 year olds: a family affair?* Developmental Neuropsychology, 2005. **28**(2): p. 645-68.
312. Hughes, C. and R. Ensor, *Behavioural problems in 2-year-olds: links with individual differences in theory of mind, executive function and harsh parenting.* Journal of Child Psychology & Psychiatry & Allied Disciplines, 2006. **47**(5): p. 488-97.
313. Hughes, C. and R. Ensor, *Positive and protective: effects of early theory of mind on problem behaviors in at-risk preschoolers.* Journal of Child Psychology & Psychiatry & Allied Disciplines, 2007. **48**(10): p. 1025-32.
314. Hughes, C. and R. Ensor, *Executive function and theory of mind: Predictive relations from ages 2 to 4.* Developmental Psychology, 2007. **43**(6): p. 1447-1459.
315. Hughes, C. and R. Ensor, *Does executive function matter for preschoolers' problem behaviors?* Journal of Abnormal Child Psychology, 2008. **36**(1): p. 1-14.
316. Hughes, C., et al., *Cooperation and conversations about the mind: A study of individual differences in 2-year-olds and their siblings.* British Journal of Developmental Psychology, 2006. **24**(1): p. 53-72.
317. Chasiotis, A., et al., *Sensory motor inhibition as a prerequisite for theory-of-mind: A comparison of clinical and normal preschoolers differing in sensory motor abilities.* International Journal of Behavioral Development, 2006. **30**(2): p. 178-190.
318. Peterson, C.C., J.L. Peterson, and J. Webb, *Factors influencing the development of a theory of mind in blind children.* British Journal of Developmental Psychology, 2000. **18**(3): p. 431-447.
319. Dalke, D.E., *Explaining young children's difficulty on the false belief task: Representational deficits or context-sensitive knowledge?* British Journal of Developmental Psychology, 1995. **13**(3): p. 209-222.
320. Marcovitch, S., et al., *A longitudinal assessment of the relation between executive function and theory of mind at 3, 4, and 5 years.* Cognitive Development, 2015. **33**: p. 40-55.
321. Charman, T., et al., *Testing joint attention, imitation, and play as infancy precursors to language and theory of mind.* Cognitive Development, 2000. **15**(4): p. 481-498.
322. Flavell, J.H., et al., *Young children's knowledge about visual perception: Further evidence for the Level 1–Level 2 distinction.* Developmental Psychology, 1981. **17**(1): p. 99-103.
323. Masangkay, Z.S., et al., *The Early Development of Inferences about the Visual Percepts of Others.* Child Development, 1974. **45**(2): p. 357-366.
324. Montoya-Rodríguez, M.M. and F.J. Molina Cobos, *Relationship between deictic relational responding and theory of mind tasks in children: A pilot study.* The Psychological Record, 2016. **66**(4): p. 573-587.
325. Gonzales, C.R., W.V. Fabricius, and A.S. Kupfer, *Introspection Plays an Early Role in Children's Explicit Theory of Mind Development.* Child Development, 2018. **89**(5): p. 1545-1552.
326. Flavell, J.H., S.G. Shipstead, and K. Croft, *Young Children's Knowledge about Visual Perception: Hiding Objects from Others.* Child Development, 1978. **49**(4): p. 1208-1211.
327. McGuigan, N. and M.J. Doherty, *The relation between hiding skill and judgment of eye direction in preschool children.* Developmental Psychology, 2002. **38**(3): p. 418-427.
328. Gonzales, C.R., W.V. Fabricius, and A.S. Kupfer, *Introspection plays an early role in children's explicit theory of mind development.* Child Development, 2017: p. No Pagination Specified-No Pagination Specified.
329. Hughes, C., K. Deater-Deckard, and A.L. Cutting, *"Speak roughly to your little boy?" Sex differences in the relations between parenting and preschoolers' understanding of mind.* Social Development, 1999. **8**(2): p. 143-160.
330. Hughes, C., S. Lecce, and C. Wilson, *"Do you know what I want?" Preschoolers' talk about desires, thoughts and feelings in their conversations with sibs and friends.* Cognition and Emotion, 2007. **21**(2): p. 330-350.

331. Lewis-Morrarty, E., et al., *Cognitive flexibility and theory of mind outcomes among foster children: preschool follow-up results of a randomized clinical trial.* Journal of Adolescent Health, 2012. **51**(2 Suppl): p. S17-22.
332. Flynn, E., *A microgenetic investigation of stability and continuity in theory of mind development.* British Journal of Developmental Psychology, 2006. **24**(3): p. 631-654.
333. Gratch, G., *RESPONSE ALTERNATION IN CHILDREN: A DEVELOPMENTAL STUDY OF ORIENTATIONS TO UNCERTAINTY.* Vita Hum Int Z Lebensalterforsch, 1964. **7**: p. 49-60.
334. Hughes, M. and M. Donaldson, *The use of hinding games for studying the coordination of viewpoints.* Educational Review, 1979. **31**(2): p. 133-140.
335. Miller, S.A., et al., *Children's understanding of false beliefs that result from developmental misconceptions.* Cognitive Development, 1997. **12**(1): p. 21-51.
336. Van Reet, J., K.F. Green, and D.M. Sobel, *Preschoolers' theory-of-mind knowledge influences whom they trust about others' theories of mind.* Journal of Cognition and Development, 2015. **16**(3): p. 471-491.
337. Flavell, J.H., F.L. Green, and E.R. Flavell, *Development of knowledge about the appearance-reality distinction.* Monogr Soc Res Child Dev, 1986. **51**(1): p. i-v, 1-87.
338. Meltzoff, A.N. and R. Brooks, *Self-experience as a mechanism for learning about others: A training study in social cognition.* Developmental Psychology, 2008. **44**(5): p. 1257-1265.
339. Herold, K.H. and N. Akhtar, *Imitative learning from a third-party interaction: relations with self-recognition and perspective taking.* Journal of Experimental Child Psychology, 2008. **101**(2): p. 114-23.
340. Peisach, E. and M. Hardeman, *Imaginative play and logical thinking in young children.* The Journal of Genetic Psychology: Research and Theory on Human Development, 1985. **146**(2): p. 233-248.
341. Peskin, J. and V. Ardino, *Representing the Mental World in Children's Social Behavior: Playing Hide-and-Seek and Keeping a Secret.* Social Development, 2003. **12**(4): p. 496-512.
342. Ricard, M., P.C. Girouard, and T.G. Decarie, *Personal pronouns and perspective taking in toddlers.* Journal of Child Language, 1999. **26**(3): p. 681-97.
343. Russell, J., D. Alexis, and N. Clayton, *Episodic future thinking in 3- to 5-year-old children: the ability to think of what will be needed from a different point of view.* Cognition, 2010. **114**(1): p. 56-71.
344. Sodian, B., C. Thoermer, and U. Metz, *Now I see it but you don't: 14-month-olds can represent another person's visual perspective.* Developmental Science, 2007. **10**(2): p. 199-204.
345. Ebersbach, M., S. Stiehler, and P. Asmus, *On the relationship between children's perspective taking in complex scenes and their spatial drawing ability.* British Journal of Developmental Psychology, 2011. **29**(Pt 3): p. 455-74.
346. Broadbent, H.J., E.K. Farran, and A. Tolmie, *Object-based mental rotation and visual perspective-taking in typical development and Williams syndrome.* Developmental Neuropsychology, 2014. **39**(3): p. 205-25.
347. Hirai, M., et al., *Developmental changes in mental rotation ability and visual perspective-taking in children and adults with Williams syndrome.* Frontiers in Human Neuroscience, 2013. **7**: p. 856.
348. Epley, N., C.K. Morewedge, and B. Keysar, *Perspective taking in children and adults: Equivalent egocentrism but differential correction.* Journal of Experimental Social Psychology, 2004. **40**(6): p. 760-768.
349. Cannella, G.S., R. Viruru, and S. Amin, *Sociocognitive growth and the young child: Comparisons with spatial and literacy content.* Child Study Journal, 1995. **25**(3): p. 213-231.
350. Frick, A., W. Mohring, and N.S. Newcombe, *Picturing perspectives: development of perspective-taking abilities in 4- to 8-year-olds.* Frontiers in Psychology, 2014. **5**: p. 386.
351. Nilsen, E.S. and S.A. Graham, *The relations between children's communicative perspective-taking and executive functioning.* Cognitive Psychology, 2009. **58**(2): p. 220-49.

352. Nadig, A.S. and J.C. Sedivy, *Evidence of Perspective-Taking Constraints in Children's On-Line Reference Resolution.* Psychological Science, 2002. **13**(4): p. 329-336.
353. Newcombe, N. and J. Huttenlocher, *Children's Early Ability to Solve Perspective-Taking Problems*. Vol. 28. 1992. 635-643.
354. Burns, P., J. Russell, and C. Russell, *Children's binding of what-where-when-who in episodic memory: Identifying oneself in the past.* Journal of Cognition and Development, 2016. **17**(2): p. 300-319.
355. Rosser, R.A., K. Chandler, and S. Lane, *Children's computation of viewpoint from locational descriptions: Initial steps in the coordination of perspectives.* Child Study Journal, 1993. **23**(1): p. 1-16.
356. Williamson, R.A., R. Brooks, and A.N. Meltzoff, *The sound of social cognition: Toddlers' understanding of how sound influences others.* Journal of Cognition and Development, 2015. **16**(2): p. 252-260.
357. Aronson, J.N. and C. Golomb, *Preschoolers' understanding of pretense and presumption of congruity between action and representation.* Developmental Psychology, 1999. **35**(6): p. 1414-25.
358. Lillard, A.S., *Young Children's Conceptualization of Pretense: Action or Mental Representational State?* Child Development, 1993. **64**(2): p. 372-386.
359. Sobel, D.M. and S.M. Letourneau, *Children's Developing Descriptions and Judgments of Pretending.* Child Development, 2019. **90**(5): p. 1817-1831.
360. Abrams, D., et al., *Children's group nous: understanding and applying peer exclusion within and between groups.* Child Development, 2009. **80**(1): p. 224-43.
361. Zhao, J., et al., *3 to 5 years old children's behavioral and verbal performances in level 1 perspective-taking.* Acta Psychologica Sinica, 2010. **42**(7): p. 754-767.
362. Wang, Z., X.C. Wang, and W.Y. Chui, *Young Children's Understanding of Teaching and Learning and Their Theory of Mind Development: A Causal Analysis from a Cross-Cultural Perspective.* Frontiers in Psychology, 2017. **8**: p. 725.
363. Schick, B., et al., *Language and theory of mind: A study of deaf children.* Child Development, 2007. **78**(2): p. 376-396.
364. Povinelli, D.J. and S. DeBlois, *Young children's (Homo sapiens) understanding of knowledge formation in themselves and others.* Journal of Comparative Psychology, 1992. **106**(3): p. 228-238.
365. Ebert, S., *Longitudinal relations between theory of mind and metacognition and the impact of language.* Journal of Cognition and Development, 2015. **16**(4): p. 559-586.
366. Lockl, K., S. Ebert, and S. Weinert, *Predicting school achievement from early theory of mind: Differential effects on achievement tests and teacher ratings.* Learning and Individual Differences, 2017. **53**: p. 93-102.
367. Viranyi, Z., et al., *A nonverbal test of knowledge attribution: A comparative study on dogs and children.* Animal Cognition, 2006. **9**(1): p. 13-26.
368. Das, S. and N. Babu, *Children's Acquisition of a Theory of Mind: The Role of Presence vs Absence of Sibling.* Psychological Studies, 2004. **49**(1): p. 36-44.
369. Chu, C.Y. and U. Minai, *Children's Demonstrative Comprehension and the Role of Non-linguistic Cognitive Abilities: A Cross-Linguistic Study.* Journal of Psycholinguistic Research, 2018. **47**(6): p. 1343-1368.
370. Patnaik, B. and N. Babu, *Relationship between children's acquisition of a theory of a mind and their understanding of mental terms.* Psycho-Lingua, 2001. **31**(1): p. 3-8.
371. Resches, M. and M. Perez Pereira, *Referential communication abilities and Theory of Mind development in preschool children.* Journal of Child Language, 2007. **34**(1): p. 21-52.
372. Lane, J.D., et al., *Relations between temperament and theory of mind development in the United States and China: biological and behavioral correlates of preschoolers' false-belief understanding.* Developmental Psychology, 2013. **49**(5): p. 825-36.
373. Lind, S.E. and D.M. Bowler, *Impaired performance on see-know tasks amongst children with autism: evidence of specific difficulties with theory of mind or domain-general task factors?* Journal of Autism & Developmental Disorders, 2010. **40**(4): p. 479-84.
374. Reed, T., *Performance of autistic and control subjects on three cognitive perspective-taking tasks.* Journal of Autism & Developmental Disorders, 1994. **24**(1): p. 53-66.

375. Ziv, Y., B.S. Hadad, and Y. Khateeb, *Social information processing in preschool children diagnosed with autism spectrum disorder.* Journal of Autism and Developmental Disorders, 2014. **44**(4): p. 846-859.
376. Birch, S.A. and P. Bloom, *Children are cursed: an asymmetric bias in mental-state attribution.* Psychological Science, 2003. **14**(3): p. 283-6.
377. Bright-Paul, A., C. Jarrold, and D.B. Wright, *Theory-of-mind development influences suggestibility and source monitoring.* Developmental Psychology, 2008. **44**(4): p. 1055-1068.
378. De Cat, C., *Egocentric definiteness errors and perspective evaluation in preschool children.* Journal of Pragmatics, 2013. **56**: p. 58-69.
379. O'Neill, D.K. and A. Gopnik, *Young children's ability to identify the sources of their beliefs.* Developmental Psychology, 1991. **27**(3): p. 390-397.
380. Welch-Ross, M.K., *Interviewer knowledge and preschoolers' reasoning about knowledge states moderate suggestibility.* Cognitive Development, 1999. **14**(3): p. 423-442.
381. Lackner, C.L., L.C. Bowman, and M.A. Sabbagh, *Dopaminergic functioning and preschoolers' theory of mind.* Neuropsychologia, 2010. **48**(6): p. 1767-74.
382. Welch-Ross, M.K., *Preschoolers' understanding of mind: Implications for suggestibility.* Cognitive Development, 1999. **14**(1): p. 101-131.
383. Ruffman, T. and D. Olson, *Children's ascriptions of knowledge to others.* Developmental Psychology, 1989. **25**(4): p. 601-606.
384. Pillow, B.H., *Early understanding of perception as a source of knowledge.* Journal of Experimental Child Psychology, 1989. **47**(1): p. 116-129.
385. Meins, E., et al., *Maternal mind-mindedness and children's behavioral difficulties: mitigating the impact of low socioeconomic status.* Journal of Abnormal Child Psychology, 2013. **41**(4): p. 543-53.
386. Suway, J.G., et al., *The relations among theory of mind, behavioral inhibition, and peer interactions in early childhood.* Social Development, 2012. **21**(2): p. 331-342.
387. Qu, L., et al., *Teachers' theory-of-mind coaching and children's executive function predict the training effect of sociodramatic play on children's theory of mind.* Social Development, 2015: p. No Pagination Specified.
388. Guajardo, N.R., R. Petersen, and T.R. Marshall, *The roles of explanation and feedback in false belief understanding: a microgenetic analysis.* Journal of Genetic Psychology, 2013. **174**(3): p. 225-52.
389. Larkin, F., et al., *How does restricted and repetitive behavior relate to language and cognition in typical development?* Development and Psychopathology, 2016: p. No Pagination Specified-No Pagination Specified.
390. Weil, T.M., S.C. Hayes, and P. Capurro, *Establishing a deictic relational repertoire in young children.* The Psychological Record, 2011. **61**(3): p. 371-390.
391. Fedra, E. and M.F.H. Schmidt, *Older (but not younger) preschoolers reject incorrect knowledge claims.* British Journal of Developmental Psychology, 2019. **37**(1): p. 130-145.
392. Sodian, B. and H. Wimmer, *Children's Understanding of Inference as a Source of Knowledge.* Child Development, 1987. **58**(2): p. 424-433.
393. Taumoepeau, M. and E. Reese, *Maternal reminiscing, elaborative talk, and children's theory of mind: An intervention study.* First Language, 2013. **33**(4): p. 388-410.
394. Wimmer, H., et al., *Children's Understanding of Informational Access as Source of Knowledge.* Child Development, 1988. **59**(2): p. 386-396.
395. Denham, S.A., *Social cognition, prosocial behavior, and emotion in preschoolers: Contextual validation.* Child Development, 1986. **57**(1): p. 194-201.
396. Yagmurlu, B., S.K. Berument, and S. Celimli, *The role of institution and home contexts in theory of mind development.* Journal of Applied Developmental Psychology, 2005. **26**(5): p. 521-537.
397. Kamawar, D. and D.R. Olson, *Children's understanding of referentially opaque contexts: The role of metarepresentational and metalinguistic ability.* Journal of Cognition and Development, 2009. **10**(4): p. 285-305.
398. Kamawar, D. and D.R. Olson, *Thinking about representations: the case of opaque contexts.* Journal of Experimental Child Psychology, 2011. **108**(4): p. 734-46.
399. Kovacs, A.M., *Early bilingualism enhances mechanisms of false-belief reasoning.* Developmental Science, 2009. **12**(1): p. 48-54.
400. Perner, J., T. Ruffman, and S.R. Leekam, *Theory of Mind Is Contagious: You Catch It from Your Sibs.* Child Development, 1994. **65**(4): p. 1228-1238.

401. Peskin, J., C. Prusky, and J. Comay, *Keeping the reader's mind in mind: Development of perspective-taking in children's dictations.* Journal of Applied Developmental Psychology, 2014. **35**(1): p. 35-43.
402. Moll, H., et al., *Infants Determine Others' Focus of Attention by Pragmatics and Exclusion.* Journal of Cognition and Development, 2006. **7**(3): p. 411-430.
403. Crivello, C., S. Phillips, and D. Poulin-Dubois, *Selective social learning in infancy: looking for mechanisms.* Developmental Science, 2018. **21**(3): p. e12592.
404. Moll, H. and M. Tomasello, *How 14- and 18-month-olds know what others have experienced.* Dev Psychol, 2007. **43**(2): p. 309-17.
405. Ensor, R., et al., *Mothers' cognitive references to 2-year-olds predict theory of mind at ages 6 and 10.* Child Development, 2014. **85**(3): p. 1222-1235.
406. Schacht, R., et al., *The relation between mind-mindedness in mothers with borderline personality disorder and mental state understanding in their children.* Infant and Child Development, 2013. **22**(1): p. 68-84.
407. Brown, J.R., N. Donelan-McCall, and J. Dunn, *Why talk about mental states? The significance of children's conversations with friends, siblings, and mothers.* Child Development, 1996. **67**(3): p. 836-49.
408. Guajardo, N.R., J. Parker, and K. Turley-Ames, *Associations among false belief understanding, counterfactual reasoning, and executive function.* British Journal of Developmental Psychology, 2009. **27**(Pt 3): p. 681-702.
409. Curenton, S.M., *Low-income preschoolers' false-belief performance.* Journal of Genetic Psychology, 2003. **164**(4): p. 411-24.
410. Olson, S.L., et al., *Individual differences in the development of early peer aggression: integrating contributions of self-regulation, theory of mind, and parenting.* Development & Psychopathology, 2011. **23**(1): p. 253-66.
411. Deleau, M., et al., *Conversational awareness and theory of mind.* Enfance, 1999. **51**(3): p. 238-247.
412. Hughes, C., R. Ensor, and A. Marks, *Individual differences in false belief understanding are stable from 3 to 6 years of age and predict children's mental state talk with school friends.* Journal of Experimental Child Psychology, 2010. **108**(1): p. 96-112.
413. Hughes, C., *Finding your marbles: does preschoolers' strategic behavior predict later understanding of mind?* Developmental Psychology, 1998. **34**(6): p. 1326-39.
414. Hwa-Froelich, D.A., H. Matsuo, and K. Jacobs, *False Belief Performance of Children Adopted Internationally.* American Journal of Speech-Language Pathology, 2017. **26**(1): p. 29-43.
415. Olson, S.L., D.E. Choe, and A.J. Sameroff, *Trajectories of child externalizing problems between ages 3 and 10 years: Contributions of children's early effortful control, theory of mind, and parenting experiences.* Development and Psychopathology, 2017: p. No Pagination Specified-No Pagination Specified.
416. Guajardo, N.R. and K.B. Cartwright, *The contribution of theory of mind, counterfactual reasoning, and executive function to pre-readers' language comprehension and later reading awareness and comprehension in elementary school.* Journal of Experimental Child Psychology, 2016. **144**: p. 27-45.
417. Galende, N., M.S. de Miguel, and E. Arranz, *The role of physical context, verbal skills, non-parental care, social support, and type of parental discipline in the development of ToM capacity in five-year-old children.* Social Development, 2011. **20**(4): p. 845-861.
418. Galende, N., M.S. de Miguel, and E. Arranz, *The role of parents' distancing strategies in the development of five-year-old children's Theory of Mind.* Early Child Development and Care, 2012. **182**(2): p. 207-220.
419. Lewis, C., Z. Huang, and M. Rooksby, *Chinese preschoolers' false belief understanding: Is social knowledge underpinned by parental styles, social interactions or executive functions?* Psychologia: An International Journal of Psychology in the Orient, 2006. **49**(4): p. 252-266.
420. Lucas, A.J., et al., *Social-cognitive processes in preschoolers' selective trust: Three cultures compared.* Developmental Psychology, 2013. **49**(3): p. 579-590.
421. Cheung, H., H.C. Chen, and W. Yeung, *Relations between mental verb and false belief understanding in Cantonese-speaking children.* Journal of Experimental Child Psychology, 2009. **104**(2): p. 141-55.
422. Lee, K., D.R. Olson, and N. Torrance, *Chinese children's understanding of false beliefs: the role of language.* Journal of Child Language, 1999. **26**(1): p. 1-21.
423. Brambring, M. and D. Asbrock, *Validity of false belief tasks in blind children.* Journal of Autism and Developmental Disorders, 2010. **40**(12): p. 1471-1484.
424. Coughlin, C., et al., *Introspection on uncertainty and judicious help-seeking during the preschool years.* Developmental Science, 2015. **18**(6): p. 957-71.

425. Moeller, M.P. and B. Schick, *Relations Between Maternal Input and Theory of Mind Understanding in Deaf Children.* Child Development, 2006. **77**(3): p. 751-766.
426. Jackson, A., *Language facility and theory of mind development in deaf children.* Journal of Deaf Studies and Deaf Education, 2001. **6**(3): p. 161-176.
427. Lundy, J.E., *Age and language skills of deaf children in relation to theory of mind development.* Journal of Deaf Studies and Deaf Education, 2002. **7**(1): p. 41-56.
428. Jones, A.C., R. Gutierrez, and A.K. Ludlow, *Confronting the language barrier: Theory of mind in deaf children.* Journal of Communication Disorders, 2015. **56**: p. 47-58.
429. De Gracia, M.R.L., C.C. Peterson, and M. de Rosnay, *A cultural conundrum: Delayed false-belief understanding in Filipino children.* Journal of Cross-Cultural Psychology, 2016. **47**(7): p. 929-940.
430. Nguyen, T.-K. and J.W. Astington, *Reassessing the bilingual advantage in theory of mind and its cognitive underpinnings.* Bilingualism: Language and Cognition, 2014. **17**(2): p. 396-409.
431. Deneault, J. and M. Ricard, *Are emotion and mind understanding differently linked to young children's social adjustment? Relationships between behavioral consequences of emotions, false belief, and SCBE.* Journal of Genetic Psychology, 2013. **174**(1): p. 88-116.
432. Adrien, J., et al., *Development and functioning of a "theory of mind" in autistic and normal children.* A N A E Approche Neuropsychologique des Apprentissages chez l'Enfant, 1995. **7**(5; 35): p. 188-196.
433. Caillies, S. and S. Le Sourn-Bissaoui, *Children's understanding of idioms and theory of mind development.* Developmental Science, 2008. **11**(5): p. 703-11.
434. Bernard, S., M. Deleau, and G. Rouxel, *Language and beliefs attribution in children: The role of conversational perspective-taking.* Archives de Psychologie, 2008. **73**(286-287): p. 167-186.
435. Renouf, A., et al., *Relations between theory of mind and indirect and physical aggression in kindergarten: Evidence of the moderating role of prosocial behaviors.* Social Development, 2010. **19**(3): p. 535-555.
436. Comte-Gervais, I., et al., *Assessment of social intelligence in children with specific language impairment: Presentation of an assessing scale.* L'Evolution Psychiatrique, 2008. **73**(2): p. 353-366.
437. Bernard, S. and M. Deleau, *Conversational perspective-taking and false belief attribution: A longitudinal study.* British Journal of Developmental Psychology, 2007. **25**(3): p. 443-460.
438. Sodian, B., C. Hulsken, and C. Thormer, *Young children's understanding of deception.* Enfance, 1999. **51**(3): p. 215-224.
439. Lohmann, H. and M. Tomasello, *The Role of Language in the Development of False Belief Understanding: A Training Study.* Child Development, 2003. **74**(4): p. 1130-1144.
440. Perner, J. and R. Horn, *Ignorance or False Negatives: Do Children of 4 to 5 Years Simulate Belief With "Not Knowing = Getting it Wrong?".* Journal of Cognition and Development, 2003. **4**(3): p. 263-273.
441. Hogrefe, G.-J., H. Wimmer, and J. Perner, *Ignorance versus False Belief: A Developmental Lag in Attribution of Epistemic States.* Child Development, 1986. **57**(3): p. 567-582.
442. Sodian, B. and S. Kristen-Antonow, *Declarative joint attention as a foundation of theory of mind.* Developmental Psychology, 2015. **51**(9): p. 1190-200.
443. Piekny, J., D. Grube, and C. Maehler, *The relation between preschool children's false-belief understanding and domain-general experimentation skills.* Metacognition and Learning, 2013. **8**(2): p. 103-119.
444. Licata, M., S. Kristen, and B. Sodian, *Mother–child interaction as a cradle of theory of mind: The role of maternal emotional availability.* Social Development, 2016. **25**(1): p. 139-156.
445. Lewis, C., et al., *Social influences on false belief access: specific sibling influences or general apprenticeship?* Child Development, 1996. **67**(6): p. 2930-47.
446. Maridaki-Kassotaki, K., *Greek children's understanding of false beliefs: The role of language.* Psychology: The Journal of the Hellenic Psychological Society, 2005. **12**(1): p. 62-69.

447. Maridaki-Kassotaki, K. and K. Antonopoulou, *Examination of the relationship between false-belief understanding and referential communication skills.* European Journal of Psychology of Education, 2011. **26**(1): p. 75-84.
448. Shamir, A., Z.R. Mevarech, and C. Gida, *The assessment of meta-cognition in different contexts: Individualized vs. peer assisted learning.* Metacognition and Learning, 2009. **4**(1): p. 47-61.
449. Kimhi, Y., et al., *Theory of mind and executive function in preschoolers with typical development versus intellectually able preschoolers with autism spectrum disorder.* Journal of Autism & Developmental Disorders, 2014. **44**(9): p. 2341-54.
450. Meir, N. and R. Novogrodsky, *Prerequisites of Third-Person Pronoun Use in Monolingual and Bilingual Children With Autism and Typical Language Development.* Frontiers in Psychology, 2019. **10**: p. 2289.
451. Lecce, S., M. Caputi, and A. Pagnin, *False-belief understanding at age 5 predicts beliefs about learning in year 3 of primary school.* European Journal of Developmental Psychology, 2015. **12**(1): p. 40-53.
452. Bellagamba, F., et al., *False belief understanding and "cool" inhibitory control in 3-and 4-years-old Italian children.* Frontiers in Psychology, 2015. **6**: p. 872.
453. Di Michele, V., et al., *Deficits in pragmatic conversation as manifestation of genetic liability in autism.* Clinical Neuropsychiatry: Journal of Treatment Evaluation, 2007. **4**(4): p. 144-151.
454. Ornaghi, V., J. Brockmeier, and I.G. Gavazzi, *The role of language games in children's understanding of mental states: A training study.* Journal of Cognition and Development, 2011. **12**(2): p. 239-259.
455. Mizokawa, A., *Association Between Children's Theory of Mind and Responses to Insincere Praise Following Failure.* Frontiers in Psychology, 2018. **9**: p. 1684.
456. Oh, S. and C. Lewis, *Korean preschoolers' advanced inhibitory control and its relation to other executive skills and mental state understanding.* Child Development, 2008. **79**(1): p. 80-99.
457. Vinden, P.G., *Parenting attitudes and children's understanding of mind. A comparison of Korean American and Anglo-American families.* Cognitive Development, 2001. **16**(3): p. 793-809.
458. Farrar, M., et al., *Language and false belief in Korean-speaking and English-speaking children.* Cognitive Development, 2013. **28**(3): p. 209-221.
459. Evans, A.D., F. Xu, and K. Lee, *When all signs point to you: Lies told in the face of evidence.* Developmental Psychology, 2011. **47**(1): p. 39-49.
460. Wang, B., et al., *Chinese preschoolers' implicit and explicit false-belief understanding.* British Journal of Developmental Psychology, 2012. **30**(Pt 1): p. 123-40.
461. Lu, H., Y. Su, and Q. Wang, *Talking about others facilitates theory of mind in Chinese preschoolers.* Developmental Psychology, 2008. **44**(6): p. 1726-1736.
462. Guiberson, M. and B.L. Rodriguez, *False belief understanding in language impaired and typically developing Spanish-speaking preschoolers from lower income backgrounds.* Early Education and Development, 2013. **24**(4): p. 517-535.
463. Andres-Roqueta, C., et al., *Which are the best predictors of theory of mind delay in children with specific language impairment?* International Journal of Language & Communication Disorders, 2013. **48**(6): p. 726-37.
464. Diaz, V. and M.J. Farrar, *The missing explanation of the false-belief advantage in bilingual children: a longitudinal study.* Developmental Science, 2018. **21**(4): p. e12594.
465. Keceli Kaysili, B. and F. Acarlar, *The development of theory of mind according to false belief performance of children ages 3 to 5.* Kuram ve Uygulamada Egitim Bilimleri, 2011. **11**(4): p. 1821-1826.
466. Yagmurlu, B., A. Sanson, and S. Koymen, *Effects of parenting and child temperament on the development of prosocial behavior: The mediating role of theory of mind.* Turk Psikoloji Dergisi, 2005. **20**(55): p. 1-24.
467. Yagmurlu, B., *Relations among sociocognitive abilities and prosocial behavior.* Journal of Child and Family Studies, 2014. **23**(3): p. 591-603.
468. Uzundag, B.A. and A.C. Kuntay, *Children's referential communication skills: The role of cognitive abilities and adult models of speech.* Journal of Experimental Child Psychology, 2018. **172**: p. 73-95.

469. Oberle, E., *The development of Theory of Mind reasoning in Micronesian children.* Journal of Cognition and Culture, 2009. **9**(1-2): p. 39-56.
470. Renouf, A., et al., *Interactive links between theory of mind, peer victimization, and reactive and proactive aggression.* Journal of Abnormal Child Psychology, 2010. **38**(8): p. 1109-23.
471. Yang, J., et al., *The relationship between theory of mind and executive function in a sample of children from mainland China.* Child Psychiatry and Human Development, 2009. **40**(2): p. 169-182.
472. Castelli, F., *The Valley task: Understanding intention from goal-directed motion in typical development and autism.* British Journal of Developmental Psychology, 2006. **24**(4): p. 655-668.
473. Lam, Y.G. and S.-s.S. Yeung, *Cognitive deficits and symbolic play in preschoolers with autism.* Research in Autism Spectrum Disorders, 2012. **6**(1): p. 560-564.
474. Pellicano, E., et al., *Multiple cognitive capabilities/deficits in children with an autism spectrum disorder: "Weak" central coherence and its relationship to theory of mind and executive control.* Development and Psychopathology, 2006. **18**(1): p. 77-98.
475. Pellicano, E., *The development of core cognitive skills in autism: a 3-year prospective study.* Child Development, 2010. **81**(5): p. 1400-16.
476. Pellicano, E., *Individual differences in executive function and central coherence predict developmental changes in theory of mind in autism.* Developmental Psychology, 2010. **46**(2): p. 530-44.
477. Pellicano, E., *Links between theory of mind and executive function in young children with autism: clues to developmental primacy.* Developmental Psychology, 2007. **43**(4): p. 974-90.
478. Peterson, C.C., et al., *Everyday social and conversation applications of theory-of-mind understanding by children with autism-spectrum disorders or typical development.* European Child & Adolescent Psychiatry, 2009. **18**(2): p. 105-15.
479. Peterson, C.C., V.P. Slaughter, and J. Paynter, *Social maturity and theory of mind in typically developing children and those on the autism spectrum.* Journal of Child Psychology & Psychiatry & Allied Disciplines, 2007. **48**(12): p. 1243-50.
480. Capps, L., M. Losh, and C. Thurber, *"The frog ate the bug and made his mouth sad": narrative competence in children with autism.* Journal of Abnormal Child Psychology, 2000. **28**(2): p. 193-204.
481. Dissanayake, C., J. Shembrey, and T. Suddendorf, *Delayed video self-recognition in children with high functioning autism and Asperger's disorder.* Autism, 2010. **14**(5): p. 495-508.
482. Swettenham, J., *Can children be taught to understand false belief using computers?* Child Psychology & Psychiatry & Allied Disciplines, 1996. **37**(2): p. 157-165.
483. Frith, U., F. Happe, and F. Siddons, *Autism and theory of mind in everyday life.* Social Development, 1994. **3**(2): p. 108-124.
484. Williams, D.M. and F. Happe, *What did I say? Versus what did I think? Attributing false beliefs to self amongst children with and without autism.* Journal of Autism & Developmental Disorders, 2009. **39**(6): p. 865-73.
485. Prior, M., B. Dahlstrom, and T.-L. Squires, *Autistic children's knowledge of thinking and feeling states in other people.* Child Psychology & Psychiatry & Allied Disciplines, 1990. **31**(4): p. 587-601.
486. Fisher, N., F. Happe, and J. Dunn, *The relationship between vocabulary, grammar, and false belief task performance in children with autistic spectrum disorders and children with moderate learning difficulties.* Journal of Child Psychology & Psychiatry & Allied Disciplines, 2005. **46**(4): p. 409-19.
487. Farrant, A., J. Boucher, and M. Blades, *Metamemory in children with autism.* Child Development, 1999. **70**(1): p. 107-31.
488. Low, J., E. Goddard, and J. Melser, *Generativity and imagination in autism spectrum disorder: evidence from individual differences in children's impossible entity drawings.* British Journal of Developmental Psychology, 2009. **27**(Pt 2): p. 425-44.
489. Sparrevohn, R. and P.M. Howie, *Theory of mind in children with autistic disorder: evidence of developmental progression and the role of verbal ability.* J Child Psychol Psychiatry, 1995. **36**(2): p. 249-63.
490. Jarrold, C., et al., *Linking theory of mind and central coherence bias in autism and in the general population.* Developmental Psychology, 2000. **36**(1): p. 126-38.

491. Kenny, L., S.J. Cribb, and E. Pellicano, *Childhood Executive Function Predicts Later Autistic Features and Adaptive Behavior in Young Autistic People: a 12-Year Prospective Study.* Journal of Abnormal Child Psychology, 2019. **47**(6): p. 1089-1099.
492. Fahie, C.M. and D.K. Symons, *Executive functioning and theory of mind in children clinically referred for attention and behavior problems.* Journal of Applied Developmental Psychology, 2003. **24**(1): p. 51-73.
493. Dyck, M. and J. Piek, *How to distinguish normal from disordered children with poor language or motor skills.* International Journal of Language & Communication Disorders, 2010. **45**(3): p. 336-44.
494. Macaulay, C.E. and R.M. Ford, *Language and theory-of-mind development in prelingually deafened children with cochlear implants: A preliminary investigation.* Cochlear Implants International, 2006. **7**(1): p. 1-14.
495. Walker, E.A., et al., *Predictors of hearing aid use time in children with mild-to-severe hearing loss.* Language, Speech, and Hearing Services in Schools, 2013. **44**(1): p. 73-88.
496. Peters, K., E. Remmel, and D. Richards, *Language, mental state vocabulary, and false belief understanding in children with cochlear implants.* Language, Speech & Hearing Services in the Schools, 2009. **40**(3): p. 245-55.
497. Farrar, M.J., et al., *Language and theory of mind in preschool children with specific language impairment.* Journal of Communication Disorders, 2009. **42**(6): p. 428-41.
498. Farrant, B.M., M.T. Maybery, and J. Fletcher, *Language, cognitive flexibility, and explicit false belief understanding: Longitudinal analysis in typical development and specific language impairment.* Child Development, 2012. **83**(1): p. 223-235.
499. Razza, R.A. and C. Blair, *Associations among false-belief understanding, executive function, and social competence: A longitudinal analysis.* Journal of Applied Developmental Psychology, 2009. **30**(3): p. 332-343.
500. Blair, C., D. Granger, and R. Peters Razza, *Cortisol reactivity is positively related to executive function in preschool children attending head start.* Child Development, 2005. **76**(3): p. 554-67.
501. Blair, C. and R.P. Razza, *Relating effortful control, executive function, and false belief understanding to emerging math and literacy ability in kindergarten.* Child Development, 2007. **78**(2): p. 647-63.
502. Holmes, H.A., C. Black, and S.A. Miller, *A cross-task comparison of false belief understanding in a Head Start population.* Journal of Experimental Child Psychology, 1996. **63**(2): p. 263-285.
503. Ford, R.M., S.J. McDougall, and D. Evans, *Parent-delivered compensatory education for children at risk of educational failure: Improving the academic and self-regulatory skills of a Sure Start preschool sample.* British Journal of Psychology, 2009. **100**(Pt 4): p. 773-97.
504. Cicchetti, D., et al., *False belief understanding in maltreated children.* Development & Psychopathology, 2003. **15**(4): p. 1067-91.
505. Hughes, C., et al., *Good test--retest reliability for standard and advanced false-belief tasks across a wide range of abilities.* Journal of Child Psychology & Psychiatry & Allied Disciplines, 2000. **41**(4): p. 483-90.
506. Razza, R.P. and C. Blair, *False-Belief Understanding in a Low-Income Population.* Early Education and Development, 2003. **14**(4): p. 425-439.
507. Tompkins, V., M.J. Farrar, and Y. Guo, *Siblings, language, and false belief in low-income children.* Journal of Genetic Psychology, 2013. **174**(4): p. 457-63.
508. Tager-Flusberg, H., K. Sullivan, and J. Boshart, *Executive functions and performance on false belief tasks.* Developmental Neuropsychology, 1997. **13**(4): p. 487-493.
509. Jones, K.M., P.R. Champion, and L.J. Woodward, *Social competence of preschool children born very preterm.* Early Human Development, 2013. **89**(10): p. 795-802.
510. Walz, N.C., et al., *First-order theory of mind skills shortly after traumatic brain injury in 3- to 5-year-old children.* Developmental Neuropsychology, 2009. **34**(4): p. 507-519.
511. McAlpine, L.M. and C.L. Moore, *The development of social understanding in children with visual impairments.* Journal of Visual Impairment & Blindness, 1995. **89**(4): p. 349-358.

512. Green, S., L. Pring, and J. Swettenham, *An investigation of first-order false belief understanding of children with congenital profound visual impairment.* British Journal of Developmental Psychology, 2004. **22**(1): p. 1-17.
513. Guajardo, N.R. and A.C. Watson, *Narrative discourse and theory of mind development.* Journal of Genetic Psychology, 2002. **163**(3): p. 305-25.
514. Harris, P.L., et al., *Young Children's Theory of Mind and Emotion.* Cognition and Emotion, 1989. **3**(4): p. 379-400.
515. Harris, P.L., et al., *False-Belief Task--Emotion*, in *Understanding atypical emotions among children with autism. Journal of Autism and Developmental Disorders, 30, 195-203.* 2000.
516. Jahromi, L.B. and C.A. Stifter, *Individual differences in preschoolers' self-regulation and theory of mind.* Merrill-Palmer Quarterly, 2008. **54**(1): p. 125-150.
517. Paynter, J. and C. Peterson, *Language and ToM development in autism versus Asperger syndrome: Contrasting influences of syntactic versus lexical/semantic maturity.* Research in Autism Spectrum Disorders, 2010. **4**(3): p. 377-385.
518. de Rosnay, M., et al., *A lag between understanding false belief and emotion attribution in young children: Relationships with linguistic ability and mothers' mental-state language.* British Journal of Developmental Psychology, 2004. **22**(2): p. 197-218.
519. Fonagy, P., S. Redfern, and T. Charman, *The relationship between belief-desire reasoning and a projective measure of attachment security (SAT).* British Journal of Developmental Psychology, 1997. **15**(Pt 1): p. 51-61.
520. Stifter, C.A., et al., *Temperament and the development of conscience: The moderating role of effortful control.* Social Development, 2009. **18**(2): p. 353-374.
521. Hughes, C., et al., *Origins of Individual Differences in Theory of Mind: From Nature to Nurture?* Child Development, 2005. **76**(2): p. 356-370.
522. Lundy, B.L., *Paternal and maternal mind-mindedness and preschoolers' theory of mind: The mediating role of interactional attunement.* Social Development, 2013. **22**(1): p. 58-74.
523. Meins, E. and C. Fernyhough, *Linguistic acquisitional style and mentalising development: The role of maternal mind-mindedness.* Cognitive Development, 1999. **14**(3): p. 363-380.
524. Shakoor, S., et al., *A prospective longitudinal study of children's theory of mind and adolescent involvement in bullying.* Journal of Child Psychology & Psychiatry & Allied Disciplines, 2012. **53**(3): p. 254-61.
525. Symons, D.K., et al., *Theory of mind and mental state discourse during book reading and story-telling tasks.* British Journal of Developmental Psychology, 2005. **23**(1): p. 81-102.
526. O'Reilly, J. and C.C. Peterson, *Scaling theory of mind development in Indigenous- and Anglo-Australian toddlers and older children.* Journal of Cross-Cultural Psychology, 2014. **45**(9): p. 1489-1501.
527. de Rosnay, M. and P.L. Harris, *Individual differences in children's understanding of emotion: the roles of attachment and language.* Attach Hum Dev, 2002. **4**(1): p. 39-54.
528. Rizzo, M.T. and M. Killen, *How social status influences our understanding of others' mental states.* Journal of Experimental Child Psychology, 2018. **169**: p. 30-41.
529. Allen, J.R. and K. Kinsey, *Teaching theory of mind.* Early Education and Development, 2013. **24**(6): p. 865-876.
530. Jenkins, J.M. and J.W. Astington, *Theory of mind and social behavior: Causal models tested in a longitudinal study.* Merrill-Palmer Quarterly, 2000. **46**(2): p. 203-220.
531. Macaulay, C.E. and R.M. Ford, *Family influences on the cognitive development of profoundly deaf children: Exploring the effects of socioeconomic status and siblings.* Journal of Deaf Studies and Deaf Education, 2013. **18**(4): p. 545-562.
532. Matthews, N.L., W.A. Goldberg, and A.F. Lukowski, *Theory of mind in children with autism spectrum disorder: do siblings matter?* Autism research : Official Journal of the International Society for Autism Research, 2013. **6**(5): p. 443-53.
533. Meins, E., et al., *Pathways to Understanding Mind: Construct Validity and Predictive Validity of Maternal Mind-Mindedness.* Child Development, 2003. **74**(4): p. 1194-1211.

534. Meins, E., et al., *Maternal mind-mindedness and attachment security as predictors of theory of mind understanding.* Child Development, 2002. **73**(6): p. 1715-26.
535. Mutter, B., M.B. Alcorn, and M. Welsh, *Theory of mind and executive function: working-memory capacity and inhibitory control as predictors of false-belief task performance.* Perceptual & Motor Skills, 2006. **102**(3): p. 819-35.
536. Newton, E. and V. Jenvey, *Play and theory of mind: Associations with social competence in young children.* Early Child Development and Care, 2011. **181**(6): p. 761-773.
537. Pellicano, E., M. Maybery, and K. Durkin, *Central coherence in typically developing preschoolers: does it cohere and does it relate to mindreading and executive control?* Journal of Child Psychology & Psychiatry & Allied Disciplines, 2005. **46**(5): p. 533-47.
538. Peterson, C.C. and M. Siegal, *Mindreading and moral awareness in popular and rejected preschoolers.* British Journal of Developmental Psychology, 2002. **20**(2): p. 205-224.
539. Randell, A.C. and C.C. Peterson, *Affective qualities of sibling disputes, mothers' conflict attitudes, and children's theory of mind development.* Social Development, 2009. **18**(4): p. 857-874.
540. de Villiers, J.G. and J.E. Pyers, *Complements to cognition: A longitudinal study of the relationship between complex syntax and false-belief-understanding.* Cognitive Development, 2002. **17**(1): p. 1037-1060.
541. Suddendorf, T., C. Fletcher-Flinn, and L. Johnston, *Pantomime and theory of mind.* The Journal of Genetic Psychology: Research and Theory on Human Development, 1999. **160**(1): p. 31-45.
542. Symons, D.K. and S.E. Clark, *A longitudinal study of mother-child relationships and theory of mind in the preschool period.* Social Development, 2000. **9**(1): p. 3-23.
543. Symons, D.K., K.-L.M. Fossum, and T. Collins, *A Longitudinal Study of Belief and Desire State Discourse During Mother-Child Play and Later False Belief Understanding.* Social Development, 2006. **15**(4): p. 676-691.
544. Talwar, V. and K. Lee, *Social and cognitive correlates of children's lying behavior.* Child Development, 2008. **79**(4): p. 866-81.
545. Bialecka-Pikul, M., *Teaching children to understand metaphors as a path leading to theory of mind development.* European Journal of Developmental Psychology, 2010. **7**(5): p. 529-544.
546. Wright, B.C. and J. Mahfoud, *A teacher-centered exploration of the relevance of social factors to theory of mind development.* Scandinavian Journal of Psychology, 2014. **55**(1): p. 17-25.
547. Capage, L. and A.C. Watson, *Individual differences in theory of mind, aggressive behavior, and social skills in young children.* Early Education and Development, 2001. **12**(4): p. 613-628.
548. Zelazo, P.D. and J.J. Boseovski, *Video reminders in a representational change task: memory for cues but not beliefs or statements.* Journal of Experimental Child Psychology, 2001. **78**(2): p. 107-29.
549. DiYanni, C., et al., *'I won't trust you if I think you're trying to deceive me': Relations between selective trust, theory of mind, and imitation in early childhood.* Journal of Cognition and Development, 2012. **13**(3): p. 354-371.
550. Flynn, E. and A. Whiten, *Experimental "microcultures" in young children: identifying biographic, cognitive, and social predictors of information transmission.* Child Development, 2012. **83**(3): p. 911-25.
551. Ford, R.M., et al., *Empathy, theory of mind, and individual differences in the appropriation bias among 4- and 5-year-olds.* Journal of Experimental Child Psychology, 2011. **110**(4): p. 626-646.
552. Kamawar, D. and D.R. Olson, *Children's representational theory of language: The problem of opaque contexts.* Cognitive Development, 1999. **14**(4): p. 531-548.
553. Mitchell, P. and L.M. Taylor, *Shape constancy and theory of mind: Is there a link?* Cognition, 1999. **70**(2): p. 167-190.
554. Muller, U., et al., *False belief understanding: The influence of person, grammatical mood, counterfactual reasoning and working memory.* British Journal of Developmental Psychology, 2007. **25**(4): p. 615-632.

555. Favez, N., et al., *The development of family alliance from pregnancy to toddlerhood and child outcomes at 5 years.* Family Process, 2012. **51**(4): p. 542-56.
556. Pelletier, J. and J.W. Astington, *Action, consciousness and theory of mind: Children's ability to coordinate story characters' actions and thoughts.* Early Education and Development, 2004. **15**(1): p. 5-22.
557. Hala, S., S. Hug, and A. Henderson, *Executive Function and False-Belief Understanding in Preschool Children: Two Tasks Are Harder Than One.* Journal of Cognition and Development, 2003. **4**(3): p. 275-298.
558. Freeman, N.H. and H. Lacohee, *Making explicit 3-year-olds' implicit competence with their own false beliefs.* Cognition, 1995. **56**(1): p. 31-60.
559. Geangu, E., *Affirmation and negation.* Cognitie Creier Comportament, 2002. **6**(3): p. 253-282.
560. Lalonde, C.E. and M.J. Chandler, *False belief understanding goes to school: On the social-emotional consequences of coming early or late to a first theory of mind.* Cognition and Emotion, 1995. **9**(2-3): p. 167-185.
561. Slade, L. and T. Ruffman, *How language does (and does not) relate to theory of mind: A longitudinal study of syntax, semantics, working memory and false belief.* British Journal of Developmental Psychology, 2005. **23**(1): p. 117-141.
562. Thompson, R. and B. Thornton, *Gender and theory of mind in preschoolers' group effort: Evidence for timing differences behind children's earliest social loafing.* The Journal of Social Psychology, 2014. **154**(6): p. 475-479.
563. Sobel, D.M., et al., *The role of probability and intentionality in preschoolers' causal generalizations.* Journal of Cognition and Development, 2009. **10**(4): p. 262-284.
564. Carlson, S.M., L.J. Moses, and L.J. Claxton, *Individual differences in executive functioning and theory of mind: An investigation of inhibitory control and planning ability.* Journal of Experimental Child Psychology, 2004. **87**(4): p. 299-319.
565. Benigno, J.P. and M. Farrar, *Determinants of joint attention in young siblings' play.* Infant and Child Development, 2012. **21**(2): p. 160-174.
566. Joseph, R.M. and H. Tager-Flusberg, *The relationship of theory of mind and executive functions to symptom type and severity in children with autism.* Development & Psychopathology, 2004. **16**(1): p. 137-55.
567. Liebermann, D., G.F. Giesbrecht, and U. Muller, *Cognitive and emotional aspects of self-regulation in preschoolers.* Cognitive Development, 2007. **22**(4): p. 511-529.
568. McAlister, A. and C.C. Peterson, *Mental playmates: Siblings, executive functioning and theory of mind.* British Journal of Developmental Psychology, 2006. **24**(4): p. 733-751.
569. Metcalf, J.L. and C.M. Atance, *Do preschoolers save to benefit their future selves?* Cognitive Development, 2011. **26**(4): p. 371-382.
570. O'Brien, K., V. Slaughter, and C.C. Peterson, *Sibling influences on theory of mind development for children with ASD.* Journal of Child Psychology & Psychiatry & Allied Disciplines, 2011. **52**(6): p. 713-9.
571. Walker, S., *Gender differences in the relationship between young children's peer-related social competence and individual differences in theory of mind.* Journal of Genetic Psychology, 2005. **166**(3): p. 297-312.
572. Walker, R.F. and T. Murachver, *Representation and theory of mind development.* Developmental Psychology, 2012. **48**(2): p. 509-20.
573. Carlson, S.M. and L.J. Moses, *Individual differences in inhibitory control and children's theory of mind.* Child Development, 2001. **72**(4): p. 1032-53.
574. Carlson, S.M., L.J. Moses, and C. Breton, *How specific is the relation between executive function and theory of mind? Contributions of inhibitory control and working memory.* Infant and Child Development, 2002. **11**(2): p. 73-92.
575. Rohrer, L.M., et al., *Effects of maternal negativity and of early and recent recurrent depressive disorder on children's false belief understanding.* Developmental Psychology, 2011. **47**(1): p. 170-181.
576. Samuels, M.C., P.J. Brooks, and D. Frye, *Strategic game playing in children through the windows task.* British Journal of Developmental Psychology, 1996. **14**(2): p. 159-172.
577. Scullin, M.H. and K. Bonner, *Theory of mind, inhibitory control, and preschool-age children's suggestibility in different interviewing contexts.* Journal of Experimental Child Psychology, 2006. **93**(2): p. 120-38.

578. McAlister, A.R. and T. Cornwell, *Children's brand symbolism understanding: Links to theory of mind and executive functioning.* Psychology & Marketing, 2010. **27**(3): p. 203-228.
579. McGlamery, M.E., et al., *Theory of mind, attention, and executive function in kindergarten boys.* Emotional & Behavioural Difficulties, 2007. **12**(1): p. 29-47.
580. Adrian, J.E., R.A. Clemente, and L. Villanueva, *Mothers' use of cognitive state verbs in picture-book reading and the development of children's understanding of mind: a longitudinal study.* Child Development, 2007. **78**(4): p. 1052-67.
581. Astington, J.W. and J.M. Jenkins, *Theory of mind development and social understanding.* Cognition and Emotion, 1995. **9**(2-3): p. 151-165.
582. Watson, A.C., K.M. Painter, and M.H. Bornstein, *Longitudinal relations between 2-year-olds' language and 4-year-olds' theory of mind.* Journal of Cognition and Development, 2001. **2**(4): p. 449-457.
583. Bernstein, D.M., et al., *Hindsight bias and developing theories of mind.* Child Development, 2007. **78**(4): p. 1374-94.
584. Astington, J.W. and J.M. Jenkins, *A longitudinal study of the relation between language and theory-of-mind development.* Developmental Psychology, 1999. **35**(5): p. 1311-20.
585. Cahill, K.R., et al., *Theory of mind, self-worth and the mother-child relationship.* Social Development, 2007. **16**(1): p. 45-56.
586. Cole, K. and P. Mitchell, *Family background in relation to deceptive ability and understanding of the mind.* Social Development, 1998. **7**(2): p. 181-197.
587. Ford, R.M., et al., *Executive and theory-of-mind contributions to event-based prospective memory in children: exploring the self-projection hypothesis.* Journal of Experimental Child Psychology, 2012. **111**(3): p. 468-89.
588. Chaplin, L.N. and M.I. Norton, *Why we think we can't dance: Theory of mind and children's desire to perform.* Child Development, 2015. **86**(2): p. 651-658.
589. Moore, C., K. Pure, and D. Furrow, *Children's understanding of the modal expression of speaker certainty and uncertainty and its relation to the development of a representational theory of mind.* Child Development, 1990. **61**(3): p. 722-30.
590. Howard, A.A., L. Mayeux, and L.R. Naigles, *Conversational correlates of children's acquisition of mental verbs and a theory of mind.* First Language, 2008. **28**(4): p. 375-402.
591. Johnson, E.A., *Children's understanding of epistemic conduct in self-deception and other false belief stories.* Child Development, 1997. **68**(6): p. 1117-32.
592. Lackner, C., et al., *Dopamine receptor D4 gene variation predicts preschoolers' developing theory of mind.* Developmental Science, 2012. **15**(2): p. 272-80.
593. Watson, A.C., et al., *Social interaction skills and theory of mind in young children.* Developmental Psychology, 1999. **35**(2): p. 386-91.
594. Sabbagh, M.A., et al., *The development of executive functioning and theory of mind. A comparison of Chinese and U.S. preschoolers.* Psychological Science, 2006. **17**(1): p. 74-81.
595. Gilpin, A.T., M.M. Brown, and J.M. Pierucci, *Relations between fantasy orientation and emotion regulation in preschool.* Early Education and Development, 2015. **26**(7): p. 920-932.
596. Taylor, M. and S.M. Carlson, *The relation between individual differences in fantasy and theory of mind.* Child Development, 1997. **68**(3): p. 436-55.
597. Buttelmann, D., et al., *Eighteen-month-olds understand false beliefs in an unexpected-contents task.* Journal of Experimental Child Psychology, 2014. **119**: p. 120-6.
598. Ziv, M. and D. Frye, *The relation between desire and false belief in children's theory of mind: no satisfaction?* Developmental Psychology, 2003. **39**(5): p. 859-76.
599. Bradmetz, J., *Acquisition of a theory of mind and intellectual development in the child aged 3 to 5.* Cahiers de Psychologie Cognitive/Current Psychology of Cognition, 1998. **17**(1): p. 95-113.
600. Hickling, A.K., H.M. Wellman, and G.M. Gottfried, *Preschoolers' understanding of others' mental attitudes towards pretend happenings.* British Journal of Developmental Psychology, 1997. **15**(3): p. 339-354.
601. Shatz, M., et al., *The influence of language and socioeconomic status on children's understanding of false belief.* Developmental Psychology, 2003. **39**(4): p. 717-29.

602. Davis, H.L. and C. Pratt, *The development of children's theory of mind: The working memory explanation.* Australian Journal of Psychology, 1995. **47**(1): p. 25-31.
603. MacLaren, R. and D. Olson, *Trick or treat: Children's understanding of surprise.* Cognitive Development, 1993. **8**(1): p. 27-46.
604. Callaghan, T.C., P. Rochat, and J. Corbit, *Young children's knowledge of the representational function of pictorial symbols: Development across the preschool years in three cultures.* Journal of Cognition and Development, 2012. **13**(3): p. 320-353.
605. Brandt, S., et al., *Children's understanding of first- and third-person perspectives in complement clauses and false-belief tasks.* Journal of Experimental Child Psychology, 2016. **151**: p. 131-43.
606. Petkova, A.V. and K.M. Cain, *Preschool Fantasy-Reality Discrimination: Influences of Trait and Primed Fearfulness.* Journal of Genetic Psychology, 2017. **178**(2): p. 133-138.
607. Ball, C.L., J.G. Smetana, and M.L. Sturge-Apple, *Following My Head and My Heart: Integrating Preschoolers' Empathy, Theory of Mind, and Moral Judgments.* Child Development, 2017. **88**(2): p. 597-611.
608. Mulvey, K.L., M.T. Rizzo, and M. Killen, *Challenging gender stereotypes: Theory of mind and peer group dynamics.* Developmental Science, 2016. **19**(6): p. 999-1010.
609. O'Toole, S.E., C.P. Monks, and S. Tsermentseli, *Executive function and theory of mind as predictors of aggressive and prosocial behavior and peer acceptance in early childhood.* Social Development, 2017: p. No Pagination Specified-No Pagination Specified.
610. Harwood, M.D. and M.J. Farrar, *Conflicting emotions: The connection between affective perspective taking and theory of mind.* British Journal of Developmental Psychology, 2006. **24**(2): p. 401-418.
611. Ruffman, T., et al., *The ABCs of Deception: Do Young Children Understand Deception in the Same Way as Adults?* Vol. 29. 1993. 74-87.
612. Mahy, C.E.V., et al., *Testing the validity of a continuous false belief task in 3- to 7-year-old children.* Journal of Experimental Child Psychology, 2017. **160**: p. 50-66.
613. Mulvey, K.L., K. Buchheister, and K. McGrath, *Evaluations of intergroup resource allocations: The role of theory of mind.* Journal of Experimental Child Psychology, 2016. **142**: p. 203-11.
614. Cunningham, S.J., et al., *Exploring early self-referential memory effects through ownership.* British Journal of Developmental Psychology, 2013. **31**(3): p. 289-301.
615. Julien, H.M., L.H. Finestack, and J. Reichle, *Requests for Communication Repair Produced by Typically Developing Preschool-Age Children.* Journal of Speech Language & Hearing Research, 2019. **62**(6): p. 1823-1838.
616. Vendetti, C., D. Kamawar, and K.E. Andrews, *Theory of mind and preschoolers' understanding of misdeed and politeness lies.* Developmental Psychology, 2019. **55**(4): p. 823-834.
617. O'Connor, A.M. and A.D. Evans, *The role of theory of mind and social skills in predicting children's cheating.* Journal of Experimental Child Psychology, 2019. **179**: p. 337-347.
618. Papera, M., et al., *Development of second-order theory of mind: Assessment of environmental influences using a dynamic system approach.* International Journal of Behavioral Development, 2019. **43**(3): p. 245-254.
619. Tahiroglu, D. and M. Taylor, *Anthropomorphism, social understanding, and imaginary companions.* British Journal of Developmental Psychology, 2019. **37**(2): p. 284-299.
620. Chasiotis, A., et al., *The emergence of the independent self: Autobiographical memory as a mediator of false belief understanding and sociocultural motive orientation in cameroonian and German preschoolers.* Journal of Cross-Cultural Psychology, 2010. **41**(3): p. 368-390.
621. Cheung, H., *False belief and language comprehension in Cantonese-speaking children.* Journal of Experimental Child Psychology, 2006. **95**(2): p. 79-98.
622. Ma, F., et al., *To lie or not to lie? The influence of parenting and theory-of-mind understanding on three-year-old children's honesty.* Journal of Moral Education, 2015. **44**(2): p. 198-212.

623. Ketelaars, M.P., et al., *Narrative competence and underlying mechanisms in children with pragmatic language impairment.* Applied Psycholinguistics, 2012. **33**(2): p. 281-303.
624. O'Reilly, K., C.C. Peterson, and H.M. Wellman, *Sarcasm and advanced theory of mind understanding in children and adults with prelingual deafness.* Developmental Psychology, 2014. **50**(7): p. 1862-1877.
625. Bradmetz, J. and C. Gauthier, *The development of interindividual sharing of knowledge and beliefs in 5- to 9-year-old children.* Journal of Genetic Psychology, 2005. **166**(1): p. 45-53.
626. Durrleman, S., M. Burnel, and A. Reboul, *Theory of mind in sli revisited: Links with syntax, comparisons with asd.* International Journal of Language & Communication Disorders, 2017: p. No Pagination Specified-No Pagination Specified.
627. Burnside, K., K. Wright, and D. Poulin-Dubois, *Social motivation and implicit theory of mind in children with autism spectrum disorder.* Autism research : Official Journal of the International Society for Autism Research, 2017. **10**(11): p. 1834-1844.
628. Burnside, K., K. Wright, and D. Poulin-Dubois, *Social orienting predicts implicit false belief understanding in preschoolers.* Journal of Experimental Child Psychology, 2018. **175**: p. 67-79.
629. Lohmann, H., M. Carpenter, and J. Call, *Guessing versus choosing - and seeing versus believing - in false belief tasks.* British Journal of Developmental Psychology, 2005. **23**(3): p. 451-469.
630. Perner, J., B. Lang, and D. Kloo, *Theory of mind and self-control: more than a common problem of inhibition.* Child Development, 2002. **73**(3): p. 752-67.
631. Perner, J., D. Kloo, and E. Gornik, *Episodic memory development: Theory of mind is part of re-experiencing experienced events.* Infant and Child Development, 2007. **16**(5): p. 471-490.
632. Priewasser, B., J. Roessler, and J. Perner, *Competition as rational action: why young children cannot appreciate competitive games.* Journal of Experimental Child Psychology, 2013. **116**(2): p. 545-59.
633. Rakoczy, H., et al., *Explicit theory of mind is even more unified than previously assumed: Belief ascription and understanding aspectuality emerge together in development.* Child Development, 2015. **86**(2): p. 486-502.
634. Carpenter, M., J. Call, and M. Tomasello, *A new false belief test for 36-month-olds.* British Journal of Developmental Psychology, 2002. **20**(3): p. 393-420.
635. Buttelmann, D., M. Carpenter, and M. Tomasello, *Eighteen-month-old infants show false belief understanding in an active helping paradigm.* Cognition, 2009. **112**(2): p. 337-42.
636. Dörrenberg, S., H. Rakoczy, and U. Liszkowski, *How (not) to measure infant theory of mind: Testing the replicability and validity of four non-verbal measures.* Cognitive Development, 2018: p. No Pagination Specified-No Pagination Specified.
637. Dorrenberg, S., et al., *Reliability and generalizability of an acted-out false belief task in 3-year-olds.* Infant Behavior & Development, 2019. **54**: p. 13-21.
638. Spanoudis, G., et al., *Embedding cognizance in intellectual development.* Journal of Experimental Child Psychology, 2015. **132**: p. 32-50.
639. Lecciso, F., S. Petrocchi, and A. Marchetti, *Hearing mothers and oral deaf children: An atypical relational context for theory of mind.* European Journal of Psychology of Education, 2013. **28**(3): p. 903-922.
640. Naito, M. and K. Nagayama, *Autistic Children's Use of Semantic Common Sense and Theory of Mind: A Comparison with Typical and Mentally Retarded Children.* Journal of Autism and Developmental Disorders, 2004. **34**(5): p. 507-519.
641. Ohtsubo, Y., *Japanese children's development of false belief understanding.* Psychological Reports, 2007. **100**(1): p. 87-97.
642. Naito, M. and Y. Seki, *The relationship between second-order false belief and display rules reasoning: The integration of cognitive and affective social understanding.* Developmental Science, 2009. **12**(1): p. 150-164.
643. Schug, J., et al., *The Development of Theory of Mind and Positive and Negative Reciprocity in Preschool Children.* Frontiers in Psychology, 2016. **7**: p. 888.
644. Miller, S.E. and S. Marcovitch, *How theory of mind and executive function co-develop.* Review of Philosophy and Psychology, 2012. **3**(4): p. 597-625.
645. Putko, A. and A. Zlotogorska, *Predictions of actions and their justifications in false-belief tasks: The role of executive function.* Polish Psychological Bulletin, 2014. **45**(4): p. 500-510.

646. Dahlgren, S., A.D. Sandberg, and M. Larsson, *Theory of mind in children with severe speech and physical impairments.* Research in Developmental Disabilities, 2010. **31**(2): p. 617-624.
647. Sundqvist, A., et al., *Understanding minds: early cochlear implantation and the development of theory of mind in children with profound hearing impairment.* International Journal of Pediatric Otorhinolaryngology, 2014. **78**(3): p. 537-43.
648. Carlsson, E., et al., *Assessing False-Belief Understanding in Children with Autism Using a Computer Application: A Pilot Study.* Journal of Psycholinguistic Research, 2018. **47**(5): p. 1085-1099.
649. Jarrold, C., R. Mansergh, and C. Whiting, *The representational status of pretence: Evidence from typical development and autism.* British Journal of Developmental Psychology, 2010. **28**(2): p. 239-254.
650. Van Herwegen, J., T.J. Smith, and D. Dimitriou, *Exploring different explanations for performance on a theory of mind task in Williams syndrome and autism using eye movements.* Research in Developmental Disabilities, 2015. **45-46**: p. 202-9.
651. Bowler, D.M., et al., *Understanding the Mind or Predicting Signal-Dependent Action? Performance of Children With and Without Autism on Analogues of the False-Belief Task.* Journal of Cognition and Development, 2005. **6**(2): p. 259-283.
652. Schopler, E.R.R.J. and R.B. R., *Childhood Autism Rating Scale*, in *Performance of autistic and control subjects on three cognitive perspective-taking tasks. Journal of Autism and Developmental Disorders, 24, 53-66.* 1994.
653. Whitehouse, A. and K. Hird, *Is grammatical competence a precondition for belief-desire reasoning? Evidence from typically developing children and those with autism.* Advances in Speech Language Pathology, 2004. **6**(1): p. 39-51.
654. Peterson, D.M. and D.M. Bowler, *Counterfactual reasoning and false belief understanding in children with autism, children with severe learning difficulties and children with typical development.* Autism, 2000. **4**(4): p. 391-405.
655. Peterson, C.C., et al., *Children with autism can track others' beliefs in a competitive game.* Developmental Science, 2013. **16**(3): p. 443-50.
656. Iao, L.-S. and S.R. Leekam, *Nonspecificity and theory of mind: New evidence from a nonverbal false-sign task and children with autism spectrum disorders.* Journal of Experimental Child Psychology, 2014. **122**: p. 1-20.
657. Branigan, H.P., A. Tosi, and K. Gillespie-Smith, *Spontaneous lexical alignment in children with an autistic spectrum disorder and their typically developing peers.* Journal of Experimental Psychology: Learning, Memory, and Cognition, 2016. **42**(11): p. 1821-1831.
658. Baron-Cohen, S., A.M. Leslie, and U. Frith, *Does the autistic child have a "theory of mind"?* Cognition, 1985. **21**(1): p. 37-46.
659. Leekam, S.R. and J. Perner, *Does the autistic child have a metarepresentational deficit?* Cognition, 1991. **40**(3): p. 203-18.
660. Clarke, M.T., D. Loganathan, and J. Swettenham, *Assessing true and false belief in young children with cerebral palsy through anticipatory gaze behaviours: a pilot study.* Research in Developmental Disabilities, 2012. **33**(6): p. 2058-66.
661. Russell, J., et al., *The "windows task" as a measure of strategic deception in preschoolers and autistic subjects.* British Journal of Developmental Psychology, 1991. **9**(2): p. 331-349.
662. Rasmussen, C., K. Wyper, and V. Talwar, *The relation between theory of mind and executive functions in children with fetal alcohol spectrum disorders.* Canadian Journal of Clinical Pharmacology, 2009. **16**(2): p. e370-80.
663. Abbeduto, L., et al., *Relationship between theory of mind and language ability in children and adolescents with intellectual disability.* Journal of Intellectual Disability Research, 2004. **48**(Pt 2): p. 150-9.
664. Miller, C.A., *False belief understanding in children with specific language impairment.* Journal of Communication Disorders, 2001. **34**(1-2): p. 73-86.
665. Miller, C., *False belief and sentence complement performance in children with specific language impairment.* International Journal of Language & Communication Disorders, 2004. **39**(2): p. 191-213.
666. Garner, P.W., S.M. Curenton, and K. Taylor, *Predictors of mental state understanding in preschoolers of varying socioeconomic backgrounds.* International Journal of Behavioral Development, 2005. **29**(4): p. 271-281.
667. Talwar, V., et al., *Lie-telling behavior in children with autism and its relation to false-belief understanding.* Focus on Autism and Other Developmental Disabilities, 2012. **27**(2): p. 122-129.

668. Shahaeian, A.H., J.D., Razmjoee, M.,Teymoori, A., Wang, C., *Towards a better understanding of the relationship between executive control and theory of mind: An intra-cultural comparison of three diverse samples.* Developmental Science, 2015. **18**: p. 671-685.
669. Leithner-Dziubas, K., et al., *[Mentalization and bonding in chronic pelvic pain patients: a pilot study].* Zeitschrift Fuer Psychosomatische Medizin und Psychotherapie, 2010. **56**(2): p. 179-90.
670. Van Herwegen, J., D. Dimitriou, and G. Rundblad, *Performance on verbal and low-verbal false belief tasks: evidence from children with Williams syndrome.* Journal of Communication Disorders, 2013. **46**(5-6): p. 440-8.
671. Murray, L., et al., *Children's social representations in dolls' house play and theory of mind tasks, and their relation to family adversity and child disturbance.* Social Development, 1999. **8**(2): p. 179-200.
672. Adrian, J.E., et al., *Parent-child picture-book reading, mothers' mental state language and children's theory of mind.* Journal of Child Language, 2005. **32**(3): p. 673-86.
673. Carroll, D.J., et al., *How do alternative ways of responding influence 3- and 4-year-olds' performance on tests of executive function and theory of mind?* Journal of Experimental Child Psychology, 2012. **112**(3): p. 312-25.
674. Carlson, S.M., L.J. Claxton, and L.J. Moses, *The relation between executive function and theory of mind is more than skin deep.* Journal of Cognition and Development, 2015. **16**(1): p. 186-197.
675. Dick, A.S., W.F. Overton, and S.L. Kovacs, *The Development of Symbolic Coordination: Representation of Imagined Objects, Executive Function, and Theory of Mind.* Journal of Cognition and Development, 2005. **6**(1): p. 133-161.
676. Moll, H., S. Kane, and L. McGowan, *Three-year-olds express suspense when an agent approaches a scene with a false belief.* Developmental Science, 2015: p. No Pagination Specified.
677. Robinson, E.J. and P. Mitchell, *Children's interpretation of messages from a speaker with a false belief.* Child Development, 1992. **63**(3): p. 639-52.
678. Ruffman, T., J. Perner, and L. Parkin, *How parenting style affects false belief understanding.* Social Development, 1999. **8**(3): p. 395-411.
679. Skarakis-Doyle, E., et al., *Preschoolers' sensitivity to the maxims of the cooperative principle: Scaffolds and developmental trends.* Discourse Processes, 2014. **51**(4): p. 333-356.
680. Abu-Akel, A. and A.L. Bailey, *Indexical and symbolic referencing: what role do they play in children's success on theory of mind tasks?* Cognition, 2001. **80**(3): p. 263-81.
681. Brown, E.L. and R. Bull, *Can task modifications influence children's performance on false belief tasks?* European Journal of Developmental Psychology, 2007. **4**(3): p. 273-292.
682. Cassidy, K.W., *Three- and four-year-old children's ability to use desire- and belief-based reasoning.* Cognition, 1998. **66**(1): p. B1-11.
683. Cassidy, K.W., *Preschoolers' use of desires to solve theory of mind problems in a pretense context.* Developmental Psychology, 1998. **34**(3): p. 503-11.
684. Curenton, S.M., *The association between narratives and theory of mind for low-income preschoolers.* Early Education and Development, 2004. **15**(2): p. 121-145.
685. Hala, S., M. Chandler, and A.S. Fritz, *Fledgling theories of mind: Deception as a marker of three-year-olds' understanding of false belief.* Child Development, 1991. **62**(1): p. 83-97.
686. Keenan, T., *Memory span as a predictor of false belief understanding.* New Zealand Journal of Psychology, 1998. **27**(2): p. 36-43.
687. Suddendorf, T. and C.M. Fletcher-Flinn, *Children's divergent thinking improves when they understand false beliefs.* Creativity Research Journal, 1999. **12**(2): p. 115-128.
688. Fletcher-Flinn, C.M. and T. Suddendorf, *Do computers affect "The Mind"?* Journal of Educational Computing Research, 1996. **15**(2): p. 97-112.
689. Garnham, W.A. and T. Ruffman, *Doesn't see, doesn't know: Is anticipatory looking really related to understanding of belief?* Developmental Science, 2001. **4**(1): p. 94-100.
690. Scott, R.M., et al., *False-belief understanding in 2.5-year-olds: evidence from two novel verbal spontaneous-response tasks.* Developmental Science, 2012. **15**(2): p. 181-93.

691. Lazaridis, M., *The emergence of a temporally extended self and factors that contribute to its development: From theoretical and empirical perspectives: Abstract.* Monographs of the Society for Research in Child Development, 2013. **78**(2): p. vii.
692. Southgate, V., A. Senju, and G. Csibra, *Action anticipation through attribution of false belief by 2-year-olds.* Psychological Science, 2007. **18**(7): p. 587-592.
693. Tan-Niam, C.S., D. Wood, and C. O'Malley, *A cross-cultural perspective on children's theories of mind and social interaction.* Early Child Development and Care, 1998. **144**: p. 55-67.
694. Zmyj, N., W. Prinz, and M.M. Daum, *Eighteen-month-olds' memory interference and distraction in a modified A-not-B task is not associated with their anticipatory looking in a false-belief task.* Frontiers in Psychology, 2015. **6**: p. 857.
695. Zmyj, N. and D. Bischof-Köhler, *The development of gender constancy in early childhood and its relation to time comprehension and false-belief understanding.* Journal of Cognition and Development, 2015. **16**(3): p. 455-470.
696. Rhodes, M. and A.C. Brandone, *Three-year-olds' theories of mind in actions and words.* Frontiers in Psychology Vol 5 Mar 2014, ArtID 263, 2014. **5**.
697. Callaghan, T., et al., *Synchrony in the onset of mental-state reasoning: evidence from five cultures.* Psychological Science, 2005. **16**(5): p. 378-84.
698. Ahn, S. and S.A. Miller, *Theory of mind and self-concept: A comparison of Korean and American children.* Journal of Cross-Cultural Psychology, 2012. **43**(5): p. 671-686.
699. Mayes, L.C., et al., *Test-retest reliability for false-belief tasks.* Journal of Child Psychology & Psychiatry & Allied Disciplines, 1996. **37**(3): p. 313-9.
700. Ellis, B.J., et al., *The predator-avoidance effect: An evolved constraint on emerging theory of mind.* Evolution and Human Behavior, 2014. **35**(3): p. 245-256.
701. Mitchell, P. and E. Robinson, *Discrepant utterances resulting from a false belief: Children's evaluations.* Child Development, 1994. **65**(4): p. 1214-1227.
702. Gliga, T., et al., *Spontaneous belief attribution in younger siblings of children on the autism spectrum.* Developmental Psychology, 2014. **50**(3): p. 903-13.
703. Anonymous, *The development of ambiguous figure perception.* Monographs of the Society for Research in Child Development, 2011. **76**(1): p. vii, 1-130.
704. Wimmer, M.C. and M.J. Doherty, *The development of ambiguous figure perception: II. Study 1-Conceptual understanding of ambiguous figures.* Monographs of the Society for Research in Child Development, 2011. **76**(1): p. 24-39.
705. Wimmer, M.C. and M.J. Doherty, *The development of ambiguous figure perception: V. Study 4-Reversing ambiguous figures and mental imagery.* Monographs of the Society for Research in Child Development, 2011. **76**(1): p. 69-86.
706. Wimmer, M.C. and M.J. Doherty, *The development of ambiguous figure perception: IV. Study 3-Reversing ambiguous figures and executive function.* Monographs of the Society for Research in Child Development, 2011. **76**(1): p. 49-68.
707. Wimmer, M.C. and M.J. Doherty, *The development of ambiguous figure perception: III. Study 2-Reversing ambiguous figures.* Monographs of the Society for Research in Child Development, 2011. **76**(1): p. 40-48.
708. McElwain, N.L. and B.L. Volling, *Attachment security and parental sensitivity during infancy: Associations with friendship quality and false-belief understanding at age 4.* Journal of Social and Personal Relationships, 2004. **21**(5): p. 639-667.
709. Melot, A.-M. and N. Angeard, *Theory of mind: Is training contagious?* Developmental Science, 2003. **6**(2): p. 178-184.
710. Doherty, M.J. and M.C. Wimmer, *Children's understanding of ambiguous figures: Which cognitive developments are necessary to experience reversal?* Cognitive Development, 2005. **20**(3): p. 407-421.
711. Glenn, S.M., K. Johnson, and F. Parry, *Onset of theory of mind: Methodological considerations.* Early Child Development and Care, 1993. **86**: p. 39-51.
712. Peterson, C. and V. Slaughter, *Opening windows into the mind: Mothers' preferences for mental state explanations and children's theory of mind.* Cognitive Development, 2003. **18**(3): p. 399-429.
713. Battacchi, M.W., G. Celani, and A. Bertocchi, *The influence of personal involvement on the performance in a false belief task: A structural analysis.* International Journal of Behavioral Development, 1997. **21**(2): p. 313-329.
714. Bright-Paul, A., et al., *Children's memory distortions following social contact with a co-witness: disentangling social and cognitive mechanisms.* Memory, 2012. **20**(6): p. 580-95.

715. Carbonero Martin, M.A., M.C. Saiz Manzanares, and J.M. Roman Sanchez, *Effect of a metacognitive training program of mentalist skills.* Psicothema, 2013. **25**(1): p. 31-7.
716. Fletcher-Flinn, C. and H. Snelson, *The relation between metalinguistic ability, social metacognition and reading: A developmental study.* New Zealand Journal of Psychology, 1997. **26**(1): p. 20-28.
717. Gordon, H.M., T.D. Lyon, and K. Lee, *Social and cognitive factors associated with children's secret-keeping for a parent.* Child Development, 2014. **85**(6): p. 2374-2388.
718. Grist, R.M. and A.P. Field, *The mediating effect of cognitive development on children's worry elaboration.* Journal of Behavior Therapy & Experimental Psychiatry, 2012. **43**(2): p. 801-7.
719. Sommerville, J.A., D.M. Bernstein, and A.N. Meltzoff, *Measuring beliefs in centimeters: private knowledge biases preschoolers' and adults' representation of others' beliefs.* Child Development, 2013. **84**(6): p. 1846-54.
720. Doherty, M.J., *Children's understanding of homonymy: metalinguistic awareness and false belief.* Journal of Child Language, 2000. **27**(2): p. 367-92.
721. Shaked, M., I. Gamliel, and N. Yirmiya, *Theory of mind abilities in young siblings of children with autism.* Autism, 2006. **10**(2): p. 173-87.
722. Takagishi, H., et al., *Theory of mind enhances preference for fairness.* Journal of Experimental Child Psychology, 2010. **105**(1-2): p. 130-7.
723. Gupta, B., *Theory of mind in autism: A case study.* Psychological Studies, 2015. **60**(3): p. 339-345.
724. Ruffman, T., et al., *Does eye gaze indicate implicit knowledge of false belief? Charting transitions in knowledge.* Journal of Experimental Child Psychology, 2001. **80**(3): p. 201-24.
725. Moll, H., S. Kane, and L. McGowan, *Three-year-olds express suspense when an agent approaches a scene with a false belief.* Developmental Science, 2016. **19**(2): p. 208-20.
726. Gollek, C. and M.J. Doherty, *Metacognitive developments in word learning: Mutual exclusivity and theory of mind.* Journal of Experimental Child Psychology, 2016. **148**: p. 51-69.
727. Papafragou, A., et al., *Learning words from speakers with false beliefs.* Journal of Child Language, 2017. **44**(4): p. 905-923.
728. Powell, L.J. and S. Carey, *Executive function depletion in children and its impact on theory of mind.* Cognition, 2017. **164**: p. 150-162.
729. San Juan, V. and J.W. Astington, *Does language matter for implicit theory of mind? The effects of epistemic verb training on implicit and explicit false-belief understanding.* Cognitive Development, 2017. **41**: p. 19-32.
730. Wimmer, H. and J. Perner, *Beliefs about beliefs: Representation and constraining function of wrong beliefs in young children's understanding of deception.* Cognition, 1983. **13**(1): p. 103-128.
731. Lavoie, J., et al., *Developmental profiles of children's spontaneous lie-telling behavior.* Cognitive Development, 2017. **41**: p. 33-45.
732. Tzuriel, D. and T. Groman, *Dynamic assessment of figurative language of children in the autistic spectrum: The relation to some cognitive and language aspects.* Journal of Cognitive Education and Psychology, 2017. **16**(1): p. 38-63.
733. Bialecka-Pikul, M., et al., *Let's do it together! The role of interaction in false belief understanding.* Journal of Experimental Child Psychology, 2019. **177**: p. 141-151.
734. Fizke, E., et al., *Are there signature limits in early theory of mind?* Journal of Experimental Child Psychology, 2017. **162**: p. 209-224.
735. Grosso, S.S., et al., *33-month-old children succeed in a false belief task with reduced processing demands: A replication of Setoh et al. (2016).* Infant Behavior & Development, 2019. **54**: p. 151-155.
736. Kulke, L., J. Johannsen, and H. Rakoczy, *Why can some implicit Theory of Mind tasks be replicated and others cannot? A test of mentalizing versus submentalizing accounts.* PLoS ONE [Electronic Resource], 2019. **14**(3): p. e0213772.
737. Roby, E. and R.M. Scott, *The relationship between parental mental-state language and 2.5-year-olds' performance on a nontraditional false-belief task.* Cognition, 2018. **180**: p. 10-23.
738. Bernstein, D.M., et al., *False-belief reasoning from 3 to 92 years of age.* PLoS ONE [Electronic Resource], 2017. **12**(9): p. e0185345.

739. D'Esterre, A.P., M.T. Rizzo, and M. Killen, *Unintentional and intentional falsehoods: The role of morally relevant theory of mind.* Journal of Experimental Child Psychology, 2019. **177**: p. 53-69.
740. Kulke, L., et al., *How robust are anticipatory looking measures of theory of mind? Replication attempts across the life span.* Cognitive Development, 2017: p. No Pagination Specified-No Pagination Specified.
741. Bradmetz, J. and R. Schneider, *Is Little Red Riding Hood afraid of her granmdmother? Cognitive vs. emotional response to a false belief.* British Journal of Developmental Psychology, 1999. **17**(4): p. 501-514.
742. Cheung, H., Y. Chan, and W.C. Tsui, *Effect of lie labelling on children's evaluation of selfish, polite, and altruistic lies.* British Journal of Developmental Psychology, 2016. **34**(3): p. 325-39.
743. Woolfe, T., S.C. Want, and M. Siegal, *Siblings and Theory of Mind in Deaf Native Signing Children.* Journal of Deaf Studies and Deaf Education, 2003. **8**(3): p. 340-347.
744. Mayer, A. and B.E. Trauble, *Synchrony in the onset of mental state understanding across cultures? A study among children in samoa.* International Journal of Behavioral Development, 2013. **37**(1): p. 21-28.
745. Woolfe, T., S.C. Want, and M. Siegal, *Signposts to development: theory of mind in deaf children.* Child Development, 2002. **73**(3): p. 768-78.
746. Brown, C.S., *Bias at school: Perceptions of racial/ethnic discrimination among Latino and European American children.* Cognitive Development, 2006. **21**(4): p. 401-419.
747. Brown, C.S., R.S. Bigler, and H. Chu, *An experimental study of the correlates and consequences of perceiving oneself to be the target of gender discrimination.* Journal of Experimental Child Psychology, 2010. **107**(2): p. 100-17.
748. Howe, N., et al., *"The driver doesn't sit, he stands up like the Flintstones!": Sibling teaching during teacher-directed and self-guided tasks.* Journal of Cognition and Development, 2012. **13**(2): p. 208-231.
749. Chandler, M.J. and D. Helm, *Developmental Changes in the Contribution of Shared Experience to Social Role-Taking Competence.* International Journal of Behavioral Development, 1984. **7**(2): p. 145-156.
750. Farrar, M., S. Ashwell, and L. Maag, *The emergence of phonological awareness: Connections to language and theory of mind development.* First Language, 2005. **25**(2): p. 157-172.
751. Farrar, M. and L. Maag, *Early language development and the emergence of a theory of mind.* First Language, 2002. **22**(65,Pt2): p. 197-213.
752. Welch-Ross, M.K., K. Diecidue, and S.A. Miller, *Young children's understanding of conflicting mental representation predicts suggestibility.* Developmental Psychology, 1997. **33**(1): p. 43-53.
753. Riggio, M.M. and K.W. Cassidy, *Preschoolers' processing of false beliefs within the context of picture book reading.* Early Education and Development, 2009. **20**(6): p. 992-1015.
754. Dinolfo, C. and T. Malti, *Interpretive understanding, sympathy, and moral emotion attribution in oppositional defiant disorder symptomatology.* Child Psychiatry & Human Development, 2013. **44**(5): p. 633-45.
755. Welch-Ross, M.K. and P.H. Miller, *Relations between children's theory of mind and a selective attention strategy.* Journal of Cognition and Development, 2000. **1**(3): p. 281-303.
756. Kennedy, K., K.H. Lagattuta, and L. Sayfan, *Sibling composition, executive function, and children's thinking about mental diversity.* Journal of Experimental Child Psychology, 2015. **132**: p. 121-139.
757. Lagattuta, K.H., L. Sayfan, and A.J. Blattman, *Forgetting common ground: six- to seven-year-olds have an overinterpretive theory of mind.* Developmental Psychology, 2010. **46**(6): p. 1417-32.
758. Taylor, M., *Conceptual perspective taking: children's ability to distinguish what they know from what they see.* Child Dev, 1988. **59**(3): p. 703-18.
759. Taylor, M. and B. Hort, *Can children be trained in making the distinction between appearance and reality?* Cognitive Development, 1990. **5**(1): p. 89-99.
760. Watson, A.C. and N.R. Guajardo, *Talking about pretending: Young children's explicit understanding of representation.* Child Study Journal, 2000. **30**(2): p. 127-141.

761. Wellman, H.M., M. Hollander, and C.A. Schult, *Young Children's Understanding of Thought Bubbles and of Thoughts.* Child Development, 1996. **67**(3): p. 768-788.
762. Kolodziejczyk, A.M. and S.L. Bosacki, *Children's understandings of characters' beliefs in persuasive arguments: Links with gender and theory of mind.* Early Child Development and Care, 2015. **185**(4): p. 564-579.
763. Kim, Y.-S. and B. Phillips, *Cognitive correlates of listening comprehension.* Reading Research Quarterly, 2014. **49**(3): p. 269-281.
764. Miller, S.A., *Children's understanding of second-order false belief: Comparisons of content and method of assessment.* Infant and Child Development, 2013. **22**(6): p. 649-658.
765. Kalish, C., M. Weissman, and D. Bernstein, *Taking decisions seriously: young children's understanding of conventional truth.* Child Development, 2000. **71**(5): p. 1289-308.
766. Sullivan, K., D. Zaitchik, and H. Tager-Flusberg, *Preschoolers can attribute second-order beliefs.* Developmental Psychology, 1994. **30**(3): p. 395-402.
767. Lavoie, J., et al., *Polite, instrumental, and dual liars: Relation to children's developing social skills and cognitive ability.* International Journal of Behavioral Development, 2017. **41**(2): p. 257-264.
768. Paine, A.L., et al., *Late, but not early, arriving younger siblings foster firstborns' understanding of second-order false belief.* Journal of Experimental Child Psychology, 2018. **166**: p. 251-265.
769. Perner, J., W. Kain, and P. Barchfeld, *Executive control and higher-order theory of mind in children at risk of ADHD.* Infant and Child Development, 2002. **11**(2): p. 141-158.
770. Ferrell, J.M., R.E. Guttentag, and J.M. Gredlein, *Children's understanding of counterfactual emotions: Age differences, individual differences, and the effects of counterfactual-information salience.* British Journal of Developmental Psychology, 2009. **27**(3): p. 569-585.
771. Lecce, S., et al., *Social competence assessed by teachers and parents: The role of theory of mind.* Eta Evolutiva, 2010. **96**: p. 5-14.
772. Recchia, H.E. and N. Howe, *Associations between social understanding, sibling relationship quality, and siblings' conflict strategies and outcomes.* Child Development, 2009. **80**(5): p. 1564-1578.
773. Astington, J.W., J. Pelletier, and B. Homer, *Theory of mind and epistemological development: The relation between children's second-order false-belief understanding and their ability to reason about evidence.* New Ideas in Psychology, 2002. **20**(2-3): p. 131-144.
774. Filippova, E. and J.W. Astington, *Further development in social reasoning revealed in discourse irony understanding.* Child Development, 2008. **79**(1): p. 126-38.
775. Qualter, P., A. Barlow, and M.S. Stylianou, *Investigating the relationship between trait and ability emotional intelligence and theory of mind.* British Journal of Developmental Psychology, 2011. **29**(Pt 3): p. 437-54.
776. Bradmetz, J., *Hermeneutics and false beliefs in children three to six years of age.* Archives de Psychologie, 1999. **67**(261): p. 71-94.
777. Dyck, M., K. Ferguson, and I. Shochet, *Do autism spectrum disorders differ from each other and from non-spectrum disorders on emotion recognition tests?* European Child & Adolescent Psychiatry, 2001. **10**(2): p. 105-116.
778. McKown, C., et al., *Direct assessment of children's social-emotional comprehension.* Psychological Assessment, 2013. **25**(4): p. 1154-1166.
779. Whyte, E.M., K.E. Nelson, and K. Scherf, *Idiom, syntax, and advanced theory of mind abilities in children with autism spectrum disorders.* Journal of Speech, Language, and Hearing Research, 2014. **57**(1): p. 120-130.
780. Kirk, E., et al., *A longitudinal investigation of the relationship between maternal mind-mindedness and theory of mind.* British Journal of Developmental Psychology, 2015. **33**(4): p. 434-445.
781. McKown, C., et al., *Social-emotional learning skill, self-regulation, and social competence in typically developing and clinic-referred children.* Journal of Clinical Child & Adolescent Psychology, 2009. **38**(6): p. 858-71.
782. O'Hare, A.E., et al., *A clinical assessment tool for advanced theory of mind performance in 5 to 12 year olds.* Journal of Autism and Developmental Disorders, 2009. **39**(6): p. 916-928.

783. Wellman, H.M. and J.D. Woolley, *From simple desires to ordinary beliefs: The early development of everyday psychology.* Cognition, 1990. **35**(3): p. 245-275.
784. Raisa, Z.H., et al., *Thinking about what he thinks of what I think: Assessing higher theory of mind abilities in Indian bilingual children between 3.0 and 8.11 years of age.* Indian Journal of Psychiatry, 2019. **61**(2): p. 167-176.
785. van Dijk, A., A.M.G. Poorthuis, and T. Malti, *Psychological processes in young bullies versus bully-victims.* Aggressive Behavior, 2017: p. No Pagination Specified-No Pagination Specified.
786. Tager-Flusberg, H. and K. Sullivan, *Attributing mental states to story characters: A comparison of narratives produced by autistic and mentally retarded individuals.* Applied Psycholinguistics, 1995. **16**(3): p. 241-256.
787. Lapan, C. and J.J. Boseovski, *Theory of mind and children's trait attributions about average and typically stigmatized peers.* Infant and Child Development, 2016. **25**(2): p. 158-178.
788. Ryan, N.P., et al., *The emergence of age-dependent social cognitive deficits after generalized insult to the developing brain: a longitudinal prospective analysis using susceptibility-weighted imaging.* Human Brain Mapping, 2015. **36**(5): p. 1677-91.
789. Dyck, M.J., K. Ferguson, and I.M. Shochet, *Do autism spectrum disorders differ from each other and from non-spectrum disorders on emotion recognition tests?* European Child & Adolescent Psychiatry, 2001. **10**(2): p. 105-16.
790. Russo-Ponsaran, N.M., et al., *Social-emotional correlates of early stage social information processing skills in children with and without autism spectrum disorder.* Autism Research, 2015: p. No Pagination Specified.
791. McKown, C., et al., *Web-based assessment of children's social-emotional comprehension.* Journal of Psychoeducational Assessment, 2016. **34**(4): p. 322-338.
792. Pujals, E., et al., *Brief Report: Translation and Adaptation of the Theory of Mind Inventory to Spanish.* Journal of Autism & Developmental Disorders, 2016. **46**(2): p. 685-90.
793. Hutchins, T.L., et al., *Mothers' and Children's Story-Telling: A Study of Dyads with Typically Developing Children and Children with ASD.* Journal of Autism & Developmental Disorders, 2017. **47**(5): p. 1288-1304.
794. Greenslade, K.J. and T.E. Coggins, *Brief Report: An Independent Replication and Extension of Psychometric Evidence Supporting the Theory of Mind Inventory.* Journal of Autism & Developmental Disorders, 2016. **46**(8): p. 2785-90.
795. Dawes, E., et al., *A profile of the language and cognitive skills contributing to oral inferential comprehension in young children with developmental language disorder.* International Journal of Language & Communication Disorders, 2018. **53**(6): p. 1139-1149.
796. Xiao, Y., et al., *Neural correlates of developing theory of mind competence in early childhood.* Neuroimage, 2019. **184**: p. 707-716.
797. Bartoli, G., D. Bulgarelli, and P. Molina, *Theory of Mind Development in Children with Visual Impairment: The Contribution of the Adapted Comprehensive Test ToM Storybooks.* Journal of Autism & Developmental Disorders, 2019. **49**(9): p. 3494-3503.
798. Braams, O.B., et al., *Epilepsy surgery in children: no further threat to theory of mind.* Epileptic Disorders, 2019. **21**(2): p. 166-176.
799. Broeren, S. and P. Muris, *The relation between cognitive development and anxiety phenomena in children.* Journal of Child and Family Studies, 2009. **18**(6): p. 702-709.
800. Muris, P., et al., *Children's internal attributions of anxiety-related physical symptoms: Age-related patterns and the role of cognitive development and anxiety sensitivity.* Child Psychiatry and Human Development, 2010. **41**(5): p. 535-548.
801. Muris, P., et al., *Theory-of-mind, cognitive development, and children's interpretation of anxiety-related physical symptoms.* Behaviour Research & Therapy, 2007. **45**(9): p. 2121-32.
802. Broeren, S., et al., *The course of childhood anxiety symptoms: developmental trajectories and child-related factors in normal children.* Journal of Abnormal Child Psychology, 2013. **41**(1): p. 81-95.
803. Smeekens, S., J. Riksen-Walraven, and H.J. van Bakel, *Profiles of competence and adaptation in preschoolers as related to the quality of parent-child interaction.* Journal of Research in Personality, 2008. **42**(6): p. 1490-1499.

804. Colonnesi, C., et al., *Social Anxiety Symptoms in Young Children: Investigating the Interplay of Theory of Mind and Expressions of Shyness.* Journal of Abnormal Child Psychology, 2017. **45**(5): p. 997-1011.
805. Rosenqvist, J., et al., *Preschoolers' recognition of emotional expressions: relationships with other neurocognitive capacities.* Child Neuropsychology, 2014. **20**(3): p. 281-302.
806. Loukusa, S., et al., *Theory of mind and emotion recognition skills in children with specific language impairment, autism spectrum disorder and typical development: group differences and connection to knowledge of grammatical morphology, word-finding abilities and verbal working memory.* International Journal of Language & Communication Disorders, 2014. **49**(4): p. 498-507.
807. Rosenqvist, J., et al., *Neurocognitive Functions in 3- to 15-Year-Old Children: An International Comparison.* Journal of the International Neuropsychological Society, 2017. **23**(4): p. 367-380.
808. Korkman, M., Kirk, U., Kemp, S., *NEPSY-II. Manuel clinique et d'interprétation, Version française*. 2012, Montreuil, France: PEARSON FRANCE-ECPA.
809. Bouchand, J. and J. Caron, *Production of mental verbs and acquisition of a theory of mind by young children.* Enfance, 1999. **51**(3): p. 225-237.
810. Narzisi, A., et al., *Neuropsychological profile in high functioning autism spectrum disorders.* Journal of Autism and Developmental Disorders, 2013. **43**(8): p. 1895-1909.
811. Ogelman, H.G., Z. Secer, and A. Onder, *Analyzing perspective taking skills of 5- to 6-year-old preschool children in relation to their self-perception and gender.* Journal of Research in Childhood Education, 2013. **27**(4): p. 427-439.
812. Korkman, M., Kirk, U, Kemp, S., *Clinical and interpretive manual NEPSY-II*. 2007, San Antonio, TX: Harcourt Assessment.
813. Sivaratnam, C.S., et al., *Brief report: assessment of the social-emotional profile in children with autism spectrum disorders using a novel comic strip task.* Journal of Autism & Developmental Disorders, 2012. **42**(11): p. 2505-12.
814. Tager-Flusberg, H. and K. Sullivan, *A second look at second-order belief attribution in autism.* Journal of Autism & Developmental Disorders, 1994. **24**(5): p. 577-86.
815. Parker, K.J., et al., *Plasma oxytocin concentrations and OXTR polymorphisms predict social impairments in children with and without autism spectrum disorder.* PNAS Proceedings of the National Academy of Sciences of the United States of America, 2014. **111**(33): p. 12258-12263.
816. Carson, D.S., et al., *Arginine Vasopressin Is a Blood-Based Biomarker of Social Functioning in Children with Autism.* PLoS ONE [Electronic Resource], 2015. **10**(7): p. e0132224.
817. Pino, M.C., et al., *Simple mindreading abilities predict complex theory of mind: Developmental delay in autism spectrum disorders.* Journal of Autism and Developmental Disorders, 2017: p. No Pagination Specified-No Pagination Specified.
818. Loukusa, S., et al., *Assessing social-pragmatic inferencing skills in children with autism spectrum disorder.* Journal of Communication Disorders, 2018. **73**: p. 91-105.
819. Serra, A., et al., *Adaptive psychological structure in childhood hearing impairment: audiological correlations.* Acta Otorhinolaryngologica Italica, 2017. **37**(3): p. 175-179.
820. Payne, J.M., et al., *Theory of mind in children with Neurofibromatosis Type 1.* Neuropsychology, 2016. **30**(4): p. 439-48.
821. Hong, D.S., B. Dunkin, and A.L. Reiss, *Psychosocial functioning and social cognitive processing in girls with Turner syndrome.* Journal of Developmental & Behavioral Pediatrics, 2011. **32**(7): p. 512-20.
822. Porter, M.A., M. Coltheart, and R. Langdon, *Theory of mind in Williams syndrome assessed using a nonverbal task.* Journal of Autism & Developmental Disorders, 2008. **38**(5): p. 806-14.
823. Krcmar, M. and E.T. Vieira, Jr., *Imitating Life, Imitating Television: The Effects of Family and Television Models on Children's Moral Reasoning.* Communication Research, 2005. **32**(3): p. 267-294.
824. Lagattuta, K.H. and L. Sayfan, *Developmental changes in children's understanding of future likelihood and uncertainty.* Cognitive Development, 2011. **26**(4): p. 315-330.

825. Lagattuta, K.H., N.M. Elrod, and H.J. Kramer, *How do thoughts, emotions, and decisions align? A new way to examine theory of mind during middle childhood and beyond.* Journal of Experimental Child Psychology, 2016. **149**: p. 116-133.
826. Davis, P.E., E. Meins, and C. Fernyhough, *Self-knowledge in childhood: relations with children's imaginary companions and understanding of mind.* British Journal of Developmental Psychology, 2011. **29**(Pt 3): p. 680-6.
827. Meins, E., et al., *Mind-mindedness and theory of mind: Mediating roles of language and perspectival symbolic play.* Child Development, 2013. **84**(5): p. 1777-1790.
828. Wade, M., et al., *Cumulative risk disparities in children's neurocognitive functioning: a developmental cascade model.* Developmental Science, 2016. **19**(2): p. 179-94.
829. Wade, M., et al., *Oxytocin and vasopressin hormone genes in children's externalizing problems: A cognitive endophenotype approach.* Hormones & Behavior, 2016. **82**: p. 78-86.
830. Dawes, E., et al., *A profile of the language and cognitive skills contributing to oral inferential comprehension in young children with developmental language disorder.* International Journal of Language & Communication Disorders, 2018. **53**(6): p. 1139-1149.
