# Supplementary material for: Systematic Review and Inventory of Theory of Mind Measures for Young Children
Source: Front Psychol. 2020 Jan 15;10:2905. doi: 10.3389/fpsyg.2019.02905 (PMC6974541; doi:10.3389/fpsyg.2019.02905)
Supplement: Supplementary file 2 [file Table_2.pdf]

Appendix III

Table i.

Psychometric properties of included measures (direct testing)

Measures (source author, year)	Studies	Implicit/explicit methods	Internal structure and consistency	Inter-rater reliability	Test-retest reliability	Other psychometric information
Category: Emotions understanding						
Affective perspective taking (Cassidy, Parke, Butkovsky, and Braugart, 1992)	Cassidy, Parke, Butkovsky, & Braungart (1992)	Explicit	Structure analyses were performed	Cohen's κ = 0.78-1.0	-	-
	Cutting & Dunn (2006)	Explicit	-	Cohen's κ = 0.87.	-	-
	Dunn, Cutting, & Demetriou (2000)	Explicit	Cronbach's α =.73	-	-	-
	Guajardo, Parker, and Turley-Ames (2009)	Explicit	-	Cohen's κ = .92	-	-
	Shields et al. (2001)	Explicit	Cronbach's α = .66-.74	Cohen's κ = .78-.80	-	-
Affective perspective-taking tests (Denham, 1986)	Cutting & Dunn (2006)	Explicit	Cronbach's α = .81	-	-	-
	Dunn et al. (2000)	Explicit	Cronbach's α = .73	-	-	-
	O'Kearney, Salmon, Liwag, Fortune, & Dawel (2017)	Explicit	-	% agreement = 100%; r = .75-.89	-	-
	Shields et al. (2001)	Explicit	Cronbach's α = .75	-	-	-
	Tarullo et al. (2016)	Explicit	Cronbach's α = .75	-	-	-
Knowledge of emotion cause (Denham, Zoller & Couchoud, 1994)	Blankson et al. (2017)	Explicit	-	Cohen's κ = .76-.83	-	-
Description of emotional situation (Feshbach & Cohen, 1988)	Feshbach & Cohen (1988)	Explicit	-	% agreement = 100%	-	-

Appendix III

Emotion situation knowledge task (Garner et al., 1994)	Shields et al. (2001)	Explicit	Cronbach's α: .66	-	-	Associated with prosocial behavior and emotion recognition
Mixed emotion understanding task (Gordis, Rosen, and Grand, 1989)	Cutting & Dunn (2002)	Explicit	Cronbach's α = .74	-	-	-
	Lecce, Caputi, & Pagnin (2014)	Explicit	Cronbach's α = .69	-	-	-
	Lecce & Hughes (2010)	Explicit	-	Cohen's κ = .86	-	-
Appearance reality of emotions (Harris, Donnelly, Guz, and Pitt-Watson, 1986); Affective false-belief task (Davis, 1998)	Banerjee & Yuill (1999)	Explicit	-	% agreement = 90%; Cohen's κ = .86	-	-
	Hiller, Weber, & Young (2014)	Explicit	-	% agreement = 100%	-	-
	Olineck & Poulin-Dubois (2007)	Explicit	-	% agreement = 100%	-	-
	Shahaeian, Nielsen, Peterson, & Slaughter (2014)	Explicit	-	Cohen's κ = .89	-	-
	Tarullo et al. (2016))	Explicit	-	% agreement = 97%	-	-
Emotion understanding assessment (Howlin, Baron-Cohen, & Hadwin, 1999)	Barbosa-Leiker, Strand, Mamey, & Downs (2014)	Explicit	Structure analyses were performed	-	« Longitudinal latent means analysis revealed nonsignificant differences over time »	-
	Downs, Strand, & Cerna (2007)	Explicit	Cronbach's α: .85-.89	-	*r* = .68	Associated with prosocial behavior<br><br>Scores and psychometric properties for 2 levels of verbal abilities available
	Strand, Downs, and Barbosa-Leiker (2016)	Explicit	Cronbach's α: ≥ .69	-	-	-
Affective attribution and reasoning task (Iannotti, 1978)	Iannotti (1978)	Explicit	Cronbach's α = .37-.55	% agreement = 91-100%	-	-

Appendix III

Test of emotion comprehension (Pons & Harris, 2000)	De Rosnay, Fink, Begeer, Slaughter, & Peterson (2014)	Explicit	Cronbach's α = .42	-	-	-
	Kårstad, Wichstrøm, Reinfjell, Belsky, & Berg-Nielsen (2015)	Explicit	Theta test : .82-.91	-	-	-
	Lecce, Demicheli, Zocchi, & Palladino (2015)	Explicit	Cronbach's α = .74	-	-	-
	Pons, Harris, & de Rosnay (2004)	Explicit	-	-	-	Scaling analyses were performed,; associated with age
	Ornaghi, Pepe, & Grazzani (2016)	Explicit	Cronbach's α = .69	-	-	-
Emotion recognition questionnaire (Ribordy, Camras, Stefani, & Spaccarelli, 1988)	Ben-Israel, Uzefovsky, Ebstein, & Knafo-Noam (2015)	Explicit	Structure analyses were performed	-	-	-
	Knafo, Steinberg, & Goldner (2011)	Explicit	Structure analyses were performed<br>Cronbach's α = .58-.73	-	-	-
	Knafo et al. (2009)	Explicit	Structure analyses were performed	-	-	-
	Martins, Osório, Veríssimo, & Martins (2016)	Explicit	Cronbach's α = .69	-	-	-
Category: Desires understanding						
Diverse desire (Bartsch & Wellman, 1989)	Bartsch & Wellman (1989)	Explicit		Cohen's κ = .82-.88		-
Gift Task (Flavell, 1968)/ Gift selection task (Jin et al, 2017)	Jin, Li, He, & Shen (2017)	Implicit/Explicit	-	Cohen's κ = .96-1.0	-	-
Discrepant desires Yummy-yucky task (Repacholi & Gopnik, 1997)	Fidler, Hepburn, Most, Philofsky, and Rogers (2007)	Implicit	-	% agreement: 80-100%	-	-
	Poulin-Dubois & Yott (2018)	Implicit	-	-	-	Replicability testing suggesting partial replicability; convergent validity testing between similar tasks suggesting poor validity

	Sodian, Licata, Kristen-Antonow, et al. (2016)	Implicit	-	Cohen's κ = .92	-	-
Common and uncommon desires (Rieffe et al., 2001)	Rieffe, Ketelaar, & Wiefferink (2010)	Implicit	Cronbach's α = .77	-	-	-
Desire and intention task (Schult, 2002)	Schult (2002)	Explicit	-	% agreement = 83-97%	-	-
	Shiverick & Moore (2013)	Explicit	-	Cohen's κ = .70	-	-
Target-hitting game (Schult, 2002)	Schult (2002)	Implicit		% agreement = 92%		
Not own desire tasks (Wellman & Wooley, 1990)	Hanson & Atance (2014)	Explicit	-	Cohen's κ = 1.0	-	-
	Hanson, Atance, & Paluck (2014)	Explicit	-	Cohen's κ = .81	-	-
	Olineck & Poulin-Dubois (2007)	Explicit	-	% agreement = 100%	-	-
	Simcock et al. (2017)	Explicit	-	Intraclass correlation = 0.97	-	-
Desire task (actions and emotions stories) (Wellman & Woolley, 1990)	Grazzani, Ornaghi, & Brockmeier (2016)	Explicit	Cronbach's α = .73-.79	-	-	-
Category: Intention understanding						
Behavior-, skill- and awareness-intentionality measures (Astington & Lee, 1991)	Mull & Evans (2010)	Explicit	-	% agreement = 94 %	-	-
Visual habituation paradigm (Buresh & Woodward, 2007)	Buresh & Woodward (2007)	Implicit	-	% agreement = 92-94%	-	-
	Sodian, Licata, Kristen-Antonow, et al. (2016)	Implicit	-	Only children with an interrater correlation of $r \geq .90$ were included in analysis	-	-
Intention and beliefs (Choi & Luo, 2015)	Choi & Luo (2015)	Implicit	-	% agreement = 95%	-	-

Appendix III

Behavioral re-enactment procedure (Meltzoff, 1995)	Bellagamba, Laghi, Lonigro, & Pace (2012)	Implicit	-	% agreement = 97%	-	-
	Camaioni, Perucchini, Bellagamba, & Colonnesi (2004)	Implicit	-	Cohen's κ = .91-1.00	-	-
	Colonnesi, Rieffe, Koops, & Perucchini (2008)	Implicit	-	Cohen's κ = 1.00	-	-
	Hahn, Fidler, Hepburn, & Rogers (2013)	Implicit	-	Cohen's κ = .84	-	-
	Kristen, Sodian, Thoermer, & Perst (2011)	Implicit	-	Cohen's κ = .95 to 1.00	-	-
	Meltzoff (1995)	Implicit	-	Pearson *r* = .98	-	No intra-scorer disagreements
	Poulin-Dubois & Yott (2018)	Implicit	-	-	-	Replicability testing suggesting good replicability; convergent validity testing between similar tasks suggesting poor validity
Accidental transgression task (MoToM; Killen, Lynn Mulvey, Richardson, Jampol & Woodward, 2011)	Sodian, Licata, Kristen-Antonow, et al. (2016)	Explicit	-	Cohen's κ = .96-1.0	-	-
Intention task (Phillips & Wellman, 2005)	Phillips & Wellman (2005)	Implicit	-	% agreement = 79-100%	-	-
	Poulin-Dubois & Yott (2018)	Implicit	-	-	-	Replicability testing suggesting good replicability; convergent validity testing between similar tasks suggesting poor validity
Attention to intention (Phillips, Wellman & Spelke, 2002)	Phillips, Wellman, & Spelke (2002)	Implicit	-	% agreement = 83-100%	-	-
Category: Percepts						
Visual perspective taking and spatial construction task (Ebersbach, Stiehler & Asmus, 2011)	Ebersbach, Stiehler, & Asmus (2011)	Explicit	-	Cohen's κ = .75	-	-

Appendix III

Photographers perspective taking (Frick, Mohring & Newcombe, 2014)	Frick, Mohring, & Newcombe (2014)	Explicit	Guttman's split-half coefficient = .91	-	-	-
Penny game task (Gratch, 1964)	Chasiotis, Kiessling, Hofer, & Campos (2006)	Implicit	Cronbach's α = .87	-	-	-
	Chasiotis, Kiessling, Winter, & Hofer (2006)	Implicit	Cronbach's α = .87	-	-	-
	Flynn (2006)	Implicit	-	% agreement $\geq$ 97%	-	-
	Gratch (1964)	Implicit	-	-	-	Scaling analyses were performed
Perception based action (Hadwin, Baron-Cohen, Howlin & Hill, 1996)	Hutchins, Prelock, & Chace (2008)	Explicit	-	-	Kappa = 0.133-1.0	-
Gaze-following task (Meltzoff & Brooks, 2008)	Meltzoff & Brooks (2008)	Implicit	-	Cohen's $\kappa \geq .90$	-	Intra-rater agreement: $\kappa \geq .90$
Occluded object task (Moll and Tomasello, 2006)	Herold & Akhtar (2008)	Implicit	-	Cohen's κ = .88	-	-
	Moll & Tomasello (2006)	Implicit	-	Cohen's κ = 1	-	-
Level-1 perspective taking tasks (Ricard et al., 1999)	Ricard, Girouard, & Decarie (1999)	Implicit	-	Cohen's κ = .73-1.0	-	-
Category: Knowledge						
Cognitive perspective taking (Brice & Torney- Purta, 1981)	Denham (1986)	Explicit	Cronbach's α = .61	-	-	-
Cognitive perspective taking (Flavell, Botkin, Fry, Wright, and Jarvis, 1968)	Carlo, Knight, Eisenberg, & Rotenberg (1991)	Explicit	-	% agreement = 92%	-	-
Familiary-focus of attention (Moll, Koring, Carpenter & Tomasello, 2006)	Moll, Koring, Carpenter, & Tomasello (2006)	Implicit	-	Cohen's κ = .82-.93	-	-
Knowledge theory of mind task (Moll & Tomasello, 2007)	Moll & Tomasello (2007)	Implicit	-	Cohen's κ = 1	-	-

Appendix III

	Crivello, Phillips, & Poulin-Dubois (2018)	Implicit	-	Cohen's κ = .88	-	-
See-know task (Pillow, 1989; Ruffman and Olson, 1989)	Fedra & Schmidt (2019)	Explicit	-	Cohen's κ = 1.0	-	-
	Hanson & Atance (2014)	Explicit	-	Cohen's κ = 1.0	-	-
	Hanson et al. (2014)	Explicit	-	Cohen's κ = .82	-	-
	Hutchins, Bonazinga, Prelock, & Taylor (2008)	Explicit	-	-	Kappa = 1.0	-
	Martins et al. (2016)	Explicit	-	Cohen's κ ≥ .88	-	-
	Olineck & Poulin-Dubois (2007)	Explicit	-	% agreement = 100%	-	-
Hide an object (Viranyi, Topal, Miklosi, & Csanyi 2006)	Viranyi, Topal, Miklosi, & Csanyi (2006)	Implicit	-	Cohen's κ = .90-.99	-	-
Category: Beliefs understanding						
Deceptive contents FB task (Bartsch & Wellman, 1989)	Flynn (2006)	Explicit	-	% agreement ≥ 97%	-	-
	Hwa-Froelich, Matsuo, & Jacobs (2017)	Explicit	-	% agreement ≥ 82.6	-	Intrarater reliability ≥ 82.6%
	Lane, Wellman, Olson, LaBounty, & Kerr (2010)	Explicit	Cronbach's α = .68-.80	-	-	-
	Olson, Choe, & Sameroff (2017)	Explicit	Cronbach's α = .80	-	-	-
Picture false-belief task (Callaghan, Rochat & Corbit, 2012)	Callaghan, Rochat, & Corbit (2012)	Explicit	-	% agreement = 100%	-	-
Lexical ambiguity (Carpendale & Chandler, 1996)	Howe, Recchia, Porta, & Funamoto (2012)	Explicit	-	Cohen's κ > .86	-	-

Appendix III

Droodle task (Chandler & Helm, 1984; Hughes, Dunn & White 1998)	Chandler & Helm (1984)	Explicit	-	*r* = 0.98	-	-
	Grosso, Schuwerk, Kaltefleiter, & Sodian (2019)	Explicit	-	Cohen's κ = 0.96	-	Replicability testing suggesting poor replicability
Appearance-reality tasks (Flavell, 1986)	Flynn (2006)	Explicit	-	% agreement ≥ 97%	-	-
Ella the Elephant or Emotion false belief task (Harris, Johnson, Hutton, Andrews, & Cooke, 1989)	Hughes et al. (2000)	Explicit	-	-	Test-retest, κ = .43-.56	-
Content false belief paradigm (Hogrefe, Wimmer, & Perner, 1986; Perner, Leekam, & Wimmer, 1987)	Buttelmann, Over, Carpenter, & Tomasello (2014)	Implicit	-	% agreement = 100%	-	-
	Atkinson, Slade, Powell, & Levy (2017)	Explicit	-	Cohen's κ = 1.0	-	-
	Bellagamba et al. (2015)	Explicit	-	% agreement = 100%	-	-
	Bialecka-Pikul et al. (2019)	Explicit	-	Cohen's κ = 1.0	-	-
	Callaghan et al. (2012)	Explicit	-	% agreement = 100%	-	-
	Flynn (2006)	Explicit	-	% agreement ≥97%	-	-
	Hanson & Atance (2014)	Explicit	-	Cohen's κ = 1.0	-	-
	Hanson et al. (2014)	Explicit	-	Cohen's κ = 1.0	-	-
	Hughes et al. (2000)	Explicit	-	-	Test-retest, κ = .29-.53	-
	Hwa-Froelich et al. (2017)	Explicit	-	% agreement ≥ 82.6%	-	Intrarater reliability ≥ 82.6%

Appendix III

	Lackner, Sabbagh, Hallinan, Liu, & Holden (2012)	Explicit	-	% agreement ≥ 95%	-	-
	Martins et al. (2016)	Explicit	-	Cohen's κ ≥ .88	-	-
	Olineck & Poulin-Dubois (2007)	Explicit	-	% agreement = 100%	-	-
	Sodian & Kristen-Antonow (2015)	Explicit	-	% agreement = 100%	-	-
	Symons, Fossum, & Collins (2006)	Explicit	Cronbach's α = .84	-	-	-
	Symons, Peterson, Slaughter, Roche, & Doyle (2005)	Explicit	Cronbach's α = .78	-	-	-
Battery of TOM tasks (Hughes, Adlam, Happe, Jackson, Taylor & Caspi, 2000)	Hughes et al. (2000)	Explicit	Cronbach's α = .60-.82	-	*r* = .66-.77	-
	Lecce & Hughes (2010)	Explicit	-	Cohen's κ = .86	-	-
	van Dijk, Poorthuis, & Malti (2017)	Explicit	Cronbach's α = .69	-	-	-
ToM task (Kim & Phillips, 2014)	Kim & Phillips (2014)	Explicit	Cronbach's α = .71-.73	-	-	-
Message-desire discrepancy (Mitchell, Saltmarsh & Russell, 1997)	Hutchins, Prelock, et al. (2008)	Explicit	-	-	Kappa = 0.760	-
False-belief suspense (Moll, Kane & McGowan, 2016)	Moll, Kane, & McGowan (2015)	Implicit	-	Cohen's κ = .80-.90	-	-
Ice-cream van test (Perner & Wimmer, 1985)	Hughes et al. (2000)	Explicit	-	-	Test-retest, κ = .48	-
	{Paine, 2018 #823}	Explicit	-	Cohen's κ = .79-1.0	-	-

Appendix III

	Pearson, Marsh, Ropar, & Hamilton (2016)	Explicit	-	Cohen's κ = .79	-	-
False belief story (Riggio & Cassidy 2009)	Riggio & Cassidy (2009)	Explicit	-	Cohen's κ = .72- 1.0	-	-
Birthday puppy (Sullivan, Zaitchik, & Tager- Flusberg, 1994)	Banerjee & Yuill (1999)	Explicit	-	% agreement = 100%	-	-
Granddad story, Window story or Tom's crayon (Sullivan, Zaitchik, & Tager-Flusberg, 1994; Astington, Pelletier & Homer, 2002)	Filippova & Astington (2008)	Explicit	-	Cohen's κ ≥ .78	-	-
	Howe et al. (2012)	Explicit	-	Cohen's κ = .92	-	-
	Hughes et al. (2000)	Explicit	-	-	Test-retest, κ= .40-.72	-
	Hutchins, Prelock, et al. (2008)	Explicit	-	-	Kappa = 0.302	-
	Kolodziejczyk & Bosacki (2015)	Explicit	Cronbach's α =.79	-	-	-
Ambiguity task (Taylor, 1988)	Kennedy, Lagattuta, & Sayfan (2015)	Explicit	-	Cohen's κ = .84-.95	-	-
	Lagattuta, Sayfan, & Blattman (2010)	Explicit	-	Cohen's κ = .84-.95	-	-
False-belief explanation task (Villiers & de Villiers, 2000)	Ornaghi et al. (2016)	Explicit	Cronbach's α = .60	-	-	-
Belief tasks (Wellman & Bartsch, 1988)	Flynn (2006)	Explicit	-	% agreement ≥ 97%	-	-
	Hanson & Atance (2014)	Explicit	-	Cohen's κ = 1.0	-	-
	Hanson et al. (2014)	Explicit	-	Cohen's κ = .94	-	-

Appendix III

	Martins et al. (2016)	Explicit	-	Cohen's κ ≥ .88	-	-
	Martins et al. (2016)	Explicit	-	Cohen's κ ≥ .78	-	-
	Olineck & Poulin-Dubois (2007)	Explicit	-	% agreement = 100%	-	-
	Sodian & Kristen-Antonow (2015)	Explicit	-	% agreement = 100%	-	-
Change-in-location paradigm (Wimmer & Perner, 1983)/Sally-Ann task, (Baron-Cohen, Leslie & Frith, 1985)	Bialecka-Pikul et al. (2019)	Explicit	-	Cohen's κ = 0.79-0.88	-	-
	Burnside, 2017 #842}	Implicit	-	Cohen's κ = 0.82	-	-
	Burnside, 2018 #730}	Implicit	-	Cohen's κ = 0.82	-	-
	Buttelmann, Carpenter, & Tomasello (2009)	Implicit	-	% agreement = 100%	-	-
	Carpenter, Call, & Tomasello (2002)	Implicit	-	% agreement = 100% *r* = .98	-	-
	Dörrenberg, Rakoczy, & Liszkowski (2018)	Implicit	-	Cohen's κ = 0.94	-	Replicability testing suggesting poor replicability; convergent validity testing between similar tasks suggesting poor validity
	Dorrenberg, Wenzel, Proft, Rakoczy, & Liszkowski (2019)	Implicit & explicit	-	-	-	Replicability testing suggesting poor replicability
	Poulin-Dubois & Yott (2018)	Implicit	-	-	-	Replicability testing suggesting poor replicability; convergent validity testing between similar tasks suggesting poor validity
	Poulin-Dubois & Yott (2014)	Implicit	-	*r* = 0,96	-	-

Appendix III

Scott, He, Baillargeon, & Cummins (2012)	Implicit	-	% agreement = 94-97%	-	-
Garnham & Ruffman (2001)	Implicit & explicit	-	% agreement = 100% (implicit data)	-	-
Atkinson et al. (2017)	Explicit	-	Cohen's κ = .92	-	-
Callaghan et al. (2005)	Explicit	-	% agreement = 100%	-	-
Callaghan et al. (2012)	Explicit	-	% agreement = 100%	-	-
Cheung (2006)	Explicit	Kuder-Richardson 20 formula = .86	-	-	-
Flynn (2006)	Explicit	-	% agreement ≥ 97% Cohen's κ = .89	-	-
Grosso et al. (2019)	Explicit	-	Cohen's κ = 1.0	-	Replicability testing suggesting good replicability
Hanson & Atance (2014)	Explicit	-	Cohen's κ = 1.0	-	-
Hanson et al. (2014)	Explicit	-	Cohen's κ = 1.0	-	-
Hughes et al. (2000)	Explicit	-	-	Cohen's κ = .62	-
Hutchins, Prelock, et al. (2008)	Explicit	-	-	Kappa = 0.325-0.849	-
Hwa-Froelich et al. (2017)	Explicit	-	% agreement ≥ 82.6%	-	Intra-rater reliability ≥ 82.6 %
Kulke, Johannsen, & Rakoczy (2019)	Implicit	-	-	-	Replicability testing suggesting poor replicability

Appendix III

	Kulke, Reiß, Krist, & Rakoczy (2017)	Implicit and explicit	-	-	-	Replicability testing suggesting partial replicability; convergent validity testing suggesting poor validity
	Lackner et al. (2012)	Explicit	-	% agreement ≥ 95%	-	-
	Martins et al. (2016)	Explicit	-	Cohen's κ ≥ .88	-	-
	Mayes, Klin, Tercyak, Cicchetti, & Cohen (1996)	Explicit	-	-	Cohen's κ = 0-1.0	-
	Moll et al. (2015)	Explicit	-	Cohen's κ = 1.0	-	-
	Putko & Zlotogorska (2014)	Explicit	-	Cohen's κ = .89	-	-
	Roby & Scott (2018)	Implicit	-	% agreement = 95%	-	-
	Symons et al. (2005)	Explicit	Cronbach's α = .40	-	-	-
	Symons et al. (2006)	Explicit	Cronbach's α = .51	-	-	-
Category: Mentalistic understanding of non-literal communication						
Irony task (Filippova & Astington, 2008)	Filippova & Astington (2008)	Explicit	-	Cohen's κ = .82-1.0	-	-
Joke stories from the Strange stories (Happé, 1994)	Lapan & Boseovski (2016)	Explicit	-	α = .92	-	-
Sarcasm stories from the Strange stories (Happé, 1994)	Peterson, Wellman, & Slaughter (2012)	Explicit	-	% agreement = 95%	-	-
	Shahaeian et al. (2014)	Explicit	-	Cohen's κ = .97	-	-
Strange stories (Happé, 1994)	Dyck, Ferguson, & Shochet (2001)	Explicit	Cronbach's α = .85	-	-	-

Appendix III

	Filippova & Astington (2008)	Explicit	-	Cohen's κ = .78-.98	-	-
	Lapan & Boseovski (2016)	Explicit	-	α = .92-95	-	-
	Lecce, Bianco, Devine, Hughes, & Banerjee (2014)	Explicit	-	Cohen's κ = .76-.84	-	-
	Lecce, Caputi, et al. (2014)	Explicit	Cronbach's α = .67	Cohen's κ = .93	-	-
	Peterson et al. (2012)	Explicit	-	% agreement= 97%	-	-
	Russo-Ponsaran et al. (2015)	Explicit	Cronbach's α = .74-.82	Cohen's κ = .72	-	-
White lies stories from the Strange stories (Happé, 1994)	Lapan & Boseovski (2016)	Explicit	-	α = .95	-	-
Recognition of faux pas (Baron-Cohen, O'Riordan, Stone, Jones, & Plaisted, 1999)	Filippova & Astington (2008)	Explicit	-	Cohen's κ = .78-.98	-	-
	Smogorzewska, Szumski, & Grygiel (2018)	Explicit	Structure analyzes were performed; Cronbach's *α* = .67-.83	-	-	Associated with other TOM tests, social functioning and language abilities
	Smogorzewska, Szumski, & Grygiel (2019)	Explicit	Cronbach's *α* = .70-.83	-	-	-
Category: Comprehensive measures						
ToM storybooks (Blijd-Hoogewys et al., 2008)	Bartoli, Bulgarelli, & Molina (2019)	Explicit	-	-	-	Associated with age; two versions of the test (audio and visual) are correlated together
	Blijd-Hoogewys, van Geert, Serra, & Minderaa (2008)	Explicit	Structure analyzes were performed, Cronbach's α = .47-.80; Intercorrelations between 4 parrallel parts (books): *r* = .67-.79;	Cohen's κ = .81-.99	*r* = .86-.98	Scaling analysis was performed, norms are available; discriminates between ASD and TD; associated with sociability, verbal ability, IQ and other TOM tests
	Bulgarelli, Testa, & Molina (2015)	Explicit	Structure analyzes were performed	-	-	-

Appendix III

Psychological explanation task (Colonnesi, Rieffe, Koops & Perucchini, 2008)	Colonnesi et al. (2008)	Explicit	-	Cohen's κ = .93-.95	-	-
Comic strip task (Cornish et al. 2010)	Sivaratnam, Cornish, Gray, Howlin, & Rinehart (2012)	Explicit	Cronbach's α = -.04-.79	-	-	Discriminates between ASD
Perspective taking task (Edelstein, Keller & Wahlen; 1984)	LeMare & Rubin (1987)	Explicit	-	% agreement = 93%	-	Scaling analyses were performed
TOM task battery (Hutchins, Prelock, Chace, 2008)	Hutchins, Prelock, et al. (2008)	Explicit	Cronbach's α = .91-.94	% agreement = 97,2 % à 100 %	Cohen's κ = .08-.1.00	-
	Nader-Grosbois & Houssa (2016)	Explicit	Cronbach's α = .75; Structure analyses was performed	% agreement = 97,2-100 %	r = 0,868, p < 0,001	Associated with age, other TOM tests and social abilities
Theory of mind subtest from a developmental neuropsychological assessment (NEPSY-II; Korkman, Kirk & Kamp, 2007)	Korkman (2007)/Korkman (2012)	Explicit	Mean consistency coefficient (split-half and Cronbach's α): *r* = .84	% agreement = 99%	*r* = .84; % consistency of percentile rank classification = .91-.96	Discriminates between ASD and TD; measurement error and norms are available
Perspective-taking (Krcmar & Vieira, 2005)	Krcmar & Vieira (2005)	Explicit	Structure analyses were performed	Cohen's κ > .80	-	-
Pragma test (Loukusa et al., 2018)	Loukusa, Makinen, Kuusikko-Gauffin, Ebeling, & Leinonen (2018)	Explicit	-	Intraclass correlations= .94-.99	-	Discriminates between TD and ASD
Social meaning scale from the SELweb (McKown, Russo-Ponsaran, Johnson, Russo & Allen, 2016)	McKown, Russo-Ponsaran, Johnson, Russo, and Allen (2016)	Explicit	Cronbach's α = .78.80; Structure analyses were performed	-	*r* = .62-.69	Associated with social skills, social acceptance and academic competence; divergent validity with behavior problems
TOM test (Muris, Steerneman, Meesters, Merckelbach, Horselenberg, van den Hogen & van Dongen, 1999)	Broeren & Muris (2009)	Explicit	Cronbach's α = .91	-	-	-
	Broeren, Muris, Diamantopoulou, & Baker (2013)	Explicit	Cronbach's α = .91	-	-	-
	Colonnesi, Nikolic, de Vente, & Bogels (2017)	Explicit	Cronbach's α = .67	Cohen's κ = .92-.99	-	-
	Muris et al. (1999)	Explicit	Cronbach's α = .95-98	Spearman rank correlation = .99, p < .001; Cohen's κ > .87	*r* = .88; intraclass correlation = .80-.99	Discriminates between ASD and other clinical groups (anxiety, ADHD), associated with age, emotion recognition, social abilities and other TOM tests

Appendix III

Perspective-taking tasks (Oppenheimer & Thijssen, 1983)	Oppenheimer & Thijssen (1983)	Explicit	Cronbach's α = .91-.97	-	-	-
Theory of mind test (Pons & Harris, 2002)	Lecce et al. (2015)	Explicit	Cronbach's α = .64-69	-	-	-
Explanation of Action task (Tager-Flusberg and Sullivan, 1994)	Tager-Flusberg & Sullivan (2000)	Explicit	-	% agreement = 100%	-	-
ToM scale (Wellman and Liu, 2004)	Bialecka-Pikul et al. (2019)	Explicit	-	Cohen's κ = 0.79-0.88	-	-
	Brooks & Meltzoff (2015)	Explicit	-	Cohen's κ = 1.0	-	-
	Burnel, Perrone-Bertolotti, Reboul, Baciu, & Durrleman (2018)	Explicit	-	-	-	Scaling analyses were performed
	Burkart & Rueth (2013)	Explicit	-	-	-	Scaling analyses were performed
	Centifanti, Meins, & Fernyhough (2016)	Explicit	Cronbach's α = .63	-	-	-
	Cochet, Jover, Rizzo, & Vauclair (2017)	Explicit	-	Cohen's κ = .92	-	-
	Davis, Meins, & Fernyhough (2011)	Explicit	Cronbach's α = .69	-	-	-
	Duh et al. (2016)	Explicit	-	-	-	Scaling analyses were performed
	Hanson et al. (2014)	Explicit	-	Cohen's κ = .81-1.0	-	-
	Hasni, Adamson, Williamson, & Robins (2017)	Explicit	-	Cohen's κ = .92	-	Scaling analyses were performed and convergent validity was found between a visual and a auditory version of the scale
	Hiller et al. (2014)	Explicit	-	-	-	Scaling analyses were performed

Appendix III

Jester & Johnson (2016)	Explicit	-	-	-	Scaling analyses were performed
Larkin, Meins, Centifanti, Fernyhough, & Leekam (2016)	Explicit	Cronbach's α = -.63-.61	-	-	-
Martins et al. (2016)	Explicit	-	Cohen's κ ≥ .88	-	-
Nathanson, Sharp, Alade, Rasmussen, & Christy (2013)	Explicit	Cronbach's α = .56	-	-	-
Olineck & Poulin-Dubois (2007)	Explicit	-	% agreement = 100%	-	-
Pauker, Perlman, Prime, & Jenkins (2016)	Explicit	Cronbach's α = -.81	-	-	-
Peterson et al. (2012)	Explicit	-	% agreement = 95%	-	Scaling analyses were performed. Associated with age, discriminates between TD, deafness and ASD
Peterson, Wellman, & Liu (2005)	Explicit	-	-	-	Scaling analyses were performed
Prime et al. (2015)	Explicit	Cronbach's α = .67	-	-	-
Remmel & Peters (2009)	Explicit	-	-	-	Scaling analyses were performed
Seidenfeld, Johnson, Cavadel, & Izard (2014)	Explicit	Cronbach's α = .74	-	-	-
Shahaeian et al. (2014)	Explicit	-	-	-	Scaling analyses were performed
Smogorzewska et al. (2018)	Explicit	Structure analyzes were performed	-	-	Associated with other TOM tests and social functioning and language abilities
Smogorzewska et al. (2019)	Explicit	Cronbach's α = .77	-	-	-

Appendix III

Sodian & Kristen-Antonow (2015)	Explicit	-	% agreement = 100%	-	-
Wade, Browne, Madigan, Plamondon, & Jenkins (2014)	Explicit	Cronbach's α = .87	-	-	-
Wade, Browne, Plamondon, Daniel, & Jenkins (2016)	Explicit	Cronbach's α = -.85-.87	-	-	-
Wade, Hoffmann, & Jenkins (2015)	Explicit	Cronbach's α = .87	-	-	-
Wade, Hoffmann, Knafo-Noam, O'Connor, & Jenkins (2016)	Explicit	Cronbach's α = .87	-	-	-
Wade et al. (2018)	Explicit	Cronbach's α = .87	-	-	-
Wellman, Fang, Liu, Zhu, & Liu (2006)	Explicit	-	-	-	Scaling analysis were performed
Wellman & Liu (2004)	Explicit	-	-	-	Scaling analyses were performed
Wong & Leung (2010)	Explicit	-	-	-	Scaling analysis were performed
Zhang, Shao, & Zhang (2016)	Explicit	-	-	-	Scaling analyses were performed. Discriminates between ASD and TD

Appendix III

Atkinson, L., Slade, L., Powell, D., & Levy, J. P. (2017). Theory of mind in emerging reading comprehension: A longitudinal study of early indirect and direct effects. *Journal of Experimental Child Psychology*, No Pagination Specified-No Pagination Specified. doi:10.1016/j.jecp.2017.04.007

Banerjee, R., & Yuill, N. (1999). Children's understanding of self-presentational display rules: Associations with mental-state understanding. *British Journal of Developmental Psychology, 17*(Pt 1), 111-124. doi:http://dx.doi.org/10.1348/026151099165186

Barbosa-Leiker, C., Strand, P. S., Mamey, M. R., & Downs, A. (2014). Psychometric properties of the Emotion Understanding Assessment with Spanish- and English-speaking preschoolers attending Head Start. *Assessment, 21*(5), 628-636. doi:http://dx.doi.org/10.1177/1073191114524017

Bartoli, G., Bulgarelli, D., & Molina, P. (2019). Theory of Mind Development in Children with Visual Impairment: The Contribution of the Adapted Comprehensive Test ToM Storybooks. *Journal of Autism & Developmental Disorders, 49*(9), 3494-3503.

Bartsch, K., & Wellman, H. (1989). Young Children's Attribution of Action to Beliefs and Desires. *Child Development, 60*(4), 946-964. doi:10.2307/1131035

Bellagamba, F., Addessi, E., Focaroli, V., Pecora, G., Maggiorelli, V., Pace, B., & Paglieri, F. (2015). False belief understanding and "cool" inhibitory control in 3-and 4-years-old Italian children. *Frontiers in Psychology, 6*, 872. doi:https://dx.doi.org/10.3389/fpsyg.2015.00872

Bellagamba, F., Laghi, F., Lonigro, A., & Pace, C. S. (2012). Re-enactment of intended acts from a video presentation by 18- and 24-month-old children. *Cognitive Processing, 13*(4), 381-386. doi:http://dx.doi.org/10.1007/s10339-012-0518-0

Ben-Israel, S., Uzefovsky, F., Ebstein, R. P., & Knafo-Noam, A. (2015). Dopamine D4 receptor polymorphism and sex interact to predict children's affective knowledge. *Frontiers in Psychology, 6*, 846. doi:https://dx.doi.org/10.3389/fpsyg.2015.00846

Blankson, A. N., Weaver, J. M., Leerkes, E. M., O'Brien, M., Calkins, S. D., & Marcovitch, S. (2017). Cognitive and emotional processes as predictors of a successful transition into school. *Early Education and Development, 28*(1), 1-20. doi:10.1080/10409289.2016.1183434

Blijd-Hoogewys, E., van Geert, P., Serra, M., & Minderaa, R. (2008). Measuring theory of mind in children. Psychometric properties of the ToM storybooks. *Journal of Autism and Developmental Disorders, 38*(10), 1907-1930. doi:http://dx.doi.org/10.1007/s10803-008-0585-3

Broeren, S., & Muris, P. (2009). The relation between cognitive development and anxiety phenomena in children. *Journal of Child and Family Studies, 18*(6), 702-709. doi:http://dx.doi.org/10.1007/s10826-009-9276-8

Broeren, S., Muris, P., Diamantopoulou, S., & Baker, J. R. (2013). The course of childhood anxiety symptoms: developmental trajectories and child-related factors in normal children. *Journal of Abnormal Child Psychology, 41*(1), 81-95. doi:http://dx.doi.org/10.1007/s10802-012-9669-9

Brooks, R., & Meltzoff, A. N. (2015). Connecting the dots from infancy to childhood: a longitudinal study connecting gaze following, language, and explicit theory of mind. *Journal of Experimental Child Psychology, 130*, 67-78. doi:http://dx.doi.org/10.1016/j.jecp.2014.09.010

Bulgarelli, D., Testa, S., & Molina, P. (2015). Factorial structure of the 'ToM Storybooks': A test evaluating multiple components of Theory of Mind. *British Journal of Developmental Psychology, 33*(2), 187-202. doi:http://dx.doi.org/10.1111/bjdp.12062

Appendix III

Buresh, J. S., & Woodward, A. L. (2007). Infants track action goals within and across agents. *Cognition, 104*(2), 287-314. doi:http://dx.doi.org/10.1016/j.cognition.2006.07.001

Burkart, J. M., & Rueth, K. (2013). Preschool children fail primate prosocial game because of attentional task demands. *PLoS ONE [Electronic Resource], 8*(7), e68440. doi:http://dx.doi.org/10.1371/journal.pone.0068440

Burnel, M., Perrone-Bertolotti, M., Reboul, A., Baciu, M., & Durrleman, S. (2018). Reducing the language content in ToM tests: A developmental scale. *Developmental Psychology, 54*(2), 293-307.

Buttelmann, D., Carpenter, M., & Tomasello, M. (2009). Eighteen-month-old infants show false belief understanding in an active helping paradigm. *Cognition, 112*(2), 337-342. doi:http://dx.doi.org/10.1016/j.cognition.2009.05.006

Buttelmann, D., Over, H., Carpenter, M., & Tomasello, M. (2014). Eighteen-month-olds understand false beliefs in an unexpected-contents task. *Journal of Experimental Child Psychology, 119*, 120-126. doi:http://dx.doi.org/10.1016/j.jecp.2013.10.002

Callaghan, T., Rochat, P., Lillard, A., Claux, M. L., Odden, H., Itakura, S., . . . Singh, S. (2005). Synchrony in the onset of mental-state reasoning: evidence from five cultures. *Psychological Science, 16*(5), 378-384.

Callaghan, T. C., Rochat, P., & Corbit, J. (2012). Young children's knowledge of the representational function of pictorial symbols: Development across the preschool years in three cultures. *Journal of Cognition and Development, 13*(3), 320-353. doi:http://dx.doi.org/10.1080/15248372.2011.587853

Camaioni, L., Perucchini, P., Bellagamba, F., & Colonnesi, C. (2004). The Role of Declarative Pointing in Developing a Theory of Mind. *Infancy, 5*(3), 291-308. doi:http://dx.doi.org/10.1207/s15327078in0503_3

Carlo, G., Knight, G. P., Eisenberg, N., & Rotenberg, K. J. (1991). Cognitive processes and prosocial behaviors among children: The role of affective attributions and reconciliations. *Developmental Psychology, 27*(3), 456-461. doi:http://dx.doi.org/10.1037/0012-1649.27.3.456

Carpenter, M., Call, J., & Tomasello, M. (2002). A new false belief test for 36-month-olds. *British Journal of Developmental Psychology, 20*(3), 393-420. doi:http://dx.doi.org/10.1348/026151002320620316

Cassidy, J., Parke, R. D., Butkovsky, L., & Braungart, J. M. (1992). Family-Peer Connections: The Roles of Emotional Expressiveness within the Family and Children's Understanding of Emotions. *Child Development, 63*(3), 603-618. doi:10.1111/j.1467-8624.1992.tb01649.x

Centifanti, L. C. M., Meins, E., & Fernyhough, C. (2016). Callous-unemotional traits and impulsivity: Distinct longitudinal relations with mind-mindedness and understanding of others. *Journal of Child Psychology and Psychiatry, 57*(1), 84-92. doi:10.1111/jcpp.12445

Chandler, M. J., & Helm, D. (1984). Developmental Changes in the Contribution of Shared Experience to Social Role-Taking Competence. *International Journal of Behavioral Development, 7*(2), 145-156. doi:10.1177/016502548400700203

Chasiotis, A., Kiessling, F., Hofer, J., & Campos, D. (2006). Theory of mind and inhibitory control in three cultures: Conflict inhibition predicts false belief understanding in Germany, Costa Rica and Cameroon. *International Journal of Behavioral Development, 30*(3), 249-260. doi:http://dx.doi.org/10.1177/0165025406066759

Chasiotis, A., Kiessling, F., Winter, V., & Hofer, J. (2006). Sensory motor inhibition as a prerequisite for theory-of-mind: A comparison of clinical and normal preschoolers differing in sensory motor abilities. *International Journal of Behavioral Development, 30*(2), 178-190. doi:http://dx.doi.org/10.1177/0165025406063637

Appendix III

Cheung, H. (2006). False belief and language comprehension in Cantonese-speaking children. *Journal of Experimental Child Psychology, 95*(2), 79-98. doi:http://dx.doi.org/10.1016/j.jecp.2006.05.002

Choi, Y.-j., & Luo, Y. (2015). 13-month-olds' understanding of social interactions. *Psychological Science, 26*(3), 274-283. doi:http://dx.doi.org/10.1177/0956797614562452

Cochet, H., Jover, M., Rizzo, C., & Vauclair, J. (2017). Relationships between declarative pointing and theory of mind abilities in 3- to 4-year-olds. *European Journal of Developmental Psychology, 14*(3), 324-336. doi:10.1080/17405629.2016.1205975

Colonnesi, C., Nikolic, M., de Vente, W., & Bogels, S. M. (2017). Social Anxiety Symptoms in Young Children: Investigating the Interplay of Theory of Mind and Expressions of Shyness. *Journal of Abnormal Child Psychology, 45*(5), 997-1011. doi:10.1007/s10802-016-0206-0

Colonnesi, C., Rieffe, C., Koops, W., & Perucchini, P. (2008). Precursors of a theory of mind: A longitudinal study. *British Journal of Developmental Psychology, 26*(4), 561-577. doi:http://dx.doi.org/10.1348/026151008X285660

Crivello, C., Phillips, S., & Poulin-Dubois, D. (2018). Selective social learning in infancy: looking for mechanisms. *Developmental Science, 21*(3), e12592.

Cutting, A. L., & Dunn, J. (2002). The cost of understanding other people: social cognition predicts young children's sensitivity to criticism. *Journal of Child Psychology and Psychiatry and Allied Disciplines, 43*(7), 849-860.

Cutting, A. L., & Dunn, J. (2006). Conversations with siblings and with friends: Links between relationship quality and social understanding. *British Journal of Developmental Psychology, 24*(1), 73-87. doi:http://dx.doi.org/10.1348/026151005X70337

Davis, P. E., Meins, E., & Fernyhough, C. (2011). Self-knowledge in childhood: relations with children's imaginary companions and understanding of mind. *British Journal of Developmental Psychology, 29*(Pt 3), 680-686. doi:http://dx.doi.org/10.1111/j.2044-835X.2011.02038.x

De Rosnay, M., Fink, E., Begeer, S., Slaughter, V., & Peterson, C. (2014). Talking theory of mind talk: young school-aged children's everyday conversation and understanding of mind and emotion. *Journal of Child Language, 41*(5), 1179-1193. doi:http://dx.doi.org/10.1017/S0305000913000433

Denham, S. A. (1986). Social cognition, prosocial behavior, and emotion in preschoolers: Contextual validation. *Child Development, 57*(1), 194-201. doi:http://dx.doi.org/10.2307/1130651

Dörrenberg, S., Rakoczy, H., & Liszkowski, U. (2018). How (not) to measure infant theory of mind: Testing the replicability and validity of four non-verbal measures. *Cognitive Development*, No Pagination Specified-No Pagination Specified. doi:10.1016/j.cogdev.2018.01.001

Dorrenberg, S., Wenzel, L., Proft, M., Rakoczy, H., & Liszkowski, U. (2019). Reliability and generalizability of an acted-out false belief task in 3-year-olds. *Infant Behavior & Development, 54*, 13-21.

Downs, A., Strand, P., & Cerna, S. (2007). Emotion Understanding in English- and Spanish-speaking Preschoolers Enrolled in Head Start. *Social Development, 16*(3), 410-439. doi:10.1111/j.1467-9507.2007.00391.x

Duh, S., Paik, J. H., Miller, P. H., Gluck, S. C., Li, H., & Himelfarb, I. (2016). Theory of mind and executive function in Chinese preschool children. *Developmental Psychology, 52*(4), 582-591. doi:10.1037/a0040068

Dunn, J., Cutting, A. L., & Demetriou, H. (2000). Moral sensibility, understanding others, and children's friendship interactions in the preschool period. *British Journal of Developmental Psychology, 18*(2), 159-177. doi:http://dx.doi.org/10.1348/026151000165625

Dyck, M., Ferguson, K., & Shochet, I. (2001). Do autism spectrum disorders differ from each other and from non-spectrum disorders on emotion recognition tests? *European Child and Adolescent Psychiatry, 10*(2), 105-116. doi:http://dx.doi.org/10.1007/s007870170033

Ebersbach, M., Stiehler, S., & Asmus, P. (2011). On the relationship between children's perspective taking in complex scenes and their spatial drawing ability. *British Journal of Developmental Psychology, 29*(Pt 3), 455-474. doi:http://dx.doi.org/10.1348/026151010X504942

Fedra, E., & Schmidt, M. F. H. (2019). Older (but not younger) preschoolers reject incorrect knowledge claims. *British Journal of Developmental Psychology, 37*(1), 130-145. doi:10.1111/bjdp.12264

Feshbach, N. D., & Cohen, S. (1988). Training affect comprehension in young children: An experimental evaluation. *Journal of Applied Developmental Psychology, 9*(2), 201-210. doi:http://dx.doi.org/10.1016/0193-3973%2888%2990023-8

Fidler, D. J., Hepburn, S. L., Most, D. E., Philofsky, A., & Rogers, S. J. (2007). Emotional responsivity in young children with Williams syndrome. *American Journal on Mental Retardation, 112*(3), 194-206. doi:http://dx.doi.org/10.1352/0895-8017%282007%29112%5B194:ERIYCW%5D2.0.CO;2

Filippova, E., & Astington, J. W. (2008). Further development in social reasoning revealed in discourse irony understanding. *Child Development, 79*(1), 126-138. doi:http://dx.doi.org/10.1111/j.1467-8624.2007.01115.x

Flynn, E. (2006). A microgenetic investigation of stability and continuity in theory of mind development. *British Journal of Developmental Psychology, 24*(3), 631-654. doi:http://dx.doi.org/10.1348/026151005X57422

Frick, A., Mohring, W., & Newcombe, N. S. (2014). Picturing perspectives: development of perspective-taking abilities in 4- to 8-year-olds. *Frontiers in Psychology, 5*, 386. doi:http://dx.doi.org/10.3389/fpsyg.2014.00386

Garnham, W. A., & Ruffman, T. (2001). Doesn't see, doesn't know: Is anticipatory looking really related to understanding of belief? *Developmental Science, 4*(1), 94-100. doi:http://dx.doi.org/10.1111/1467-7687.00153

Gratch, G. (1964). RESPONSE ALTERNATION IN CHILDREN: A DEVELOPMENTAL STUDY OF ORIENTATIONS TO UNCERTAINTY. *Vita Hum Int Z Lebensalterforsch, 7*, 49-60.

Grazzani, I., Ornaghi, V., & Brockmeier, J. (2016). Conversation on mental states at nursery: Promoting social cognition in early childhood. *European Journal of Developmental Psychology, 13*(5), 563-581. doi:10.1080/17405629.2015.1127803

Grosso, S. S., Schuwerk, T., Kaltefleiter, L. J., & Sodian, B. (2019). 33-month-old children succeed in a false belief task with reduced processing demands: A replication of Setoh et al. (2016). *Infant Behavior & Development, 54*, 151-155.

Guajardo, N. R., Parker, J., & Turley-Ames, K. (2009). Associations among false belief understanding, counterfactual reasoning, and executive function. *British Journal of Developmental Psychology, 27*(Pt 3), 681-702.

Hahn, L. J., Fidler, D. J., Hepburn, S. L., & Rogers, S. J. (2013). Early intersubjective skills and the understanding of intentionality in young children with Down syndrome. *Research in*

Developmental Disabilities, 34(12), 4455-4465. doi:http://dx.doi.org/10.1016/j.ridd.2013.09.027
Hanson, L. K., & Atance, C. M. (2014). Brief report: Episodic foresight in autism spectrum disorder. *Journal of Autism and Developmental Disorders, 44*(3), 674-684. doi:http://dx.doi.org/10.1007/s10803-013-1896-6
Hanson, L. K., Atance, C. M., & Paluck, S. W. (2014). Is thinking about the future related to theory of mind and executive function? Not in preschoolers. *Journal of Experimental Child Psychology, 128*, 120-137. doi:http://dx.doi.org/10.1016/j.jecp.2014.07.006
Hasni, A. A., Adamson, L. B., Williamson, R. A., & Robins, D. L. (2017). Adding sound to theory of mind: Comparing children's development of mental-state understanding in the auditory and visual realms. *Journal of Experimental Child Psychology, 164*, 239-249.
Herold, K. H., & Akhtar, N. (2008). Imitative learning from a third-party interaction: relations with self-recognition and perspective taking. *Journal of Experimental Child Psychology, 101*(2), 114-123. doi:http://dx.doi.org/10.1016/j.jecp.2008.05.004
Hiller, R. M., Weber, N., & Young, R. L. (2014). The validity and scalability of the Theory of Mind Scale with toddlers and preschoolers. *Psychological Assessment, 26*(4), 1388-1393. doi:http://dx.doi.org/10.1037/a0038320
Howe, N., Recchia, H., Porta, S. D., & Funamoto, A. (2012). "The driver doesn't sit, he stands up like the Flintstones!": Sibling teaching during teacher-directed and self-guided tasks. *Journal of Cognition and Development, 13*(2), 208-231. doi:http://dx.doi.org/10.1080/15248372.2011.577703
Hughes, C., Adlam, A., Happe, F., Jackson, J., Taylor, A., & Caspi, A. (2000). Good test--retest reliability for standard and advanced false-belief tasks across a wide range of abilities. *Journal of Child Psychology and Psychiatry and Allied Disciplines, 41*(4), 483-490.
Hutchins, T. L., Bonazinga, L. A., Prelock, P. A., & Taylor, R. S. (2008). Beyond false beliefs: the development and psychometric evaluation of the perceptions of children's theory of mind measure-experimental version (PCToMM-E). *Journal of Autism and Developmental Disorders, 38*(1), 143-155.
Hutchins, T. L., Prelock, P. A., & Chace, W. (2008). Test-retest reliability of a theory of mind task battery for children with Autism Spectrum Disorders. *Focus on Autism and Other Developmental Disabilities, 23*(4), 195-206. doi:http://dx.doi.org/10.1177/1088357608322998
Hwa-Froelich, D. A., Matsuo, H., & Jacobs, K. (2017). False Belief Performance of Children Adopted Internationally. *American Journal of Speech-Language Pathology, 26*(1), 29-43. doi:https://dx.doi.org/10.1044/2016_AJSLP-15-0152
Iannotti, R. J. (1978). Effect of role-taking experiences on role taking, empathy, altruism, and aggression. *Developmental Psychology, 14*(2), 119-124. doi:10.1037/0012-1649.14.2.119
Jester, M., & Johnson, C. J. (2016). Differences in theory of mind and pretend play associations in children with and without specific language impairment. *Infant and Child Development, 25*(1), 24-42.
Jin, X., Li, P., He, J., & Shen, M. (2017). Cooperation, but not competition, improves 4-year-old children's reasoning about others' diverse desires. *Journal of Experimental Child Psychology, 157*, 81-94. doi:10.1016/j.jecp.2016.12.010
Kårstad, S. B., Wichstrøm, L., Reinfjell, T., Belsky, J., & Berg-Nielsen, T. S. (2015). What enhances the development of emotion understanding in young children? A longitudinal study of interpersonal predictors. *British Journal of Developmental Psychology, 33*(3), 340-354. doi:10.1111/bjdp.12095

Kennedy, K., Lagattuta, K. H., & Sayfan, L. (2015). Sibling composition, executive function, and children's thinking about mental diversity. *Journal of Experimental Child Psychology, 132*, 121-139. doi:http://dx.doi.org/10.1016/j.jecp.2014.11.007

Kim, Y.-S., & Phillips, B. (2014). Cognitive correlates of listening comprehension. *Reading Research Quarterly, 49*(3), 269-281.

Knafo, A., Steinberg, T., & Goldner, I. (2011). Children's low affective perspective-taking ability is associated with low self-initiated pro-sociality. *Emotion, 11*(1), 194-198. doi:http://dx.doi.org/10.1037/a0021240

Knafo, A., Zahn-Waxler, C., Davidov, M., Van Hulle, C., Robinson, J. L., & Rhee, S. H. (2009). Empathy in Early Childhood. *Annals of the New York Academy of Sciences, 1167*(1), 103-114. doi:10.1111/j.1749-6632.2009.04540.x

Kolodziejczyk, A. M., & Bosacki, S. L. (2015). Children's understandings of characters' beliefs in persuasive arguments: Links with gender and theory of mind. *Early Child Development and Care, 185*(4), 564-579. doi:http://dx.doi.org/10.1080/03004430.2014.940930

Korkman, M., Kirk, U, Kemp, S. (2007). *Clinical and interpretive manual NEPSY-II*. San Antonio, TX: Harcourt Assessment.

Korkman, M., Kirk, U., Kemp, S. (2012). *NEPSY-II. Manuel clinique et d'interprétation, Version française*. Montreuil, France: PEARSON FRANCE-ECPA.

Krcmar, M., & Vieira, E. T., Jr. (2005). Imitating Life, Imitating Television: The Effects of Family and Television Models on Children's Moral Reasoning. *Communication Research, 32*(3), 267-294. doi:http://dx.doi.org/10.1177/0093650205275381

Kristen, S., Sodian, B., Thoermer, C., & Perst, H. (2011). Infants' joint attention skills predict toddlers' emerging mental state language. *Developmental Psychology, 47*(5), 1207-1219. doi:http://dx.doi.org/10.1037/a0024808

Kulke, L., Johannsen, J., & Rakoczy, H. (2019). Why can some implicit Theory of Mind tasks be replicated and others cannot? A test of mentalizing versus submentalizing accounts. *PLoS ONE [Electronic Resource], 14*(3), e0213772.

Kulke, L., Reiß, M., Krist, H., & Rakoczy, H. (2017). How robust are anticipatory looking measures of theory of mind? Replication attempts across the life span. *Cognitive Development*, No Pagination Specified-No Pagination Specified. doi:10.1016/j.cogdev.2017.09.001

Lackner, C., Sabbagh, M. A., Hallinan, E., Liu, X., & Holden, J. J. (2012). Dopamine receptor D4 gene variation predicts preschoolers' developing theory of mind. *Developmental Science, 15*(2), 272-280. doi:http://dx.doi.org/10.1111/j.1467-7687.2011.01124.x

Lagattuta, K. H., Sayfan, L., & Blattman, A. J. (2010). Forgetting common ground: six- to seven-year-olds have an overinterpretive theory of mind. *Developmental Psychology, 46*(6), 1417-1432. doi:http://dx.doi.org/10.1037/a0021062

Lane, J. D., Wellman, H. M., Olson, S. L., LaBounty, J., & Kerr, D. C. (2010). Theory of mind and emotion understanding predict moral development in early childhood. *British Journal of Developmental Psychology, 28*(Pt 4), 871-889.

Lapan, C., & Boseovski, J. J. (2016). Theory of mind and children's trait attributions about average and typically stigmatized peers. *Infant and Child Development, 25*(2), 158-178. doi:10.1002/icd.1923

Larkin, F., Meins, E., Centifanti, L. C. M., Fernyhough, C., & Leekam, S. R. (2016). How does restricted and repetitive behavior relate to language and cognition in typical development? *Development and Psychopathology*, No Pagination Specified-No Pagination Specified. doi:10.1017/S0954579416000535

Appendix III

Lecce, S., Bianco, F., Devine, R. T., Hughes, C., & Banerjee, R. (2014). Promoting theory of mind during middle childhood: a training program. *Journal of Experimental Child Psychology, 126*, 52-67. doi:http://dx.doi.org/10.1016/j.jecp.2014.03.002

Lecce, S., Caputi, M., & Pagnin, A. (2014). Long-term effect of theory of mind on school achievement: The role of sensitivity to criticism. *European Journal of Developmental Psychology, 11*(3), 305-318. doi:http://dx.doi.org/10.1080/17405629.2013.821944

Lecce, S., Demicheli, P., Zocchi, S., & Palladino, P. (2015). The origins of children's metamemory: The role of theory of mind. *Journal of Experimental Child Psychology, 131*, 56-72. doi:http://dx.doi.org/10.1016/j.jecp.2014.11.005

Lecce, S., & Hughes, C. (2010). 'The Italian job?': comparing theory of mind performance in British and Italian children. *British Journal of Developmental Psychology, 28*(Pt 4), 747-766.

LeMare, L. J., & Rubin, K. H. (1987). Perspective taking and peer interaction: Structural and developmental analyses. *Child Development, 58*(2), 306-315. doi:http://dx.doi.org/10.2307/1130508

Loukusa, S., Makinen, L., Kuusikko-Gauffin, S., Ebeling, H., & Leinonen, E. (2018). Assessing social-pragmatic inferencing skills in children with autism spectrum disorder. *Journal of Communication Disorders, 73*, 91-105.

Martins, E. C., Osório, A., Veríssimo, M., & Martins, C. (2016). Emotion understanding in preschool children: The role of executive functions. *International Journal of Behavioral Development, 40*(1), 1-10. doi:10.1177/0165025414556096

Mayes, L. C., Klin, A., Tercyak, K. P., Jr., Cicchetti, D. V., & Cohen, D. J. (1996). Test-retest reliability for false-belief tasks. *Journal of Child Psychology and Psychiatry and Allied Disciplines, 37*(3), 313-319.

McKown, C., Russo-Ponsaran, N. M., Johnson, J. K., Russo, J., & Allen, A. (2016). Web-based assessment of children’s social-emotional comprehension. *Journal of Psychoeducational Assessment, 34*(4), 322-338. doi:10.1177/0734282915604564

Meltzoff, A. N. (1995). Understanding the Intentions of Others: Re-Enactment of Intended Acts by 18-Month-Old Children. *Developmental Psychology, 31*(5), 838-850. doi:10.1037/0012-1649.31.5.838

Meltzoff, A. N., & Brooks, R. (2008). Self-experience as a mechanism for learning about others: A training study in social cognition. *Developmental Psychology, 44*(5), 1257-1265. doi:http://dx.doi.org/10.1037/a0012888

Moll, H., Kane, S., & McGowan, L. (2015). Three-year-olds express suspense when an agent approaches a scene with a false belief. *Developmental Science*, No Pagination Specified. doi:http://dx.doi.org/10.1111/desc.12310

Moll, H., Koring, C., Carpenter, M., & Tomasello, M. (2006). Infants Determine Others' Focus of Attention by Pragmatics and Exclusion. *Journal of Cognition and Development, 7*(3), 411-430. doi:http://dx.doi.org/10.1207/s15327647jcd0703_9

Moll, H., & Tomasello, M. (2006). Level 1 perspective-taking at 24 months of age. *British Journal of Developmental Psychology, 24*(3), 603-613. doi:10.1348/026151005X55370

Moll, H., & Tomasello, M. (2007). How 14- and 18-month-olds know what others have experienced. *Developmental Psychology, 43*(2), 309-317. doi:10.1037/0012-1649.43.2.309

Mull, M. S., & Evans, E. M. (2010). Did she mean to do it? Acquiring a folk theory of intentionality. *Journal of Experimental Child Psychology, 107*(3), 207-228. doi:http://dx.doi.org/10.1016/j.jecp.2010.04.001

Muris, P., Steerneman, P., Meesters, C., Merckelbach, H., Horselenberg, R., van den Hogen, T., & van Dongen, L. (1999). The TOM test: a new instrument for assessing theory of mind in normal children and children with pervasive developmental disorders. *Journal of Autism and Developmental Disorders, 29*(1), 67-80.

Nader-Grosbois, N., & Houssa, M. (2016). La Batterie de tâches de Théorie de l'esprit: Validation de la version francophone [Validation of the French version of the ToM Task Battery]. *Enfance: Psychologie, Pedagogie, Neuropsychiatrie, Sociologie, 66*(2), 141-163.

Nathanson, A. I., Sharp, M. L., Alade, F., Rasmussen, E. E., & Christy, K. (2013). The relation between television exposure and theory of mind among preschoolers. *Journal of Communication, 63*(6), 1088-1108. doi:http://dx.doi.org/10.1111/jcom.12062

O'Kearney, R., Salmon, K., Liwag, M., Fortune, C. A., & Dawel, A. (2017). Emotional Abilities in Children with Oppositional Defiant Disorder (ODD): Impairments in Perspective-Taking and Understanding Mixed Emotions are Associated with High Callous-Unemotional Traits. *Child Psychiatry and Human Development, 48*(2), 346-357. doi:https://dx.doi.org/10.1007/s10578-016-0645-4

Olineck, K. M., & Poulin-Dubois, D. (2007). Imitation of intentional actions and internal state language in infancy predict preschool theory of mind skills. *European Journal of Developmental Psychology, 4*(1), 14-30. doi:http://dx.doi.org/10.1080/17405620601046931

Olson, S. L., Choe, D. E., & Sameroff, A. J. (2017). Trajectories of child externalizing problems between ages 3 and 10 years: Contributions of children's early effortful control, theory of mind, and parenting experiences. *Development and Psychopathology*, No Pagination Specified-No Pagination Specified. doi:10.1017/S095457941700030X

Oppenheimer, L., & Thijssen, F. (1983). Children's thinking about friendships and its relation to popularity. *The Journal of Psychology: Interdisciplinary and Applied, 114*(1), 69-78. doi:10.1080/00223980.1983.9915398

Ornaghi, V., Pepe, A., & Grazzani, I. (2016). False-Belief Understanding and Language Ability Mediate the Relationship between Emotion Comprehension and Prosocial Orientation in Preschoolers. *Frontiers in Psychology, 7*, 1534. doi:https://dx.doi.org/10.3389/fpsyg.2016.01534

Pauker, S., Perlman, M., Prime, H., & Jenkins, J. M. (2016). Differential parenting and children's social understanding. *Social Development*, No Pagination Specified-No Pagination Specified. doi:10.1111/sode.12214

Pearson, A., Marsh, L., Ropar, D., & Hamilton, A. (2016). Cognitive mechanisms underlying visual perspective taking in typical and ASC children. *Autism Research, 9*(1), 121-130. doi:10.1002/aur.1501

Peterson, C. C., Wellman, H. M., & Liu, D. (2005). Steps in theory-of-mind development for children with deafness or autism. *Child Development, 76*(2), 502-517.

Peterson, C. C., Wellman, H. M., & Slaughter, V. (2012). The mind behind the message: advancing theory-of-mind scales for typically developing children, and those with deafness, autism, or Asperger syndrome. *Child Development, 83*(2), 469-485. doi:http://dx.doi.org/10.1111/j.1467-8624.2011.01728.x

Phillips, A. T., & Wellman, H. M. (2005). Infants' understanding of object-directed action. *Cognition, 98*(2), 137-155. doi:10.1016/j.cognition.2004.11.005

Phillips, A. T., Wellman, H. M., & Spelke, E. S. (2002). Infants' ability to connect gaze and emotional expression to intentional action. *Cognition, 85*(1), 53-78. doi:http://dx.doi.org/10.1016/S0010-0277(02)00073-2

Pons, F., Harris, P. L., & de Rosnay, M. (2004). Emotion comprehension between 3 and 11 years: Developmental periods and hierarchical organization. *European Journal of Developmental Psychology, 1*(2), 127-152. doi:10.1080/17405620344000022

Poulin-Dubois, D., & Yott, J. (2014). Executive functions and theory of mind understanding in young children: A reciprocal relation? *Psychologie Francaise, 59*(1), 59-69. doi:http://dx.doi.org/10.1016/j.psfr.2013.11.002

Poulin-Dubois, D., & Yott, J. (2018). Probing the depth of infants' theory of mind: disunity in performance across paradigms. *Developmental Science, 21*(4), e12600.

Prime, H., Browne, D., Akbari, E., Wade, M., Madigan, S., & Jenkins, J. M. (2015). The development of a measure of maternal cognitive sensitivity appropriate for use in primary care health settings. *Journal of Child Psychology and Psychiatry, 56*(4), 488-495. doi:http://dx.doi.org/10.1111/jcpp.12322

Putko, A., & Zlotogorska, A. (2014). Predictions of actions and their justifications in false-belief tasks: The role of executive function. *Polish Psychological Bulletin, 45*(4), 500-510. doi:http://dx.doi.org/10.2478/ppb-2014-0060

Remmel, E., & Peters, K. (2009). Theory of mind and language in children with cochlear implants. *Journal of Deaf Studies & Deaf Education, 14*(2), 218-236. doi:http://dx.doi.org/10.1093/deafed/enn036

Ricard, M., Girouard, P. C., & Decarie, T. G. (1999). Personal pronouns and perspective taking in toddlers. *Journal of Child Language, 26*(3), 681-697.

Rieffe, C., Ketelaar, L., & Wiefferink, C. H. (2010). Assessing empathy in young children: Construction and validation of an Empathy Questionnaire (EmQue). *Personality and Individual Differences, 49*(5), 362-367. doi:http://dx.doi.org/10.1016/j.paid.2010.03.046

Riggio, M. M., & Cassidy, K. W. (2009). Preschoolers' processing of false beliefs within the context of picture book reading. *Early Education and Development, 20*(6), 992-1015. doi:http://dx.doi.org/10.1080/10409280903375685

Roby, E., & Scott, R. M. (2018). The relationship between parental mental-state language and 2.5-year-olds' performance on a nontraditional false-belief task. *Cognition, 180*, 10-23.

Russo-Ponsaran, N. M., McKown, C., Johnson, J. K., Allen, A. W., Evans-Smith, B., & Fogg, L. (2015). Social-emotional correlates of early stage social information processing skills in children with and without autism spectrum disorder. *Autism Research*, No Pagination Specified. doi:http://dx.doi.org/10.1002/aur.1463

Schult, C. A. (2002). Children's Understanding of the Distinction between Intentions and Desires. *Child Development, 73*(6), 1727-1747. doi:10.1111/1467-8624.t01-1-00502

Scott, R. M., He, Z., Baillargeon, R., & Cummins, D. (2012). False-belief understanding in 2.5-year-olds: evidence from two novel verbal spontaneous-response tasks. *Developmental Science, 15*(2), 181-193. doi:http://dx.doi.org/10.1111/j.1467-7687.2011.01103.x

Seidenfeld, A. M., Johnson, S. R., Cavadel, E. W., & Izard, C. E. (2014). Theory of mind predicts emotion knowledge development in Head Start children. *Early Education and Development, 25*(7), 933-948. doi:http://dx.doi.org/10.1080/10409289.2014.883587

Shahaeian, A., Nielsen, M., Peterson, C. C., & Slaughter, V. (2014). Cultural and family influences on children's theory of mind development: A comparison of Australian and Iranian school-age children. *Journal of Cross-Cultural Psychology, 45*(4), 555-568. doi:http://dx.doi.org/10.1177/0022022113513921

Shields, A., Dickstein, S., Seifer, R., Giusti, L., Magee, K. D., & Spritz, B. (2001). Emotional competence and early school adjustment: A study of preschoolers at risk. *Early Education and Development, 12*(1), 73-96. doi:http://dx.doi.org/10.1207/s15566935eed1201_5

Shiverick, S. M., & Moore, C. F. (2013). Fulfilment of intention and desire in children's judgements of emotion for sociomoral events. *British Journal of Developmental Psychology, 31*(4), 395-407. doi:http://dx.doi.org/10.1111/bjdp.12015

Simcock, G., Kildea, S., Elgbeili, G., Laplante, D. P., Cobham, V., & King, S. (2017). Prenatal maternal stress shapes children's theory of mind: the QF2011 Queensland Flood Study. *Journal of Developmental Origins of Health and Disease, 8*(4), 483-492.

Sivaratnam, C. S., Cornish, K., Gray, K. M., Howlin, P., & Rinehart, N. J. (2012). Brief report: assessment of the social-emotional profile in children with autism spectrum disorders using a novel comic strip task. *Journal of Autism and Developmental Disorders, 42*(11), 2505-2512. doi:http://dx.doi.org/10.1007/s10803-012-1498-8

Smogorzewska, J., Szumski, G., & Grygiel, P. (2018). Same or different? Theory of mind among children with and without disabilities. *PLoS ONE [Electronic Resource], 13*(10), e0202553.

Smogorzewska, J., Szumski, G., & Grygiel, P. (2019). The Children's Social Understanding Scale: An advanced analysis of a parent-report measure for assessing theory of mind in Polish children with and without disabilities. *Developmental Psychology, 55*(4), 835-845. doi:10.1037/dev0000673

Sodian, B., & Kristen-Antonow, S. (2015). Declarative joint attention as a foundation of theory of mind. *Developmental Psychology, 51*(9), 1190-1200. doi:https://dx.doi.org/10.1037/dev0000039

Sodian, B., Licata, M., Kristen-Antonow, S., Paulus, M., Killen, M., & Woodward, A. (2016). Understanding of Goals, Beliefs, and Desires Predicts Morally Relevant Theory of Mind: A Longitudinal Investigation. *Child Development, 87*(4), 1221-1232. doi:10.1111/cdev.12533

Sodian, B., Licata, M., Kristen-Antonow, S., Paulus, M., Killen, M., & Woodward, A. (2016). Understanding of goals, beliefs, and desires predicts morally relevant theory of mind: A longitudinal investigation. *Child Development, 87*(4), 1221-1232. doi:10.1111/cdev.12533

Strand, P. S., Downs, A., & Barbosa-Leiker, C. (2016). Does facial expression recognition provide a toehold for the development of emotion understanding? *Developmental Psychology, 52*(8), 1182-1191. doi:10.1037/dev0000144

Symons, D. K., Fossum, K.-L. M., & Collins, T. (2006). A Longitudinal Study of Belief and Desire State Discourse During Mother-Child Play and Later False Belief Understanding. *Social Development, 15*(4), 676-691. doi:http://dx.doi.org/10.1111/j.1467-9507.2006.00364.x

Symons, D. K., Peterson, C. C., Slaughter, V., Roche, J., & Doyle, E. (2005). Theory of mind and mental state discourse during book reading and story-telling tasks. *British Journal of Developmental Psychology, 23*(1), 81-102. doi:http://dx.doi.org/10.1348/026151004X21080

Tager-Flusberg, H., & Sullivan, K. (2000). A componential view of theory of mind: evidence from Williams syndrome. *Cognition, 76*(1), 59-90.

Tarullo, A. R., Youssef, A., Frenn, K. A., Wiik, K., Garvin, M. C., & Gunnar, M. R. (2016). Emotion understanding, parent mental state language, and behavior problems in internationally adopted children. *Development and Psychopathology, 28*(2), 371-383. doi:10.1017/S095457941500111X

van Dijk, A., Poorthuis, A. M. G., & Malti, T. (2017). Psychological processes in young bullies versus bully-victims. *Aggressive Behavior*, No Pagination Specified-No Pagination Specified. doi:10.1002/ab.21701

Viranyi, Z., Topal, J., Miklosi, A., & Csanyi, V. (2006). A nonverbal test of knowledge attribution: A comparative study on dogs and children. *Animal Cognition, 9*(1), 13-26. doi:http://dx.doi.org/10.1007/s10071-005-0257-z

Wade, M., Browne, D., Madigan, S., Plamondon, A., & Jenkins, J. (2014). Normal birth weight variation and children's neuropsychological functioning: Links between language, executive functioning, and theory of mind. *Journal of the International Neuropsychological Society, 20*(9), 909-919. doi:http://dx.doi.org/10.1017/S1355617714000745

Wade, M., Browne, D. T., Plamondon, A., Daniel, E., & Jenkins, J. M. (2016). Cumulative risk disparities in children's neurocognitive functioning: a developmental cascade model. *Developmental Science, 19*(2), 179-194. doi:https://dx.doi.org/10.1111/desc.12302

Wade, M., Hoffmann, T. J., & Jenkins, J. M. (2015). Gene-environment interaction between the oxytocin receptor (OXTR) gene and parenting behaviour on children's theory of mind. *Social Cognitive and Affective Neuroscience, 10*(12), 1749-1757. doi:https://dx.doi.org/10.1093/scan/nsv064

Wade, M., Hoffmann, T. J., Knafo-Noam, A., O'Connor, T. G., & Jenkins, J. M. (2016). Oxytocin and vasopressin hormone genes in children's externalizing problems: A cognitive endophenotype approach. *Hormones and Behavior, 82*, 78-86. doi:https://dx.doi.org/10.1016/j.yhbeh.2016.05.002

Wade, M., Madigan, S., Plamondon, A., Rodrigues, M., Browne, D., & Jenkins, J. M. (2018). Cumulative psychosocial risk, parental socialization, and child cognitive functioning: A longitudinal cascade model. *Developmental Psychology, 54*(6), 1038-1050.

Wellman, H. M., Fang, F., Liu, D., Zhu, L., & Liu, G. (2006). Scaling of theory-of-mind understandings in Chinese children. *Psychological Science, 17*(12), 1075-1081.

Wellman, H. M., & Liu, D. (2004). Scaling of theory-of-mind tasks. *Child Development, 75*(2), 523-541.

Wong, P. M., & Leung, S. O. (2010). The development of theory of mind ability in autistic children. *Psychologia: An International Journal of Psychological Sciences, 53*(3), 151-162. doi:http://dx.doi.org/10.2117/psysoc.2010.151

Zhang, T., Shao, Z., & Zhang, Y. (2016). Developmental steps in theory of mind of typical Chinese children and Chinese children with autism spectrum disorder. *Research in Autism Spectrum Disorders, 23*, 210-220. doi:10.1016/j.rasd.2015.10.005
