# Supplementary material for: Systematic Review and Inventory of Theory of Mind Measures for Young Children
Source: Front Psychol. 2020 Jan 15;10:2905. doi: 10.3389/fpsyg.2019.02905 (PMC6974541; doi:10.3389/fpsyg.2019.02905)
Supplement: Supplementary file 3 [file Table_3.pdf]

Appendix III

Table j.

Psychometric properties of questionnaires

Measures (source author, year)	Studies	Internal structure and consistency	Inter-rater reliability	Test-retest reliability	Other psychometric information
Supplementary social and maladaptive items/*Échelle d'adaptation sociale pour enfants* (Frith, Happé & Sidons, 1994)	Comte-Gervais, Giron, Soares-Boucaud, and Poussin (2008)	Cronbach's α = .77	-	-	Discriminates between ASD and TD and between language impairment and TD; associated with age, verbal abilities; norms are presented
	Hughes, Soares-Boucaud, Hochmann, and Frith (1997)	-	Spearman's rho = 20-75	-	Discriminates between ASD and TD; associated with age and ASD symptoms
Theory of mind inventory & Perceptions of children's theory of mind measure-experimental version (Hutchins, Prelock & Bonazinga, 2012; Hutchins, Bonazinga, et al., 2008)	Greenslade and Coggins (2016)	Cronbach's α = .96	-	-	Discriminates between ASD and TD; associated with age, social abilities and communication abilities.
	Houssa, Mazzone, and Nader-Grosbois (2014)	Cronbach's α = .94; Structure analyzes were performed	-	*r* = .89	Associated with age, TOM tests and social competence
	Hutchins, Bonazinga, Prelock, and Taylor (2008)	-	-	*r* = .89-.98	Discriminates between ASD and TD; associated with age, verbal abilities, TOM tests, parent's prediction of child performance on TOM tests.
	Hutchins, Prelock, and Bonazinga (2012)	Cronbach's α = .98; Structure analyses were performed	-	*r* = .89	Associated with TOM tests and verbal abilities
	Pujals et al. (2016)	Cronbach's α = .96	-	-	Discriminates between ASD and TD
Everyday mindreading skills and difficulties scale (Peterson, Garnett, Kelly & Attwood, 2009)	Peterson, Garnett, Kelly, and Attwood (2009)	Cronbach's α = .82	-	-	Discriminates between ASD and TD, associated with TOM tests
Children's social understanding scale (Tahiroglu, Moses, Carlson, Mahy, Olofson & Sabbagh, 2014)	Smogorzewska, Szumski, and Grygiel (2019)	Cronbach's α = .93-.96; Structure analyzes were performed	-	*r= .63-.72*	Discriminates between TD and children with disabilities (intellectual disabilities and hearing impairments); associated with age, social skills and TOM tests
	Tahiroglu et al. (2014)	Cronbach's α = .81-.94, Structure analyses were performed	-	*r* = .88	Associated with TOM tests, age, working memory; divergent validity: prospective memory and planning
	Tahiroglu and Taylor (2019)	Cronbach's α = .86	-	-	-

Appendix III

Comte-Gervais, I., Giron, A., Soares-Boucaud, I., & Poussin, G. (2008). Assessment of social intelligence in children with specific language impairment: Presentation of an assessing scale. *L'Evolution Psychiatrique, 73*(2), 353-366. doi:http://dx.doi.org/10.1016/j.evopsy.2008.02.004

Greenslade, K. J., & Coggins, T. E. (2016). Brief Report: An Independent Replication and Extension of Psychometric Evidence Supporting the Theory of Mind Inventory. *Journal of Autism and Developmental Disorders, 46*(8), 2785-2790. doi:https://dx.doi.org/10.1007/s10803-016-2784-7

Houssa, M., Mazzone, S., & Nader-Grosbois, N. (2014). Validation of a French version of the Theory of Mind Inventory (ToMI-vf). *European Review of Applied Psychology / Revue Europeenne de Psychologie Appliquee, 64*(4), 169-179. doi:http://dx.doi.org/10.1016/j.erap.2014.02.002

Hughes, C., Soares-Boucaud, I., Hochmann, J., & Frith, U. (1997). Social behaviour in pervasive developmental disorders: effects of informant, group and "theory-of-mind". *European Child and Adolescent Psychiatry, 6*(4), 191-198.

Hutchins, T. L., Bonazinga, L. A., Prelock, P. A., & Taylor, R. S. (2008). Beyond false beliefs: the development and psychometric evaluation of the perceptions of children's theory of mind measure-experimental version (PCToMM-E). *Journal of Autism and Developmental Disorders, 38*(1), 143-155.

Hutchins, T. L., Prelock, P. A., & Bonazinga, L. (2012). Psychometric evaluation of the Theory of Mind Inventory (ToMI): a study of typically developing children and children with autism spectrum disorder. *Journal of Autism and Developmental Disorders, 42*(3), 327-341. doi:http://dx.doi.org/10.1007/s10803-011-1244-7

Peterson, C. C., Garnett, M., Kelly, A., & Attwood, T. (2009). Everyday social and conversation applications of theory-of-mind understanding by children with autism-spectrum disorders or typical development. *European Child and Adolescent Psychiatry, 18*(2), 105-115. doi:http://dx.doi.org/10.1007/s00787-008-0711-y

Pujals, E., Batlle, S., Camprodon, E., Pujals, S., Estrada, X., Acena, M., . . . Perez-Sola, V. (2016). Brief Report: Translation and Adaptation of the Theory of Mind Inventory to Spanish. *Journal of Autism and Developmental Disorders, 46*(2), 685-690. doi:https://dx.doi.org/10.1007/s10803-015-2576-5

Smogorzewska, J., Szumski, G., & Grygiel, P. (2019). The Children’s Social Understanding Scale: An advanced analysis of a parent-report measure for assessing theory of mind in Polish children with and without disabilities. *Developmental Psychology, 55*(4), 835-845. doi:10.1037/dev0000673

Tahiroglu, D., Moses, L. J., Carlson, S. M., Mahy, C. E., Olofson, E. L., & Sabbagh, M. A. (2014). The Children's Social Understanding Scale: construction and validation of a parent-report measure for assessing individual differences in children's theories of mind. *Developmental Psychology, 50*(11), 2485-2497. doi:http://dx.doi.org/10.1037/a0037914

Tahiroglu, D., & Taylor, M. (2019). Anthropomorphism, social understanding, and imaginary companions. *British Journal of Developmental Psychology, 37*(2), 284-299.

Appendix III
