# Supplementary material for: Systematic Review and Inventory of Theory of Mind Measures for Young Children
Source: Front Psychol. 2020 Jan 15;10:2905. doi: 10.3389/fpsyg.2019.02905 (PMC6974541; doi:10.3389/fpsyg.2019.02905)
Supplement: Supplementary file 4 [file Data_Sheet_1.pdf]

Appendix I

List of potentially relevant articles written in a language other than French or English

Abel, C. G., Stein, G., Galarregui, M., Garretto, N., Mangone, C., Genovese, O., . . . Sica, R. E. (2007). [Social cognition and theory of mind assessment in non-demented patients with isolated cerebellar degeneration]. *Arquivos de Neuro-Psiquiatria, 65*(2A), 304-312.

Adrian, J.-E., Clemente, R.-A., & Villanueva, L. (2006). Emotional attribution dependent on false beliefs: Relations with peers' social interaction and children's language. *Infancia y Aprendizaje / Journal for the Study of Education and Development, 29*(2), 191-201. doi:http://dx.doi.org/10.1174/021037006776789890

Aliakbari, M., Juibari, A. K., Amirabadi, F., Shaghaghi, F., Zare, N., & Khaleghi, F. (2013). The role of Theory of Mind and executive function in predicting empathy. *Advances in Cognitive Science, 15*(2[58]; 58), 1-10.

Amorim, L. C. D., & Assumpcao, F. B., Jr. (2012). The concept of death and Asperger Syndrome. *Estudos de Psicologia, 29*(3), 363-370. doi:http://dx.doi.org/10.1590/S0103-166X2012000300006

Andres Roqueta, C., & Clemente Estevan, R. A. (2010). [Pragmatic difficulties in children with specific language impairment. The role of mentalistic tasks]. *Psicothema, 22*(4), 677-683.

Arranz Freijo, E., Olabarrieta Artetxe, F., Yenes Conde, F., & Martin Ayala, J. L. (2001). Perceptions of sibling relationships in 8-yr-olds and 11-yr-olds. *Revista de Psicologia General y Aplicada, 54*(3), 425-441.

Baguena, M. J., & Diaz, A. (1989). Evaluation of interpersonal social abilities in delinquent and nondelinquent adolescents using the BHSSPI-III. *Analisis y Modificacion de Conducta, 15*(44), 273-295.

Baumgartner, E., Bombi, A. S., & Pistorio, B. (2002). Psychological references in children's description of interpersonal relationships. *Eta Evolutiva, 73*, 48-54.

Baumgartner, E., & Dessi, V. (2002). Understanding and explaining others' actions: The development of the notion of intentionality. *Rassegna di Psicologia, 19*(1), 143-156.

Bellagamba, F., & Colonnesi, C. (2005). Evolution Of The Understanding Of The Other People's Intentions: The Origins of The Theory of The Mind in Infancy. *Giornale Italiano di Psicologia, 32*(4), 781-797.

Bermudez-Jaimes, M. E., & Sastre-Gomez, L. V. (2010). False belief and semantic language development in children aged 2 to 4 years. *Universitas Psychologica, 9*(3), 849-861.

Biao, S., Lili, M., & Ciping, D. (2004). An Experimental Study Between the Using of Mental Terms and the Development of Theory of Mind. *Psychological Science (China), 27*(3), 584-589.

Biao, S., Zhen, R., & Ciping, D. (2006). Central Coherence in Autistic Children and Its Links with Theory of Mind. *Psychological Science (China), 29*(1), 52-56.

Biscaldi, M., Paschke-Müller, M., Rauh, R., & Schaller, U. M. (2016). Evaluation des Freiburger TOMTASS: Ein soziales kompetenztraining mit schwerpunkt auf theory of mind für kinder und Jugendliche mit hochfunktionalen autismus-

spektrum-störungen. [Evaluation of the Freiburg TOMTASS: A theory-of-mind based social skills group training for children and adolescents with a high-functioning autism spectrum disorder.]. *Zeitschrift für Psychiatrie, Psychologie und Psychotherapie, 64*(4), 269-275.
Blijd-Hoogewys, E. M., Huyghen, A.-M. N., van Geert, P. L., Serra, M., Loth, F. L., & Minderaa, R. B. (2003). The Theory of Mind Story Books: Construction and setting standard norms for an instrument measuring theory of mind in young children. *Nederlands Tijdschrift voor de Psychologie en Haar Grensgebieden, 58*(2), 19-33.
Bohmann, J., Fritsch, S., Luck, M., Stumpe, A., Taubner, S., & Vesterling, C. (2014). [Mentalization and attachment transmission]. *Praxis der Kinderpsychologie und Kinderpsychiatrie, 63*(9), 699-722. doi:http://dx.doi.org/10.13109/prkk.2014.63.9.699
Bordbar, M., & Sheikholeslami, R. (2015a). Maternal mental states input and children social behavior: The mediator role of theory of mind. *Journal of Iranian Psychologists, 11*(42), 123-134.
Bordbar, M., & Sheikholeslami, R. (2015b). Maternal mental states input and children social behavior: The mediator role of theory of mind. [Maternal mental states input and children social behavior: The mediator role of theory of mind.]. *Journal of Iranian Psychologists, 11*(42), 123-134.
Caixeta, L., & Nitrini, R. (2002). Theory of mind: A review with focus on its incorporation into medical psychology. *Psicologia: Reflexao e Critica, 15*(1), 105-112. doi:http://dx.doi.org/10.1590/S0102-79722002000100012
Calderoni, S., Fantozzi, P., Maestro, S., Narzisi, A., & Muratori, F. (2012). Impaired cognitive empathy in adolescents with restrictive type anorexia nervosa. *Infanzia e Adolescenza, 11*(2), 81-90.
Camisasca, E. (2007). Maternal reflectiveness and mind-mindedness in the development of security of attachment and theory of mind. *Eta Evolutiva, 86*, 5-15.
Carlier, I., Hamel, B., & Koops, W. (1989). Perspective-taking as a condition for verbal politeness of kindergarten children. *Nederlands Tijdschrift voor de Psychologie en Haar Grensgebieden, 44*(8), 375-381.
Carvalho, L. R., Mecca, F. F., & Lichtig, I. (2008). [Assessment of self-representation abilities in seven-, eight-year-old children]. *Profono, 20*(2), 81-86.
Celani, G., Battistelli, P., & Battacchi, M. W. (1998). Deception and the comprehension of false beliefs: Knowledge of the deceitful consequences of their own actions among children less than 4 years old. *Giornale Italiano di Psicologia, 25*(3), 583-605.
Chasiotis, A., & Kiesling, F. (2004). Does the specifity of theory of mind and inhibitory control persist over the life-span? The relation of mentalistic and self-regulatory competence in adult age. *Zeitschrift für Entwicklungspsychologie und Pädagogische Psychologie, 36*(2), 105-114. doi:http://dx.doi.org/10.1026/0049-8637.36.2.105
Chiesi, F. (1999). False belief understanding in 3-yr-olds: A comparison among one's own false beliefs, others' false beliefs, and behavioral predictions. *Rassegna di Psicologia, 16*(1), 89-105.

Cicogni, A., Castagnoli, A., Domenichetti, S., Perone, R., & Sestini, S. (2008). Asperger syndrome, a diagnostic therapeutic paradox. *Giornale Italiano di Psicopatologia / Italian Journal of Psychopathology, 14*(4), 356-366.

Cusimano, G., Fama, F., & Miano, P. (2011). Mentalization processes in an home visiting intervention for a child with autistic disorder. *Infanzia e Adolescenza, 10*(1), 53-63.

da Silva Alves, A. C., das Gracas Bom pastor Borges Dias, M., & Sobral, A. B. C. (2007). The relationship between make-believe play and the development of abilities to acquisition of theory of mind. *Psicologia em Estudo, 12*(2), 325-334. doi:http://dx.doi.org/10.1590/S1413-73722007000200013

de Abreu, C. S., Cardoso-Martins, C., & Barbosa, P. G. (2014). The relationship between joint attention and theory of mind: A longitudinal study. *Psicologia: Reflexao e Critica, 27*(2), 409-414. doi:http://dx.doi.org/10.1590/1678-7153.201427222

de Jou, G. I., & Sperb, T. M. (2004). The Experimental Context and the Theory of Mind. *Psicologia: Reflexao e Critica, 17*(2), 167-176. doi:http://dx.doi.org/10.1590/S0102-79722004000200005

Dejko, K., Janusz, B., & Treger, B. (2016). Charakterystyka wzorców mentalizacyjnych u rodziców dzieci z trudnościami w realizowaniu zadań rozwojowych okresu latencji—Wyniki analizy jakościowej. [Characteristics of mentalization patterns in parents of children with difficulties in realizing developmental objectives of the latency stage—Qualitative analysis results.]. *Psychiatria Polska, 50*(3), 597-606. doi:10.12740/PP/OnlineFirst/38904

Delgado, J. B., & Moreno, M. R. (2011). Three fundamental discussions in the field of the theory of mind: Theoretical and methodological approaches. *Acta Colombiana de Psicologia, 14*(1), 109-118.

Di Sano, S. (2003). Referential communication in children. *Giornale Italiano di Psicologia, 30*(4), 689-717.

Diges, M., Moreno, A., & Perez-Mata, N. (2010). Suggestion effects in preschoolers: Mentalist skills and individual differences in suggestibility. *Infancia y Aprendizaje / Journal for the Study of Education and Development, 33*(2), 235-254. doi:http://dx.doi.org/10.1174/021037010791114634

Dobnik Renko, B. (2016). Psihološki ocenjevalni postopek za zgodnje odkrivanje avtizma pri malčkih. [Early psychological assessment of autism in toddlers.]. *Anthropos, 48*(3-4), 109-131.

Dziobek, I., & Bolte, S. (2011). [Neuropsychological models of autism spectrum disorders - behavioral evidence and functional imaging]. *Zeitschrift fur Kinder- und Jugendpsychiatrie und Psychotherapie, 39*(2), 79-90. doi:http://dx.doi.org/10.1024/1422-4917/a000094

Echeverria, A. V. (2015). Episodic foresight in preschool age: Equivalence between measures and the relation with future oriented processes and the theory of mind. *Psicologia: Reflexao e Critica, 28*(1), 157-165. doi:http://dx.doi.org/10.1590/1678-7153.201528117

Fang, D., & Qiwei, L. (2005). A Research on the Relationship between Young Children's Theory of Mind and Executive Function. *Psychological Science (China), 28*(3), 544-549.

Fang, F.-X., Wellman, H. M., Liu, Y.-J., Liu, G.-X., & Kang, R. (2009). Longitudinal perspectives: The sequences of theory-of-mind development in Chinese preschoolers. *Acta Psychologica Sinica, 41*(8), 706-714. doi:http://dx.doi.org/10.3724/SP.J.1041.2009.00706

Fujino, H. (2013a). Basic research on developmental disorders and clinical applications: Autism spectrum disorder and theory of mind. *Japanese Journal of Developmental Psychology, 24*(4), 429-438.

Fujino, H. (2013b). Current trends in research on psychosocial interventions for school-aged children with high-functioning autism spectrum disorder: A review. *Japanese Journal of Special Education, 51*(1), 63-72.

Fukuda, Y. (1991). Developmental change of operations in visual image: Comparison between three mountain task and mental rotation task. *Japanese Journal of Educational Psychology, 39*(3), 348-354.

Gallardo-Pauls, B. (2008). [The linguistic marks in the theory of mind: intersubjectivity and enunciation in attention deficit hyperactivity disorder]. *Revista de Neurología, 46 Suppl 1*, S29-35.

Gamannossi, B. A., Bigozzi, L., & Pinto, G. (2007). "So he understood he has been deceived": Language of mind in children's narratives and theory of mind. *Eta Evolutiva, 88*, 95-103.

Garcia-Sanchez, J.-N. (1994). Are the cognitive-sensorimotor disorders observed in autistic specific disorders? *Infancia y Aprendizaje / Journal for the Study of Education and Development, 67-68*, 147-162. doi:http://dx.doi.org/10.1174/021037094321268912

Garcia, M. J. M., Gomez-Becerra, I., Chavez-Brown, M., & Greer, D. (2006). Capture of perspective and theory of the mind: Conceptual and empirical aspects. A complementary and pragmatic proposal. *Salud Mental, 29*(6), 5-14.

Gavazzi, I. G., Ornaghi, V., & Piralli, F. (2011). Theory of mind and children's comprehension of psychological lexicon: Preliminary data from the validation of Test of Emotional Lexicon (TLE). *Psicologia Clinica dello Sviluppo, 15*(1), 257-266.

Ghorbali, A., & Besharat, M. A. (2013). Evaluating the possible relationship between the maternal emotional intelligence and children's theory of mind. *Advances in Cognitive Science, 15*(2[58]; 58), 40-49.

Gorriz, A.-B., Villanueva, L., & Clemente, R.-A. (2009). Mind understanding and communication skills in peer rejected children. *Infancia y Aprendizaje / Journal for the Study of Education and Development, 32*(1), 17-32. doi:http://dx.doi.org/10.1174/021037009787138211

Goukon, A., & Hosokawa, T. (2007). Relation between executive functions and theory of mind: A review. *Japanese Journal of Special Education, 45*(1), 25-33.

Greimel, E., Schulte-Ruther, M., Kamp-Becker, I., Remschmidt, H., Herpertz-Dahlmann, B., & Konrad, K. (2011). [Self-report and parental report of empathy in adolescents with autism]. *Zeitschrift fur Kinder-und Jugendpsychiatrie und Psychotherapie, 39*(2), 113-121. doi:http://dx.doi.org/10.1024/1422-4917/a000097

Hayashi, S., & Takeuchi, Y. (1994). Can preschoolers understand another person's perspective?-A reexamination of Borke's task. *Japanese Journal of Educational Psychology, 42*(2), 129-137.

Hulsken, C. (2000). Young children's difficulty to understand visual ambiguity. *Sprache & Kognition, 19*(1-2), 71-80. doi:http://dx.doi.org/10.1024//0253-4533.19.12.71

Ikuko, S. (2011). Longitudinal Relations among maternal mind-mindedness and children's understanding of other people's beliefs and emotions. *Japanese Journal of Developmental Psychology, 22*(3), 240-250.

Ishisaka, Y. (1998). Cognitive deficits and autism: A consideration based on the module theory. *Japanese Journal of Child and Adolescent Psychiatry, 39*(4), 321-339.

Janke, B. (2010). [Which is the best way for not feeling fear? Young children's strategies to regulate fear]. *Praxis der Kinderpsychologie und Kinderpsychiatrie, 59*(7), 561-575.

Jepsen, J. R. M. (2004). Pervasive developmental disorders in childhood and adolescence; A review of the literature. *Nordisk Psykologi, 56*(1), 50-72.

Jiao, Q. (2001). Research on Theory of Mind in autism. *Chinese Mental Health Journal, 15*(1), 60-62.

Juen, F. (2014). [Facets of diagnostics of mentalization in children]. *Praxis der Kinderpsychologie und Kinderpsychiatrie, 63*(9), 723-729. doi:http://dx.doi.org/10.13109/prkk.2014.63.9.723

Kamio, Y., Toichi, M., Ishisaka, Y., & Zen, C. (1997). Affective understanding in high-functioning autism: Relationship to theory of mind. *Seishin Igaku (Clinical Psychiatry), 39*(10), 1089-1095.

Karakelle, S., & Ertugrul, Z. (2012). Do developmental relationships between theory of mind, language, working memory, and executive functions show differences across early (36-48 months) and late (53-72 months) age groups? *Turk Psikoloji Dergisi, 27*(70), 1-25.

Kazama, M., Hirabayashi, H., Karasawa, M., Tardif, T., & Olson, S. (2013). Ambiguous parenting and four-year-olds' understanding of others: A comparison between mothers in Japan and the U.S. *Japanese Journal of Developmental Psychology, 24*(2), 138-149.

Kinjho, Y., & Maehara, T. (1991). Perceived competence in kindergarten: Its relations to cognitive abilities and ratings by teachers. *Japanese Journal of Educational Psychology, 39*(4), 400-408.

Kisgen, R., & Schleiffer, R. (2002). About the specificity of a theory of mind deficit in autism. *Zeitschrift für Kinder- und Jugendpsychiatrie und Psychotherapie, 30*(1), 29-40. doi:http://dx.doi.org/10.1024//1422-4917.30.1.29

Kissgen, R., & Schleiffer, R. (2002). [Specificity hypothesis of a theory of mind deficit in early childhood autism]. *Zeitschrift fur Kinder-und Jugendpsychiatrie und Psychotherapie, 30*(1), 29-40.

Kocsis, E., Forgacs, A., & Marton, S. (2001). [The role of knowledge about breast-feeding and infant nutrition in selecting the method of feeding]. *Orvosi Hetilap, 142*(51), 2845-2849.

Kristen, S., Thoermer, C., Hofer, T., Aschersleben, G., & Sodian, B. (2006). Validation of the "Theory of Mind" Scale. *Zeitschrift für Entwicklungspsychologie und*

Pädagogische Psychologie, 38(4), 186-195. doi:http://dx.doi.org/10.1026/0049-8637.38.4.186
Kyriakopoulou, N., & Vosniadou, S. (2011). Probing the relation between Theory of Mind and epistemic beliefs. *Psychology: The Journal of the Hellenic Psychological Society, 18*(2), 137-158.
Lara, E. M., & Herrero, P. L. (2004). Considerations on the development of the theory of mind with respect to language. *Revista de Psicologia General y Aplicada, 57*(1), 49-67.
Laszlo, S., Gyorgy, R., & Zoltan, D. (2009). Development of perceptual reversal of ambiguous figures in monolingual and bilingual children. *Erdelyi Pszichologiai Szemle, 10*(2), 121-140.
Lecbych, M., & Hosakova, K. (2014). Interpersonal decentering levels in inpatients with schizophrenia as measured by the Thematic Apperception Test. *Ceskoslovenska Psychologie, 58*(2), 98-106.
Lecce, S., Caputi, M., Pagnin, A., De Rosnay, M., & Pons, F. (2010). Social competence assessed by teachers and parents: The role of theory of mind. *Eta Evolutiva, 96*, 5-14.
Lecciso, F., Petrocchi, S., Sempio, O. L., & Marchetti, A. (2011). A contribution to a new tool of the relationships between affects and mentalization: The Trust Story. *Psicologia Clinica dello Sviluppo, 15*(1), 63-93.
Lee, H., & Tanaka, M. (2011). Narrative study in children with autistic spectrum disorder: A review. *Japanese Journal of Special Education, 49*(4), 377-386.
Lehnhardt, F., Gawronski, A., Volpert, K., Schilbac, L., Tepe, R., Hu, W., & Vogeley, K. (2011). Autism spectrum disorders in adulthood: Clinical and neuropsychological findings of Aspergers syndrome diagnosed late in life. *Fortschritte der Neurologie, Psychiatrie, 79*(5), 290-297. doi:http://dx.doi.org/10.1055/s-0031-1273233
Leithner-Dziubas, K., Bluml, V., Naderer, A., Tmej, A., & Fischer-Kern, M. (2010). [Mentalization and bonding in chronic pelvic pain patients: a pilot study]. *Zeitschrift Fuer Psychosomatische Medizin und Psychotherapie, 56*(2), 179-190.
Li, J., Cheng, J., Feng, L., & Wang, Y.-F. (2012). Relationship of vestibular balancing function to executive function in children with attention-deficit/hyperactivity disorder. *Chinese Mental Health Journal, 26*(6), 410-414.
Li, J., & Su, Y. (2005). The Development of Emotion Understanding in Naxi and Han Children. *Psychological Science (China), 28*(5), 1131-1134.
Li, T., Liu, L., & Zhu, L. (2017). 4-6 year-old children's trust in economic game and its influencing factors. [4-6 year-old children's trust in economic game and its influencing factors.]. *Acta Psychologica Sinica, 49*(1), 17-27. doi:10.3724/SP.J.1041.2017.00017
Li, X.-m., Wang, K., Wu, J.-X., Hong, Y.-f., & Zhao, J.-p. (2012). Correlation between inference processing and theory of mind in children with cerebral palsy. *Chinese Journal of Clinical Psychology, 20*(6), 794-797.
Li, X., Liu, J., Yang, W., Cao, B.-D., He, X.-X., Li, Z.-H., . . . Guo, Y.-Q. (2012). Executive function, theory of mind, and symptom in children with high functioning autism. *Chinese Mental Health Journal, 26*(8), 584-589.

Lin, H.-L., Yang, S.-P., Yang, S.-Y., & Li, P.-H. (2015). A longitudinal study on sentential complements and theory of mind in native Chinese-speaking children and adolescents with autism spectrum disorders. [A longitudinal study on sentential complements and theory of mind in native Chinese-speaking children and adolescents with autism spectrum disorders.]. *Bulletin of Educational Psychology, 46*(3), 401-423.

Liping, G., Xiaolei, W., & Shunmei, W. (2004). An Investigation of Cognitive Development with Upside-down Pictures. *Psychological Science (China), 27*(4), 850-854.

Lo Cricchio, M. G., Liga, F., Ingoglia, S., & Lo Coco, A. (2012). Emotional detachment and separation in parent-adolescent relationship. *Psicologia Clinica dello Sviluppo, 16*(2), 399-419.

Lockl, K., Schwarz, S., & Schneider, W. (2004). Language and theory of mind: A longitudinal study of three- to four-year-olds. *Zeitschrift für Entwicklungspsychologie und Pädagogische Psychologie, 36*(4), 207-220. doi:http://dx.doi.org/10.1026/0049-8637.36.4.207

Longobardi, E., Ferrari, F., Renna, M., & Spataro, P. (2013). The role of syntactic and narrative structure in the production of psychological lexicon: A comparison between fictional and personal stories at school age. *Rassegna di Psicologia, 30*(2), 9-28.

Loureiro, C. P., & de Hollanda Souza, D. (2013). The relationship between theory of mind and moral development in preschool children. *Paideia, 23*(54), 93-101. doi:http://dx.doi.org/10.1590/1982-43272354201311

Lu, H.-J., & Su, Y.-J. (2009). Relations between judgment of others' memory and theory of mind in preschoolers. *Acta Psychologica Sinica, 41*(2), 135-143. doi:http://dx.doi.org/10.3724/SP.J.1041.2009.00135

Malo-Machado, P., Verissimo, M., & Rebelo, A. (2013). Emotional knowledge: Differences in function of gender and age. *Psicologia Educacao Cultura, 17*(1), 10-28.

Maluf, M. R., Gallo-Penna, E. C., & dos Santos, M. J. (2011). Attribution of mental states and conversational awareness: A study with preschool students. *Paideia, 21*(48), 41-50. doi:http://dx.doi.org/10.1590/S0103-863X2011000100006

Margoni, F. (2015). Ipotesi sullo sviluppo del ragionamento morale basato sulle intenzioni. [Hypotheses on the development of an intention-based moral reasoning.]. *Infanzia e Adolescenza, 14*(3), 218-231.

Margoni, F., Scarpa, L., & Surian, L. (2017). L’acquisizione del giudizio morale basato sulle intenzioni nei bambini con sviluppo tipico o con autismo. [The acquisition of intent-based moral judgment in children with typical development and children with autism.]. *Psicologia Clinica dello Sviluppo, 21*(1), 51-77.

Marsova, K., Mezulanikova, K., D'Souza, H., & Lacinova, L. (2014). Theory of mind in children in pre-school age: Connection with institutional care and type of education. *Ceskoslovenska Psychologie, 58*(1), 2-13.

Martin, E., & Munoz de Bustillo, M. C. (2009). [A contextual analysis of peer acceptance and peer rejection at school]. *Psicothema, 21*(3), 439-445.

Martin Garcia, M. J., Becerra, I. G., & Garro Espin, M. J. (2012). Theory of Mind in a child with autism: How to train her? *Psicothema, 24*(4), 542-547.

Mashhadi, A., Juzdani, M. H., & Borzabadi, H. H. (2011). Theory of mind development and its relation to social skills in children and adolescence with visual impairment. *Journal of Iranian Psychologists, 7*(27), 219-230.

Mazzoni, G., & Chiesi, F. (1997). Theory of mind and deceit: The role of emotional involvement. *Eta Evolutiva, 58,* 36-49.

Mei-Fang, W., & Hui-Chang, C. (2009). The development of trait inference at behavioral and psychological levels. *Acta Psychologica Sinica, 41*(10), 947-957. doi:http://dx.doi.org/10.3724/SP.J.1041.2009.00947

Meshcheryakov, B., Dubovskaya, I., & Dubyaga, E. (2011). Theory of mind without the concept of "mind" itself. *Cultural-Historical Psychology, 2*, 66-73.

Miranda-Casas, A., Baixauli-Fortea, I., Colomer-Diago, C., & Rosello-Miranda, B. (2013). [Autism and attention deficit hyperactivity disorder: similarities and differences in executive functioning and theory of mind]. *Revista de Neurología, 57 Suppl 1*, S177-184.

Misailidi, P., & Kosta, A. (2016). Comprehension of emotion metaphors and theory of mind in children aged 5–9 years. [Comprehension of emotion metaphors and theory of mind in children aged 5–9 years.]. *Hellenic Journal of Psychology, 13*(2), 131-155.

Miyamoto, Y. (1998). [Young children's representational theory of mind in understanding masked facial expression]. *Shinrigaku Kenkyu - Japanese Journal of Psychology, 69*(4), 271-278.

Mizokawa, A., & Koyasu, M. (2011). Understanding false beliefs, hidden emotions, and social interactions among five- and six-year olds. *Japanese Journal of Developmental Psychology, 22*(2), 168-178.

Morino, M. (2005). Preschoolers' Theory of Mind, Understanding of Emotions, and Interactions with Peers. *Japanese Journal of Developmental Psychology, 16*(1), 36-45.

Muller-Gottken, T., White, L. O., von Klitzing, K., & Klein, A. M. (2014). [Maternal reflective functioning as a predictor of therapeutic success of psychoanalytic short-term therapy for children aged 4 to 10 years]. *Praxis der Kinderpsychologie und Kinderpsychiatrie, 63*(10), 795-811. doi:http://dx.doi.org/10.13109/prkk.2014.63.10.795

Murakami, T., Nishimura, T., & Sakurai, S. (2014). Relation between cognitive/emotional empathy and prosocial and aggressive behaviors in elementary and middle school students. *Japanese Journal of Developmental Psychology, 25*(4), 399-411.

Nieri, L. P. (2017). Relación entre la sensibilidad paterna y los estilos de apego, la personalidad y la capacidad empática, según variables sociodemográficas. [Relationship between parental sensitivity and attachment styles, personality and empathic capacity, according to sociodemographic variables.]. *Psicología desde el Caribe, 34*(1), 1-27.

Nunez, M., & Riviere, A. (2007). An empirical re-evaluation of some inferential assumptions in the false belief paradigm. *Infancia y Aprendizaje / Journal for the Study of Education and Development, 30*(3), 289-308. doi:http://dx.doi.org/10.1174/021037007781787507

Okada, T. (2012). [Phenotype and cognitive neurobiology of pervasive developmental disorders]. *Nihon Shinkei Seishin Yakurigaku Zasshi, 32*(1), 19-24.
Okuda, K., & Inoue, M. (2000). A behavior analytic view of teaching "theory of mind" to children with autism: Stimulus control and generalization on false belief tasks. *Japanese Psychological Review, 43*(3), 427-442.
Ornaghi, V., Gavazzi, I. G., & Zanetti, M. A. (2010). Mental state talk and theory of mind: A study with school aged children. *Eta Evolutiva, 97*, 54-71.
Otsui, K., & Tanaka-Matsumi, J. (2007). Preschoolers's social skills as predicted by perspective taking, regulation of emotion, and social problem solving. *The Japanese Journal of Social Psychology, 22*(3), 223-233.
Ozbaran, B., Kose, S., & Erermis, S. (2009). Social cognition in pervasive developmental disorders. *Klinik Psikofarmakoloji Bulteni / Bulletin of Clinical Psychopharmacology, 19*(3), 322-331.
Padilla-Mora, M., Rodriguez-Villagra, O., & Fornaguera-Trias, J. (2009). Interactions between false-belief understanding and verbal ability development: Sex differences in preschool children. *Interdisciplinaria Revista de Psicologia y Ciencias Afines, 26*(2), 317-344.
Padilla, M. L. (1995). Cognitive basis of empathy: A developmental study. *Revista Latina de Pensamiento y Lenguaje, 3*(2), 173-196.
Pascual, B., Aguado, G., & Sotillo, M. (2006). Approximation to different theoretical perspectives of "theory of mind". *Revista de Logopedia, Foniatria y Audiologia, 26*(3), 154-164. doi:http://dx.doi.org/10.1016/S0214-4603%2806%2970208-X
Pavarini, G., & de Hollanda Souza, D. (2010). Theory of mind, empathy and prosocial motivation in preschool children. *Psicologia em Estudo, 15*(3), 613-622.
Pavarini, G., Loureiro, C. P., & de Hollanda Souza, D. (2011). Emotion understanding, social acceptance and evaluation of behavioral attributes in school-age children. *Psicologia: Reflexao e Critica, 24*(1), 135-143. doi:http://dx.doi.org/10.1590/S0102-79722011000100016
Pereda, N., Arch, M., Guerra-Gonzalez, R., Llerena, G., Berta-Alemany, M., Saccinto, E., & Gascon, E. (2012). Knowledge and beliefs on child sexual abuse by Spanish university students. *Anales de Psicologia, 28*(2), 524-531.
Perez-Albeniz, A., & de Paul, J. (2005). Empathy in high- and low-risk students for child physical abuse. *Psicologia Conductual Revista Internacional de Psicologia Clinica de la Salud, 13*(1), 5-18.
Perez-Leroux, A. (2008). Subjunctive and awareness of subjectivity in children's acquisition of language and theory of mind. *Revista de Logopedia, Foniatria y Audiologia, 28*(2), 90-98. doi:http://dx.doi.org/10.1016/S0214-4603%2808%2970048-2
Piekny, J., Thomsen, T., Schuchardt, K., Lessing, N., Greve, W., & Mähler, C. (2017). Kognitive Kompetenzen und kognitive Bewältigungsstrategien im Vor- und Grundschulalter. Bedingungen, Wechselwirkungen und Entwicklungsverläufe. [Cognitive competencies and coping in preschool and primary school: Developmental precursors, interactions, and relations.]. *Kindheit und Entwicklung: Zeitschrift für Klinische Kinderpsychologie, 26*(1), 28-38. doi:10.1026/0942-5403/a000214

Pokrajac-Bulian, A., & Tatalovic, S. (2001). A Multidimensional Approach to Measuring of Sex and Age Differences in Empathy. *Psihologijske Teme, 10*, 77-87.

Putko, A. (2004). Theory of mind and the breadth of older siblings effect and its relations to pretend play and inhibitory control. *Studia Psychologiczne, 42*(2), 69-80.

Putko, A. (2006). The role of syntax and executive function in the transition from implicit and explicit mental competence. *Studia Psychologiczne, 44*(2), 55-68.

Quintanilla, L., & Sarria, E. (2003). Realism, Animism and Theory of Mind: Cultural and universal characteristics of mental knowledge. *Estudios de Psicologia, 24*(3), 313-335. doi:http://dx.doi.org/10.1174/021093903770411201

Rakickiene, L., & Girdzijauskiene, S. (2012). The development of executive function. *Psichologija*, *45*, 42-54.

Reichelova, E. (2012). Predictive validity of traditional and dynamic testing of intelligence. *Psychologia a Patopsychologia Dietata, 46*(4), 336-352.

Resch, F. (1999). Representation and structure in a developmental psychopathology perspective. *Praxis der Kinderpsychologie und Kinderpsychiatrie, 48*(8), 556-563.

Riquelme, E. H., Henriquez, C., & Alvarez, B. (2003). Relationship Between Attachment Styles and Theory of Mind. *Psykhe: Revista de la Escuela de Psicologia, 12*(1), 73-81.

Ruiz-Murugarren, S., Garcia-Ramos, P., & Lahera, G. (2011). Social cognition deficit in bipolar disorder: Relevance and rehabilitation strategies. *Revista Colombiana de Psiquiatria, 40*(1), 99-114.

Saiz Manzanares, M. C., Carbonero-Martin, M. A., & Lucas, V. F. (2010). Types of responses in 4-5-year-old children in conservation, classification, and theory of mind tasks. *Psicothema, 22*(4), 772-777.

Sampaio, L. R., Bagano Guimaraes, P. R., dos Santos Camino, C. P., Formiga, N. S., & Menezes, I. G. (2011). Studies on the dimensionality of empathy: translation and adaptation of the Interpersonal Reactivity Index (IRI). *Psico, 42*(1), 67-76.

Santelli, E., & Pinelli, M. (2006). Is there a link between attachment patterns and Theory of Mind skills? *Psicologia Dell' Educazione E Della Formazione, 8*(3), 353-373.

Schmitz, M., & Röhr-Sendlmeier, U. M. (2016). Sozial-Emotionale Kompetenz. Erprobung eines Kurzzeittrainingsprogramms für Kinder in vierten Klassen. [Social–emotional competence: Evaluation of a short-term training program for elementary school children.]. *Kindheit und Entwicklung: Zeitschrift für Klinische Kinderpsychologie, 25*(2), 114-121. doi:10.1026/0942-5403/a000194

Schwenck, C., Schmitt, D., Sievers, S., Romanos, M., Warnke, A., & Schneider, W. (2011). [Cognitive and emotional empathy in children with ADHD and conduct disorder]. *Zeitschrift fur Kinder-und Jugendpsychiatrie und Psychotherapie, 39*(4), 265-276. doi:http://dx.doi.org/10.1024/1422-4917/a000118

Sempio, O. L., Cavalli, G., Fabio, R. A., & Marchetti, A. (2009). Italian validation of the "Vocal Test about Mental States" (TVSM), A new advanced theory of mind task for school age children. *Ricerche di Psicologia, 32*(1), 111-134.

Serra, M., Henríquez, L., Lorenzo, T., & Duque, N. (2017). La memoria de trabajo, capacidad y procedimientos, en el trastorno específico del lenguaje: Análisis visual de eventos y su expresión oral. [Working memory, ability and procedures in specific language impairment: Visual analysis of events and their oral

expression.]. *Revista de Logopedia, Foniatria y Audiologia, 37*(1), 14-29. doi:10.1016/j.rlfa.2016.05.003
Shao, W.-T., Xu, S., Feng, H., Jin, S.-J., Zhu, S.-S., Li, D., . . . Xu, X.-J. (2017). Revision of the Theory of Mind Tests for Children with Autism Spectrum Disorder. [Revision of the Theory of Mind Tests for Children with Autism Spectrum Disorder.]. *Chinese Mental Health Journal, 31*(2), 144-149.
Shi, B., & Su, Y. (2007). Children's deceptive behavior and related social characteristics. *Acta Psychologica Sinica, 39*(1), 111-117.
Shiwa, T. (2010). The distinction between young children's understanding of intentions and false-beliefs on a word-learning task. *Japanese Journal of Developmental Psychology, 21*(3), 244-253.
Shiwa, T. (2011). The obstructive effect of visual representations on the dimensional change card sort task in young children. *Japanese Journal of Developmental Psychology, 22*(1), 11-21.
Sotillo, M., & Riviere, A. (2001). When children use words to deceive. Lying as an instrument for developing mentalistic inference abilities. *Infancia y Aprendizaje / Journal for the Study of Education and Development, 24*(3), 291-305. doi:http://dx.doi.org/10.1174/021037001316949239
Spotakova, M., Filippova, E., & Dockal, V. (2003). Some aspects of theory of mind in children with cerebral palsy II. Relations of false beliefs with linguistic and cognitive abilities. *Psychologia a Patopsychologia Dietata, 38*(1), 3-20.
Steerneman, P., & Muris, P. (1997). Deficits in the understanding of false belief: Specific for autism and related disorders? *Gedrag & Gezondheid: Tijdschrift voor Psychologie en Gezondheid, 25*(5), 258-264.
Suzuki, A. (2014). Young children's understanding of morally relevant false belief. *Japanese Journal of Developmental Psychology, 25*(4), 379-386.
Suzuki, T., Hirano, M., Kita, Y., Goukon, A., Noguchi, K., & Hosokawa, T. (2013). Self-recognition in a child with high-functioning autism. *Japanese Journal of Special Education, 51*(2), 105-113.
Tallandini, M. A., & Palmerone, E. (1987). Interpersonal perception in the child. *Eta Evolutiva, 26*, 104-112.
Tanaka, M. (2013). Causal attributions in children and adults who have been diagnosed as having attention deficit/hyperactivity disorder (AD/HD): A review. *Japanese Journal of Educational Psychology, 61*(2), 193-205. doi:http://dx.doi.org/10.5926/jjep.61.193
Taubner, S., Hasper, F., & Wahl, K. (2013). [Xenophobia and experienced abandonment--a depth-hermeneutic analysis of a single case]. *Praxis der Kinderpsychologie und Kinderpsychiatrie, 62*(10), 758-777.
Toyama, K. (2007). Examining theory-of-mind tasks with Japanese children: The Wellman and Liu tasks. *Japanese Journal of Educational Psychology, 55*(3), 359-369.
Toyama, K. (2011). Relation between 5- to 6-year-old children theory of mind and their mothers' use of mind-related words. *Japanese Journal of Educational Psychology, 59*(4), 427-440. doi:http://dx.doi.org/10.5926/jjep.59.427

Wang, S., & Su, Y. (2013). From understanding to utilizing: Theory of Mind and children's distributive justice in different contexts. *Acta Psychologica Sinica, 45*(11), 1242-1250. doi:http://dx.doi.org/10.3724/SP.J.1041.2013.01242

Wu, W.-J., Zhang, L., Feng, T.-Y., & Li, H. (2008). The effect of hot executive function on children's test with the standard windows task. *Acta Psychologica Sinica, 40*(3), 319-326. doi:http://dx.doi.org/10.3724/SP.J.1041.2008.00319

Xiao, X., Yang, N., Qian, L.-q., & Zhou, S.-j. (2014). Pretend playing training improves theory of mind in children with autism. *Chinese Journal of Clinical Psychology, 22*(4), 742-745.

Xiaoshuang, S., & Yanjie, S. (2003). Verifying the cognitive model of theory of mind. *Acta Psychologica Sinica, 35*(1), 56-62.

Xu, W. (2015). Relationship between children's theory of mind and moral judgement. *Chinese Journal of Clinical Psychology, 23*(1), 67-70.

Yagmurlu, B., Sanson, A., & Koymen, S. (2005). Effects of parenting and child temperament on the development of prosocial behavior: The mediating role of theory of mind. *Turk Psikoloji Dergisi, 20*(55), 1-24.

Yan-Yan, L., & Biao, S. (2006). Mothers' Parenting Style and the Development of Children's Theory of Mind. *Chinese Mental Health Journal, 20*(1), 5-9.

Yanjie, S., & Ke, W. (2004). Influence of Animation on Autistic Children's False Belief Understanding. *Chinese Mental Health Journal, 18*(3), 147-150.

Yazdi, S. A. A., & Nikkhah, P. (2011). Role of conversational awareness of children's performance in false belief tasks. *Psychological Research, 14*(1), 79-100.

Yiwen, W., Chongde, L., & Wenxin, Z. (2004). A Study on the Relation of Children's Aggression to Their Theory-of-mind. *Psychological Science (China), 27*(3), 540-544.

Yiyuan, L., Ruiming, W., Xingwang, H., Hong, L., & Zelazo, P. (2006). The Development of Executive Function in Deaf Children: Comparing with Normal Children. *Acta Psychologica Sinica, 38*(3), 356-364.

Yoshii, H., & Yoshimatsu, Y. (2003). Self-Understanding, Understanding of Others, and Affective Understanding in Adolescents with Autism. *Japanese Journal of Special Education, 41*(2), 217-226.

Zhang, L.-J., & Wu, N. (2010). The relation between verbal skills and theory of mind in preschoolers: A short-term longitudinal study. *Acta Psychologica Sinica, 42*(12), 1166-1174. doi:http://dx.doi.org/10.3724/SP.J.1041.2010.01166

Zhang, Q.-Q., Zhou, S.-J., Huang, Q.-P., & Mo, X.-Z. (2013). Desire understanding of autistic children in China. *Chinese Journal of Clinical Psychology, 21*(6), 924-927.

Zhang, Q., Wang, M.-f., & Bai, W. (2011). Development of preschoolers' episodic feature thinking and its relationship with theory of mind. *Chinese Journal of Clinical Psychology, 19*(3), 377-379.

Zhang, W.-j., & Deng, L.-f. (2016). Child sexual abuse prevention and theory of mind in preschool-aged children. [Child sexual abuse prevention and theory of mind in preschool-aged children.]. *Chinese Journal of Clinical Psychology, 24*(1), 28-31.

Zhang, W., & Lin, C. (1999). The development of children's social perspective-taking and its relation to their peer interaction. *Acta Psychologica Sinica, 31*(4), 418-427.

Zhang, W., & Zheng, J. (1999). The development of children's social perspective taking and the differences between perspective taking subtypes. *Psychological Science (China), 22*(2), 116-119.

Ziegenhain, U. (1999). [Contribution of maternal sensitivity in transgenerational promotion of binding quality]. *Praxis der Kinderpsychologie und Kinderpsychiatrie, 48*(2), 86-100.

Zrinscak, I., Simlesa, S., & Jagodic, G. K. (2014). Individual and family factors of theory-of-mind understanding among preschool children. *Hrvatska Revija Za Rehabilitacijska Istrazivanja, 50*(2), 61-79.
